# Supplementary material for: Whole-genome resequencing reveals world-wide ancestry and adaptive introgression events of domesticated cattle in East Asia
Source: Nat Commun. 2018 Jun 14;9:2337. doi: 10.1038/s41467-018-04737-0 (PMC6002414; doi:10.1038/s41467-018-04737-0)
Supplement: Supplementary file 1 — Supplementary Information [file 41467_2018_4737_MOESM1_ESM.pdf]

Whole-genome resequencing reveals world-wide ancestry and multiple adaptive introgression events of domesticated cattle in East Asia

Chen *et al*.

Supplementary Information

Supplementary Figures	Page
Supplementary Figure 1	3
Supplementary Figure 2	4
Supplementary Figure 3	5
Supplementary Figure 4	6
Supplementary Figure 5	7
Supplementary Figure 6	8
Supplementary Figure 7	9
Supplementary Figure 8	10
Supplementary Figure 9	11
Supplementary Figure 10	12
Supplementary Figure 11	13
Supplementary Figure 12	14
Supplementary Figure 13	15
Supplementary Figure 14	16
Supplementary Figure 15	17-22
Supplementary Figure 16	23
Supplementary Figure 17	24-29
Supplementary Figure 18	30
Supplementary Figure 19	31
Supplementary Figure 20	32
Supplementary Figure 21	33
Supplementary Figure 22	34

Supplementary Tables	
Supplementary Table 1	35-38
Supplementary Table 2	39
Supplementary Table 3	40
Supplementary Table 4	41
Supplementary Table 5	42
Supplementary Table 6	43
Supplementary Table 7	44
Supplementary Table 8	45-47
Supplementary Table 9	48
Supplementary Table 10	49
Supplementary Table 11	50
Supplementary Table 12	51

Supplementary Table 13 52-53
Supplementary Table 14 54
Supplementary Table 15 55
Supplementary Table 16 56
Supplementary Table 17 57
Supplementary Table 18 58
Supplementary Table 19 59
Supplementary Table 20 60
Supplementary Table 21 61-87
Supplementary Table 22 88
Supplementary Table 23 89
Supplementary Table 24 90
Supplementary Table 25 91
Supplementary Table 26 92
Supplementary Table 27 93
Supplementary Table 28 121
Supplementary Table 29 122
Supplementary Table 30 123

Supplementary Notes

Supplementary Note 1 124
Supplementary Note 2 125
Supplementary Note 3 127
Supplementary Note 4 128
Supplementary Note 5 130
Supplementary Note 6 134

Supplementary Reference 135

Supplementary Figures

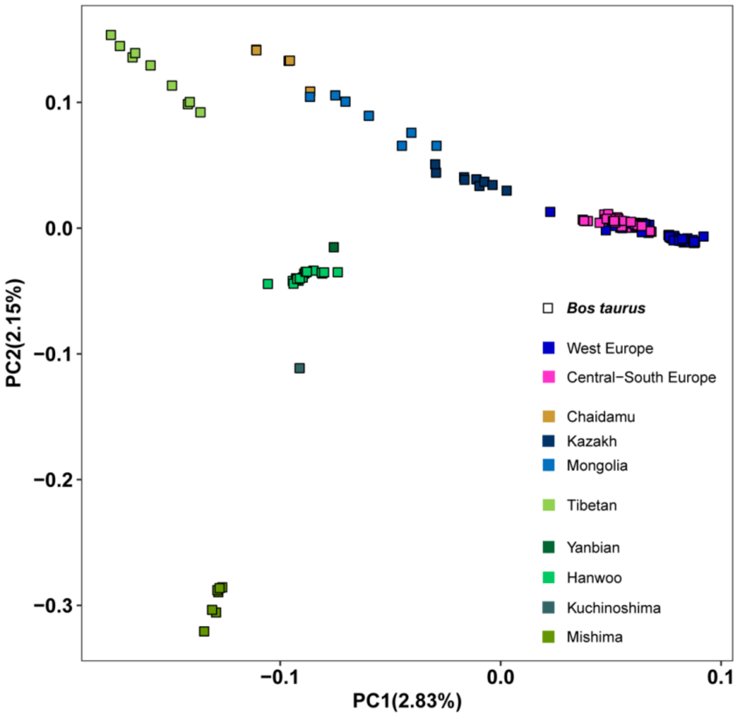

Supplementary Figure 1. Principal component analysis (PCA) of taurine cattle, with PC1 plotted against PC2. In total, 142 individuals from 21 taurine cattle breeds were used for the PCA analysis, including six West European breeds (Red Angus, Angus, Devon, Hereford, Holstein, and Jersey), seven Central-South European breeds (Maine Anjou, Salers, Limousin, Charolais, Piedmontese, Gelbvieh, and Simmental), three breeds from Northwest China (Chaidamu, Kazakh, and Mongolian), four East Asian breeds (Hanwoo, Kuchinoshima, Mishima, and Yanbian), and Tibetan cattle. The first component was driven by difference between European and East Asian taurine cattle, and the second component was by a split of Northeast Asian cattle (Hanwoo, Kuchinoshima, Mishima, and Yanbian cattle).

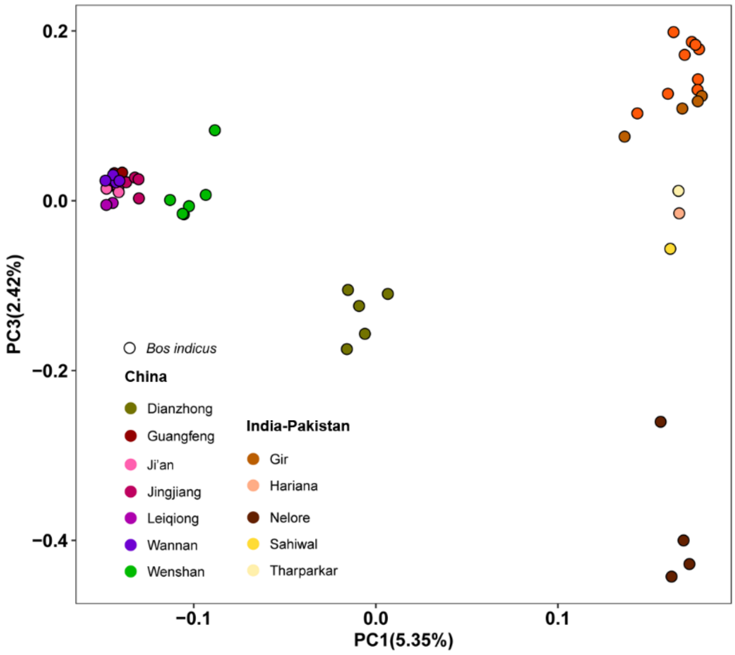

Supplementary Figure 2. Principal component analysis (PCA) of indicine cattle, with PC1 plotted against PC3. In total, 50 individuals from 13 indicine cattle breeds were used for PCA analysis, including six Indian indicine breeds (Hariana, Sahiwal, Tharparkar, Brahman, Gir, and Nelore) and seven Chinese indicine breeds (Guangfeng, Ji'an, Jingjiang, Leiqiong, Wannan, Dianzhong, and Wenshan). Please note that we excluded the hybrid individuals of two Wenshan and one Dianzhong cattle. The first component was driven by differences between Indian and Chinese indicine cattle, and the second component by a split of different Indian indicine cattle.

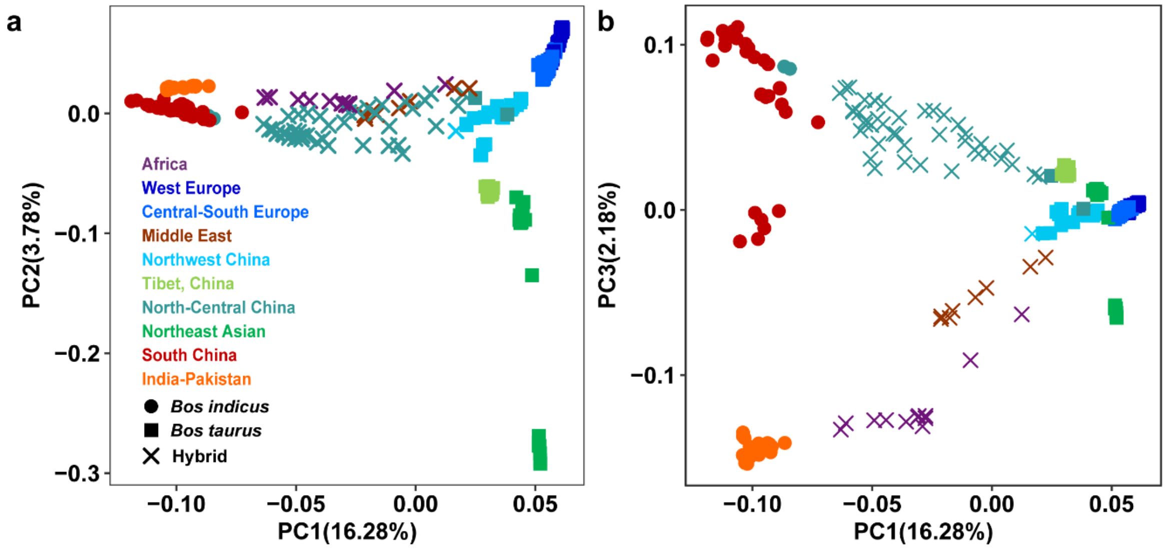

Supplementary Figure 3. Principal component analysis (PCA) showing PC1 against PC2 (a) and PC1 against PC3 (b) by using ANGSD.

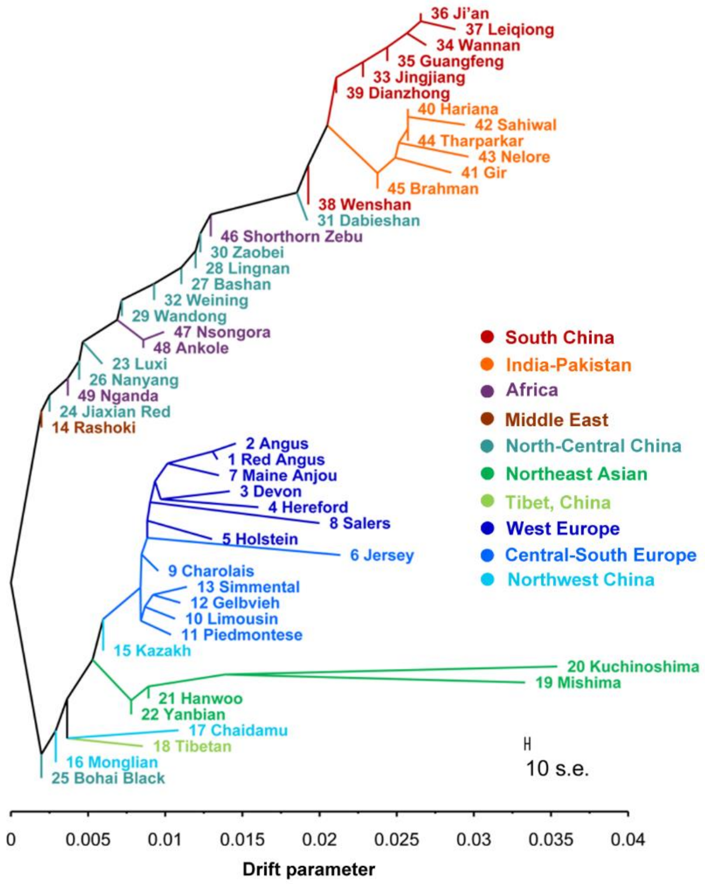

Supplementary Figure 4. TreeMix relationships between 48 cattle breeds. Breeds 1-13 and 16-22 are taurine origin; 37-44 indicine origin; 14-15, 223-36, and 45-47 hybrid origin. The window size of 1000 was used to account for linkage disequilibrium (-k) and "-global" to generate the ML tree.

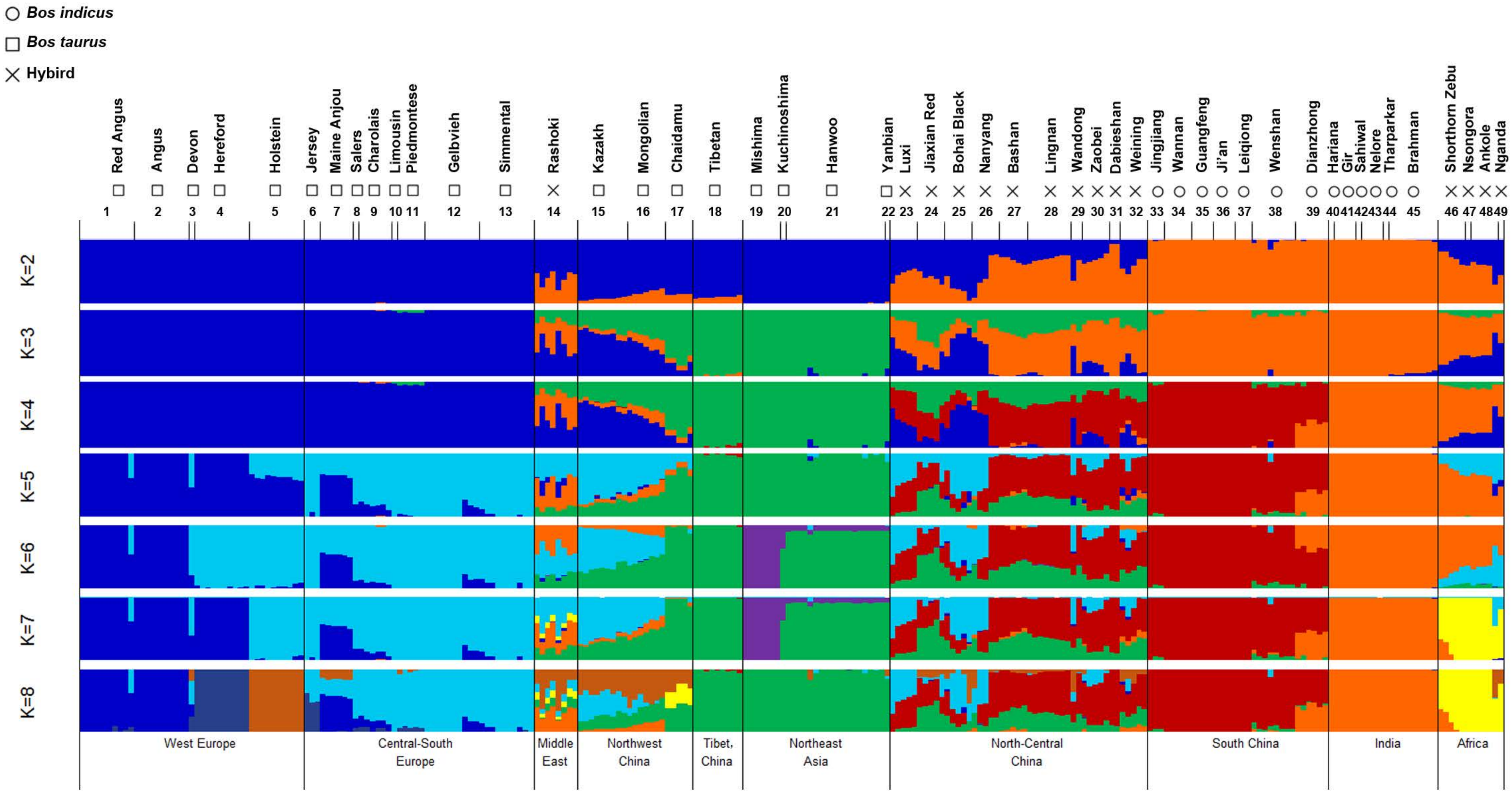

Supplementary Figure 5. **Model-based clustering of cattle breeds using the program ADMIXTURE with *K* = 2 to 8 (plotted in R).**

The breed name associated with serial number is listed in the "Figure legend" column in Supplementary Table 2. "□" represent *Bos taurus*, "○" represents *Bos indicus*, "×" represent hybrid between *Bos taurus* and *Bos indicus*. We found strong support for two population subdivisions (*K* = 2), which is consistent with the deep division of the *Bos taurus* and *Bos indicus* lineages. The population subdivision at *K* = 3 reproduces the second PCA coordinated by separating the European cattle (West Europe and Central-South Europe) from East Asia cattle (Tibetan and Northeast Asian cattle). The Chinese indicine cattle (Leiqiong, Jingjiang, Guangfeng, Ji'an, Wannan, and Wenshan) was clearly separated from the Indian indicine cattle (Hariana, Sahiwal, Tharparkar, Brahman, Gir, and Nelore) at *K* = 4. Population subdivisions at *K* = 5 produced different *Bos taurus* groups and revealed a strong clustering of taurine cattle into three main clusters: European taurine ancestry (Hereford, Holstein, and Angus), Eurasian taurine ancestry (Piedmontese, Gelbvieh, Limousin, Simmental, Kazakh, Mongolian, and Chaidamu), and East Asian taurine ancestry (Tibetan, Mishima, Kuchinoshima, Hanwoo, and Yanbian).

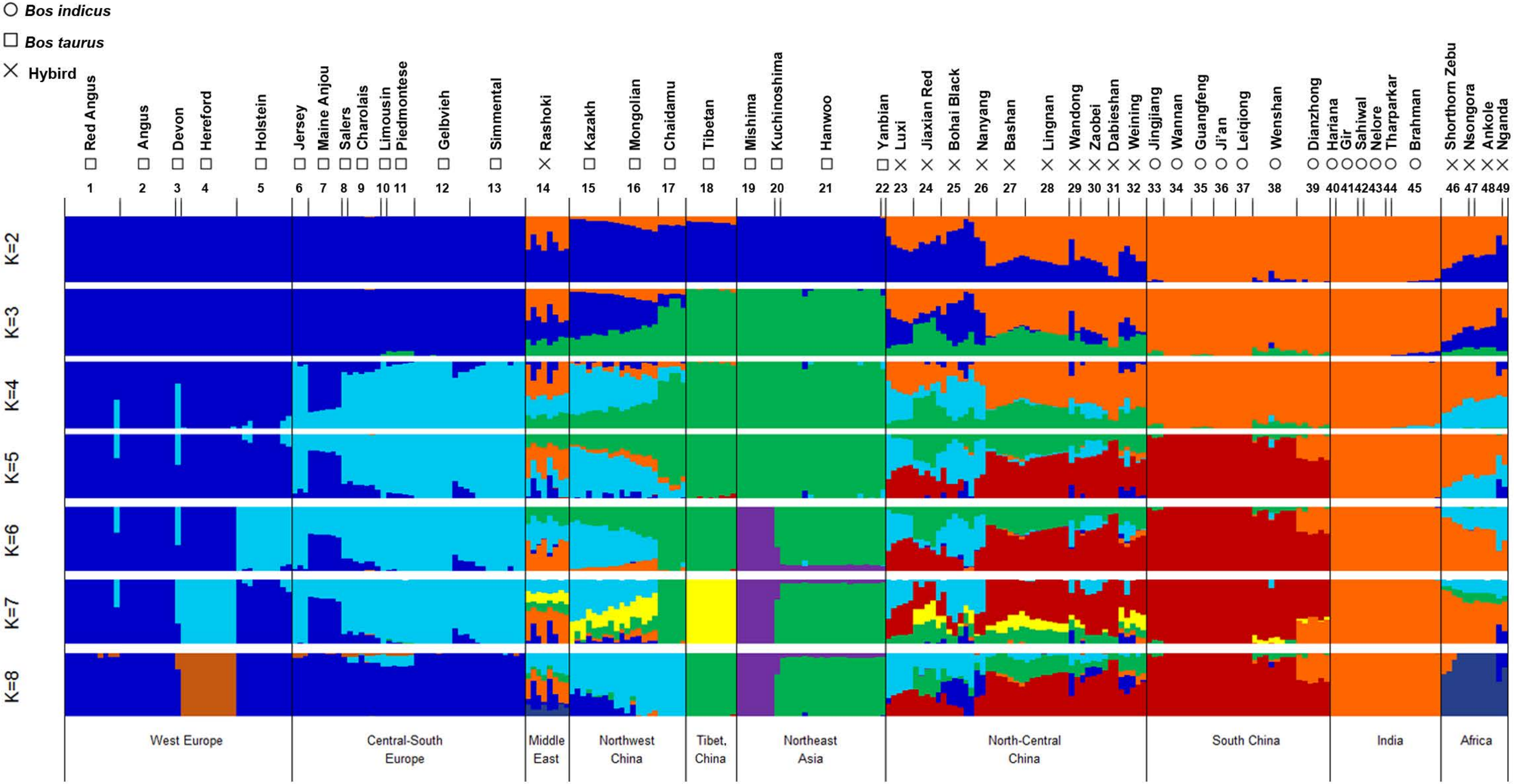

Supplementary Figure 6. Model-based clustering of cattle breeds using the program NGSadmix with *K* = 2 to 8 (plotted in R).

The breed name associated with earial number is listed in the "Figure legend" column in Supplementary Table 2. "□" represent *Bos taurus*, "○" represents *Bos indicus*, "×" represent hybrid between *Bos taurus* and *Bos indicus*. We found strong support for two population subdivisions (K = 2), which is consistent with the deep division of the *Bos taurus* and *Bos indicus* lineages. The population subdivision at *K* = 3 reproduces the second PCA coordinated by separating the European cattle (West Europe and Central-South Europe) from East Asia cattle (Tibetan and Northeast Asian cattle). Population subdivisions at *K* = 4 produced different *Bos taurus* groups and revealed a strong clustering of taurine cattle into three main clusters: European taurine ancestry (Hereford, Holstein, and Angus), Eurasian taurine ancestry (Piedmontese, Gelbvieh, Limousin, Simmental, Kazakh, Mongolian, and Chaidamu), and East Asian taurine ancestry (Tibetan, Mishima, Kuchinoshima, Hanwoo, and Yanbian). The Chinese indicine cattle (Leiqiong, Jingjiang, Guangfeng, Ji'an, Wannan, and Wenshan) was clearly separated from the Indian indicine (Hariana, Sahiwal, Tharparkar, Brahman, Gir, and Nelore) at *K* = 5.

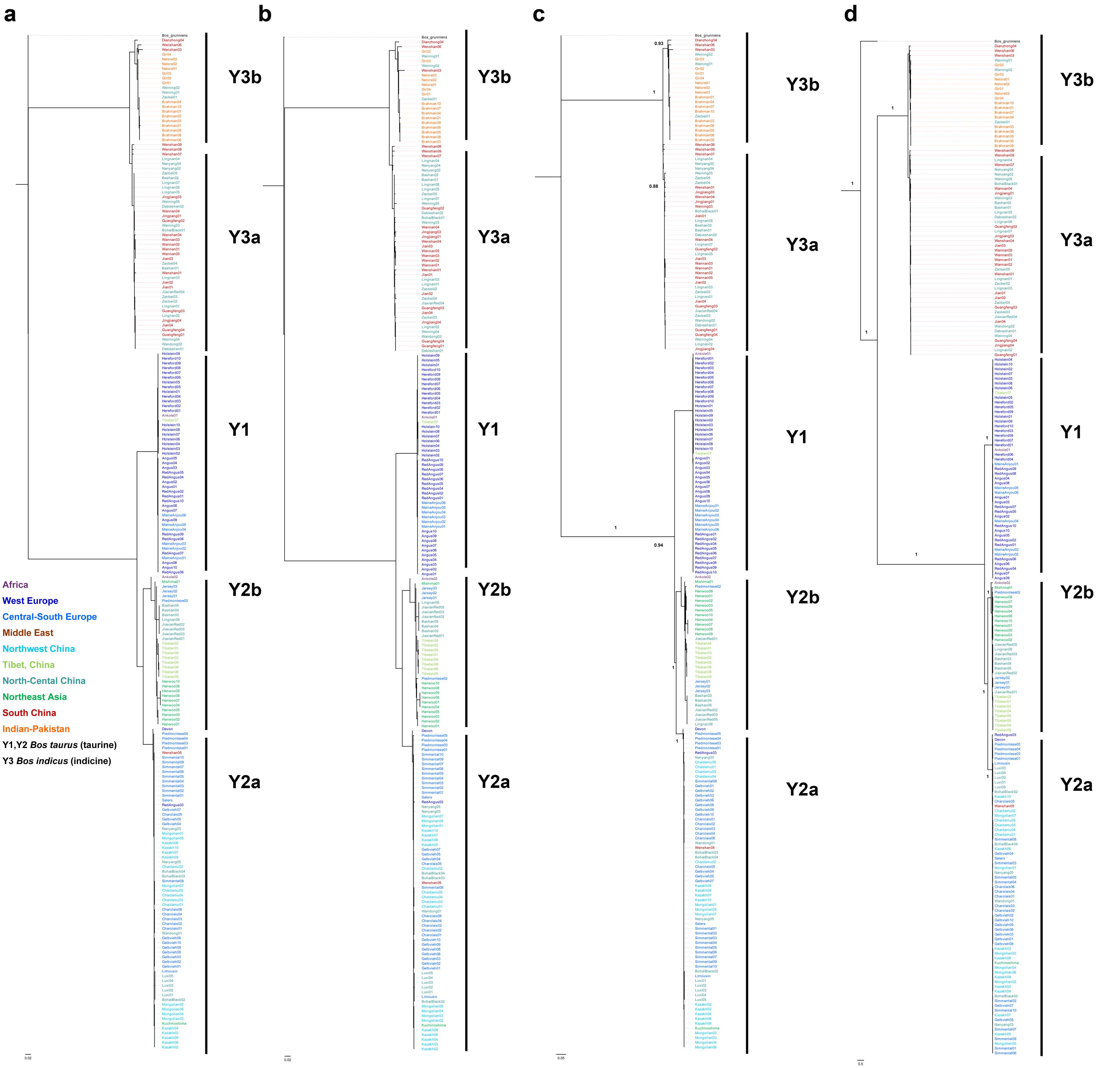

Supplementary Figure 7. Phylogenetic tree of 214 Y chromosomes using 745 SNPs
(a) Rooted maximum parsimony tree from PHYLIP using non-imputed sequences. (b) Rooted maximum parsimony tree from PHYLIP using imputed sequences. (c) Maximum-likelihood (ML) tree from MEGA 7.0. ML bootstrap values are shown on branches. (d) MCMC tree from Beast. Scale bars are based on substitutions per site. Bayesian posterior probability are shown on branches. Colors reflect geographic regions of sampling. Y1 and Y2 belong to *Bos taurus*, Y3 belongs to *Bos indicus.*

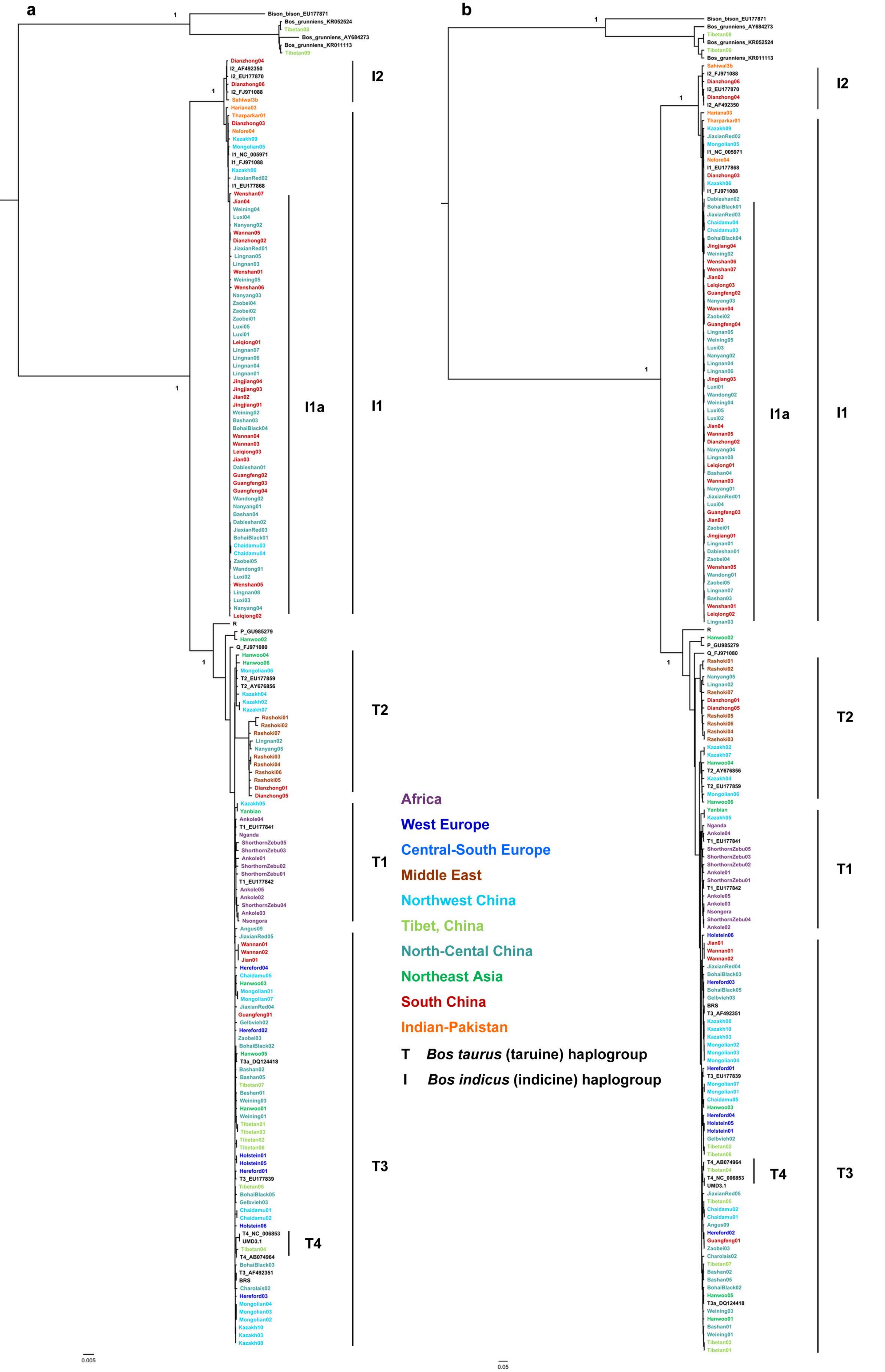

Supplementary Figure 8. Phylogenetic trees inferred from whole mitochondrial genomes

Maximum-likelihood tree from PhyML(a) and MCMC tree from Beast (b). Scale bars are based on substitutions per site. These trees encompasse 171 sequences and are rooted using three published yak sequences and a sequence from American bison. The black lines represent published reference sequences. T1,T2,T3, and T4 belong to *Bos taurus*, I1 and I2 belong to *Bos indicus*.

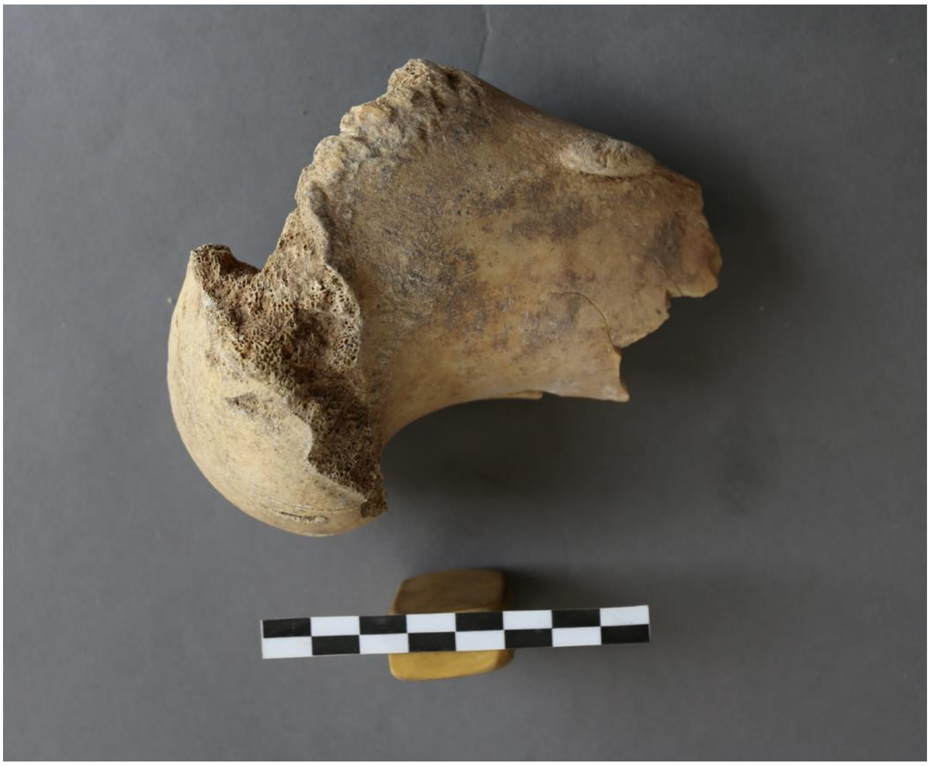

Supplementary Figure 9. Humerus (right) of the Shimao05 specimen used for direct dating.

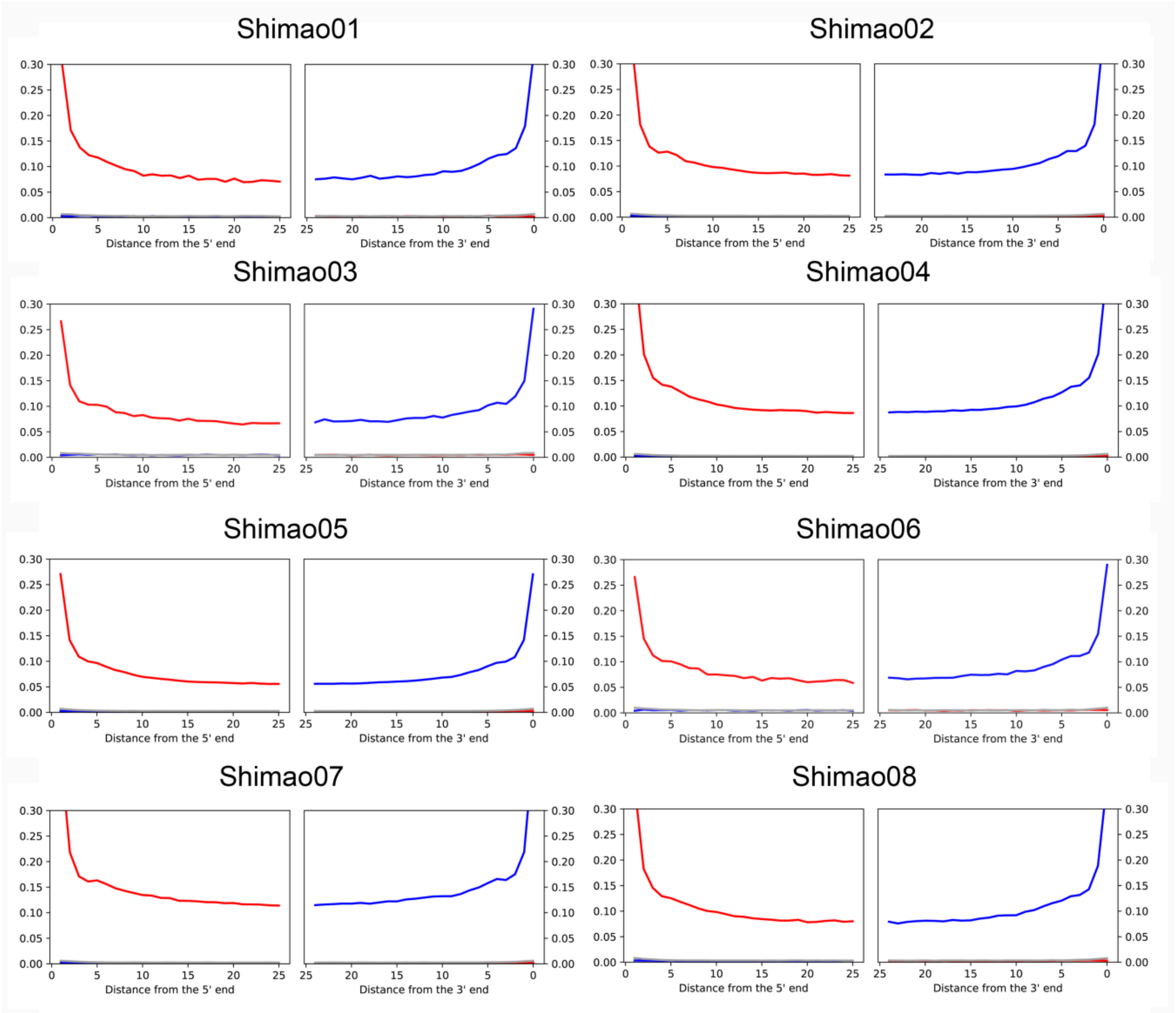

Supplementary Figure 10. Nucleotide mis-incorporation patterns at 5'- and 3'- read termini for eight ancient samples. Nucleotide mis-incorporation patterns along the first and last 25 read positions obtained for the eight Shimao cattle before trimming and rescaling. All libraries were blunt-ended libraries (New England Biolabs) amplified with AmpliTaq Gold DNA polymerase (Life Technologies). Mis-incorporation frequencies are shown for the first and last 25 nucleotides of the reads aligned to the bovine reference nuclear genome Btau_5.0.1. The x-axis provides read positions relative to the read starts (positive numbers) and read ends (negative numbers). Red: C to T substitutions; Blue: G to A substitutions; and Grey: All other substitutions.

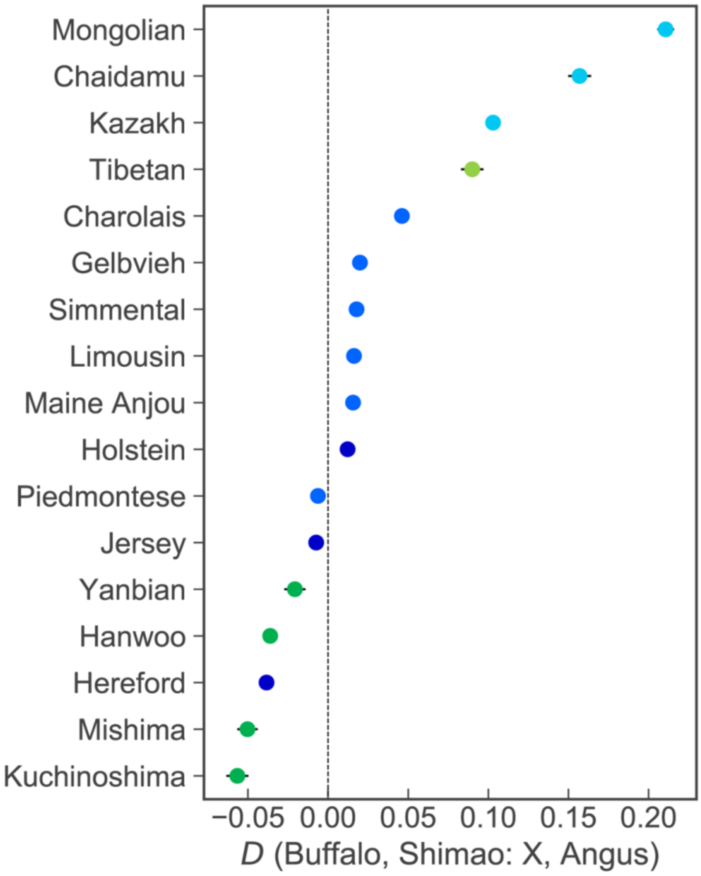

Supplementary Figure 11. Allele frequency-based *D* statistical test of the forms D (Buffalo, Shimao; X, Angus), where X represents modern-day *Bos taurus* populations from Europe and East Asia. The *D* statistic test is negative for Japanese cattle (Mishima and Kuchinoshima), followed by Hereford, Hanwoo, and Yanbian cattle, which provides evidence of the gene flow between ancient Chinese cattle and the Northeast Asian taurine group. Tibetan cattle were perhaps subjected to a relative stronger drift after the separation which may result in the positive *D* scores.

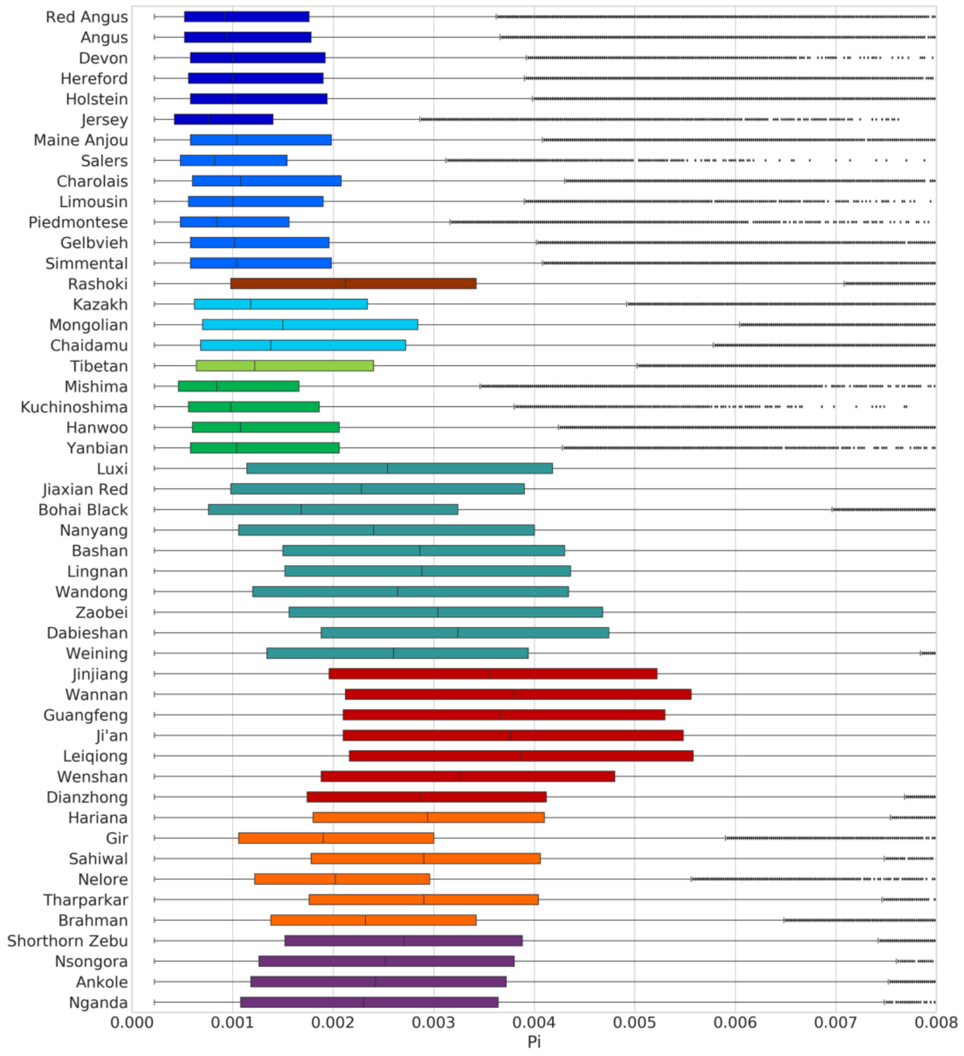

Supplementary Figure 12. Genome-wide distribution of nucleotide diversity by using VCFtools. The vertical line inside the box corresponds to the median of this distribution, the left and right of the box are the first and third quartiles. Data points outside the whiskers can be considered as outliers.

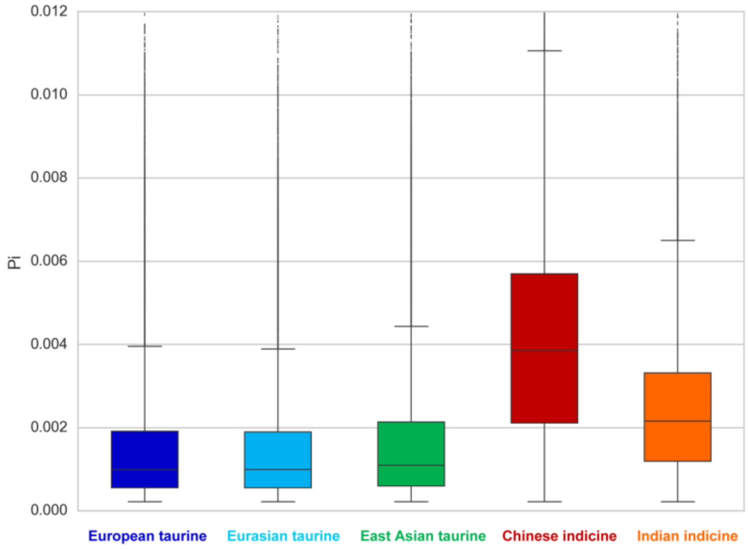

Supplementary Figure 13. Genome-wide distribution of nucleotide diversity of five core group. Nucleotide diversity of the five groups were repeated by using ANGSD. The horizontal line inside the box corresponds to the median of this distribution, the bottom and top of the box are the first and third quartiles and data points outside the whiskers can be considered as outliers.

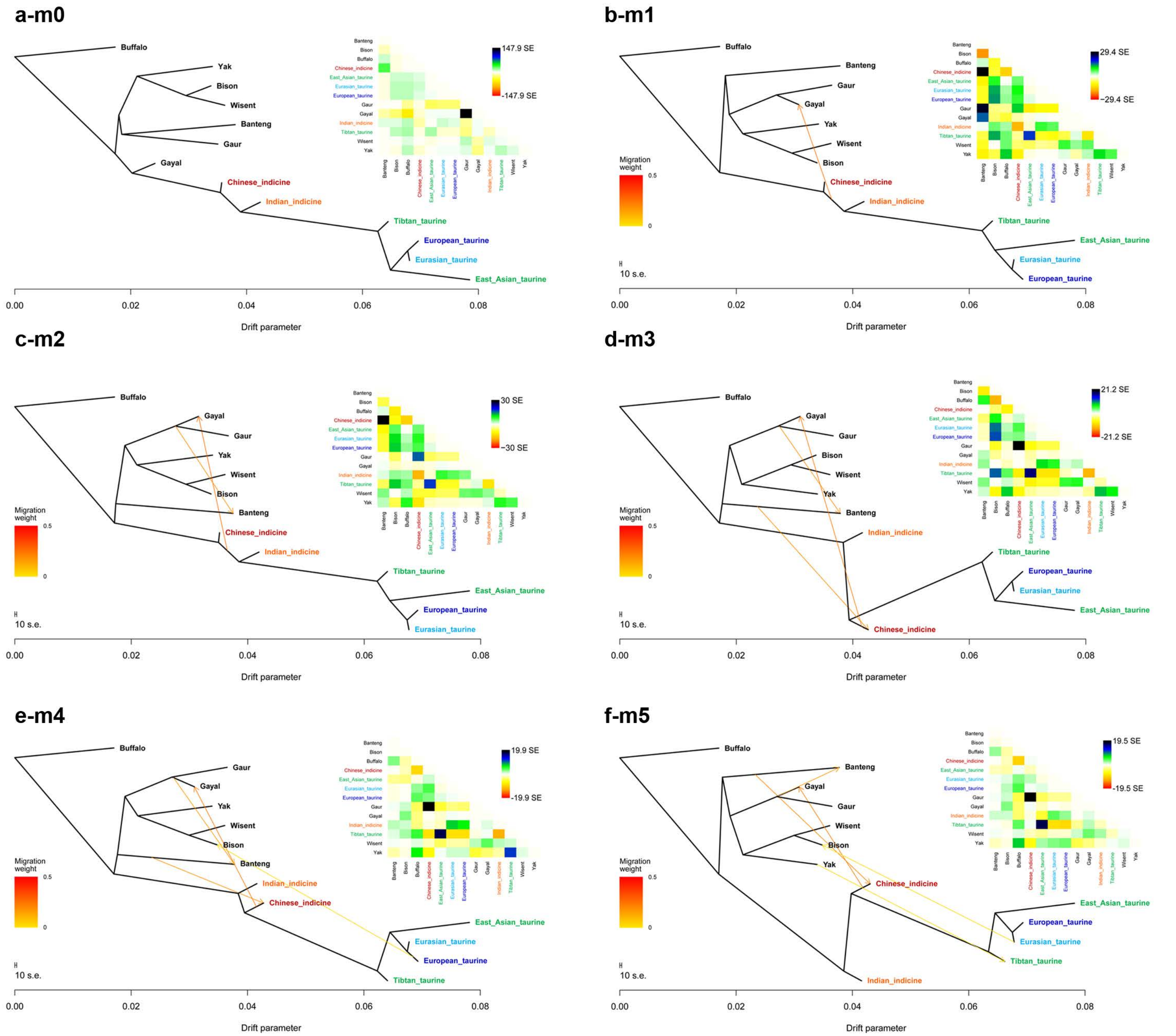

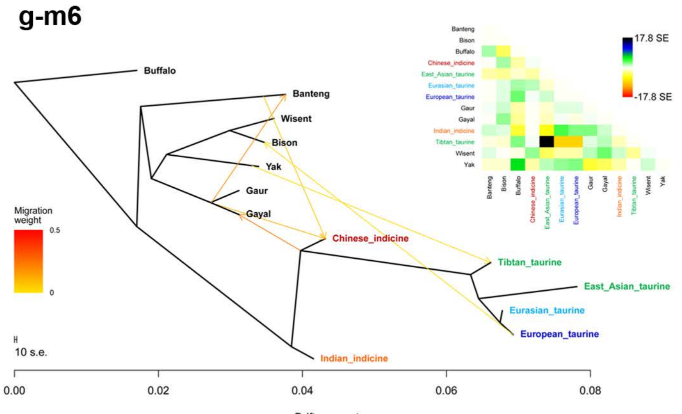

Supplementary Figure 14. TreeMix population splits between five "core" cattle groups and other species in the tribe of Bovini. Left: Population relationships as inferred from the Treemix analyses, with 0-6 possible migration edges (m0-m6). Right: Residuals of the covariance matrices. Proportions of the variance explained by the tree were: a. 99.46%; b. 99.90%; c. 99.93%; d. 99.95%; e. 99.97%; f. 99.97%; and g. 99.98%.

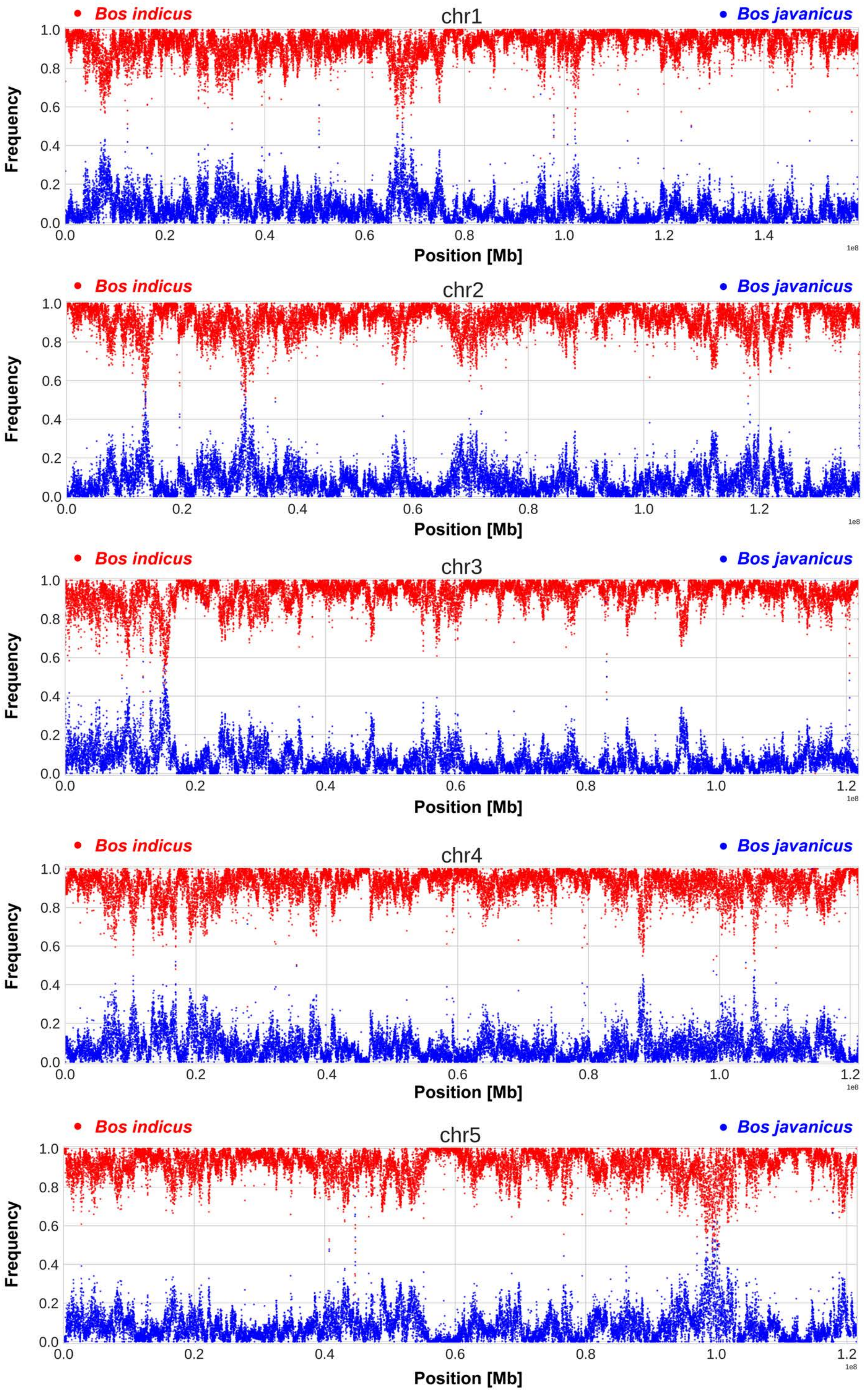

Bos indicus
chr1
Bos javanicus
Frequency
Position [Mb]
1e8
Bos indicus
chr2
Bos javanicus
Frequency
Position [Mb]
1e8
Bos indicus
chr3
Bos javanicus
Frequency
Position [Mb]
1e8
Bos indicus
chr4
Bos javanicus
Frequency
Position [Mb]
1e8
Bos indicus
chr5
Bos javanicus
Frequency
Position [Mb]
1e8

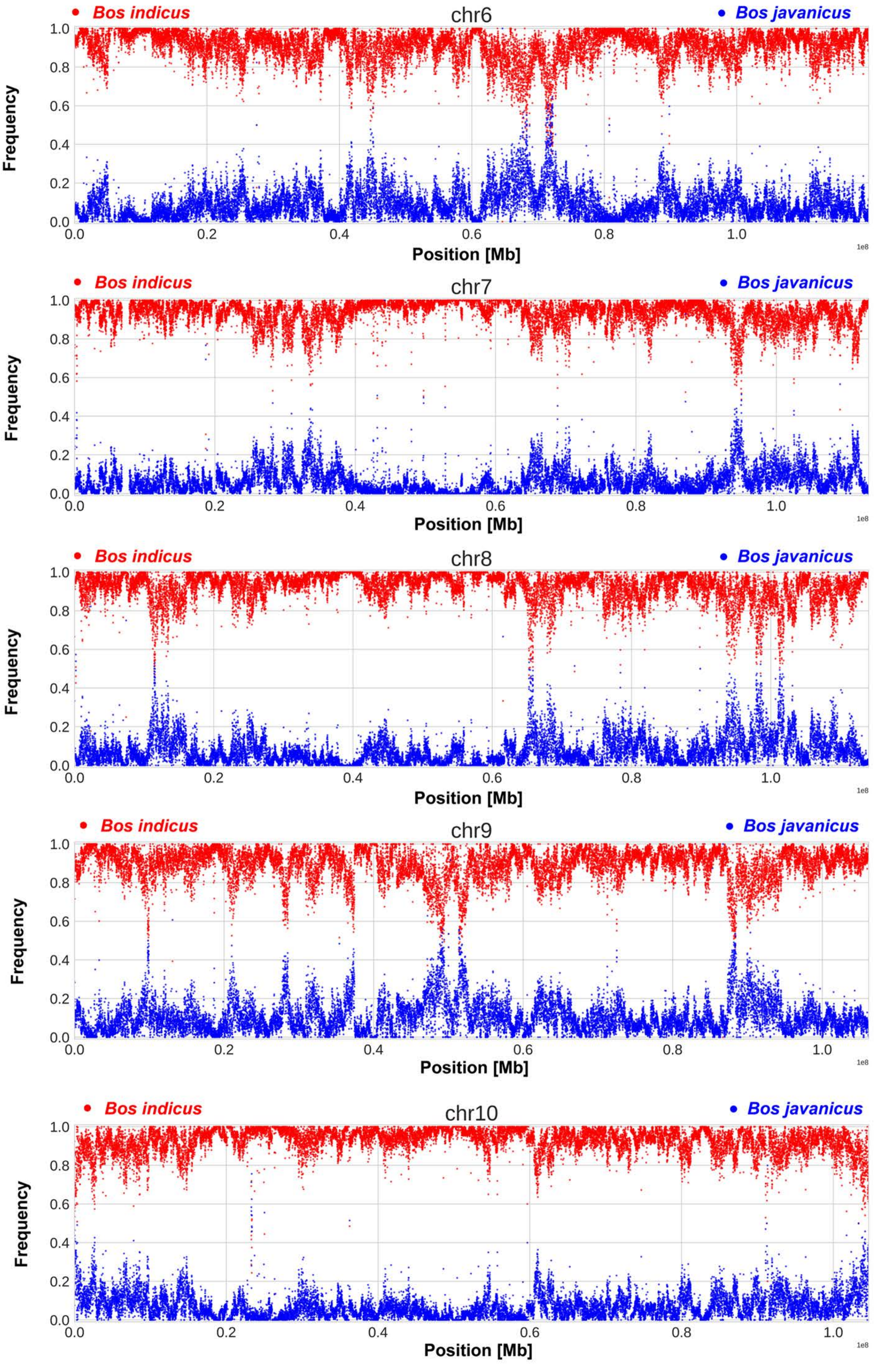
• Bos indicus
chr6
• Bos javanicus
Frequency
Position [Mb]
1e8
• Bos indicus
chr7
• Bos javanicus
Frequency
Position [Mb]
1e8
• Bos indicus
chr8
• Bos javanicus
Frequency
Position [Mb]
1e8
• Bos indicus
chr9
• Bos javanicus
Frequency
Position [Mb]
1e8
• Bos indicus
chr10
• Bos javanicus
Frequency
Position [Mb]
1e8

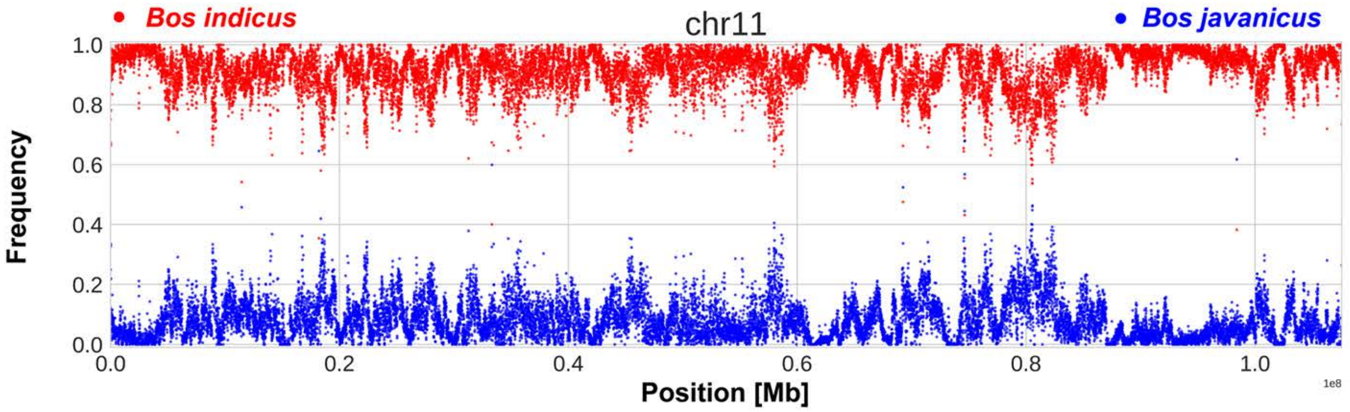

Bos indicus
chr11
Bos javanicus
Frequency
Position [Mb]
1e8

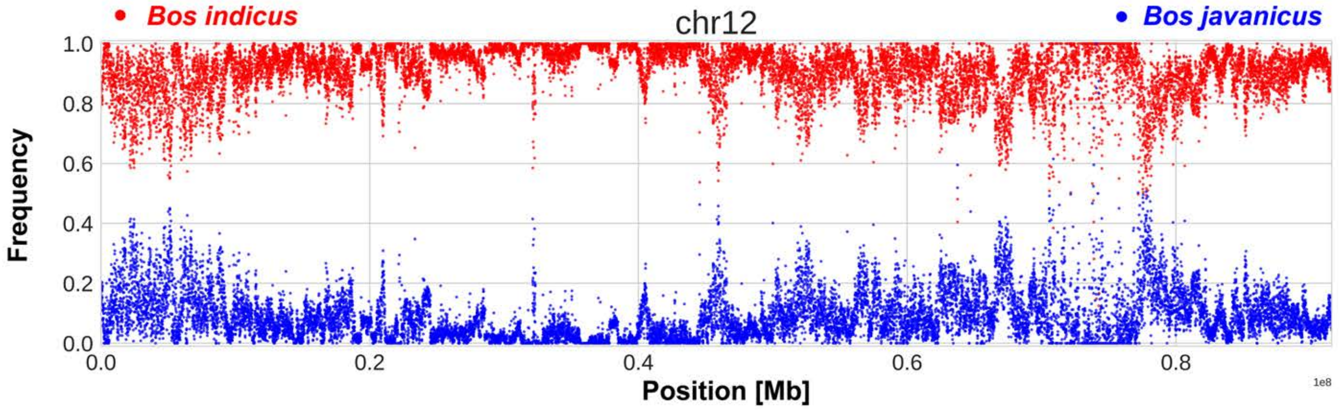

Bos indicus
chr12
Bos javanicus
Frequency
Position [Mb]
1e8

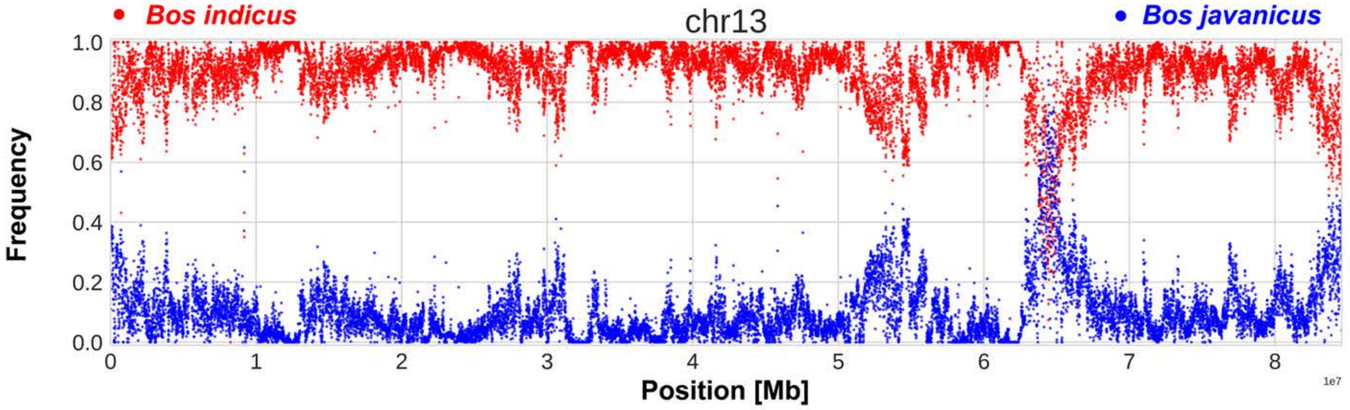

Bos indicus
chr13
Bos javanicus
Frequency
Position [Mb]
1e7

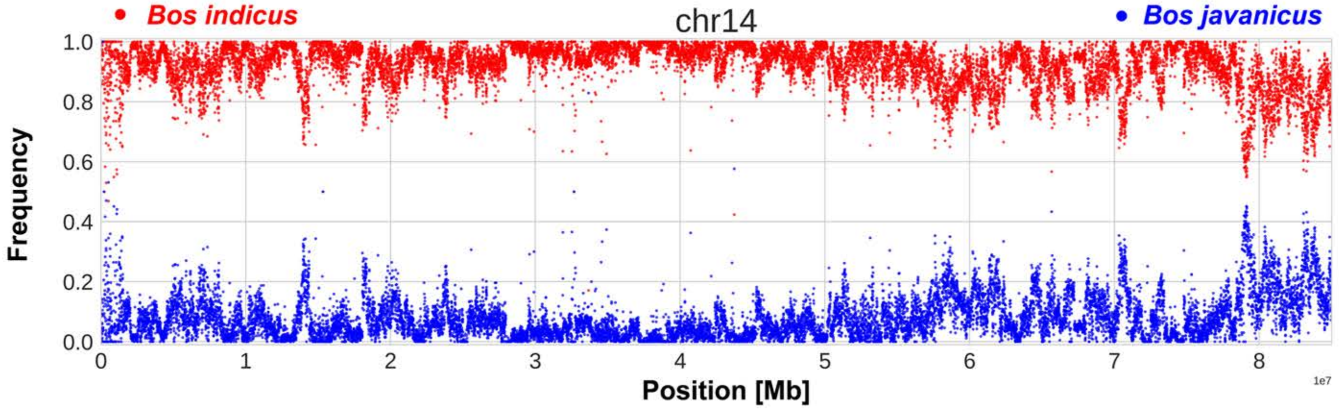

Bos indicus
chr14
Bos javanicus
Frequency
Position [Mb]
1e7

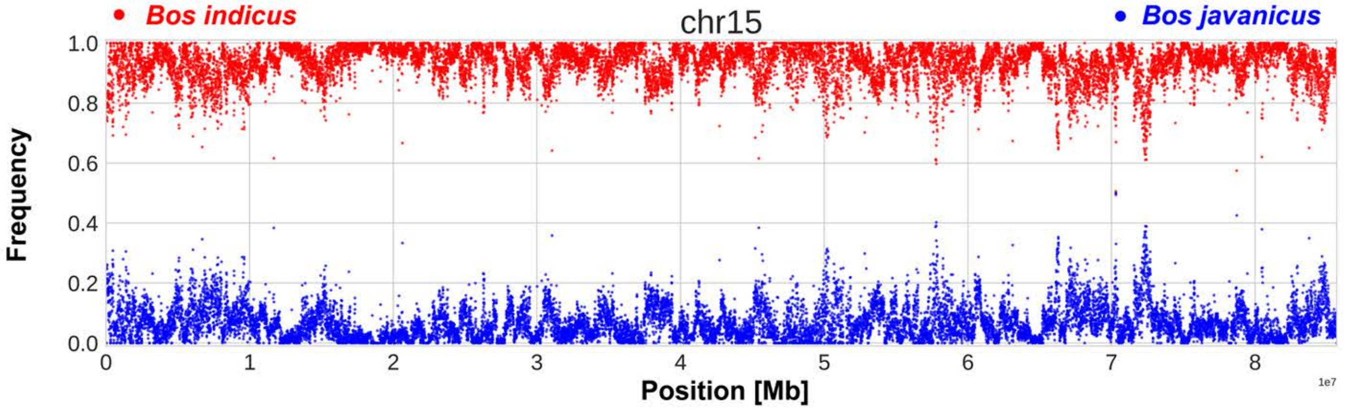

Bos indicus
chr15
Bos javanicus
Frequency
Position [Mb]
1e7

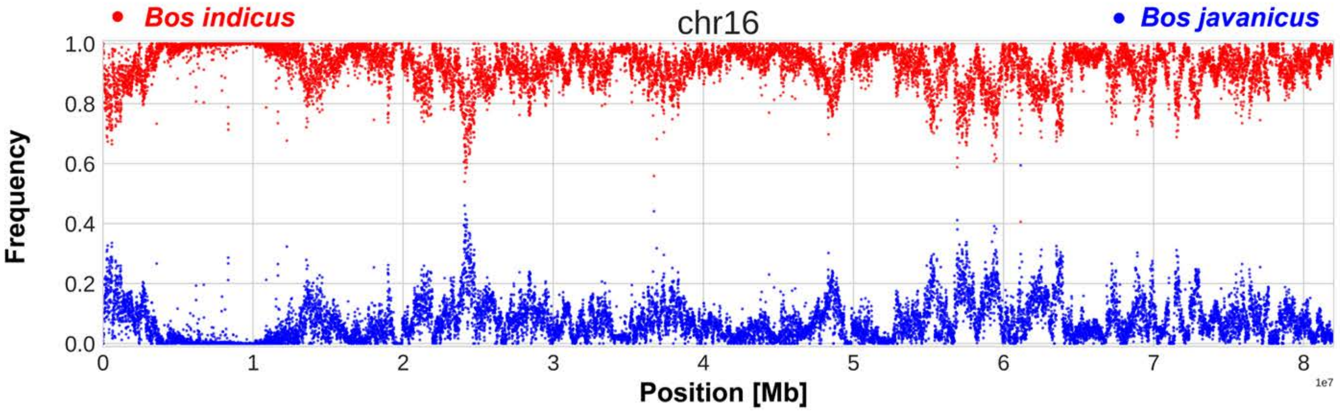

Bos indicus
chr16
Bos javanicus
Frequency
1.0
0.8
0.6
0.4
0.2
0.0
0
1
2
3
4
5
6
7
8
Position [Mb]
1e7

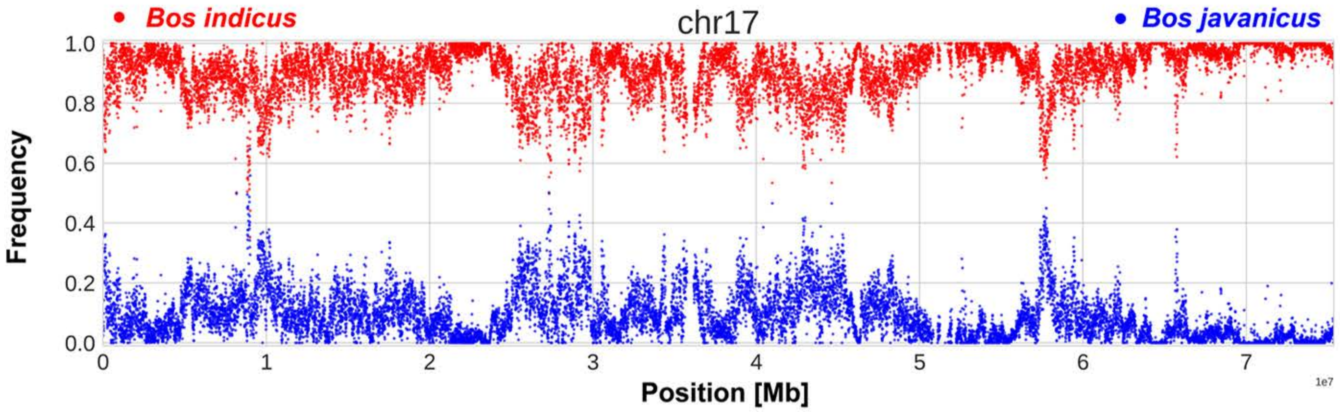

Bos indicus
chr17
Bos javanicus
Frequency
1.0
0.8
0.6
0.4
0.2
0.0
0
1
2
3
4
5
6
7
Position [Mb]
1e7

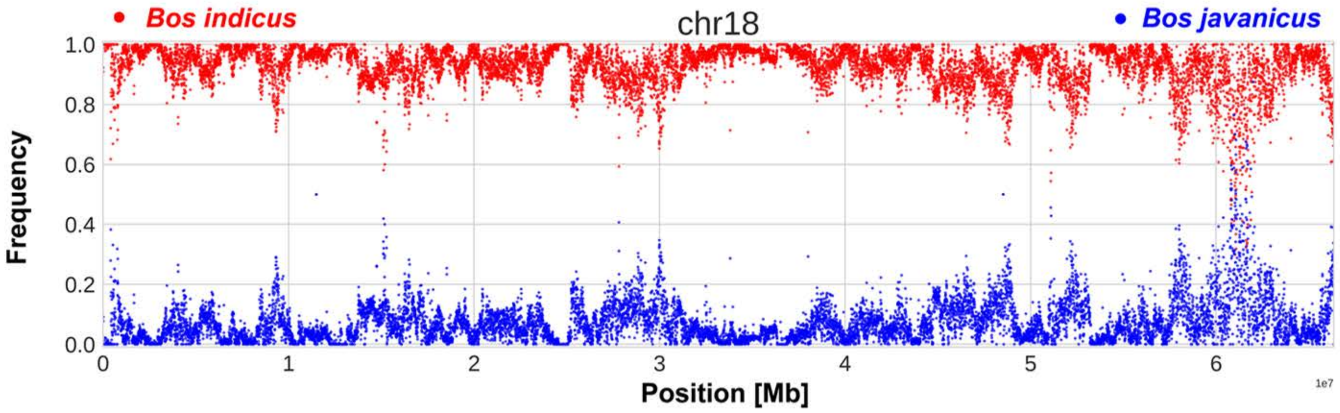

Bos indicus
chr18
Bos javanicus
Frequency
1.0
0.8
0.6
0.4
0.2
0.0
0
1
2
3
4
5
6
Position [Mb]
1e7

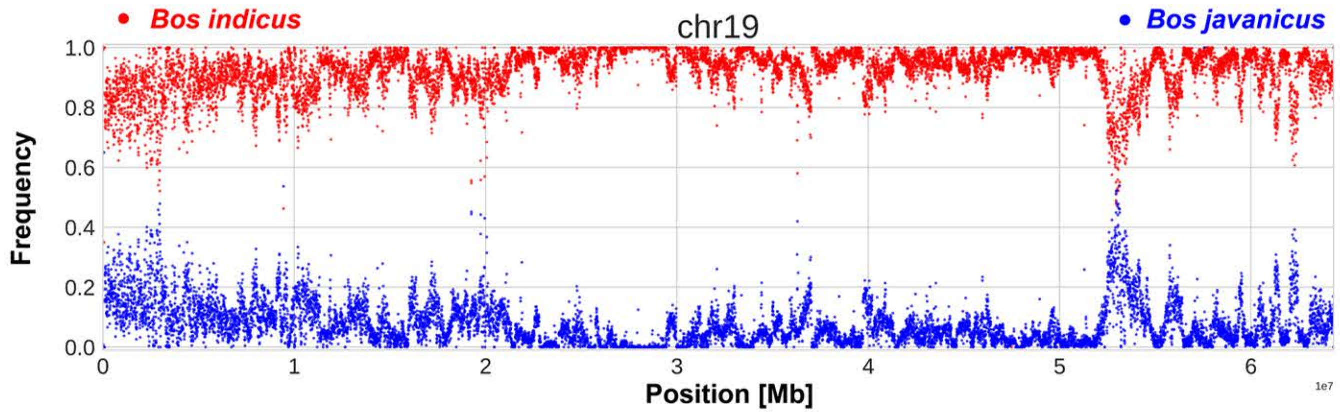

Bos indicus
chr19
Bos javanicus
Frequency
1.0
0.8
0.6
0.4
0.2
0.0
0
1
2
3
4
5
6
Position [Mb]
1e7

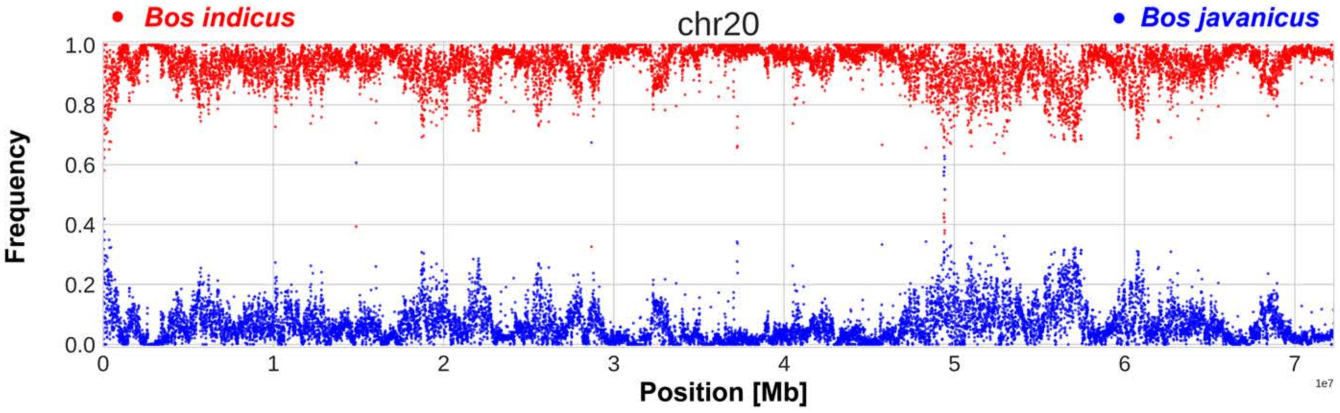

Bos indicus
chr20
Bos javanicus
Frequency
1.0
0.8
0.6
0.4
0.2
0.0
0
1
2
3
4
5
6
7
Position [Mb]
1e7

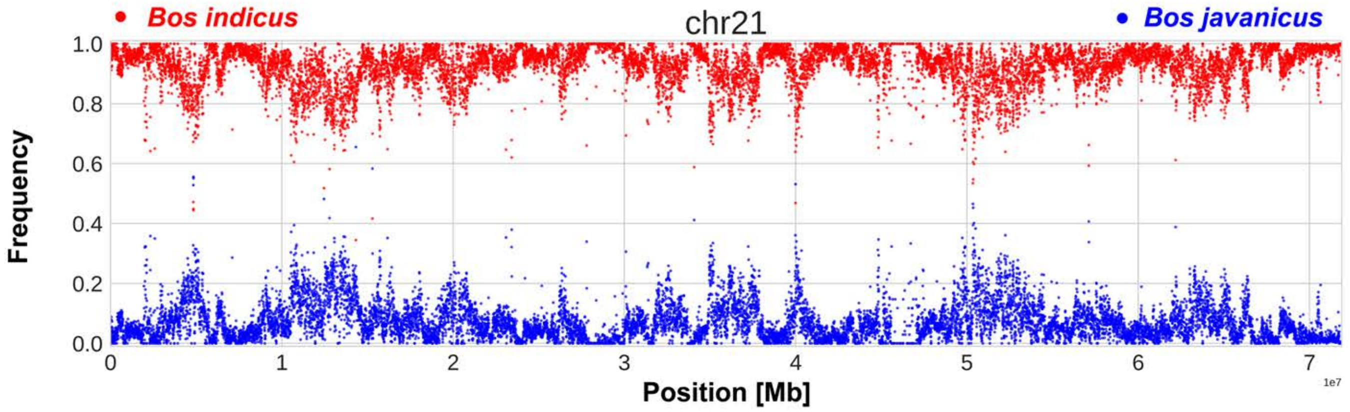

Bos indicus
chr21
Bos javanicus
Frequency
Position [Mb]
1e7

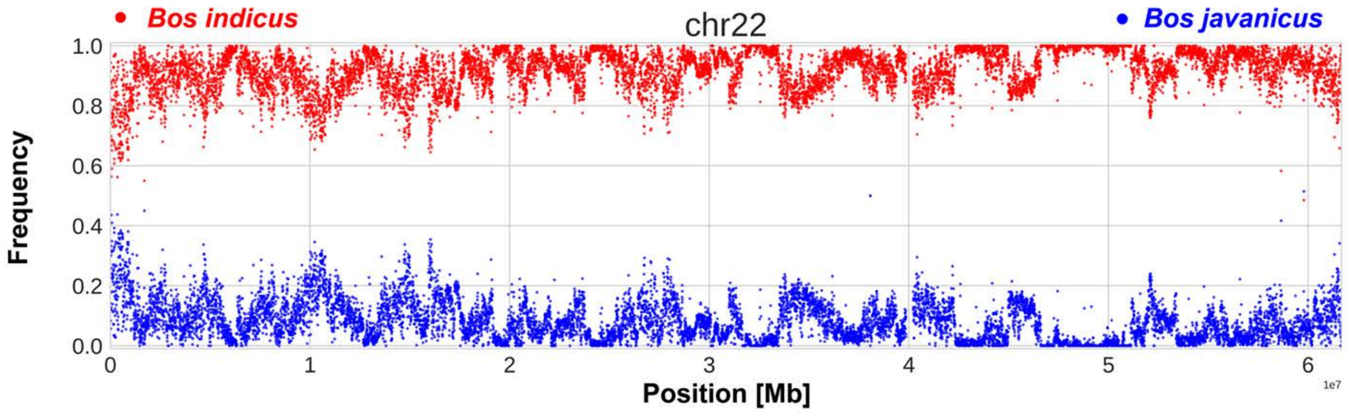

Bos indicus
chr22
Bos javanicus
Frequency
Position [Mb]
1e7

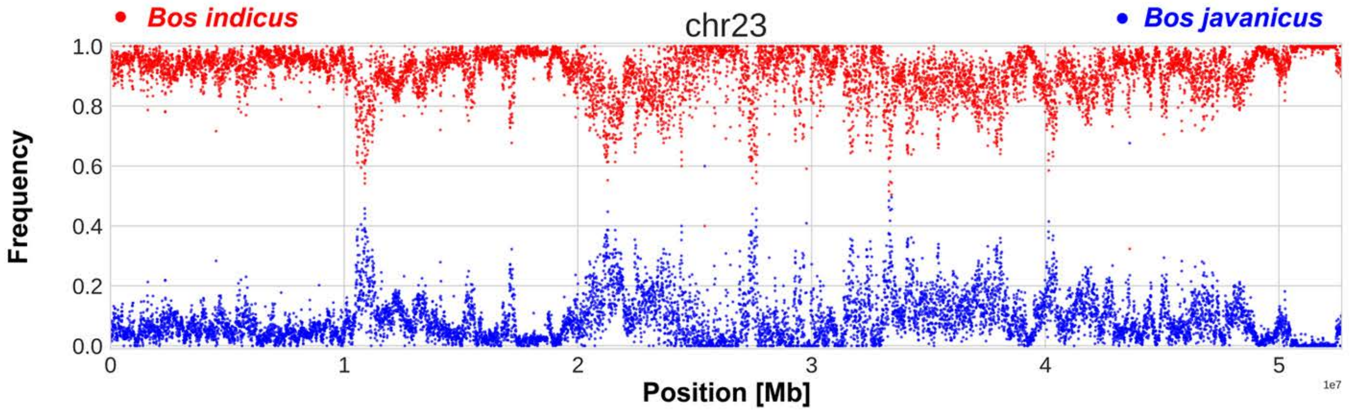

Bos indicus
chr23
Bos javanicus
Frequency
Position [Mb]
1e7

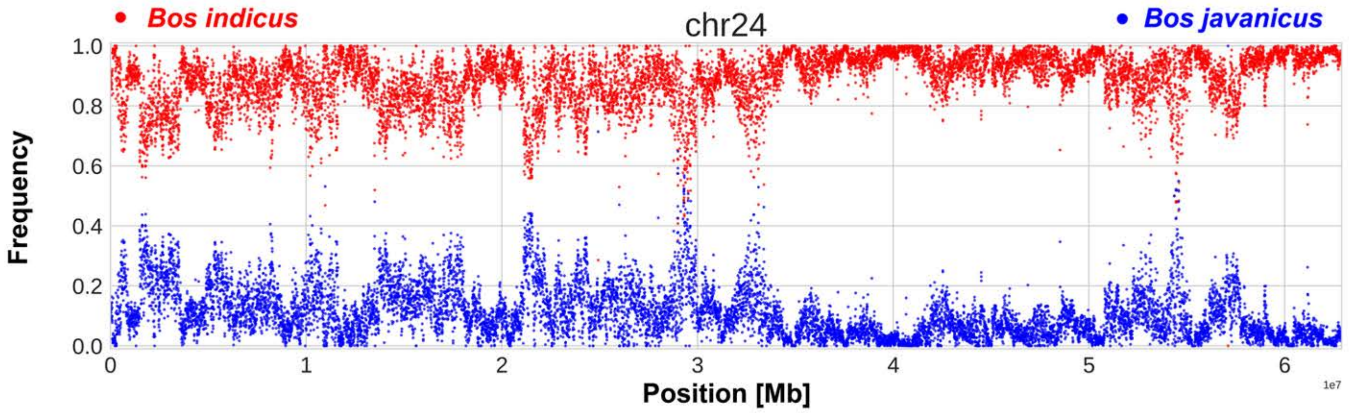

Bos indicus
chr24
Bos javanicus
Frequency
Position [Mb]
1e7

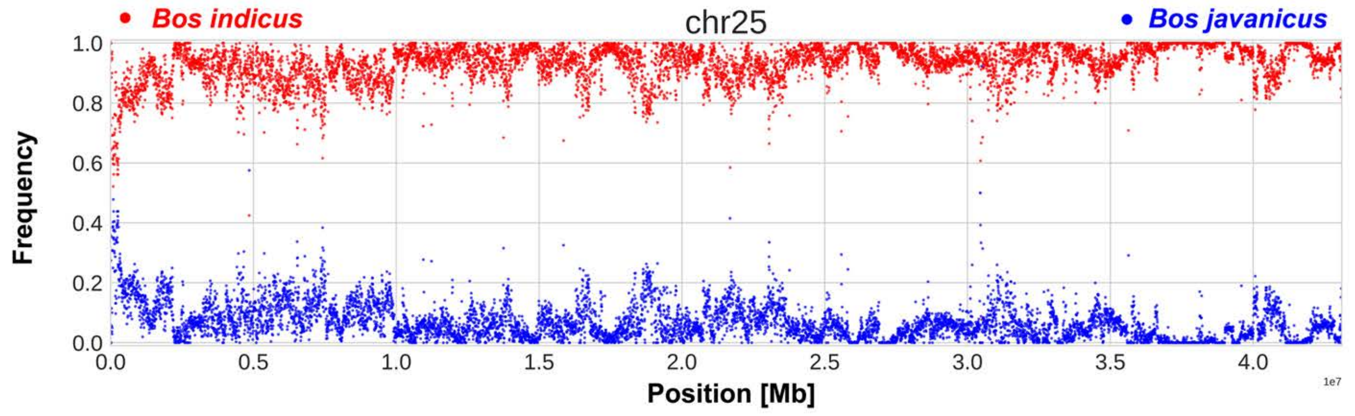

Bos indicus
chr25
Bos javanicus
Frequency
Position [Mb]
1e7

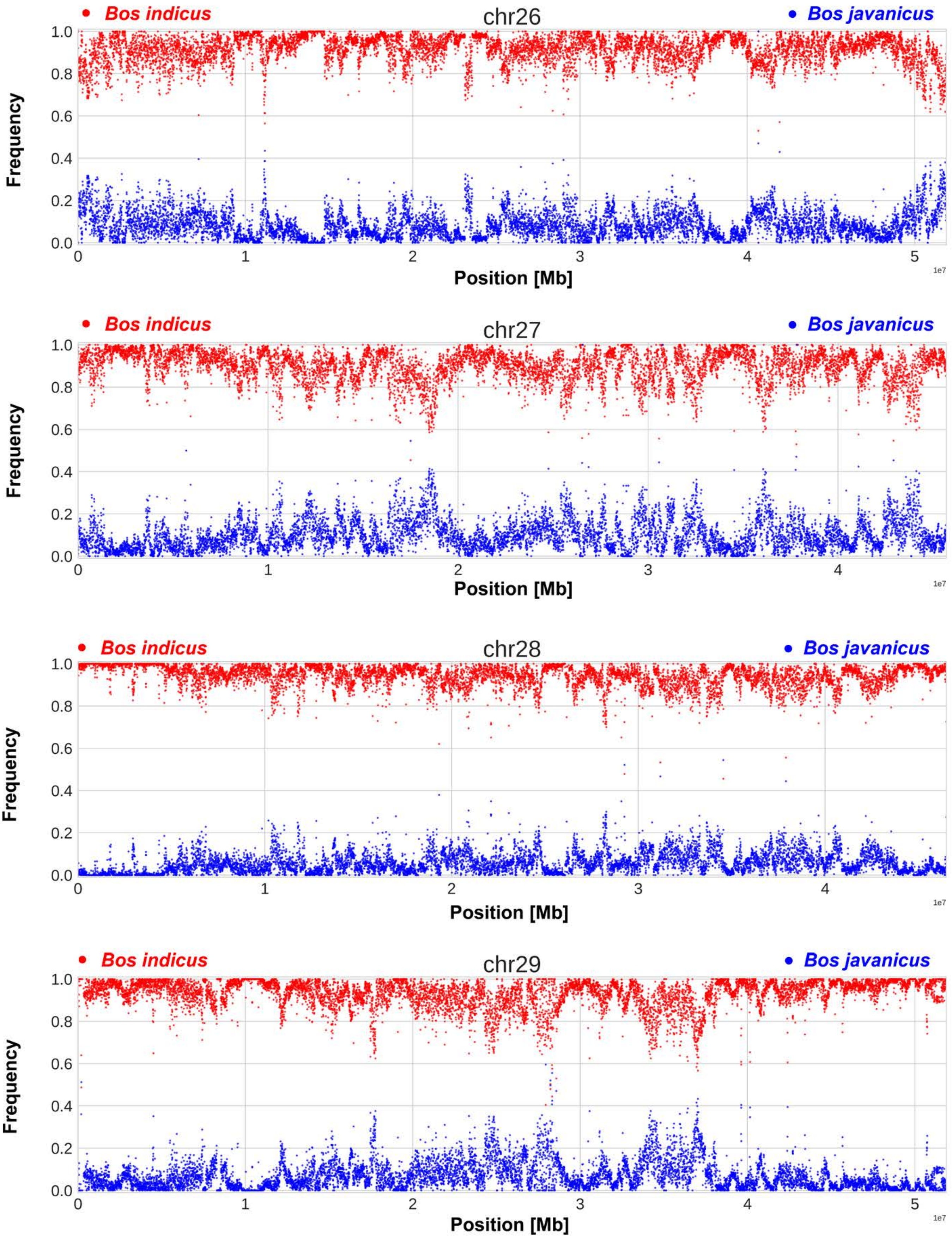

Supplementary Figure 15. Frequency of bateng and indicine alleles for all positions of banteng-specific SNPs in 5-kb sliding windows. Red and blue dots show the relative frequencies of indicine cattle and banteng-specific alleles, respectively.

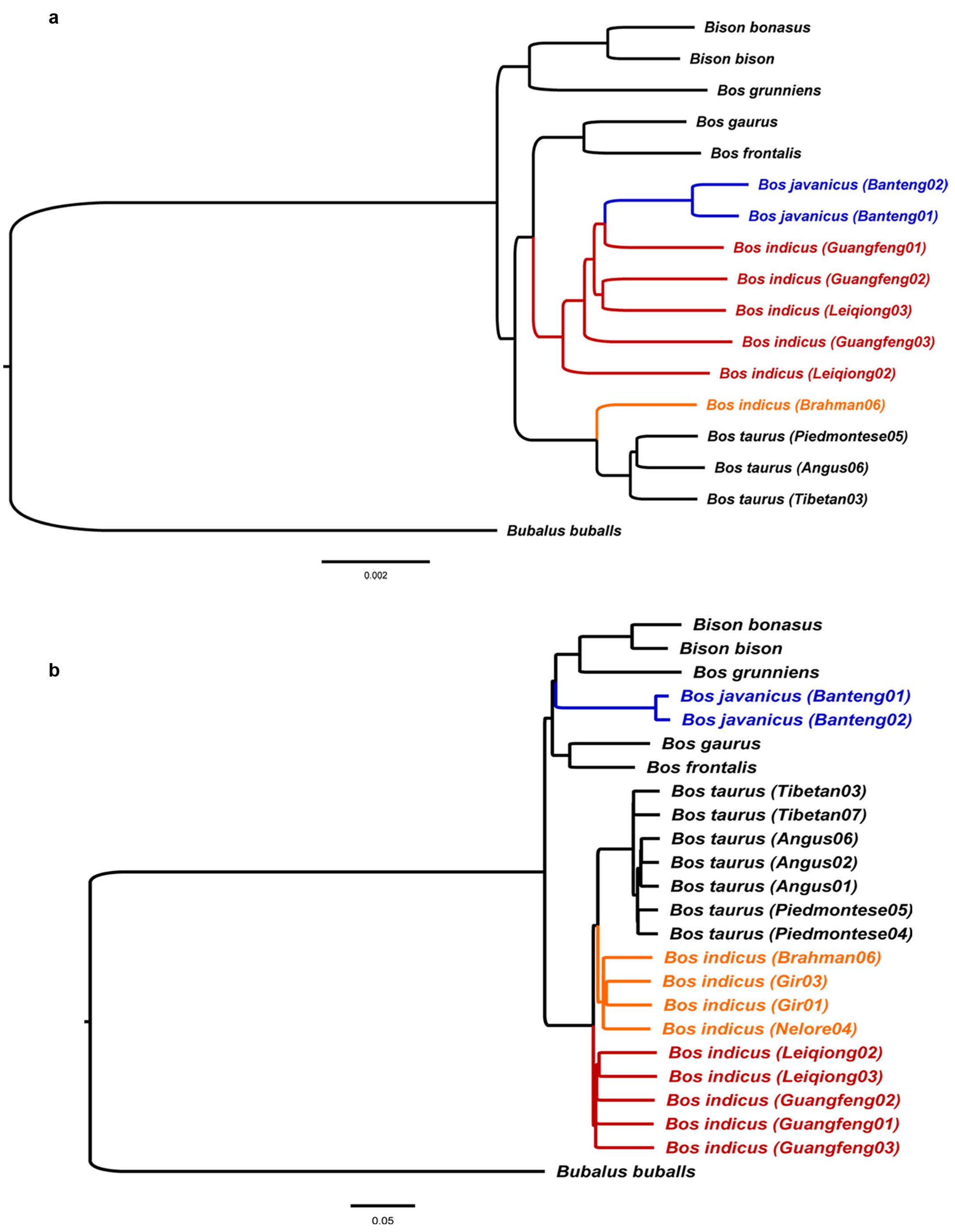

Supplementary Figure 16. Rooted neighbour-joining (NJ) tree for segments introgressed in 15 or more Chinese indicine haploids. (a) NJ tree reconstructed using PHYLIP based on the SNPs in 13 introgression intervals (Supplementary Table 23) in 15 or more Chinese indicine haploids. (b) NJ tree reconstructed using PHYLIP based on whole-genome SNPs without all introgression intervals. The path to pure *Bos inidicus* (Nelore) is emphasized by an orange line and letters whereas paths to introgressed *Bos indicus* are emphasized by red lines and letters. Two *Bos javanicus* cattle are emphasized by blue lines and letters.

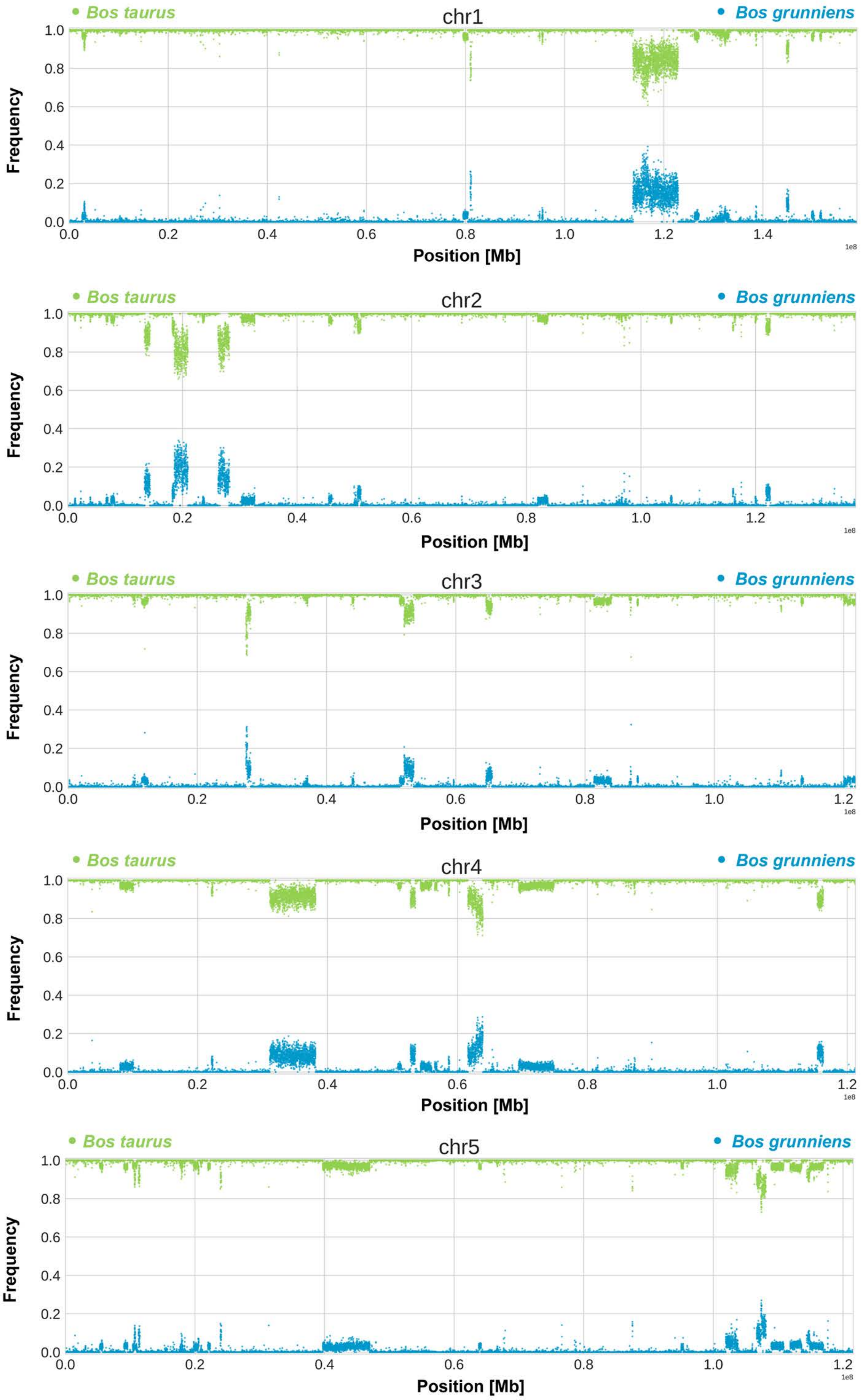

Bos taurus
chr1
Bos grunniens
Frequency
Position [Mb]
1e8
Bos taurus
chr2
Bos grunniens
Frequency
Position [Mb]
1e8
Bos taurus
chr3
Bos grunniens
Frequency
Position [Mb]
1e8
Bos taurus
chr4
Bos grunniens
Frequency
Position [Mb]
1e8
Bos taurus
chr5
Bos grunniens
Frequency
Position [Mb]
1e8

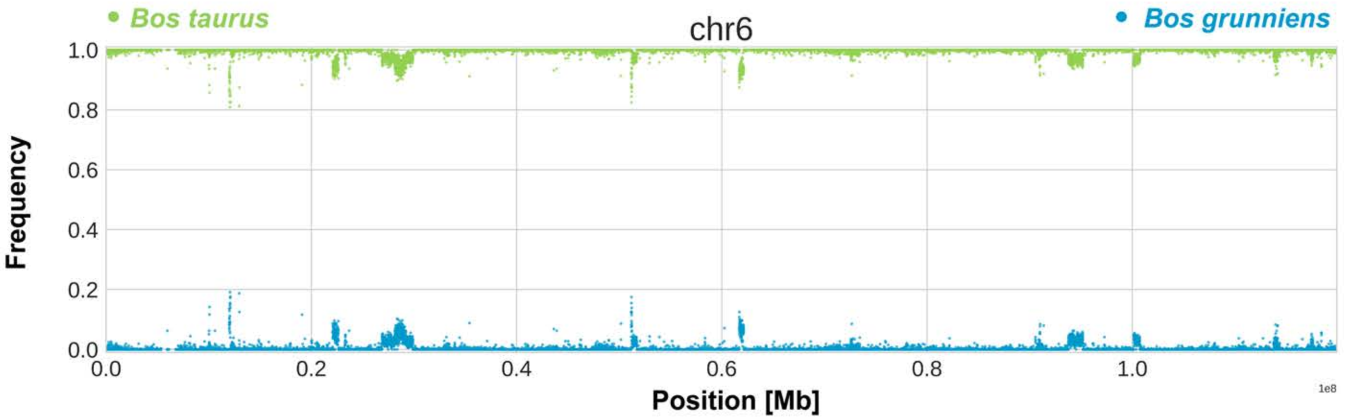
Bos taurus
chr6
Bos grunniens
Frequency
1.0
0.8
0.6
0.4
0.2
0.0
0.0
0.2
0.4
0.6
0.8
1.0
1e8
Position [Mb]

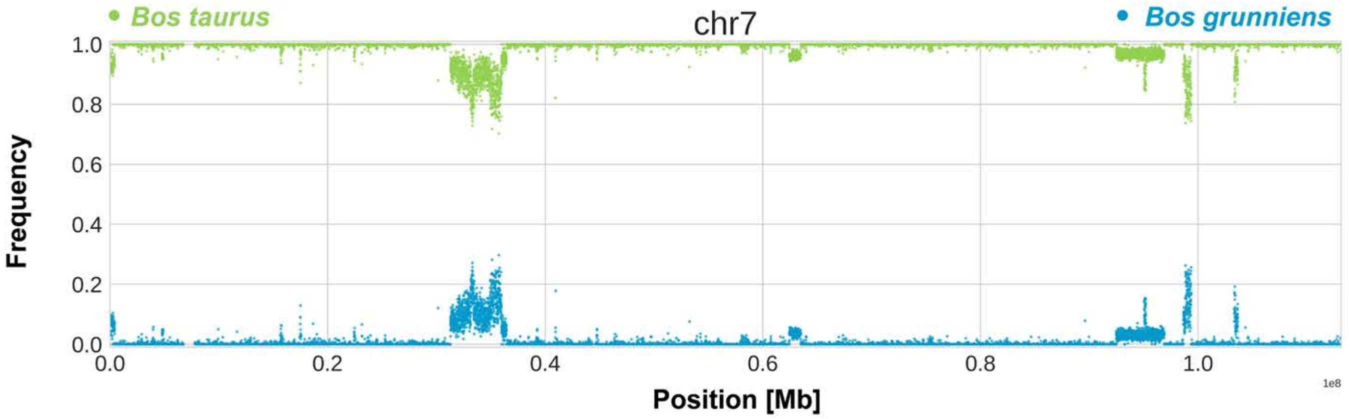
Bos taurus
chr7
Bos grunniens
Frequency
1.0
0.8
0.6
0.4
0.2
0.0
0.0
0.2
0.4
0.6
0.8
1.0
1e8
Position [Mb]

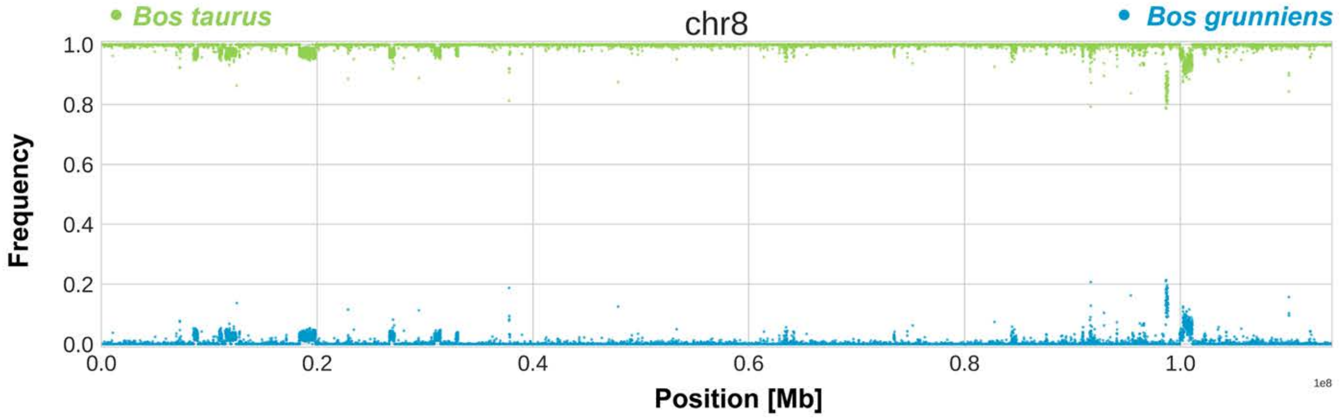
Bos taurus
chr8
Bos grunniens
Frequency
1.0
0.8
0.6
0.4
0.2
0.0
0.0
0.2
0.4
0.6
0.8
1.0
1e8
Position [Mb]

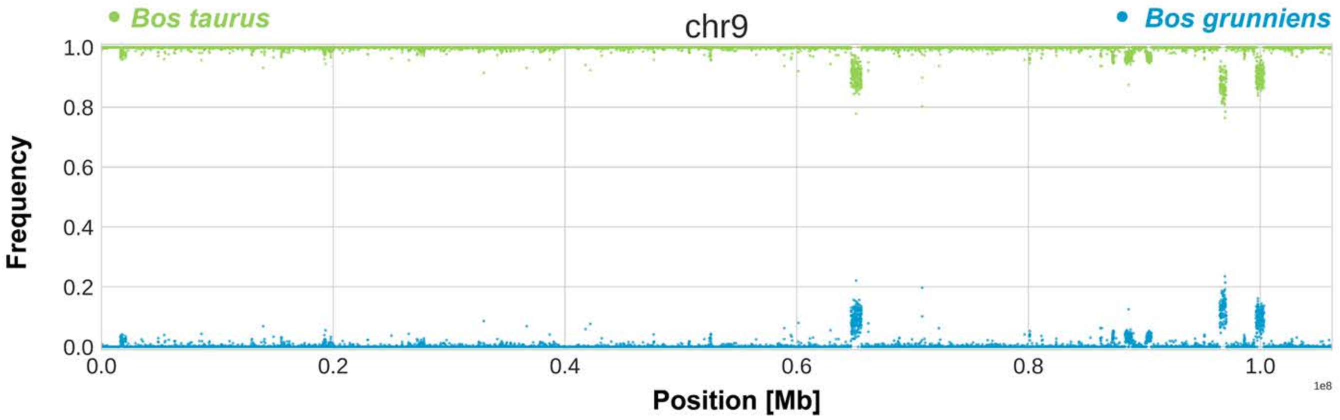
Bos taurus
chr9
Bos grunniens
Frequency
1.0
0.8
0.6
0.4
0.2
0.0
0.0
0.2
0.4
0.6
0.8
1.0
1e8
Position [Mb]

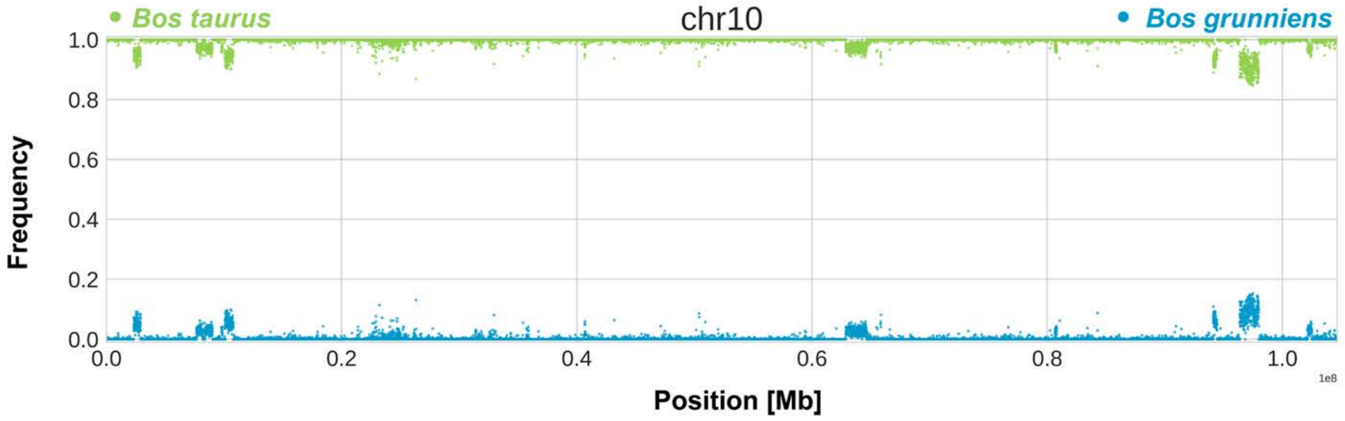
Bos taurus
chr10
Bos grunniens
Frequency
1.0
0.8
0.6
0.4
0.2
0.0
0.0
0.2
0.4
0.6
0.8
1.0
1e8
Position [Mb]

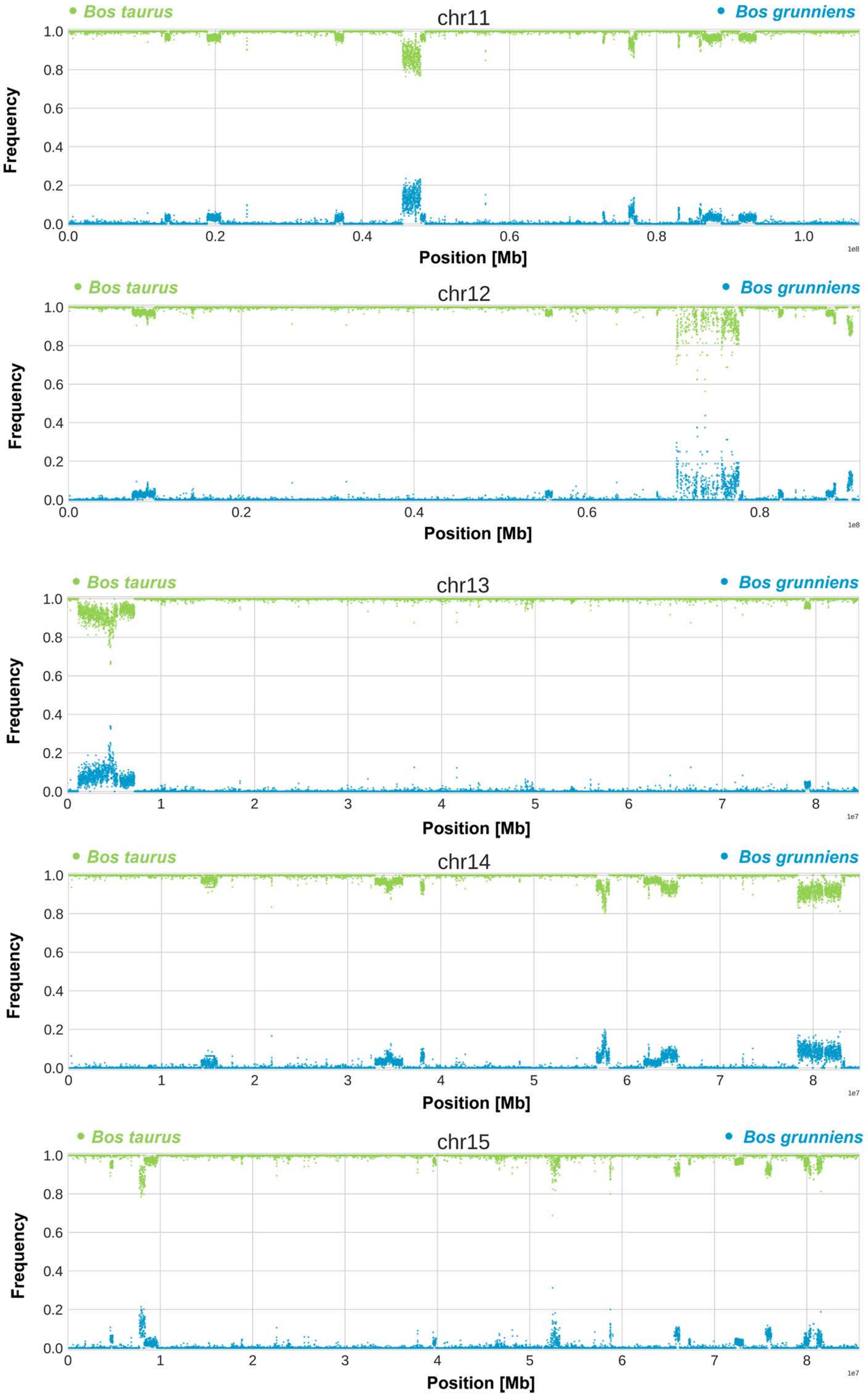
Bos taurus
chr11
Bos grunniens
Frequency
Position [Mb]
1e8
Bos taurus
chr12
Bos grunniens
Frequency
Position [Mb]
1e8
Bos taurus
chr13
Bos grunniens
Frequency
Position [Mb]
1e7
Bos taurus
chr14
Bos grunniens
Frequency
Position [Mb]
1e7
Bos taurus
chr15
Bos grunniens
Frequency
Position [Mb]
1e7

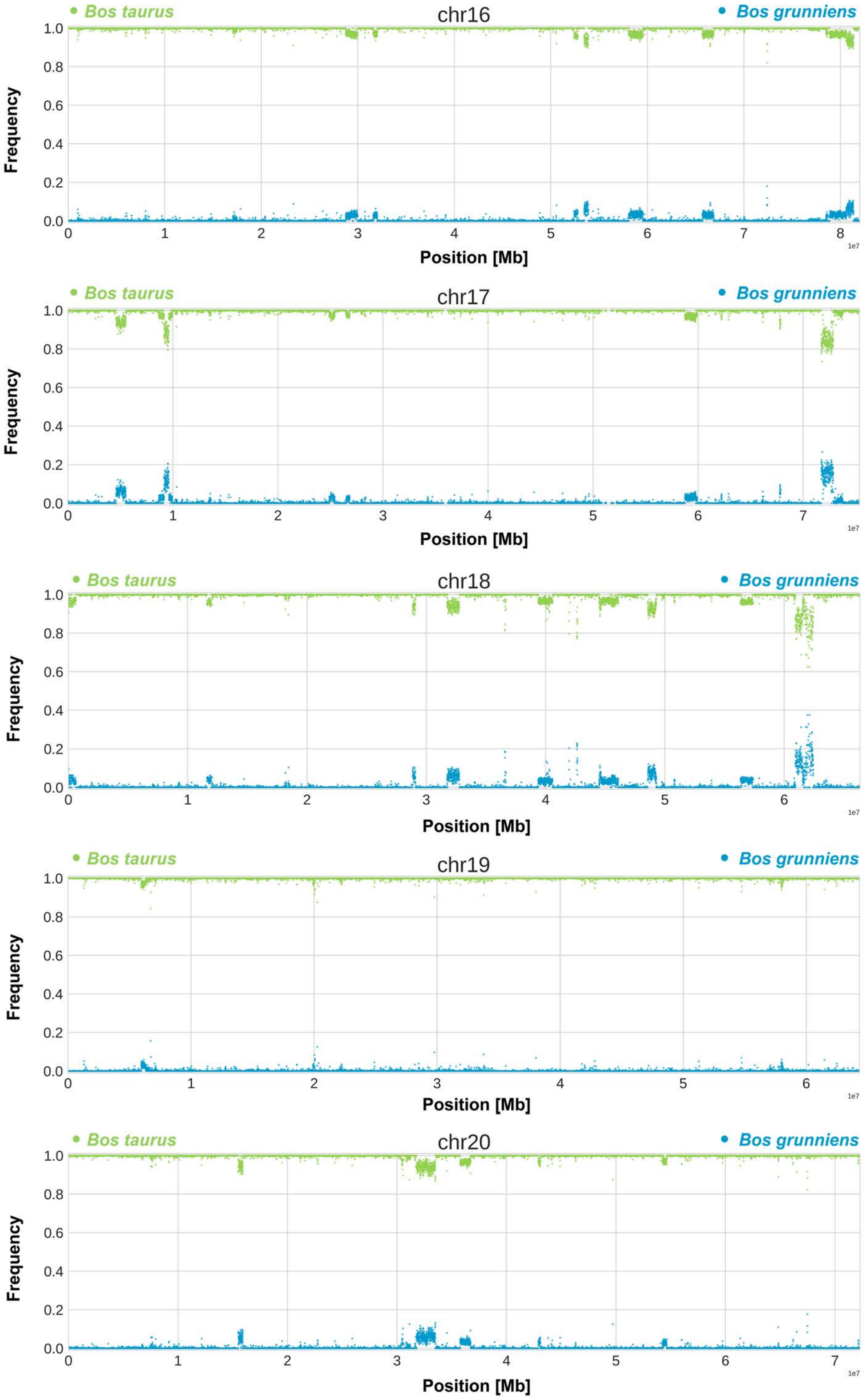
Bos taurus
chr16
Bos grunniens
Frequency
1.0
0.8
0.6
0.4
0.2
0.0
0
1
2
3
4
5
6
7
8
1e7
Position [Mb]
Bos taurus
chr17
Bos grunniens
Frequency
Position [Mb]
1e7
Bos taurus
chr18
Bos grunniens
Frequency
Position [Mb]
1e7
Bos taurus
chr19
Bos grunniens
Frequency
Position [Mb]
1e7
Bos taurus
chr20
Bos grunniens
Frequency
Position [Mb]
1e7

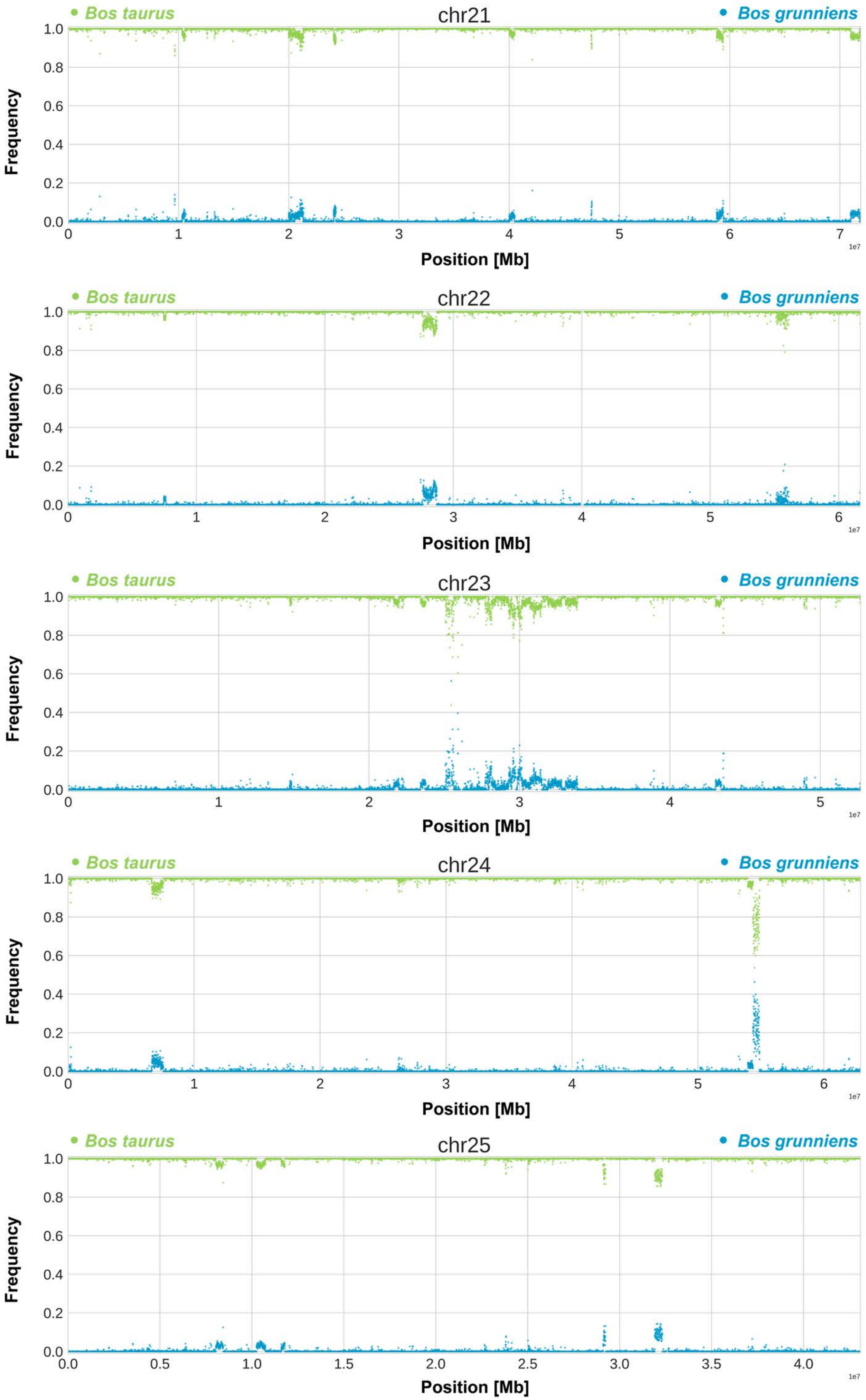

Bos taurus
chr21
Bos grunniens
Frequency
Position [Mb]
1e7
Bos taurus
chr22
Bos grunniens
Frequency
Position [Mb]
1e7
Bos taurus
chr23
Bos grunniens
Frequency
Position [Mb]
1e7
Bos taurus
chr24
Bos grunniens
Frequency
Position [Mb]
1e7
Bos taurus
chr25
Bos grunniens
Frequency
Position [Mb]
1e7

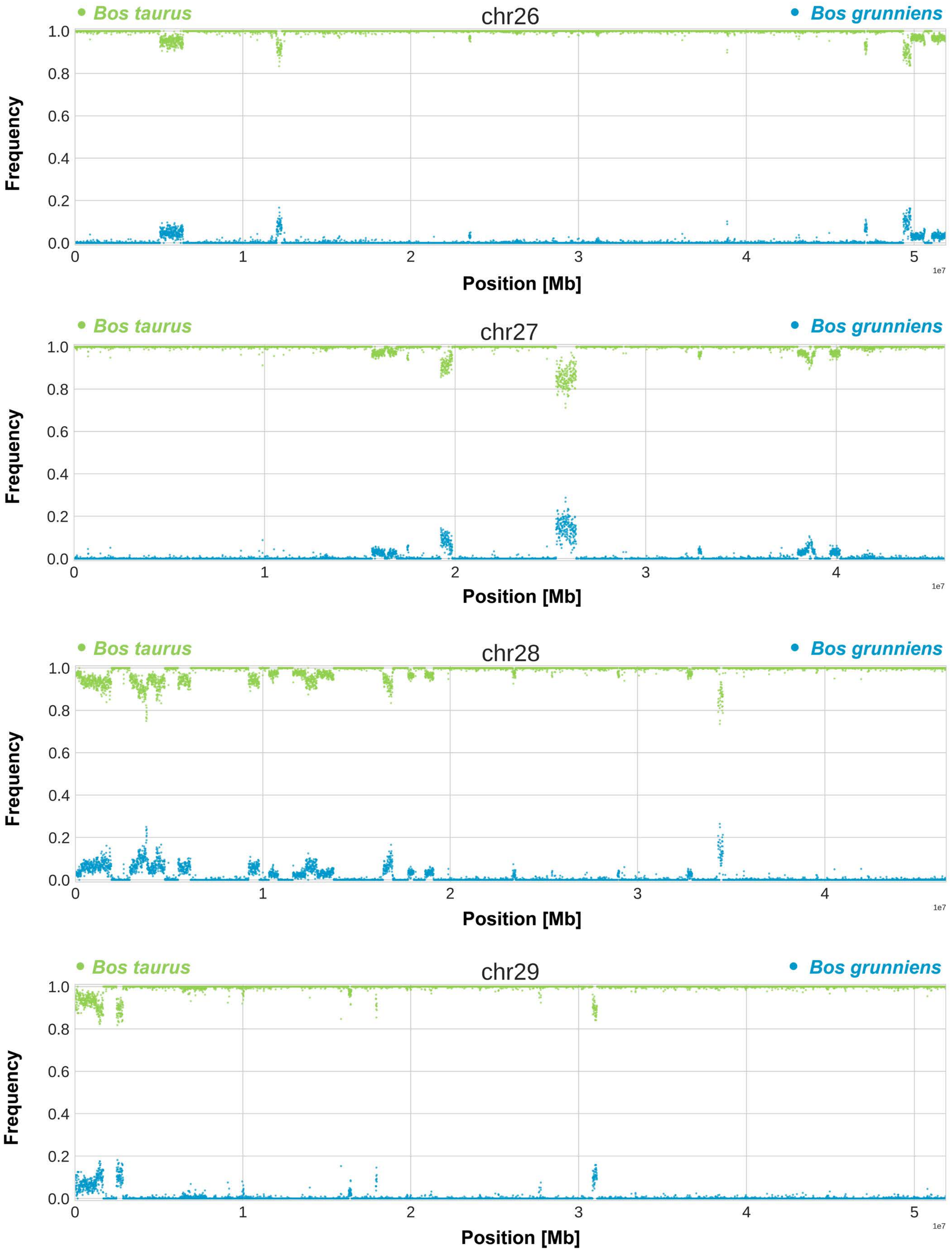

Supplementary Figure 17. Frequency of yak and bovine alleles for all positions of yak-specific SNPs in 5-kb sliding windows. Green and sky-blue dots show the relative frequencies of taurine- and yak-specific alleles, respectively.

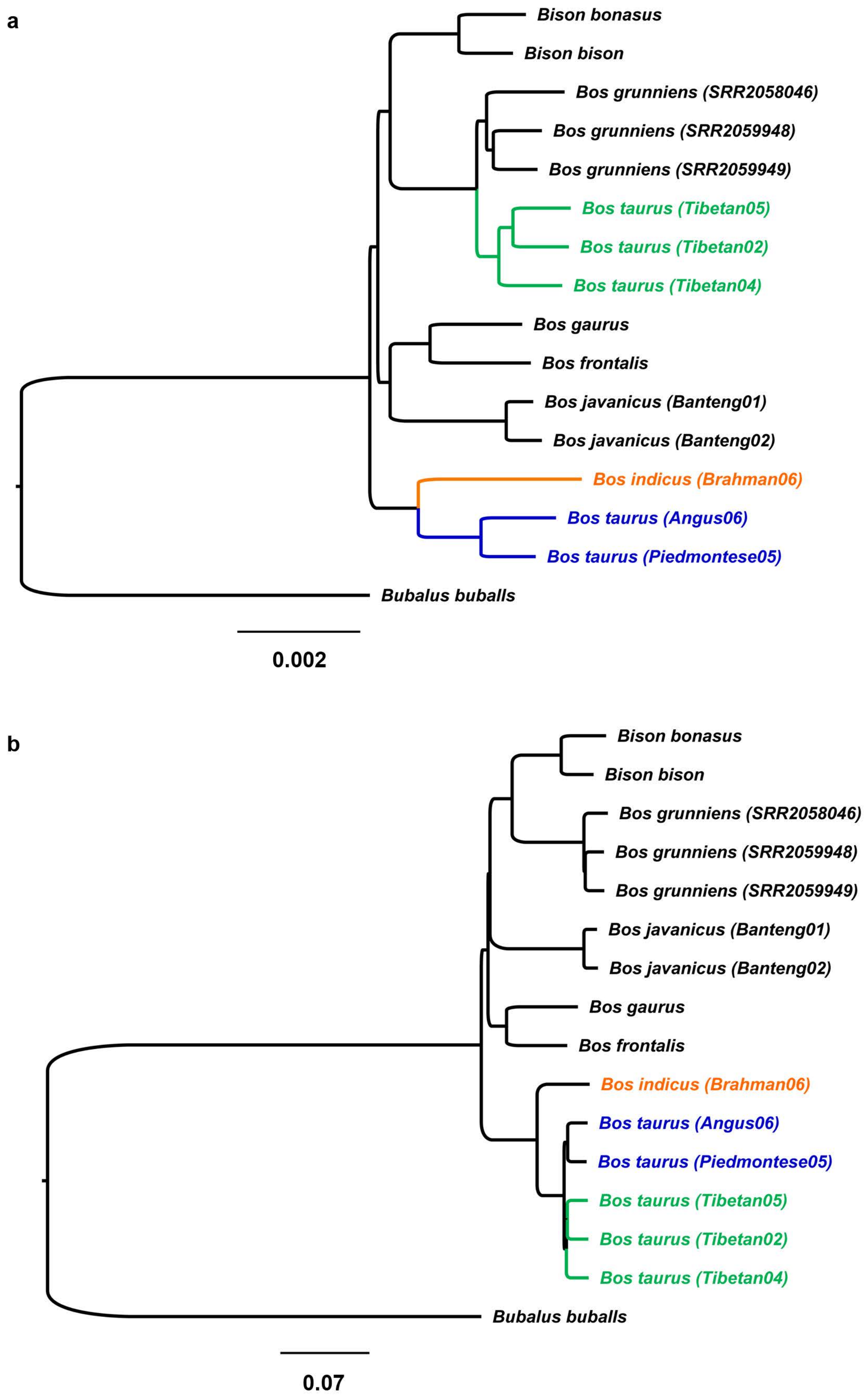

Supplementary Figure 18. Rooted neighbour-joining (NJ) tree for segments introgressed in ten or more yak haploids (a) Rooted NJ tree reconstructed using PHYLIP based on the SNPs in 30 introgression intervals in eight or more Tibetan taurine haploids. (b) NJ tree reconstructed using PHYLIP based on whole-genome SNPs without all introgression intervals. The path to pure *Bos taurus* are emphasized by blue lines and letters, whereas paths to introgressed Tibetan taurine cattle are emphasized by green lines and letters. The *Bos indicus* individual is emphasized by an orange line and letters.

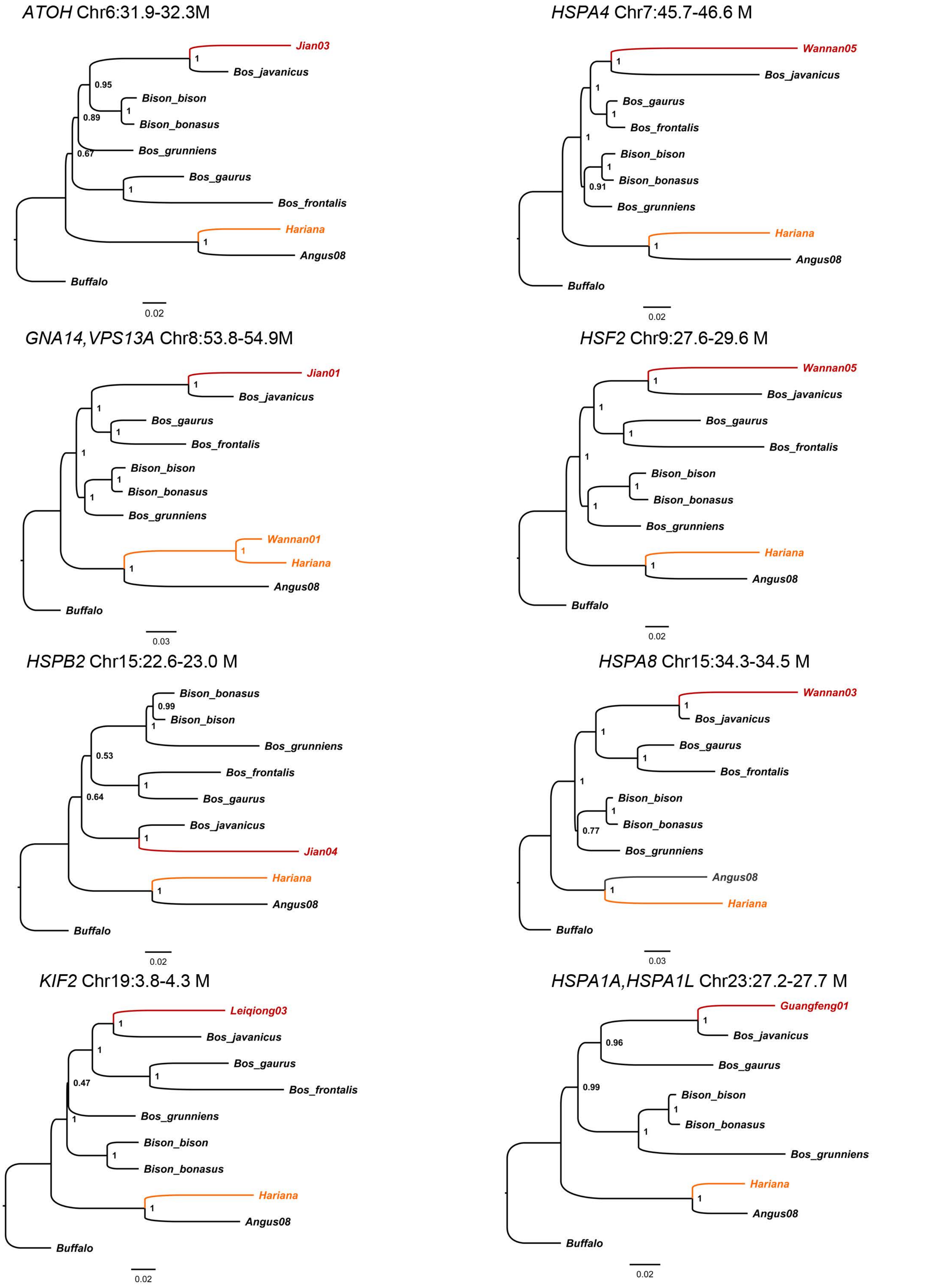

Supplementary Figure 19. Phylogenetic analyses of eight introgressed segments involving in the thermotolerance in Chinese indicine cattle genomes. Each phylogenetic tree is named by gene name and appromimate genomic position. Detail information of investigated sequences is presented in Supplementary Table 23. Seven Bovini haploids were: Banteng (*Bos javanicus*), Gaurus (*Bos gaurus*), Gayal (*Bos frontalis*), Yak (*Bos grunniens*), American Bision (*Bison bison*), Wisent (*Bison bonasus*), and Buffalo (*Bubalus bubalis*). Indian indicine postulated as pure (orange letters) and Chiniese indicine with estimated cattle introgression for this segment (red letters).

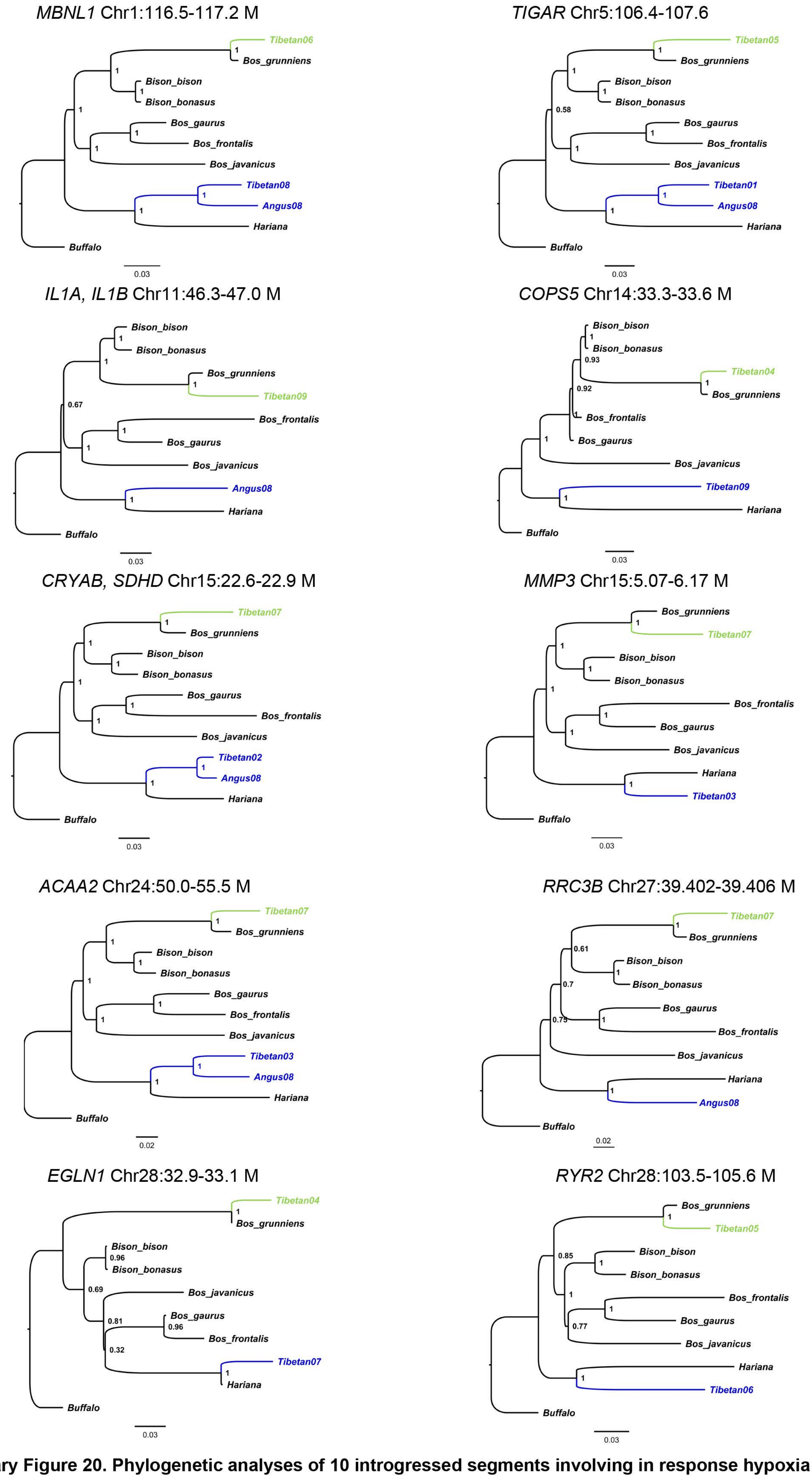

Supplementary Figure 20. Phylogenetic analyses of 10 introgressed segments involving in response hypoxia in Tibetan taurine cattle genomes. Each phylogenetic tree is named by gene name and appromimate genomic position. Detail information of investigated sequences is presented in Supplementary Table 28. Seven Bovini haploids were: Banteng (*Bos javanicus*), Gaurus (*Bos gaurus*), Gayal (*Bos frontalis*), Yak (*Bos grunniens*), American Bision (*Bison bison*), Wisent (*Bison bonasus*), and Buffalo (Bubalus bubalis). Taurine cattle postulated as pure (blue letters) and Tibetan cattle with estimated yak introgression for this segment (green letters).

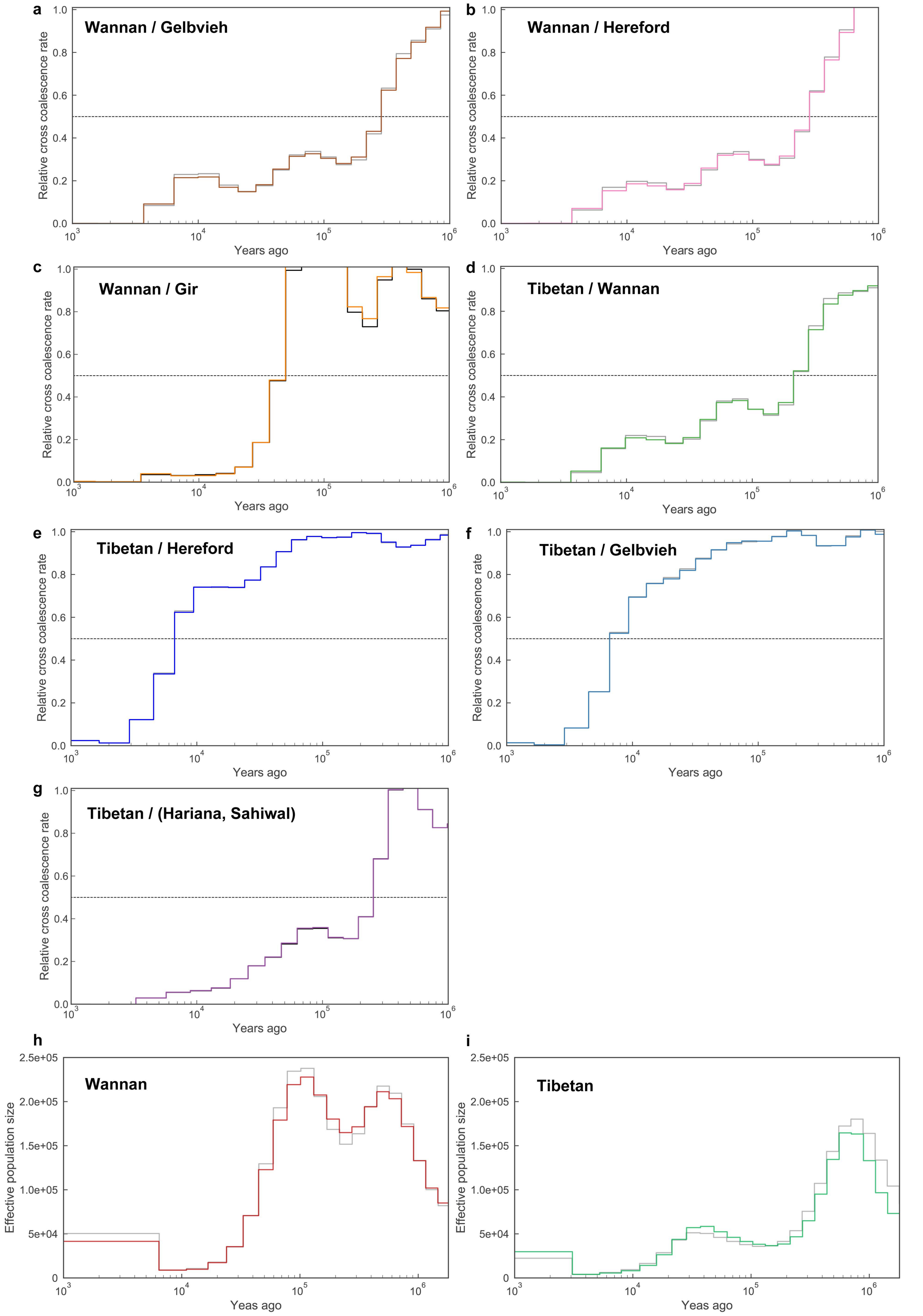

Supplementary Figure 21. Impact of introgression on demographic history using MSMC. We repeated the MSMC analysis using the same data but excluding the introgressed regions from banteng into two Chinese indicine cattle (~3.5%) and from yak into two Tibetan taurine cattle (~1.3%) (Supplementary Tables 20 and 26), respectively. The grey line in each picture represents the masked data. (a-g) Inferred relative cross coalescence rates between pairs of populations over time based on four haplotypes each from Hereford, Gelbvieh, Tibetan, Wannan, and Indian breeds (Hariana and Sahiwal). The x-axis shows time, and the y-axis shows a measure of similarity for each pair of compared populations.(h, i) Population size history inference based on four haplotypes each from high-coverage East Asian taurine (Tibetan) and Chinese indicine cattle (Wannan).

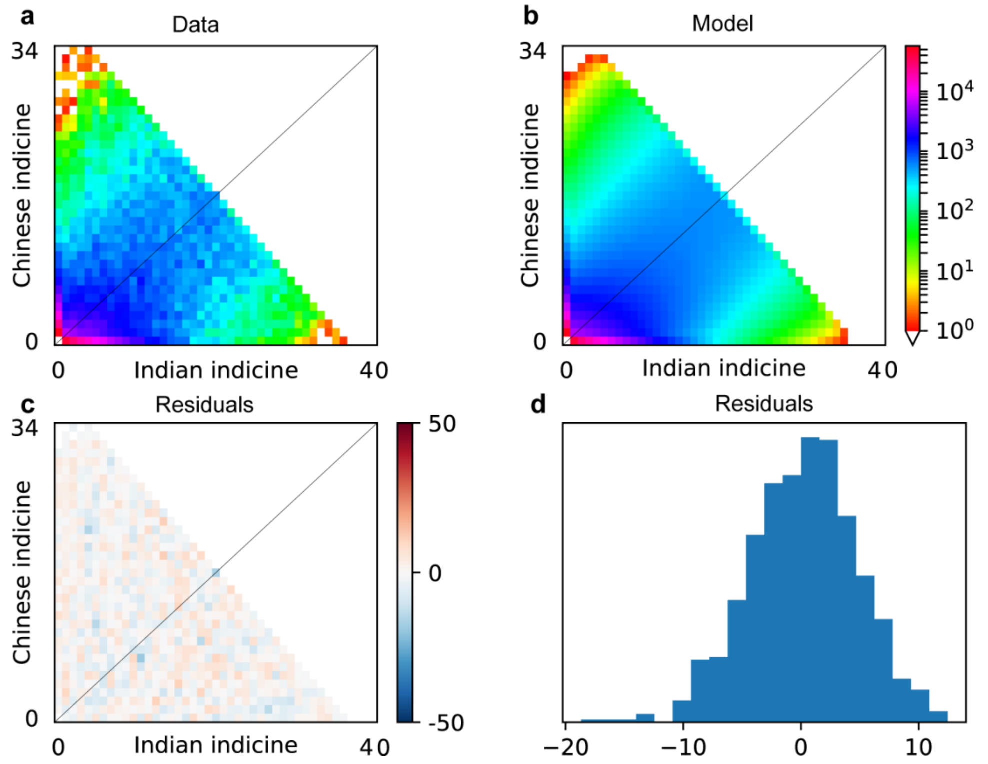

Supplementary Figure 22. Comparisons of allele frequency spectra (AFS) between the modelled and real data of Chinese indicine and Indian indicine populations using ∂a∂i. (a) Marginal AFS of the real data for each pair of populations. (b) AFS of the maximum-likelihood model simulation based on the real data. The residuals between the modelled and real data are shown in heat maps (c) and bar graphs (d).

Supplementary Tables

Supplementary Table 1. Overview of sample information and sequencing statistics

Number	Sample ID#	Accession number	Country of origin	Species/Subspecies	Mapping rate	Mean depth (X)	Gender	BioProject	Sample origin place
1	Angus01	SRR1525582	United Kingdom	*Bos taurus*	99.49%	7.14	Male	PRJNA256210	Aberdeenshire, Scotland, UK
2	Angus02	SRR1525583	United Kingdom	*Bos taurus*	99.51%	7.45	Male	PRJNA256210	Aberdeenshire, Scotland, UK
3	Angus03	SRR1525686	United Kingdom	*Bos taurus*	99.07%	6.67	Male	PRJNA256210	Aberdeenshire, Scotland, UK
4	Angus04	SRR1525687	United Kingdom	*Bos taurus*	99.68%	6.70	Male	PRJNA256210	Aberdeenshire, Scotland, UK
5	Angus05	SRR1525688	United Kingdom	*Bos taurus*	97.15%	7.25	Male	PRJNA256210	Aberdeenshire, Scotland, UK
6	Angus06	SRR1355237	United Kingdom	*Bos taurus*	99.02%	15.16	Male	PRJNA176557	Aberdeenshire, Scotland, UK
7	Angus07	SRR1365144	United Kingdom	*Bos taurus*	99.01%	17.91	Male	PRJNA176557	Aberdeenshire, Scotland, UK
8	Angus08	SRR1425124	United Kingdom	*Bos taurus*	99.12%	18.52	Male	PRJNA176557	Aberdeenshire, Scotland, UK
9	Angus09	SRR1365129	United Kingdom	*Bos taurus*	98.88%	16.92	Male	PRJNA176557	Aberdeenshire, Scotland, UK
10	Angus10	SRR1346376	United Kingdom	*Bos taurus*	99.21%	13.92	Male	PRJNA176557	Aberdeenshire, Scotland, UK
11	Red Angus01	SRR1525580	United Kingdom	*Bos taurus*	99.41%	9.52	Male	PRJNA256210	Aberdeenshire, Scotland, UK
12	Red Angus02	SRR1525581	United Kingdom	*Bos taurus*	99.48%	7.45	Male	PRJNA256210	Aberdeenshire, Scotland, UK
13	Red Angus03	SRR1525600	United Kingdom	*Bos taurus*	99.18%	7.02	Male	PRJNA256210	Aberdeenshire, Scotland, UK
14	Red Angus04	SRR1525613	United Kingdom	*Bos taurus*	99.38%	6.71	Male	PRJNA256210	Aberdeenshire, Scotland, UK
15	Red Angus05	SRR1525614	United Kingdom	*Bos taurus*	99.26%	7.31	Male	PRJNA256210	Aberdeenshire, Scotland, UK
16	Red Angus06	SRR1343172	United Kingdom	*Bos taurus*	98.61%	14.39	Male	PRJNA176557	Aberdeenshire, Scotland, UK
17	Red Angus07	SRR1355239	United Kingdom	*Bos taurus*	99.04%	15.42	Male	PRJNA176557	Aberdeenshire, Scotland, UK
18	Red Angus08	SRR1365103	United Kingdom	*Bos taurus*	98.63%	16.57	Male	PRJNA176557	Aberdeenshire, Scotland, UK
19	Red Angus09	SRR1365113	United Kingdom	*Bos taurus*	98.81%	14.93	Male	PRJNA176557	Aberdeenshire, Scotland, UK
20	Red Angus10	SRR1425153	United Kingdom	*Bos taurus*	99.42%	18.08	Male	PRJNA176557	Aberdeenshire, Scotland, UK
21	Hereford01	SRR1343160	United Kingdom	*Bos taurus*	98.01%	11.93	Male	PRJNA176557	Hereford, UK
22	Hereford02	SRR1346382	United Kingdom	*Bos taurus*	94.77%	11.57	Male	PRJNA176557	Hereford, UK
23	Hereford03	SRR1346383	United Kingdom	*Bos taurus*	98.59%	11.63	Male	PRJNA176557	Hereford, UK
24	Hereford04	SRR1346385	United Kingdom	*Bos taurus*	98.68%	12.32	Male	PRJNA176557	Hereford, UK
25	Hereford05	SRR1346387	United Kingdom	*Bos taurus*	98.90%	12.72	Male	PRJNA176557	Hereford, UK
26	Hereford06	SRR1365124	United Kingdom	*Bos taurus*	98.95%	16.12	Male	PRJNA176557	Hereford, UK
27	Hereford07	SRR1365126	United Kingdom	*Bos taurus*	99.22%	17.34	Male	PRJNA176557	Hereford, UK
28	Hereford08	SRR1365128	United Kingdom	*Bos taurus*	99.20%	16.40	Male	PRJNA176557	Hereford, UK
29	Hereford09	SRR1365131	United Kingdom	*Bos taurus*	98.91%	16.75	Male	PRJNA176557	Hereford, UK
30	Hereford10	SRR1365137	United Kingdom	*Bos taurus*	98.66%	14.54	Male	PRJNA176557	Hereford, UK
31	Devon	SRR1346378	United Kingdom	*Bos taurus*	99.11%	13.57	Male	PRJNA176557	Cork, UK
32	Salers	SRR1525608	France	*Bos taurus*	99.40%	7.17	Male	PRJNA256210	Auvergne, Franch
33	Charolais01	SRR1343167	France	*Bos taurus*	96.55%	17.70	Male	PRJNA176557	Charolais, France
34	Charolais02	SRR1343168	France	*Bos taurus*	98.50%	13.62	Male	PRJNA176557	Charolais, France
35	Charolais03	SRR1343169	France	*Bos taurus*	98.44%	11.60	Male	PRJNA176557	Charolais, France
36	Charolais04	SRR1348571	France	*Bos taurus*	99.27%	16.62	Male	PRJNA176557	Charolais, France
37	Charolais05	SRR1355258	France	*Bos taurus*	98.06%	17.57	Male	PRJNA176557	Charolais, France
38	Charolais06	SRR1365122	France	*Bos taurus*	99.02%	15.87	Male	PRJNA176557	Charolais, France
39	Limousin	SRR1365134	France	*Bos taurus*	98.47%	13.95	Male	PRJNA176557	Limousin, France
40	Maine Anjou01	SRR1355238	France	*Bos taurus*	98.73%	14.15	Male	PRJNA176557	Lyon, France
41	Maine Anjou02	SRR1355245	France	*Bos taurus*	99.37%	15.06	Male	PRJNA176557	Lyon, France
42	Maine Anjou03	SRR1355259	France	*Bos taurus*	95.23%	17.91	Male	PRJNA176557	Lyon, France
43	Maine Anjou04	SRR1365125	France	*Bos taurus*	91.94%	15.72	Male	PRJNA176557	Lyon, France
44	Maine Anjou05	SRR1365127	France	*Bos taurus*	99.03%	16.34	Male	PRJNA176557	Lyon, France
45	Maine Anjou06	SRR1365130	France	*Bos taurus*	99.16%	17.55	Male	PRJNA176557	Lyon, France
46	Piedmontese01	SRR1525596	Italy	*Bos taurus*	99.43%	8.22	Male	PRJNA256210	Piedmontese, Italy
47	Piedmontese02	SRR1525599	Italy	*Bos taurus*	99.47%	6.40	Male	PRJNA256210	Piedmontese, Italy
48	Piedmontese03	SRR1525603	Italy	*Bos taurus*	99.39%	7.81	Male	PRJNA256210	Piedmontese, Italy
49	Piedmontese04	SRR1525604	Italy	*Bos taurus*	99.48%	6.89	Male	PRJNA256210	Piedmontese, Italy
50	Piedmontese05	SRR1525606	Italy	*Bos taurus*	99.46%	8.34	Male	PRJNA256210	Piedmontese, Italy
51	Gelbvieh01	SRR1343161	Germany	*Bos taurus*	98.36%	12.85	Male	PRJNA176557	München, Bavaria, Germany
52	Gelbvieh02	SRR1343162	Germany	*Bos taurus*	97.00%	13.32	Male	PRJNA176557	München, Bavaria, Germany
53	Gelbvieh03	SRR1343164	Germany	*Bos taurus*	94.55%	15.72	Male	PRJNA176557	München, Bavaria, Germany
54	Gelbvieh04	SRR1343165	Germany	*Bos taurus*	98.58%	25.26	Male	PRJNA176557	München, Bavaria, Germany
55	Gelbvieh05	SRR1343166	Germany	*Bos taurus*	98.78%	10.66	Male	PRJNA176557	München, Bavaria, Germany
56	Gelbvieh06	SRR1365112	Germany	*Bos taurus*	99.14%	15.28	Male	PRJNA176557	München, Bavaria, Germany
57	Gelbvieh07	SRR1425154	Germany	*Bos taurus*	99.33%	17.67	Male	PRJNA176557	München, Bavaria, Germany
58	Gelbvieh08	SRR1355236	Germany	*Bos taurus*	95.64%	15.02	Male	PRJNA176557	München, Bavaria, Germany
59	Gelbvieh09	SRR1355240	Germany	*Bos taurus*	98.00%	15.08	Male	PRJNA176557	München, Bavaria, Germany
60	Gelbvieh10	SRR1355260	Germany	*Bos taurus*	99.08%	18.99	Male	PRJNA176557	München, Bavaria, Germany
61	Simmental01	SRR1525617	Switzerland	*Bos taurus*	98.93%	14.90	Male	PRJNA256210	Geneve, Switzerland
62	Simmental02	SRR1525618	Switzerland	*Bos taurus*	98.84%	13.33	Male	PRJNA256210	Geneve, Switzerland
63	Simmental03	SRR1525619	Switzerland	*Bos taurus*	97.34%	13.70	Male	PRJNA256210	Geneve, Switzerland
64	Simmental04	SRR1525620	Switzerland	*Bos taurus*	99.02%	14.80	Male	PRJNA256210	Geneve, Switzerland
65	Simmental05	SRR1525621	Switzerland	*Bos taurus*	98.31%	13.16	Male	PRJNA256210	Geneve, Switzerland
66	Simmental06	SRR1525700	Switzerland	*Bos taurus*	98.91%	14.33	Male	PRJNA256210	Geneve, Switzerland
67	Simmental07	SRR1525701	Switzerland	*Bos taurus*	98.80%	14.39	Male	PRJNA256210	Geneve, Switzerland
68	Simmental08	SRR1525702	Switzerland	*Bos taurus*	99.19%	15.14	Male	PRJNA256210	Geneve, Switzerland
69	Simmental09	SRR1525703	Switzerland	*Bos taurus*	93.80%	17.25	Male	PRJNA256210	Geneve, Switzerland
70	Simmental10	SRR1525705	Switzerland	*Bos taurus*	98.78%	15.41	Male	PRJNA256210	Geneve, Switzerland
71	Jersey01	SRR2016774	Channel Islands	*Bos taurus*	99.74%	7.74	Male	PRJNA277147	Channel Island of Jersey, UK
72	Jersey02	SRR2016776	Channel Islands	*Bos taurus*	99.70%	9.89	Male	PRJNA277147	Channel Island of Jersey, UK
73	Jersey03	SRR2016799	Channel Islands	*Bos taurus*	99.84%	3.92	Male	PRJNA277147	Channel Island of Jersey, UK
74	Holstein01	SRR1346386	Netherlands	*Bos taurus*	99.17%	13.28	Male	PRJNA176557	De Kooy, North Holland, Holland
75	Holstein02	SRR1346390	Netherlands	*Bos taurus*	99.12%	12.74	Male	PRJNA176557	De Kooy, North Holland, Holland
76	Holstein03	SRR1346392	Netherlands	*Bos taurus*	98.75%	10.44	Male	PRJNA176557	De Kooy, North Holland, Holland
77	Holstein04	SRR1348583	Netherlands	*Bos taurus*	99.03%	24.35	Male	PRJNA176557	De Kooy, North Holland, Holland
78	Holstein05	SRR1348592	Netherlands	*Bos taurus*	99.65%	13.75	Male	PRJNA176557	De Kooy, North Holland, Holland
79	Holstein06	SRR1365147	Netherlands	*Bos taurus*	98.49%	18.44	Male	PRJNA176557	De Kooy, North Holland, Holland
80	Holstein07	SRR1425129	Netherlands	*Bos taurus*	99.06%	17.19	Male	PRJNA176557	De Kooy, North Holland, Holland
81	Holstein08	SRR1425133	Netherlands	*Bos taurus*	98.95%	17.27	Male	PRJNA176557	De Kooy, North Holland, Holland
82	Holstein09	SRR1425134	Netherlands	*Bos taurus*	99.02%	17.03	Male	PRJNA176557	De Kooy, North Holland, Holland
83	Holstein10	SRR1425144	Netherlands	*Bos taurus*	99.05%	17.19	Male	PRJNA176557	De Kooy, North Holland, Holland
84	Rashoki01	ERR454987	Iran	*Bos taurus*	99.89%	14.08	Female	PRJEB5462	Sanandaj, Iran
85	Rashoki02	ERR454988	Iran	*Bos taurus*	99.90%	12.63	Female	PRJEB5462	Divandare, Iran
86	Rashoki03	ERR454989	Iran	*Bos taurus*	99.91%	13.94	Female	PRJEB5462	Marivan, Iran
87	Rashoki04	ERR454991	Iran	*Bos taurus*	99.75%	16.58	Female	PRJEB5462	Divandar, Iran
88	Rashoki05	ERR454992	Iran	*Bos taurus*	99.86%	14.63	Female	PRJEB5462	Sanandajl, ran
89	Rashoki06	ERR454993	Iran	*Bos taurus*	99.91%	12.34	Female	PRJEB5462	Marivan, Iran
90	Rashoki07	ERR454994	Iran	*Bos taurus*	99.90%	13.87	Female	PRJEB5462	Marivan, Iran
91	Rashoki08	ERR454990	Iran	*Bos taurus*	99.89%	13.60	Female	PRJEB5462	Marivan, Iran

92	Hanwoo01	SRR934415	Korea	*Bos taurus*	99.44%	14.35	Male	PRJNA210519	Chuncheon, Kangwon-do, Korea
93	Hanwoo02	SRR934417	Korea	*Bos taurus*	99.38%	14.24	Male	PRJNA210519	Chuncheon, Kangwon-do, Korea
94	Hanwoo03	SRR934418	Korea	*Bos taurus*	99.34%	15.00	Male	PRJNA210519	Chuncheon, Kangwon-do, Korea
95	Hanwoo04	SRR934419	Korea	*Bos taurus*	99.43%	13.89	Male	PRJNA210519	Chuncheon, Kangwon-do, Korea
96	Hanwoo05	SRR934432	Korea	*Bos taurus*	99.46%	15.02	Male	PRJNA210519	Chuncheon, Kangwon-do, Korea
97	Hanwoo06	SRR934433	Korea	*Bos taurus*	98.71%	13.49	Male	PRJNA210519	Chuncheon, Kangwon-do, Korea
98	Hanwoo07	SRR934434	Korea	*Bos taurus*	99.45%	13.75	Male	PRJNA210519	Chuncheon, Kangwon-do, Korea
99	Hanwoo08	SRR934435	Korea	*Bos taurus*	99.49%	12.44	Male	PRJNA210519	Chuncheon, Kangwon-do, Korea
100	Hanwoo09	SRR934436	Korea	*Bos taurus*	99.39%	12.36	Male	PRJNA210519	Chuncheon, Kangwon-do, Korea
101	Hanwoo10	SRR934437	Korea	*Bos taurus*	99.48%	12.82	Male	PRJNA210519	Chuncheon, Kangwon-do, Korea
102	Hanwoo11	SRR934395	Korea	*Bos taurus*	99.31%	10.03	Female	PRJNA210523	Chuncheon, Kangwon-do, Korea
103	Hanwoo12	SRR934397	Korea	*Bos taurus*	99.32%	8.69	Female	PRJNA210523	Chuncheon, Kangwon-do, Korea
104	Hanwoo13	SRR934398	Korea	*Bos taurus*	99.11%	10.68	Female	PRJNA210523	Chuncheon, Kangwon-do, Korea
105	Hanwoo14	SRR934400	Korea	*Bos taurus*	99.44%	13.33	Female	PRJNA210523	Chuncheon, Kangwon-do, Korea
106	Hanwoo15	SRR934401	Korea	*Bos taurus*	99.52%	14.63	Female	PRJNA210523	Chuncheon, Kangwon-do, Korea
107	Hanwoo16	SRR934402	Korea	*Bos taurus*	98.68%	12.00	Female	PRJNA210523	Chuncheon, Kangwon-do, Korea
108	Hanwoo17	SRR934403	Korea	*Bos taurus*	99.24%	10.76	Female	PRJNA210523	Chuncheon, Kangwon-do, Korea
109	Hanwoo18	SRR934404	Korea	*Bos taurus*	99.45%	12.63	Female	PRJNA210523	Chuncheon, Kangwon-do, Korea
110	Kuchinoshima	DRR000429-32	Japan	*Bos taurus*	95.75%	23.22	Male	PRJDA48395	Kuchinoshima, Japan
111	Mishima01	DRR001771,78,81	Japan	*Bos taurus*	94.24%	13.73	Male	PRJDB2660	Mishima Island, Japan
112	Mishima02	DRR001768,75,82	Japan	*Bos taurus*	94.27%	14.10	Female	PRJDB2661	Mishima Island, Japan
113	Mishima03	DRR001763,65	Japan	*Bos taurus*	77.53%	18.79	Female	PRJDB2662	Mishima Island, Japan
114	Mishima04	DRR001770,77,80	Japan	*Bos taurus*	94.15%	14.98	Female	PRJDB2663	Mishima Island, Japan
115	Mishima05	DRR001762,72,74	Japan	*Bos taurus*	85.66%	19.24	Female	PRJDB2665	Mishima Island, Japan
116	Mishima06	DRR001769,76,79	Japan	*Bos taurus*	94.44%	16.45	Female	PRJDB2666	Mishima Island, Japan
117	Mishima07	DRR001764,66	Japan	*Bos taurus*	85.23%	16.17	Female	PRJDB2667	Mishima Island, Japan
118	Yanbian	SRR5507273	China	*Bos taurus*	99.65%	10.64	Female	PRJNA379859	Yanbian, Jilin, China
119	Chaidamu01	SRR5507272	China	*Bos taurus*	99.68%	12.23	Male	PRJNA379859	Geermu, Haixi, Qinghai, China
120	Chaidamu02	SRR5507271	China	*Bos taurus*	99.67%	10.14	Male	PRJNA379859	Geermu, Haixi, Qinghai, China
121	Chaidamu03	SRR5507270	China	*Bos taurus*	99.38%	11.51	Male	PRJNA379859	Geermu, Haixi, Qinghai, China
122	Chaidamu04	SRR5507269	China	*Bos taurus*	99.80%	13.38	Male	PRJNA379859	Geermu, Haixi, Qinghai, China
123	Chaidamu05	SRR5507268	China	*Bos taurus*	99.80%	10.65	Male	PRJNA379859	Geermu, Haixi, Qinghai, China
124	Mongolian01	SRR5507267	China	*Bos taurus*	99.81%	11.72	Male	PRJNA379859	Kuerl, Bayingolin, Xinjiang, China
125	Mongolian02	SRR5507266	China	*Bos taurus*	99.71%	11.88	Male	PRJNA379859	Kuerl, Bayingolin, Xinjiang, China
126	Mongolian03	SRR5507265	China	*Bos taurus*	99.70%	13.07	Male	PRJNA379859	Kuerl, Bayingolin, Xinjiang, China
127	Mongolian04	SRR5507264	China	*Bos taurus*	99.76%	12.42	Male	PRJNA379859	Kuerl, Bayingolin, Xinjiang, China
128	Mongolian05	SRR5507263	China	*Bos taurus*	99.77%	12.48	Male	PRJNA379859	Kuerl, Bayingolin, Xinjiang, China
129	Mongolian06	SRR5507262	China	*Bos taurus*	99.74%	12.57	Male	PRJNA379859	Kuerl, Bayingolin, Xinjiang, China
130	Mongolian07	SRR5507261	China	*Bos taurus*	99.75%	11.11	Male	PRJNA379859	Kuerl, Bayingolin, Xinjiang, China
131	Kazakh01	SRR5507260	China	*Bos taurus*	99.78%	13.11	Male	PRJNA379859	Yining,Yili, Xinjing, China
132	Kazakh02	SRR5507259	China	*Bos taurus*	99.79%	12.18	Male	PRJNA379859	Yining,Yili, Xinjing, China
133	Kazakh03	SRR5507258	China	*Bos taurus*	99.83%	12.80	Male	PRJNA379859	Yining,Yili, Xinjing, China
134	Kazakh04	SRR5507257	China	*Bos taurus*	99.84%	9.04	Male	PRJNA379859	Yining,Yili, Xinjing, China
135	Kazakh05	SRR5507256	China	*Bos taurus*	99.77%	12.14	Male	PRJNA379859	Yining,Yili, Xinjing, China
136	Kazakh06	SRR5507255	China	*Bos taurus*	99.56%	11.01	Male	PRJNA379859	Yining,Yili, Xinjing, China
137	Kazakh07	SRR5507254	China	*Bos taurus*	99.81%	10.50	Male	PRJNA379859	Yining,Yili, Xinjing, China
138	Kazakh08	SRR5507253	China	*Bos taurus*	99.79%	11.95	Male	PRJNA379859	Yining,Yili, Xinjing, China
139	Kazakh09	SRR5507252	China	*Bos taurus*	99.57%	10.40	Male	PRJNA379859	Yining,Yili, Xinjing, China
140	Tibetan01	SRR5507251	China	*Bos taurus*	99.78%	11.27	Male	PRJNA379859	Changdu,Xizang, China
141	Tibetan02	SRR5507250	China	*Bos taurus*	99.75%	11.30	Male	PRJNA379859	Changdu,Xizang, China
142	Tibetan03	SRR6024571,SRR5507249	China	*Bos taurus*	99.84%	27.06	Male	PRJNA379859	Changdu,Xizang, China
143	Tibetan04	SRR6024572,SRR5507248	China	*Bos taurus*	99.03%	25.36	Male	PRJNA379859	Changdu,Xizang, China
144	Tibetan05	SRR5507247	China	*Bos taurus*	99.85%	11.36	Male	PRJNA379859	Changdu,Xizang, China
145	Tibetan06	SRR5507246	China	*Bos taurus*	98.45%	10.58	Male	PRJNA379859	Changdu,Xizang, China
146	Tibetan07	SRR5507245	China	*Bos taurus*	99.84%	11.19	Male	PRJNA379859	Changdu,Xizang, China
147	Tibetan08	SRR5507244	China	*Bos taurus*	99.82%	11.42	Male	PRJNA379859	Changdu,Xizang, China
148	Tibetan09	SRR5507243	China	*Bos taurus*	99.84%	14.20	Male	PRJNA379859	Changdu,Xizang, China
149	Luxi01	SRR5507242	China	*Bos taurus x Bos indicus*	99.73%	11.75	Male	PRJNA379859	Jining, Shandong, China
150	Luxi02	SRR5507241	China	*Bos taurus x Bos indicus*	99.47%	10.81	Male	PRJNA379859	Jining, Shandong, China
151	Luxi03	SRR5507240	China	*Bos taurus x Bos indicus*	99.50%	9.61	Male	PRJNA379859	Jining, Shandong, China
152	Luxi04	SRR5507239	China	*Bos taurus x Bos indicus*	99.48%	9.10	Male	PRJNA379859	Jining, Shandong, China
153	Luxi05	SRR5507238	China	*Bos taurus x Bos indicus*	99.62%	11.06	Male	PRJNA379859	Jining, Shandong, China
154	Nanyang01	SRR5507237	China	*Bos taurus x Bos indicus*	99.26%	10.16	Female	PRJNA379859	Nanyang, Henan, China
155	Nanyang02	SRR5507236	China	*Bos taurus x Bos indicus*	99.70%	9.75	Male	PRJNA379859	Nanyang, Henan, China
156	Nanyang03	SRR5507235	China	*Bos taurus x Bos indicus*	99.62%	10.87	Male	PRJNA379859	Nanyang, Henan, China
157	Nanyang04	SRR5507234	China	*Bos taurus x Bos indicus*	99.23%	10.49	Male	PRJNA379859	Nanyang, Henan, China
158	Nanyang05	SRR5507233	China	*Bos taurus x Bos indicus*	99.72%	10.61	Male	PRJNA379859	Nanyang, Henan, China
159	Jiaxian Red01	SRR5507232	China	*Bos taurus x Bos indicus*	98.92%	12.36	Male	PRJNA379859	Jiaxian, Pingdingshan, Henan, China
160	Jiaxian Red02	SRR5507231	China	*Bos taurus x Bos indicus*	99.78%	12.69	Male	PRJNA379859	Jiaxian, Pingdingshan, Henan, China
161	Jiaxian Red03	SRR5507230	China	*Bos taurus x Bos indicus*	99.82%	12.08	Male	PRJNA379859	Jiaxian, Pingdingshan, Henan, China
162	Jiaxian Red04	SRR5507229	China	*Bos taurus x Bos indicus*	99.81%	14.38	Male	PRJNA379859	Jiaxian, Pingdingshan, Henan, China
163	Jiaxian Red05	SRR5507228	China	*Bos taurus x Bos indicus*	97.73%	12.08	Male	PRJNA379859	Jiaxian, Pingdingshan, Henan, China
164	Bohai Black01	SRR5507227	China	*Bos taurus x Bos indicus*	99.78%	10.09	Male	PRJNA379859	Binzhou, Shandong, China
165	Bohai Black02	SRR5507226	China	*Bos taurus x Bos indicus*	99.75%	10.25	Male	PRJNA379859	Binzhou, Shandong, China
166	Bohai Black03	SRR5507225	China	*Bos taurus x Bos indicus*	99.81%	11.45	Male	PRJNA379859	Binzhou, Shandong, China
167	Bohai Black04	SRR5507224	China	*Bos taurus x Bos indicus*	99.80%	11.11	Male	PRJNA379859	Binzhou, Shandong, China
168	Bohai Black05	SRR5507223	China	*Bos taurus x Bos indicus*	87.22%	9.93	Female	PRJNA379859	Binzhou, Shandong, China
169	Lingnan01	SRR5507222	China	*Bos taurus x Bos indicus*	99.73%	11.39	Male	PRJNA379859	Shangluo, Shanxi, China
170	Lingnan02	SRR5507221	China	*Bos taurus x Bos indicus*	99.65%	9.08	Male	PRJNA379859	Shangluo, Shanxi, China
171	Lingnan03	SRR5507220	China	*Bos taurus x Bos indicus*	99.62%	10.87	Male	PRJNA379859	Shangluo, Shanxi, China
172	Lingnan04	SRR5507219	China	*Bos taurus x Bos indicus*	99.71%	10.91	Male	PRJNA379859	Shangluo, Shanxi, China
173	Lingnan05	SRR5507218	China	*Bos taurus x Bos indicus*	99.67%	9.51	Male	PRJNA379859	Shangluo, Shanxi, China
174	Lingnan06	SRR5507217	China	*Bos taurus x Bos indicus*	99.61%	9.53	Male	PRJNA379859	Shangluo, Shanxi, China
175	Lingnan07	SRR5507216	China	*Bos taurus x Bos indicus*	99.62%	11.01	Male	PRJNA379859	Shangluo, Shanxi, China
176	Lingnan08	SRR5507215	China	*Bos taurus x Bos indicus*	99.34%	10.74	Male	PRJNA379859	Shangluo, Shanxi, China
177	Zaobei01	SRR5507214	China	*Bos taurus x Bos indicus*	99.66%	11.37	Male	PRJNA379859	Zaoyang, Xiangyang, Hubei, China
178	Zaobei02	SRR5507213	China	*Bos taurus x Bos indicus*	99.40%	9.98	Male	PRJNA379859	Zaoyang, Xiangyang, Hubei, China
179	Zaobei03	SRR5507212	China	*Bos taurus x Bos indicus*	99.54%	11.12	Male	PRJNA379859	Zaoyang, Xiangyang, Hubei, China
180	Zaobei04	SRR5507211	China	*Bos taurus x Bos indicus*	99.73%	10.37	Male	PRJNA379859	Zaoyang, Xiangyang, Hubei, China
181	Zaobei05	SRR5507210	China	*Bos taurus x Bos indicus*	99.82%	11.31	Male	PRJNA379859	Zaoyang, Xiangyang, Hubei, China
182	Bashan01	SRR5507209	China	*Bos taurus x Bos indicus*	99.81%	11.52	Male	PRJNA379859	Xuanhan, Dazhou, Sichuan, China
183	Bashan02	SRR5507208	China	*Bos taurus x Bos indicus*	99.78%	10.63	Male	PRJNA379859	Xuanhan, Dazhou, Sichuan, China
184	Bashan03	SRR5507207	China	*Bos taurus x Bos indicus*	99.77%	12.21	Male	PRJNA379859	Xixiang, Hanzhong, Shaanxi, China
185	Bashan04	SRR5507206	China	*Bos taurus x Bos indicus*	99.73%	10.19	Male	PRJNA379859	Xixiang, Hanzhong, Shaanxi, China
186	Bashan05	SRR5507205	China	*Bos taurus x Bos indicus*	99.72%	11.57	Male	PRJNA379859	Xixiang, Hanzhong, Shaanxi, China
187	Wandong01	SRR5507192	China	*Bos taurus x Bos indicus*	99.09%	9.35	Male	PRJNA379859	Weining, Bijie, Guizhou, China

188	Wandong02	SRR5507191	China	*Bos taurus x Bos indicus*	99.64%	12.60	Male	PRJNA379859	Weining, Bijie, Guizhou, China
189	Dabieshan01	SRR5507194	China	*Bos taurus x Bos indicus*	99.04%	10.32	Male	PRJNA379859	Jixi, Anhui, China
190	Dabieshan02	SRR5507193	China	*Bos taurus x Bos indicus*	97.66%	8.87	Male	PRJNA379859	Jixi, Anhui, China
191	Weining01	SRR5507204	China	*Bos taurus x Bos indicus*	96.72%	9.06	Male	PRJNA379859	Weining, Bijie, Guizhou, China
192	Weining02	SRR5507203	China	*Bos taurus x Bos indicus*	99.14%	9.71	Male	PRJNA379859	Weining, Bijie, Guizhou, China
193	Weining03	SRR5507202	China	*Bos taurus x Bos indicus*	99.11%	11.47	Male	PRJNA379859	Weining, Bijie, Guizhou, China
194	Weining04	SRR5507201	China	*Bos taurus x Bos indicus*	99.09%	9.85	Male	PRJNA379859	Jixi, Anhui, China
195	Weining05	SRR5507200	China	*Bos taurus x Bos indicus*	99.28%	10.25	Male	PRJNA379859	Jixi, Anhui, China
196	Dianzhong01	SRR6024567	China	*Bos indicus*	99.38%	9.08	Female	PRJNA379859	Jiangcheng, Puer,Yunnan,China
197	Dianzhong02	SRR6024568	China	*Bos indicus*	99.52%	9.15	Female	PRJNA379859	Jiangcheng, Puer,Yunnan,China
198	Dianzhong03	SRR6024565	China	*Bos indicus*	99.34%	8.37	Female	PRJNA379859	Jiangcheng, Puer,Yunnan,China
199	Dianzhong04	SRR6024566	China	*Bos indicus*	99.34%	8.78	Male	PRJNA379859	Jiangcheng, Puer,Yunnan,China
200	Dianzhong05	SRR6024563	China	*Bos indicus*	99.67%	9.76	Female	PRJNA379859	Jiangcheng, Puer,Yunnan,China
201	Dianzhong06	SRR6024564	China	*Bos indicus*	99.66%	9.01	Female	PRJNA379859	Jiangcheng, Puer,Yunnan,China
202	Wenshan01	SRR6024561	China	*Bos indicus*	99.60%	10.51	Male	PRJNA379859	Guangnan,Wenshan,China
203	Wenshan03	SRR6024562	China	*Bos indicus*	99.41%	11.01	Male	PRJNA379859	Guangnan,Wenshan,China
204	Wenshan04	SRR6024569	China	*Bos indicus*	98.60%	10.98	Male	PRJNA379859	Guangnan,Wenshan,China
205	Wenshan05	SRR6024570	China	*Bos indicus*	99.68%	11.62	Male	PRJNA379859	Guangnan,Wenshan,China
206	Wenshan06	SRR6024577	China	*Bos indicus*	99.67%	11.54	Male	PRJNA379859	Guangnan,Wenshan,China
207	Wenshan07	SRR6024578	China	*Bos indicus*	99.70%	14.46	Male	PRJNA379859	Guangnan,Wenshan,China
208	Wenshan08	SRR6024575	China	*Bos indicus*	99.63%	10.78	Male	PRJNA379859	Guangnan,Wenshan,China
209	Wenshan09	SRR6024576	China	*Bos indicus*	99.72%	8.29	Male	PRJNA379859	Guangnan,Wenshan,China
210	Wannan01	SRR6024573,SRR5507199	China	*Bos indicus*	99.35%	23.96	Male	PRJNA379859	Jixi, Anhui, China
211	Wannan02	SRR6024574,SRR5507198	China	*Bos indicus*	99.37%	24.32	Male	PRJNA379859	Jixi, Anhui, China
212	Wannan03	SRR5507197	China	*Bos indicus*	99.03%	10.31	Male	PRJNA379859	Jixi, Anhui, China
213	Wannan04	SRR5507196	China	*Bos indicus*	98.99%	11.43	Male	PRJNA379859	Jixi, Anhui, China
214	Wannan05	SRR5507195	China	*Bos indicus*	99.39%	12.49	Male	PRJNA379859	Jixi, Anhui, China
215	Guangfeng01	SRR5507286	China	*Bos indicus*	99.80%	10.05	Male	PRJNA379859	Guangfeng, Shangrao, Jiangxi, China
216	Guangfeng02	SRR5507285	China	*Bos indicus*	99.81%	10.84	Male	PRJNA379859	Guangfeng, Shangrao, Jiangxi, China
217	Guangfeng03	SRR5507284	China	*Bos indicus*	99.78%	9.89	Male	PRJNA379859	Guangfeng, Shangrao, Jiangxi, China
218	Guangfeng04	SRR5507283	China	*Bos indicus*	99.80%	12.03	Male	PRJNA379859	Guangfeng, Shangrao, Jiangxi, China
219	Ji'an01	SRR5507282	China	*Bos indicus*	99.82%	11.62	Male	PRJNA379859	Ji’an, Jiangxi, China
220	Ji'an02	SRR5507281	China	*Bos indicus*	99.70%	10.30	Male	PRJNA379859	Ji’an, Jiangxi, China
221	Ji'an03	SRR5507280	China	*Bos indicus*	99.79%	11.17	Male	PRJNA379859	Ji’an, Jiangxi, China
222	Ji'an04	SRR5507279	China	*Bos indicus*	99.83%	11.95	Male	PRJNA379859	Ji’an, Jiangxi, China
223	Jinjiang01	SRR5507278	China	*Bos indicus*	99.80%	11.27	Male	PRJNA379859	Gaoan, Yichuan, Jiangxi, China
224	Jinjiang03	SRR5507275	China	*Bos indicus*	99.74%	10.37	Male	PRJNA379859	Gaoan, Yichuan, Jiangxi, China
225	Jinjiang04	SRR5507274	China	*Bos indicus*	99.80%	12.05	Male	PRJNA379859	Gaoan, Yichuan, Jiangxi, China
226	Leiqiong01	SRR5507190	China	*Bos indicus*	99.78%	10.80	Female	PRJNA379859	Leizhou, Guangdong,China
227	Leiqiong02	SRR5507189	China	*Bos indicus*	99.15%	12.15	Female	PRJNA379859	Leizhou, Guangdong,China
228	Leiqiong03	SRR5507188	China	*Bos indicus*	99.42%	11.46	Female	PRJNA379859	Leizhou, Guangdong,China
229	Hariana	SRR6936539	India	*Bos indicus*	99.53%	36.85	Female	PRJNA379859	Gurgaon, Haryana, India
230	Sahiwal	SRR6936540	Pakistan	*Bos indicus*	99.67%	21.99	Female	PRJNA379859	Sahiwal, Punjab, Pakistan
231	Tharparkar	SRR6936538	Pakistan	*Bos indicus*	99.29%	17.26	Female	PRJNA379859	Tharparkar, Sindh, Pakistan
232	Brahman01	SRR2016745	United States (via India)	*Bos indicus*	99.27%	4.87	Male	PRJNA277147	Brahman, West Bengal, India
233	Brahman02	SRR2016748	United States (via India)	*Bos indicus*	99.71%	8.46	Male	PRJNA277147	Brahman, West Bengal, India
234	Brahman03	SRR2016749	United States (via India)	*Bos indicus*	99.32%	6.61	Male	PRJNA277147	Brahman, West Bengal, India
235	Brahman04	SRR2016795	United States (via India)	*Bos indicus*	99.69%	6.70	Male	PRJNA277147	Brahman, West Bengal, India
236	Brahman05	SRR4002987-3008	United States (via India)	*Bos indicus*	98.87%	14.08	Male	PRJNA324822	Brahman, West Bengal, India
237	Brahman06	SRR4002969-2986	United States (via India)	*Bos indicus*	99.04%	16.62	Male	PRJNA324822	Brahman, West Bengal, India
238	Brahman07	SRR4002956-2968	United States (via India)	*Bos indicus*	98.97%	15.38	Male	PRJNA324822	Brahman, West Bengal, India
239	Brahman08	SRR4002953-55, SRR4003443-	United States (via India)	*Bos indicus*	98.35%	18.60	Male	PRJNA324822	Brahman, West Bengal, India
240	Brahman09	SRR4003434-42	United States (via India)	*Bos indicus*	98.71%	18.10	Male	PRJNA324822	Brahman, West Bengal, India
241	Gir01	SRR2016752	United States (via India)	*Bos indicus*	99.21%	13.61	Male	PRJNA277147	Brahman, West Bengal, India
242	Gir02	SRR2016753	United States (via India)	*Bos indicus*	98.37%	4.70	Male	PRJNA277147	Amblash, Gujarat, India
243	Gir03	SRR2016754	United States (via India)	*Bos indicus*	99.70%	10.66	Male	PRJNA277147	Amblash, Gujarat, India
244	Gir04	SRR2016755	United States (via India)	*Bos indicus*	99.64%	6.73	Male	PRJNA277147	Amblash, Gujarat, India
245	Nelore01	SRR2016756	Brazil (via India)	*Bos indicus*	99.53%	7.19	Male	PRJNA277147	Amblash, Gujarat, India
246	Nelore02	SRR2016757	Brazil (via India)	*Bos indicus*	98.85%	5.96	Male	PRJNA277147	Nelore, Andhra, India
247	Nelore03	SRR2016759	Brazil (via India)	*Bos indicus*	99.69%	8.27	Male	PRJNA277147	Nelore, Andhra, India
248	Nelore04	SRR2016762	Brazil (via India)	*Bos indicus*	97.80%	13.86	Female	PRJNA277147	Nelore, Andhra, India
249	Ankole01	ERR320247	Uganda	*Bos taurus x Bos indicus*	99.84%	13.71	Male	PRJEB1829	Masindi, Uganda (Closest city)
250	Ankole02	ERR320264	Uganda	*Bos taurus x Bos indicus*	99.86%	14.20	Male	PRJEB1829	Hoima, Uganda (Closest city)
251	Ankole03	ERR320245	Uganda	*Bos taurus x Bos indicus*	99.88%	13.28	Female	PRJEB1829	Rukungiri, Uganda (Closest city)
252	Ankole04	ERR320251	Uganda	*Bos taurus x Bos indicus*	99.68%	13.35	Female	PRJEB1829	Kaberamaido, Ugandan (Closest city)
253	Ankole05	ERR320255	Uganda	*Bos taurus x Bos indicus*	99.85%	13.59	Female	PRJEB1829	Mubende, Uganda (Closest city)
254	Shorthorn Zebu01	ERR320241	Uganda	*Bos taurus x Bos indicus*	99.82%	13.76	Female	PRJEB1829	Mubende, Uganda (Closest city)
255	Shorthorn Zebu02	ERR320244	Uganda	*Bos taurus x Bos indicus*	99.82%	14.04	Female	PRJEB1829	Kitgum, Uganda (Closest city)
256	Shorthorn Zebu03	ERR320246	Uganda	*Bos taurus x Bos indicus*	99.83%	13.53	Female	PRJEB1829	Adjumani, Uganda (Closest city)
257	Shorthorn Zebu04	ERR320249	Uganda	*Bos taurus x Bos indicus*	99.82%	14.33	Female	PRJEB1829	Kaberamaido, Uganda (Closest city)
258	Shorthorn Zebu05	ERR320252	Uganda	*Bos taurus x Bos indicus*	99.80%	14.52	Female	PRJEB1829	Kotido, Uganda (Closest city)
259	Nganda	ERR320263	Uganda	*Bos taurus x Bos indicus*	99.83%	13.30	Female	PRJEB1829	Masaka, Uganda (Closest city)
260	Nsongora	ERR320256	Uganda	*Bos taurus x Bos indicus*	99.85%	14.65	Female	PRJEB1829	Kasese, Uganda (Closest city)
261	Banteng01	SRR4035276-79	Indonesia	*Bos javanicus*	97.00%	10.65	Male	PRJNA325061	San Diego Zoo, L. Chemnick and O.
262	Banteng02	SRR4035280-83	Indonesia	*Bos javanicus*	99.25%	13.93	Female	PRJNA325061	San Diego Zoo, L. Chemnick and O.
263	Yak01	SRR2058046	China	*Bos grunniens*	99.79%	18.61	Male	PRJNA285834	China
264	Yak02	SRR2059936	China	*Bos grunniens*	99.79%	18.61	Male	PRJNA285834	China
265	Yak03	SRR2059966	China	*Bos grunniens*	98.88%	21.70	Female	PRJNA285834	China
266	Yak04	SRR2059950	China	*Bos grunniens*	99.68%	4.92	Male	PRJNA285834	China
267	Yak05	SRR2059951	China	*Bos grunniens*	99.81%	5.64	Male	PRJNA285834	China
268	Yak06	SRR2059952	China	*Bos grunniens*	99.73%	7.40	Male	PRJNA285834	China
269	Yak07	SRR2059962	China	*Bos grunniens*	99.78%	8.66	Male	PRJNA285834	China
270	Yak08	SRR2059963	China	*Bos grunniens*	99.73%	7.43	Male	PRJNA285834	China
271	Yak09	SRR2059964	China	*Bos grunniens*	99.71%	6.48	Male	PRJNA285834	China
272	Yak10	SRR2059969	China	*Bos grunniens*	81.17%	18.70	Male	PRJNA285834	China
273	Yak11	SRR2059968	China	*Bos grunniens*	65.66%	6.20	Male	PRJNA285834	China
274	Yak12	SRR2059965	China	*Bos grunniens*	99.64%	7.63	Male	PRJNA285834	China
275	Yak13	SRR2062306	China	*Bos grunniens*	99.56%	21.37	Female	PRJNA285834	China
276	Gayal	SRR2182212	China	*Bos frontalis*	99.37%	14.37	Female	PRJNA293670	China
277	Buffalo01	SRR4477891	India	*Bubalus bubalis*	98.61%	11.61	Male	PRJNA350833	India
278	Buffalo02	SRR4477892	India	*Bubalus bubalis*	98.12%	10.96	Male	PRJNA350833	India
279	Wisent01	SRR3178074	Poland	*Bison bonasus*	99.98%	10.36	Male	PRJNA312492	Poland
280	Wisent02	SRS1439150	Poland	*Bison bonasus*	98.78%	37.34	Male	PRJNA321590	Poland
281	Wisent03	Wisent	Poland	*Bison bonasus*	99.39%	38.96	Female	PRJNA321590	Poland
282	Gaurus01	SRR4035250-7, SRR4035262,73,95	India	*Bos gaurus*	98.58%	4.12	Female	PRJNA325061	Henry Doorly Zoo, D. Armstrong and E. Louis

283	Gaurus02	SRR4035258-1, SRR4035263-72, SRR4035274-75	India	*Bos gaurus*	98.49%	7.42	Male	PRJNA325061	Henry Doorly Zoo, D. Armstrong and E. Louis
284	Bision01	SRR4035285-91	America	*Bison bision*	96.98%	9.44	Male	PRJNA325061	Henry Doorly Zoo, D. Armstrong and E. Louis
285	Bision02	SRS1437873	America	*Bison bision*	99.85%	31.79	Male	PRJNA321590	

Samples are shown in bold fonts sequenced in this study.

Supplementary Table 2. Summary information of the 260 individuals from 49 cattle breeds and other 25 individuals of the tribe Bovini.

Figure legend	Breeds	Origin region	Animal count	Study	Geographic regions	Region definitions
1	Red Angus	Great British	10	Stothard *et al* . 2015	West Europe	Cattle breeds from modern United Kingdom, Channel Islands and Netherlands.
2	Angus	Great British	10	Stothard *et al* . 2015		
3	Devon	Great British	1	Stothard *et al* . 2015		
4	Hereford	Great British	10	Stothard *et al* . 2015		
5	Holstein	Netherlands	10	Stothard *et al* . 2015		
6	Jersey	Channel Islands	3	Heaton *et al* . 2016	Central-South Europe	Cattle breeds from modern Central and South Europe, including countries of France, Italy, Germany and Switzerland.
7	Maine Anjou	France, South Europe	6	Stothard *et al* . 2015		
8	Salers	France, South Europe	1	Stothard *et al* . 2015		
9	Charolais	France, South Europe	6	Stothard *et al* . 2015		
10	Limousin	France, South Europe	1	Stothard *et al* . 2015		
11	Piedmontese	Italy, South Europe	5	Stothard *et al* . 2015		
12	Gelbvieh	Germany,Central Europe	10	Stothard *et al* . 2015		
13	Simmental	Switzerland,Central Europe	10	Stothard *et al* . 2015		
14	Rashoki	Iran, Middle East	8	NextGen project	The Middle East	Cattle from modern Iran.
15	Kazakh	Northwest China	9	This study	Northwest China	Cattle breeds from modern northwest Chinese provinces of Xinjiang and Qinghai.
16	Mongolian	Northwest China	7	This study		
17	Chaidamu	Northwest China	5	This study		
18	Tibetan	Tibet, China	9	This study	Tibet	Cattle from Tibetan Plateau.
19	Mishima	Japan, Northeast Asia	7	Tsuda *et al* . 2013	Northeast Asia	Cattle breeds from modern Korea, Japan and Northeast Chinese provinces of Jilin.
20	Kuchinoshima	Japan, Northeast Asia	1	Kawahara-Miki *et al* . 2011		
21	Hanwoo	Korea, Northeast Asia	18	Shin *et al.* 2014		
22	Yanbian	Jilin, China, Northeast Asia	1	This study		
23	Luxi	North China	5	This study	North-Central China	Cattle breeds from modern Chinese provinces of Shandong, Henan, Shaanxi, Anhui, Hubei. And Weining breed of southwest Chinese province of Guizhou.
24	Jiaxian Red	North China	5	This study		
25	Bohai Black	North China	5	This study		
26	Nanyang	North China	5	This study		
27	Bashan	Central China	5	This study		
28	Lingnan	Central China	8	This study		
29	Wandong	Central China	2	This study		
30	Zaobei	Central China	5	This study		
31	Dabieshan	Central China	2	This study		
32	Weining	Central China	5	This study		
33	Jinjiang	South China	3	This study	South China	Cattle breeds from modern southeast Chinese provinces of Anhui, Jiangxi, Guangdong, Hainan and modern southwast Chinese province of Yunnan.
34	Wannan	South China	5	This study		
35	Guangfeng	South China	4	This study		
36	Ji'an	South China	4	This study		
37	Leiqiong	South China	3	This study		
38	Wenshan	South China	8	This study		
39	Dianzhong	South China	6	This study		
40	Hariana	India	1	This study	India-Paksitan	Cattle breeds from India and Paksitan. Three indicine cattle breeds (Gir, Brahman, and Nelore) were imported from India to modern Brazil and USA. Please note that the American Brahman was the first beef cattle breed developed in the United States.
41	Gir	India	4	Bickhart *et al* . 2016		
42	Sahiwal	Pakistan	1	This study		
43	Nelore	India	4	Bickhart *et al* . 2016; Heaton *et al* . 2016		
44	Tharparkar	Pakistan	1	This study		
45	Brahman	India	9	Bickhart *et al* . 2016		
46	Shorthorn Zebu	Uganda, Africa	5	NextGen project	Africa	Four breeds from African country of Uganda.
47	Nsongora	Uganda, Africa	1	NextGen project		
48	Ankole	Uganda, Africa	5	NextGen project		
49	Nganda	Uganda, Africa	1	NextGen project		
Sub total			260			
50	*Bos javanicus*	Southeast Asian	2	Heaton *et al* . 2016		
51	*Bos grunniens*	China	13	Qiu *et al* . 2015		
52	*Bos frontalis*	China	1	Mei *et al* . 2016		
53	*Bubalus bubalis*	India	2	Whitacre *et al* . 2017		
54	*Bison bonasus*	Poland	3	Gautier *et al* . 2016; Wang *et al* . 2015		
55	*Bos gaurus*	India	2	Heaton *et al* . 2016		
56	*Bison bision*	America	2	Heaton *et al* . 2016		
Total			285			

Supplementary Table 3. Distribution of SNPs within various genomic regions

Variant type	SNP count
Total number	60,449,904
Intergenic	36,108,854
Intronic	22,505,972
Downstream	455,885
Upstream	403,373
Exonic	611,221
UTRs	362,689
Synonymous	241,397
Non-synonymous	166,069
Splicing	1,910
Stop gain	1,984
Stop loss	188
Others	22,424

Supplementary Table 4. Tracy-Widom (TW) statistics and *P*-values for the first 10 eigenvalues in the PCA of 260 individuals. The significant *P*-values are in bold fonts.

Number	Eigenvalues	TW	*P*
1	11.698184	198.147	**0**
2	4.070315	112.424	**0**
3	3.423612	96.689	**2.41609e-277**
4	2.578969	54.138	**2.43152e-117**
5	2.439277	49.685	**2.11984e-103**
6	1.928385	15.279	**3.64978e-19**
7	1.714968	0.118	0.147729
8	1.700554	-0.145	0.195776
9	1.648901	-3.408	0.968426
10	1.639959	-3.373	0.965987

Supplementary Table 5. Tracy-Widom (TW) statistics and *P*-values for the first 10 eigenvalues in the PCA of taurine cattle. The significant *P*-values are in bold fonts.

Number	Eigenvalues	TW	*P*
1	3.959023	48.120	**1.2077e-98**
2	3.017990	31.273	**1.3819e-52**
3	2.561145	20.917	**1.3214e-29**
4	2.252381	12.531	**1.0773e-14**
5	2.075111	7.604	**2.4727e-08**
6	1.959948	4.475	**7.7183e-05**
7	1.904414	3.522	**0.0006025**
8	1.835636	1.822	0.0139572
9	1.831692	2.902	0.0020588
10	1.785953	2.103	0.0087439

Supplementary Table 6. Tracy-Widom (TW) statistics and *P*-values for the first 10 eigenvalues in the PCA of indicine cattle. The significant *P*-values are in bold fonts.

Number	Eigenvalues	TW	*P*
1	3.958	31.030	**5.37075e-52**
2	1.560	5.061	**1.97862e-05**
3	1.336	-2.798	0.902362
4	1.239	-6.460	1
5	1.226	-6.408	1
6	1.200	-6.979	1
7	1.187	-6.987	1
8	1.182	-6.587	1
9	1.176	-6.209	1
10	1.168	-5.914	1

Supplementary Table 7. Cross-validation (CV) errors for ADMIXTURE ancestry models with *K* ranging from 2 to 8.

K value	CV error
K = 2	0.23739
K = 3	0.23251
K = 4	0.23099
K = 5	0.23149
K = 6	0.22844
K = 7	0.23110
K = 8	0.23572

Supplementary Table 8. Mapping results for 147 present-day cattle mitochondrial genomes and information of the 24 reference genomes.

Number	Sample ID#	Country of origin	Region of origin	Genbank ID	Haplotype	Coverage
1	Angus09	United Kingdom	West Europe		T3	970.73
2	Hereford01	United Kingdom	West Europe		T3	1608.69
3	Hereford02	United Kingdom	West Europe		T3	298.39
4	Hereford03	United Kingdom	West Europe		T3	1127.81
5	Hereford04	United Kingdom	West Europe		T3	625.68
6	Charolais02	France	Southern Europe		T3	1050.89
7	Gelbvieh02	Germany	Northern Europe		T3	1463.44
8	Gelbvieh03	Germany	Northern Europe		T3	1440.98
9	Holstein01	Netherlands	Northern Europe		T3	768.23
10	Holstein05	Netherlands	Northern Europe		T3	1272.87
11	Holstein06	Netherlands	Northern Europe		T3	2147.33
12	Rashoki01	Iran	Middle East		T2	441.72
13	Rashoki02	Iran	Middle East		T2	281.06
14	Rashoki03	Iran	Middle East		T2	1047.21
15	Rashoki04	Iran	Middle East		T2	651.83
16	Rashoki05	Iran	Middle East		T2	329.96
17	Rashoki06	Iran	Middle East		T2	349.76
18	Rashoki07	Iran	Middle East		T2	324.09
19	Hanwoo01	Korea	Northeast Asia		T3	856.56
20	Hanwoo02	Korea	Northeast Asia		P	794.23
21	Hanwoo03	Korea	Northeast Asia		T3	866.89
22	Hanwoo04	Korea	Northeast Asia		T2	1023.72
23	Hanwoo05	Korea	Northeast Asia		T3	1021.93
24	Hanwoo06	Korea	Northeast Asia		T2	860.67
25	**Yanbian**	**China**	**Northeast Asia**		**T3**	**1161.68**
26	**Chaidamu01**	**China**	**Northern China**		**T3**	**1277.16**
27	**Chaidamu02**	**China**	**Northern China**		**T3**	**1343.93**
28	**Chaidamu03**	**China**	**Northern China**		**I1a**	**744.71**
29	**Chaidamu04**	**China**	**Northern China**		**I1a**	**931.95**
30	**Chaidamu05**	**China**	**Northern China**		**T3**	**1017.65**
31	**Mongolian01**	**China**	**Northern China**		**T3**	**2212.03**
32	**Mongolian02**	**China**	**Northern China**		**T3**	**3147.08**
33	**Mongolian03**	**China**	**Northern China**		**T3**	**2725.61**
34	**Mongolian04**	**China**	**Northern China**		**T3**	**3414.17**
35	**Mongolian05**	**China**	**Northern China**		**I1**	**2077.25**
36	**Mongolian06**	**China**	**Northern China**		**T2**	**1970.53**
37	**Mongolian07**	**China**	**Northern China**		**T3**	**1931.25**
38	**Kazakh01**	**China**	**Northern China**		**T2**	**989.18**
39	**Kazakh02**	**China**	**Northern China**		**T3**	**2599.75**
40	**Kazakh03**	**China**	**Northern China**		**T2**	**1788.21**
41	**Kazakh04**	**China**	**Northern China**		**T3**	**1602.23**
42	**Kazakh05**	**China**	**Northern China**		**I1**	**1267.30**
43	**Kazakh06**	**China**	**Northern China**		**T2**	**1551.82**
44	**Kazakh07**	**China**	**Northern China**		**T3**	**1057.20**
45	**Kazakh08**	**China**	**Northern China**		**I1**	**1496.54**
46	**Kazakh09**	**China**	**Northern China**		**T3**	**1047.36**
47	**Tibetan01**	**China**	**Northern China**		**T3**	**2661.82**
48	**Tibetan02**	**China**	**Northern China**		**T3**	**3074.78**
49	**Tibetan03**	**China**	**Northern China**		**T3**	**3057.57**
50	**Tibetan04**	**China**	**Northern China**		**T4**	**1736.17**
51	**Tibetan05**	**China**	**Northern China**		**T3**	**1954.04**
52	**Tibetan06**	**China**	**Northern China**		**T3**	**2886.23**
53	**Tibetan07**	**China**	**Northern China**		**T3**	**1106.24**
54	**Tibetan08**	**China**	**Northern China**		**Yak**	**1522.38**
55	**Tibetan09**	**China**	**Northern China**		**Yak**	**1212.29**
56	**Luxi01**	**China**	**Northern China**		**I1a**	**721.29**
57	**Luxi02**	**China**	**Northern China**		**I1a**	**702.18**
58	**Luxi03**	**China**	**Northern China**		**I1a**	**735.41**
59	**Luxi04**	**China**	**Northern China**		**I1a**	**651.77**
60	**Luxi05**	**China**	**Northern China**		**I1a**	**826.60**
61	**Nanyang01**	**China**	**Northern China**		**I1a**	**241.19**

62	**Nanyang02**	**China**	**Northern China**	**I1a**	**410.81**
63	**Nanyang03**	**China**	**Northern China**	**I1a**	**1743.20**
64	**Nanyang04**	**China**	**Northern China**	**I1a**	**1909.07**
65	**Nanyang05**	**China**	**Northern China**	**T2**	**186.42**
66	**JiaxianRed01**	**China**	**Northern China**	**I1a**	**2080.59**
67	**JiaxianRed02**	**China**	**Northern China**	**I1**	**2122.92**
68	**JiaxianRed03**	**China**	**Northern China**	**I1a**	**1410.28**
69	**JiaxianRed04**	**China**	**Northern China**	**T3**	**2125.63**
70	**JiaxianRed05**	**China**	**Northern China**	**T3**	**686.16**
71	**BohaiBlack01**	**China**	**Northern China**	**I1a**	**572.26**
72	**BohaiBlack02**	**China**	**Northern China**	**T3**	**319.56**
73	**BohaiBlack03**	**China**	**Northern China**	**T3**	**619.68**
74	**BohaiBlack04**	**China**	**Northern China**	**I1a**	**552.55**
75	**BohaiBlack05**	**China**	**Northern China**	**T3**	**608.07**
76	**Lingnan01**	**China**	**Southern China**	**I1a**	**1063.34**
77	**Lingnan02**	**China**	**Southern China**	**T2**	**154.53**
78	**Lingnan03**	**China**	**Southern China**	**I1a**	**234.64**
79	**Lingnan04**	**China**	**Southern China**	**I1a**	**370.52**
80	**Lingnan05**	**China**	**Southern China**	**I1a**	**852.47**
81	**Lingnan06**	**China**	**Southern China**	**I1a**	**232.10**
82	**Lingnan07**	**China**	**Southern China**	**I1a**	**788.15**
83	**Lingnan08**	**China**	**Southern China**	**I1a**	**1955.32**
84	**Zaobei01**	**China**	**Southern China**	**I1a**	**670.58**
85	**Zaobei02**	**China**	**Southern China**	**I1a**	**574.63**
86	**Zaobei03**	**China**	**Southern China**	**T3**	**713.06**
87	**Zaobei04**	**China**	**Southern China**	**I1a**	**539.93**
88	**Zaobei05**	**China**	**Southern China**	**I1a**	**661.25**
89	**Bashan01**	**China**	**Southern China**	**T3**	**1674.55**
90	**Bashan02**	**China**	**Southern China**	**T3**	**1324.80**
91	**Bashan03**	**China**	**Southern China**	**I1a**	**1384.07**
92	**Bashan04**	**China**	**Southern China**	**I1a**	**1521.73**
93	**Bashan05**	**China**	**Southern China**	**T3**	**1371.25**
94	**Weining01**	**China**	**Southern China**	**T3**	**1327.53**
95	**Weining02**	**China**	**Southern China**	**I1a**	**210.63**
96	**Weining03**	**China**	**Southern China**	**T3**	**1369.17**
97	**Weining04**	**China**	**Southern China**	**I1a**	**1360.17**
98	**Weining05**	**China**	**Southern China**	**I1a**	**2251.16**
99	**Dianzhong01**	**China**	**Southern China**	**T2**	**742.48**
100	**Dianzhong02**	**China**	**Southern China**	**I1a**	**996.87**
101	**Dianzhong03**	**China**	**Southern China**	**I1a**	**818.1**
102	**Dianzhong04**	**China**	**Southern China**	**I1a**	**660.45**
103	**Dianzhong05**	**China**	**Southern China**	**T2**	**643.49**
104	**Dianzhong06**	**China**	**Southern China**	**I1a**	**623.16**
105	**Wenshan01**	**China**	**Southern China**	**I1a**	**138.77**
106	**Wenshan05**	**China**	**Southern China**	**I1a**	**153.56**
107	**Wenshan06**	**China**	**Southern China**	**I1a**	**196.12**
108	**Wenshan07**	**China**	**Southern China**	**I1a**	**217.28**
109	**Wannan01**	**China**	**Southern China**	**T3**	**414.76**
110	**Wannan02**	**China**	**Southern China**	**T3**	**423.78**
111	**Wannan03**	**China**	**Southern China**	**I1a**	**485.60**
112	**Wannan04**	**China**	**Southern China**	**I1a**	**298.54**
113	**Wannan05**	**China**	**Southern China**	**I1a**	**436.59**
114	**Wandong01**	**China**	**Southern China**	**I1a**	**1664.90**
115	**Wandong02**	**China**	**Southern China**	**I1a**	**3626.71**
116	**Dabieshan01**	**China**	**Southern China**	**I1a**	**365.05**
117	**Dabieshan02**	**China**	**Southern China**	**I1a**	**2059.40**
118	**Guangfeng01**	**China**	**Southern China**	**T3**	**1292.19**
119	**Guangfeng02**	**China**	**Southern China**	**I1a**	**1500.51**
120	**Guangfeng03**	**China**	**Southern China**	**I1a**	**1372.01**
121	**Guangfeng04**	**China**	**Southern China**	**I1a**	**1679.77**
122	**Ji'an01**	**China**	**Southern China**	**T3**	**1389.03**
123	**Ji'an02**	**China**	**Southern China**	**I1a**	**1382.27**
124	**Ji'an03**	**China**	**Southern China**	**I1a**	**1274.81**
125	**Ji'an04**	**China**	**Southern China**	**I1a**	**1730.81**

126	**Jingjiang01**	**China**	**Southern China**		**I1a**	**1249.82**
127	**Jingjiang03**	**China**	**Southern China**		**I1a**	**1245.20**
128	**Jingjiang04**	**China**	**Southern China**		**I1a**	**1479.09**
129	**Leiqiong01**	**China**	**Southern China**		**I1a**	**1671.18**
130	**Leiqiong02**	**China**	**Southern China**		**I1a**	**2160.81**
131	**Leiqiong03**	**China**	**Southern China**		**I1a**	**475.16**
132	**Sahiwal3b**	**Pakistan**	**India-Pakistan**		**I2**	**1030.03**
133	**Hariana03**	**India**	**India-Pakistan**		**I1**	**1658.57**
134	**Tharparkar01**	**Pakistan**	**India-Pakistan**		**I1**	**1658.00**
135	Nelore04	Brazil (via India)	India-Pakistan		I1	621.36
136	Ankole01	Uganda	Africa		T1	606.27
137	Ankole02	Uganda	Africa		T1	591.19
138	Ankole03	Uganda	Africa		T1	846.30
139	Ankole04	Uganda	Africa		T1	268.64
140	Ankole05	Uganda	Africa		T1	696.52
141	Shorthorn Zebu01	Uganda	Africa		T1	1123.18
142	Shorthorn Zebu02	Uganda	Africa		T1	837.56
143	Shorthorn Zebu03	Uganda	Africa		T1	707.47
144	Shorthorn Zebu04	Uganda	Africa		T1	410.18
145	Shorthorn Zebu05	Uganda	Africa		T1	730.13
146	Nganda	Uganda	Africa		T1	1703.51
147	Nsongora	Uganda	Africa		T1	971.09
148	P_ GU985279			GU985279	P	
149	Q_ FJ971080			FJ971080	Q	
150	R_ FJ971084			FJ971084	R	
151	I1_NC005971			NC_005971	I1	
152	I1_ FJ971088			FJ971088	I1	
153	I1_ EU177868			EU177868	I1	
154	I2_ AF492350			AF492350	I2	
155	I2_ EU177870			EU177870	I2	
156	I2_FJ971088			FJ971088	I2	
157	T1_EU177842			EU177842	T1	
158	T1_EU177841			EU177841	T1	
159	T2_AY676856			AY676856	T2	
160	T2_EU177859			EU177859	T2	
161	T3_EU177839			EU177839	T3	
162	T3_AF492351			AF492351	T3	
163	T3a_DQ124418			DQ124418	T3a	
164	T4_NC_006853			NC_006853	T4	
165	T4_AB074964			AB074964	T4	
166	T4			UMD3.1	T4	
167	BRS			V00654	T3	
168	Bos_grunniens01			KR011113	Yak	
169	Bos_grunniens02			AY684273	Yak	
170	Bos_grunniens03			KR052524	Yak	
171	Bison_bison			EU177871	Bison	
Max coverage						1152.04
Min coverage						3626.71
Average coverage						138.77

Sample names in bold fonts were sequenced in this study.

Supplementary Table 9. Radio carbon dating details for the Shimao05 specimen.

Protein quality assessment1	
	d13C = -9.5<br>d15N = +7.0
Carbon dating results	
Conventional radiocarbon age	3590 ± 30 BP
Age cal yr BP	
(1σ, 95.4% prob.)	3975 - 3835 BP
(2σ, 68.2% prob.)	3955 - 3950 BP

Results are ISO/IEC-17025:2005 accredited. All work was performed at the Beta Analytic Radiocarbon Dating Laboratory using 4 in-house NEC accelerator mass spectrometers and 4 Thermo IRMSs. The "Conventional Radiocarbon Age" was calculated using the Libby half-life (5568 years) corrected for total isotopic fraction, and it was also used for calendar calibration where applicable. The age is rounded to the nearest 10 years and reported as radiocarbon years before present (BP), “present” = AD 1950. Results greater than the modern reference are reported as percent modern carbon (pMC). The modern reference standard was 95% of the 14C signature of NIST SRM-4990C (oxalic acid). Calculated sigmas less than 30 BP on the Conventional Radiocarbon Age are conservatively rounded up to 30. d13C values are for the material itself (not the AMS d13C). d13C and d15N values are relative to VPDB-1.

Supplementary Table 10. Overview of ancient sample information and sequencing statistics.

Sample ID	Accession	Raw total reads	Raw total bases	Clean total reads	Clean total bases	Mapping rate	Duplication	Mean depth (X)	1X	2X
Shimao01	SRR6942512	93,522,476	14,028,371,400	41,386,217	4,496,914,115	4.83 %	29.84 %	0.02	2.11 %	0.08 %
Shimao02	SRR6942513	81,996,370	12,299,455,500	37,207,468	3,843,193,609	12.44 %	36.22 %	0.07	6.11 %	0.44 %
Shimao03	SRR6942510	92,171,766	13,825,764,900	41,354,778	4,075,336,474	5.78 %	32.22 %	0.03	2.54 %	0.10 %
Shimao04	SRR6942511	81,546,778	12,232,016,700	36,549,466	3,147,474,616	47.79 %	38.48 %	0.25	19.33 %	3.91 %
Shimao05	SRR6942508	1,783,427,012	267,514,051,800	828,686,274	67,487,144,053	75.45 %	83.75 %	2.64	78.05 %	54.06 %
Shimao06	SRR6942509	90,348,666	13,552,299,900	40,860,187	4,252,414,263	4.03 %	25.08 %	0.02	1.75 %	0.06 %
Shimao07	SRR6942506	90,309,282	13,546,392,300	41,978,683	3,592,715,359	17.78 %	38.64 %	0.10	8.74 %	0.85 %
Shimao08	SRR6942507	87,860,794	13,179,119,100	38,320,227	3,701,317,363	11.69 %	37.64 %	0.05	4.86 %	0.29 %

Supplementary Table 11. Examples of the most significant *f3* statistics for cattle breeds (Mongolian and Kazakh) of Northwest China. Only breeds with more than one sample were considered as test populations in the three-population tests.

Pop A (Source population)	Pop B (Source population)	Pop C (Admixed population)	*f3* statistic	Standard error	Z
Holstein	Leiqiong	Mongolian	-0.0771	0.00159	-47.157
Angus	Leiqiong	Mongolian	-0.0763	0.00165	-44.784
Hereford	Leiqiong	Mongolian	-0.0763	0.00163	-45.284
Jersey	Leiqiong	Mongolian	-0.0760	0.00163	-45.096
Piedmontese	Leiqiong	Mongolian	-0.0736	0.00161	-44.268
Holstein	Nelore	Mongolian	-0.0751	0.00165	-44.072
Angus	Nelore	Mongolian	-0.0743	0.00173	-41.555
Jersey	Nelore	Mongolian	-0.0736	0.00169	-42.029
Hereford	Nelore	Mongolian	-0.0730	0.00169	-41.582
Simmental	Nelore	Mongolian	-0.070	0.00167	-41.608
Piedmontese	Nelore	Kazakh	-0.0460	0.00142	-32.401
Limousin	Nelore	Kazakh	-0.0486	0.00156	-30.984
Angus	Nelore	Kazakh	-0.0423	0.00138	-30.61
Holstein	Nelore	Kazakh	-0.0420	0.00138	-30.308
Hereford	Nelore	Kazakh	-0.0410	0.00138	-29.709
Simmental	Leiqiong	Kazakh	-0.0546	0.00141	-38.74
Piedmontese	Leiqiong	Kazakh	-0.0537	0.00147	-36.41
Limousin	Leiqiong	Kazakh	-0.0548	0.00162	-33.649
Hereford	Leiqiong	Kazakh	-0.0456	0.00142	-32.053
Simmental	Tibetan	Kazakh	-0.0040	0.00054	-7.368
Limousin	Tibetan	Kazakh	-0.0036	0.00078	-4.569
Jersey	Tibetan	Kazakh	-0.0022	0.00071	-3.175
Piedmontese	Tibetan	Kazakh	-0.0018	0.00053	-3.516
Gelbvieh	Tibetan	Kazakh	-0.0018	0.00058	-3.217
Simmental	Mishima	Kazakh	-0.0035	0.00078	-4.481
RedAngus	Mishima	Kazakh	-0.0025	0.00086	-3.009
Limousin	Mishima	Kazakh	-0.0006	0.00131	-0.472
Piedmontese	Mishima	Kazakh	-0.0003	0.00085	-0.373

Supplementary Table 12. Examples of the most significant *f3* statistics for various breeds of African and European taurine cattle, with Indian indicine cattle as one of the source populations. Only breeds with more than one sample were considered as test populations in the three-population tests.

Pop A (Source population)	Pop B (Source population)	Pop C (Admixed population)	*f3* statistics	Standard error	Z-score
Holstein	Nelore	Ankole	-0.07601	0.00184	-41.256
Limousin	Nelore	Ankole	-0.07543	0.00181	-41.561
MaineAnjou	Nelore	Ankole	-0.07531	0.00186	-40.382
Simmental	Nelore	Ankole	-0.07464	0.00179	-41.512
Angus	Nelore	Ankole	-0.07453	0.00183	-40.618
Simmental	Nelore	Shorthorn Zebu	-0.06405	0.00177	-36.1
MaineAnjou	Nelore	Shorthorn Zebu	-0.06376	0.00178	-35.644
Holstein	Nelore	Shorthorn Zebu	-0.06364	0.00177	-35.909
Gelbvieh	Nelore	Shorthorn Zebu	-0.06336	0.00178	-35.552
Piedmontese	Nelore	Shorthorn Zebu	-0.06321	0.00177	-35.581

Supplementary Table 13. Examples of the most significant *f3* statistics for various cattle breeds of North-Central China, with Chinese indicine cattle (Leiqiong) as one of the source populations. Only breeds with more than one sample were considered as test populations in the three-population tests.

Pop A (Source population)	Pop B (Source population)	Pop C (Admixed population)	*f3* statistics	Standard error	Z-score
Hanwoo	Leiqiong	Bashan	-0.1074	0.001397	-76.958
Hereford	Leiqiong	Bashan	-0.1065	0.001404	-75.866
Holstein	Leiqiong	Bashan	-0.1064	0.001418	-75.062
Angus	Leiqiong	Bashan	-0.1062	0.001404	-75.697
Piedmontese	Leiqiong	Bashan	-0.1059	0.001422	-74.522
Angus	Leiqiong	Bohai Black	-0.1070	0.001698	-63.036
Hereford	Leiqiong	Bohai Black	-0.1068	0.001677	-63.719
Holstein	Leiqiong	Bohai Black	-0.1064	0.001693	-62.882
Jersey	Leiqiong	Bohai Black	-0.1061	0.001724	-61.568
Simmental	Leiqiong	Bohai Black	-0.1053	0.001698	-62.075
Hereford	Leiqiong	Dabieshan	-0.0517	0.002339	-22.14
Salers	Leiqiong	Dabieshan	-0.0514	0.002367	-21.737
Hanwoo	Leiqiong	Dabieshan	-0.0514	0.002336	-22.018
Holstein	Leiqiong	Dabieshan	-0.0514	0.002342	-21.96
Piedmontese	Leiqiong	Dabieshan	-0.0513	0.002344	-21.915
Angus	Leiqiong	Jiaxian Red	-0.1137	0.001492	-76.236
Hereford	Leiqiong	Jiaxian Red	-0.1136	0.001493	-76.142
Holstein	Leiqiong	Jiaxian Red	-0.1133	0.001519	-74.62
Limousin	Leiqiong	Jiaxian Red	-0.1133	0.001553	-72.96
Jersey	Leiqiong	Jiaxian Red	-0.1128	0.001535	-73.492
Hanwoo	Leiqiong	Lingnan	-0.1069	0.001339	-79.832
Hereford	Leiqiong	Lingnan	-0.1064	0.001344	-79.17
Holstein	Leiqiong	Lingnan	-0.1061	0.001348	-78.703
Red Angus	Leiqiong	Lingnan	-0.1060	0.001355	-78.31
Jersey	Leiqiong	Lingnan	-0.1058	0.001336	-79.287
Hereford	Leiqiong	Luxi	-0.0903	0.002112	-42.786
Simmental	Leiqiong	Luxi	-0.0903	0.002121	-42.58
Angus	Leiqiong	Luxi	-0.0900	0.002103	-42.829
Holstein	Leiqiong	Luxi	-0.0895	0.002111	-42.448
Piedmontese	Leiqiong	Luxi	-0.0895	0.002109	-42.468
Hereford	Leiqiong	Nanyang	-0.1243	0.001424	-87.309
Gelbvieh	Leiqiong	Nanyang	-0.1240	0.001422	-87.251
Jersey	Leiqiong	Nanyang	-0.1239	0.001439	-86.102
Simmental	Leiqiong	Nanyang	-0.1239	0.00143	-86.661

Angus	Leiqiong	Nanyang	-0.1237	0.001416	-87.354
Holstein	Leiqiong	Wandong	-0.1183	0.002007	-58.974
Angus	Leiqiong	Wandong	-0.1174	0.001992	-58.984
RedAngus	Leiqiong	Wandong	-0.1174	0.002012	-58.355
Hereford	Leiqiong	Wandong	-0.1165	0.001985	-58.717
Piedmontese	Leiqiong	Wandong	-0.1160	0.002004	-57.907
Holstein	Leiqiong	Weining	-0.1162	0.001599	-72.693
Hereford	Leiqiong	Weining	-0.1150	0.001585	-72.58
Angus	Leiqiong	Weining	-0.1149	0.001584	-72.605
Jersey	Leiqiong	Weining	-0.1140	0.001578	-72.267
Piedmontese	Leiqiong	Weining	-0.1136	0.00159	-71.522
Simmental	Leiqiong	Zaobei	-0.0989	0.001834	-53.944
Hereford	Leiqiong	Zaobei	-0.0988	0.001839	-53.738
Angus	Leiqiong	Zaobei	-0.0985	0.001823	-54.06
Jersey	Leiqiong	Zaobei	-0.0983	0.00182	-54.006
Holstein	Leiqiong	Zaobei	-0.0982	0.001829	-53.715

Supplementary Table 14. Number of significant and non-significant three-population tests for each breed. A total of 741 tests were conducted for all possible combination of reference populations. A lack of significant tests from all possible combinations indicates isolated breed structure or high post-admixture drift, whereas an abundance of significant tests indicates that a breed (test population) is admixed or has a complex population history.

Breed	Region	No. of non-significant tests	No. of significant tests
Hereford	West Europe	741	0
Holstein	West Europe	741	0
Angus	West Europe	741	0
Red Angus	West Europe	741	0
Jersey	West Europe	741	0
Maine Anjou	Central-South Europe	741	0
Charolais	Central-South Europe	699	42
Piedmontese	Central-South Europe	741	0
Gelbvieh	Central-South Europe	741	0
Simmental	Central-South Europe	741	0
Kazakh	Northwest China	510	231
Mongolia	Northwest China	484	257
Chaidamu	Northwest China	741	0
Tibetan	Tibet	741	0
Mishima	Northeast Asia	741	0
Hanwoo	Northeast Asia	720	21
Luxi Cattle	Central-North China	594	147
Jiaxian Red	Central-North China	406	335
Bohai Black	Central-North China	377	364
Nanyang	Central-North China	447	294
Bashan	Central-North China	442	299
Lingnan	Central-North China	533	208
Wandong	Central-North China	473	268
Zaobei	Central-North China	544	197
Dabieshan	Central-North China	630	111
Weining	Central-North China	487	254
Dianzhong	South China	611	130
Wenshan	South China	736	5
Jinjiang	South China	621	120
Guangfeng	South China	672	69
Ji'an	South China	713	28
Wannan	South China	741	0
Leiqiong	South China	741	0
Gir	India-Pakistan	741	0
Nelore	America	741	0
Brahman	America	683	58
Shorthorn Zebu	Africa	595	146
Ankole	Africa	537	204

Supplementary Table 15. Five “core” cattle groups of different ancestry composition.

Ancestry composition	Number	Individuals
European taurine	28	Angus01, Angus02, Angus03, Angus04, Angus05, Angus06, Angus07, Angus08, Angus09, Red Angus01, Red Angus02, Red Angus03, Red Angus04, Red Angus05, Red Angus06, Red Angus07, Red Angus08, Red Angus09, Hereford01, Hereford02, Hereford03, Hereford04, Hereford05, Hereford06, Hereford07, Hereford08, Hereford09, Hereford10
Eurasian taurine	21	Jersey01, Jersey02, Jersey03, Limousin, Piedmontese01, Piedmontese02, Piedmontese03, Piedmontese04, Piedmontese05, Gelbvieh05, Gelbvieh06, Gelbvieh07, Gelbvieh08, Simmental01, Simmental03, Simmental05, Simmental06, Simmental07, Simmental08, Simmental09, Simmental10
East Asian taurine	24	Hanwoo01, Hanwoo02, Hanwoo03, Hanwoo04, Hanwoo05, Hanwoo06, Hanwoo07, Hanwoo08, Hanwoo09, Hanwoo10, Hanwoo11, Hanwoo12, Hanwoo13, Hanwoo14, Hanwoo15, Yanbian, Tibetan03, Tibetan04, Tibetan05, Tibetan07, Mishima01, Mishima02, Mishima03, Mishima04
Indian indicine	19	Brahman01, Brahman02, Brahman03, Brahman04, Brahman05, Brahman06, Brahman07, Brahman08, Gir01, Gir02, Gir03, Gir04, Nelore01, Nelore02, Nelore03, Nelore04, Hariana, Sahiwal, Tharparkar
Chinese indicine	17	Wannan01, Wannan02, Wannan03, Wannan04, Wannan05, Guangfeng01, Guangfeng02, Guangfeng03, Guangfeng04, Ji'an01, Ji'an02, Ji'an03, Ji'an04, Jingjiang01, Leiqiong01, Leiqiong02, Leiqiong03

Supplementary Table 16. Results of the *D* statistics tests performed to detect admixtures from ancient Chinese cattle (Y) to either W (Angus) or X. Negative *D* statistics indicate that gene flow has occurred from Y to X, and positive *D* statistics indicate that gene flow has occurred from Y to W. Significant Z-values are in bold fonts.

W	X	Y	Z	*D* statistic	Z-value
Angus	Kuchinoshima	Ancient	Buffalo	-0.0566	**-8.221**
Angus	Mishima	Ancient	Buffalo	-0.0503	**-7.902**
Angus	Hereford	Ancient	Buffalo	-0.0384	**-10.957**
Angus	Hanwoo	Ancient	Buffalo	-0.0361	**-8.475**
Angus	Yanbian	Ancient	Buffalo	-0.0207	**-3.128**
Angus	Jersey	Ancient	Buffalo	-0.0074	**-1.856**
Angus	Piedmontese	Ancient	Buffalo	-0.0063	**-1.887**
Angus	Holstein	Ancient	Buffalo	0.0122	3.809
Angus	MaineAnjou	Ancient	Buffalo	0.0156	5.046
Angus	Limousin	Ancient	Buffalo	0.0162	3.773
Angus	Simmental	Ancient	Buffalo	0.0178	5.153
Angus	Gelbvieh	Ancient	Buffalo	0.0199	6.433
Angus	Charolais	Ancient	Buffalo	0.0461	10.082
Angus	Tibetan	Ancient	Buffalo	0.09	12.890
Angus	Kazakh	Ancient	Buffalo	0.103	25.809
Angus	Chaidamu	Ancient	Buffalo	0.157	21.941
Angus	Mongolian	Ancient	Buffalo	0.2107	40.342
Angus	Bohai Black	Ancient	Buffalo	0.294	46.699
Angus	Jiaxian Red	Ancient	Buffalo	0.4086	67.782
Angus	Nanyang	Ancient	Buffalo	0.4845	100.000
Angus	Ankole	Ancient	Buffalo	0.4861	87.970
Angus	Luxi	Ancient	Buffalo	0.4895	81.984
Angus	Wandong	Ancient	Buffalo	0.5217	74.588
Angus	Weining	Ancient	Buffalo	0.5248	100.000
Angus	Bashan	Ancient	Buffalo	0.5637	100.000
Angus	Lingnan	Ancient	Buffalo	0.5803	100.000
Angus	Zaobei	Ancient	Buffalo	0.6046	100.000
Angus	Dabieshan	Ancient	Buffalo	0.7038	100.000
Angus	Jingjiang	Ancient	Buffalo	0.7317	100.000
Angus	Guangfeng	Ancient	Buffalo	0.7479	100.000
Angus	Wannan	Ancient	Buffalo	0.7577	100.000
Angus	Ji’an	Ancient	Buffalo	0.7681	100.000
Angus	Leiqiong	Ancient	Buffalo	0.7845	100.000

Supplementary Table 17. Chinese indicine-specific SNPs shared by different extant species in the tribe of Bovini. A total of 12,696,752 SNPs were specific to Chinese indicine cattle compared with the other four "core" cattle groups (Europe taurine, Eurasian taurine, East Asian taurine, and Indian indicine). ***Total shared*** **represents the cumulative number of shared Chinese indicine specific SNPs in different species in the tribe of Bovini.**

Species	Shared SNPs						
Bos javanicus	4,669,638	4,669,638	4,669,638	4,669,638	4,669,638	4,669,638	4,669,638
Bos taurus		3,661,861	3,661,861	3,661,861	3,661,861	3,661,861	3,661,861
Bos frontalis			3,532,177	3,532,177	3,532,177	3,532,177	3,532,177
Bison bonasus				2,241,654	2,241,654	2,241,654	2,241,654
Bison bison					2,206,222	2,206,222	2,206,222
Bos grunniens						2,379,053	2,379,053
Bubalus bubalis							2,100,288
Total shared	4,669,638	6,197,006	6,814,297	6,978,772	7,015,018	7,126,744	7,309,508
Unshared	8,027,114	6,499,746	5,882,455	5,717,980	5,681,734	5,570,008	53,87,244

Supplementary Table 18. Most negative and significant *f3* statistics for various of genus of *Bos* and *Bison*, with Chinese indicine cattle as the target population and *Bos javanicus* as one of the source population.

Source1	Source2	Target	*f3*	Standard error	Z-score
**Indian indicine**	**Banteng**	**Chinese indicine**	**-0.0558**	**0.0020**	**-26.97**
Indian indicine	Gaur	Chinese indicine	-0.0291	0.0017	-16.91
Indian indicine	Gayal	Chinese indicine	-0.0105	0.0021	-5.02
Indian indicine	Wisent	Chinese indicine	-0.0088	0.0014	-6.01
Indian indicine	Yak	Chinese indicine	-0.0079	0.0014	-5.35
Indian indicine	Bison	Chinese indicine	-0.0051	0.0015	-3.26
Indian indicine	European taurine	Chinese indicine	0.0833	0.0019	42.68

Supplementary Table 19. The result of *D* statistic tests performed to detect admixture from species of the tribe of Bovini (Y) to either W or X. Negative *D* statistic values indicate that gene flow has occurred from Y to X, and positive *D* statistic values indicate that gene flow has occurred from Y to W.

W	X	Y	Z	*D* statistic	Z-value
**Indian indicine**	**Chinese indicine**	**Banteng**	**Buffalo**	**-0.274**	**-51.542**
Indian indicine	Chinese indicine	Gaur	Buffalo	-0.165	-53.632
Indian indicine	Chinese indicine	Wisent	Buffalo	-0.060	-42.609
Indian indicine	Chinese indicine	Yak	Buffalo	-0.060	-35.352
Indian indicine	Chinese indicine	Gayal	Buffalo	-0.055	-7.798
Indian indicine	Chinese indicine	Bison	Buffalo	-0.039	-11.840
European taurine	Chinese indicine	Banteng	Buffalo	-0.281	-52.476

Supplementary Table 20. Statistics on introgressed intervals for 17 Chinese indicine cattle. The following values are shown for each core Chinese indicine cattle: the number of introgressed intervals (Count); the minimal (Min), maximal (Max), mean (Mean) and sum (Sum) of introgressed interval lengths. The proportion of the cattle genome that is introgressed (proportion).

Number	Sample	Count	Introgression Interval length				Proportion
			Min	Max	Mean	Sum	
1	Leiqiong01	422	2,780	4,469,509	377,755	159,412,628	0.03160
2	Leiqiong02	600	2,894	2,408,128	262,561	157,537,079	0.02847
3	Leiqiong03	505	2,974	2,415,827	284,422	143,633,351	0.03123
4	Guangfeng01	439	934	4,000,863	273,358	120,004,243	0.02379
5	Guangfeng02	427	2,802	4,078,722	323,439	138,108,727	0.02738
6	Guangfeng03	449	2,439	2,422,378	270,036	121,246,368	0.02404
7	Guangfeng04	495	2,439	3,694,015	313,889	155,375,272	0.03080
8	Ji'an01	534	3,104	2,415,827	267,835	143,024,355	0.02835
9	Ji'an02	440	4,269	2,674,867	295,351	129,954,678	0.02576
10	Ji'an03	420	3,259	3,274,144	301,378	126,578,961	0.02509
11	Ji'an04	468	1,349	2,227,940	280,785	131,407,552	0.02605
12	Jinjiang01	465	2,780	2,400,236	264,900	123,178,922	0.02442
13	Wannan01	519	2,439	3,688,508	311,523	161,680,654	0.03205
14	Wannan02	603	1,349	3,683,034	318,472	192,038,687	0.03807
15	Wannan03	465	3,135	3,688,508	305,842	142,216,994	0.02819
16	Wannan04	548	2,439	4,050,313	310,260	170,022,558	0.03371
17	Wannan05	607	2,439	3,172,076	319,446	193,904,273	0.03844
	Max	607	4,269	4,469,509	377,755	193,904,273	0.03844
	Min	420	934	2,227,940	262,561	120,004,243	0.02379
	Mean	494	2,578	3,221,464	298,897	147,607,371	0.02926
	Standard deviation	64	795	750,967	29,230	22,833,985	0.00453
	Standard error	15	192	182,136	7,089	5,538,054	0.00109

Supplementary Table 21. List of introgression intervals from *Bos javanicus* into Chinese indicine cattle detected by RFMix analyses and were shared by at least two haplotypes

Chromosome (Btau_5.0.1) start and end positions and number of introgressed haplotype (Hap(n)) and gene content of introgessed intervals.

Chr	Start (bp)	End (bp)	Length (bp)	Hap (n)	GeneID
1	3741155	4352533	611379	2	*KRTAP11-1, KRTAP7-1, LOC101906247, LOC101906373, LOC101907866, LOC101907950 , LOC525831, LOC782140, LOC785105*
1	6336595	10025102	3688508	3	*ADAMTS1, ADAMTS5, APP, CCT8, CYYR1, LOC100138661, LOC100337436, LOC100851369, LOC104970784, LOC104970786, LOC104970788, LOC104970790, LOC107131365, LOC107132174, LOC526789, LOC615183, LTN1, MAP3K7CL, N6AMT1, RWDD2B, TRNAR-CCU, USP16*
1	10025102	10327999	302898	4	*ATP5J, GABPA, JAM2, MIR155, MRPL39, TRNAQ-UUG*
1	10327999	11072293	744295	2	*LOC783459*
1	12457358	12488462	31105	4	.
1	12488462	12966974	478513	4	*LOC100847925*
1	12966974	13604661	637688	2	.
1	13604661	13745095	140435	5	.
1	13745095	14586602	841508	4	.
1	14586602	14699627	113026	2	.
1	14699627	15107801	408175	2	*NCAM2*
1	16475220	16480530	5311	7	.
1	16480530	16606207	125678	8	.
1	26554467	28203209	1648743	10	*LOC100848584, LOC101907726, ROBO1, TRNAC-ACA*
1	30979742	31242429	262688	5	.
1	31242429	31709360	466932	7	.
1	31709360	32032388	323029	4	.
1	35201160	38475303	3274144	2	*ARL13B, C1H3orf38, CGGBP1, EPHA3, HTR1F, LOC100335242, LOC101904492, LOC104970809, LOC107132179, NSUN3, PROS1, STX19*
1	38475303	39823639	1348337	4	*LOC617944*
1	41498072	42232848	734777	2	*ARL6, CRYBG3, EPHA6, GABRR3, LOC100138923, LOC100140523, LOC100140741, LOC101905242, LOC101905413, LOC101905600, LOC101906449, LOC104970812, LOC104970813, LOC783514, LOC783578, LOC783655, LOC783812, LOC783843, LOC783884, MINA, OR5AC2, OR5H6, OR5K3*
1	42232848	42287871	55024	2	*LOC100139155, LOC783917, LOC783950*
1	42287871	42321999	34129	2	*LOC100140204, LOC789957*
1	43675905	44587960	912056	8	*CMSS1, COL8A1, FILIP1L, LNP1, LOC107132180, LOC529036, NIT2, TBC1D23, TMEM30C, TOMM70A*
1	44587960	44645808	57849	6	*LOC529036*
1	45852418	45972871	120454	4	*ABI3BP, LOC104970828*
1	45972871	46323349	350479	3	*IMPG2, SENP7*
1	50275098	53969112	3694015	2	*ALCAM, BBX, CBLB, CCDC54, CD47, DZIP3, HHLA2, IFT57, KIAA1524, LOC100296365, LOC100848469, LOC100848494, LOC101910021, LOC104970838, LOC104970840, LOC781370, LOC783477, LOC785980, MIR2286, MYH15, TMSB4*
1	53969112	54032710	63599	2	*DZIP3*
1	54032710	54631881	599172	5	*GUCA1C, LOC100297006, LOC101903052, MORC1, TRAT1, TRNAC-GCA*
1	54631881	55548551	916671	3	*DPPA2, DPPA4, LOC100337136, LOC614129, LOC782629, LOC783844*
1	58842657	59881348	1038692	2	*ATP6V1A, CCDC191, DRD3, GRAMD1C, LOC100337152, LOC101908760, MIR568, NAA50, QTRTD1, SIDT1, TIGIT, USF3, ZBTB20, ZDHHC23*
1	61594908	61900615	305708	2	*LSAMP, TRNAK-UUU*
1	67072811	67682813	610003	7	*CASR, CCDC58, CD86, CSTA, EAF2, FAM162A, ILDR1, IQCB1, LOC101908792, LOC104970874, LOC104970876, LOC104970885, LOC516421, SLC15A2, WDR5B*
1	67682813	67707380	24568	5	*CCDC58*
1	69169609	70158055	988447	5	*CCDC14, ITGB5, KALRN, LOC101905265, ROPN1, UMPS*
1	70158055	70508133	350079	5	*HEG1, ITGB5, MUC13, SLC12A8*
1	74798762	76057452	1258691	5	*ATP13A4, ATP13A5, FGF12, HRASLS, LOC104970906, MB21D2*
1	99785149	100365175	580027	2	*LOC100139843, LOC107131228, MIR551B*
1	110867173	112061776	1194604	2	*CCNL1, LEKR1, PTX3, TRNAW-CCA, VEPH1*
1	112061776	112545103	483328	4	*KCNAB1, LEKR1, LOC101902535, SSR3, TIPARP, TRNAE-UUC, TRNAQ-CUG*
1	119415091	119543574	128484	6	*LOC101905340*
1	120917814	121561995	644182	2	*LOC104968807, LOC781192*
1	121561995	122951858	1389864	3	*LOC100139345, LOC100295922, LOC101905819, LOC101906885, LOC786256, TRNAC-ACA, ZIC1, ZIC4*
1	122951858	123537204	585347	2	*LOC104971015, LOC107132207, LOC782279, PLSCR1, PLSCR5*
1	141137637	141745751	608115	3	*BRWD1, HMGN1, LCA5L, LOC534913, PSMG1, SH3BGR, WRB*
1	144643604	145122423	478820	3	*LOC101905728, PDE9A, RSPH1, SLC37A1, TFF1, TFF2, TMPRSS3, UBASH3A*
2	6762076	7067052	304977	2	*LOC100848294, LOC101905730, LOC101906294, WDR75*
2	7067052	7985124	918073	5	*COL3A1, COL5A2, GULP1, LOC100140504, LOC104971110, MIR2917*
2	9607147	9679430	72284	3	*FAM171B, ITGAV*
2	9679430	9938129	258700	7	*ITGAV, LOC104971113, LOC107132235, MIR2351, TRNAG-UCC, ZC3H15*
2	9938129	9977653	39525	6	.
2	9977653	10167704	190052	5	.
2	10167704	10187315	19612	5	.
2	10187315	10318606	131292	5	.
2	10318606	10768140	449535	3	*FSIP2, LOC100139132*
2	10768140	11052136	283997	3	.
2	11052136	11244889	192754	3	*LOC100138706*
2	12587933	12955788	367856	5	*LOC101907972*
2	12955788	13139828	184041	5	*LOC100848878, LOC787311*
2	13139828	13159357	19530	5	.
2	13159357	14480109	1320753	8	*DNAJC10, DUSP19, FRZB, LOC101905179, LOC107132237, NCKAP1, NUP35, PDE1A, TRNAC-GCA*
2	14480109	14497141	17033	4	*PDE1A*
2	14497141	14515364	18224	3	*PDE1A*
2	14515364	14985968	470605	3	*LOC101907472, LOC104971118, PDE1A, PPP1R1C, SSFA2*
2	19563640	19578540	14901	2	.

2	19578540	19654032	75493	4	*AGPS*
2	28742126	29511210	769085	2	*B3GALT1, MIR2285W, XIRP2*
2	29619238	30921250	1302013	4	*CSRNP3, GALNT3, LOC100141080, LOC104971177, LOC104971179, LOC104971180, LOC107132249, LOC107132250, LOC780963, LOC787863, SCN1A, SCN7A, SCN9A, TTC21B*
2	30921250	33343627	2422378	2	*COBLL1, FIGN, GRB14, LOC101904870, LOC104971181, LOC104971229, LOC107131350, LOC107132251, LOC534223, SCN2A, SLC38A11, TRNAC-GCA*
2	33343627	33966988	623362	2	*KCNH7*
2	35743324	36177070	433747	3	*LOC101906588, RBMS1*
2	36177070	37728548	1551479	3	*BAZ2B, CD302, DAPL1, ITGB6, LOC101906513, LOC101906908, LOC107132253, LOC781943, LY75, MARCH7, PLA2R1, RBMS1, TANC1, WDSUB1*
2	37728548	38516481	787934	5	*CCDC148, DAPL1, LOC107132254, PKP4, TRNAC-GCA, UPP2*
2	38516481	40924608	2408128	2	*ACVR1, ACVR1C, CYTIP, ERMN, GALNT5, GPD2, LOC101907729, LOC101907802, NR4A2, TRNAA-CGC, TRNAC-ACA, TRNAC-GCA, TRNAG-GCC*
2	54779176	54791168	11993	2	*LOC101908745*
2	54791168	54822604	31437	2	*LOC104971226, LOC107132257*
2	54822604	56108710	1286107	3	*LOC101904447, LOC107132258, LOC107132259*
2	56108710	56688313	579604	3	*LOC101908548, TRNAC-GCA*
2	56688313	57570924	882612	5	*LOC107131283, LOC782142, LRP1B*
2	65232852	66493943	1261092	2	*ACTR3, GPR39, LOC100847374, LOC101903503, LYPD1, NCKAP5, SLC35F5*
2	66493943	67907416	1413474	2	*DPP10, LOC101904117, LOC101904159, LOC107132266, LOC781274, MIR2887-1, MIR2904-1*
2	67907416	68227197	319782	8	*DPP10, LOC101904919*
2	68227197	68549782	322586	9	*DPP10*
2	68549782	68641750	91969	7	*LOC101904311*
2	68641750	68689023	47274	7	.
2	68689023	69142467	453445	4	.
2	69142467	69669201	526735	4	*LOC100138369*
2	69669201	69915322	246122	7	.
2	69915322	70099281	183960	8	*LOC107131415*
2	70099281	70434413	335133	9	*CCDC93, DDX18, INSIG2, LOC101904652, LOC107131415, LOC107131416, LOC523484*
2	70434413	71966346	1531934	6	*C1QL2, C2H2orf76, DBI, EN1, LOC100138575, LOC100848049, LOC101907751, LOC104971253, LOC104971255, LOC107131426, LOC616930, MARCO, SCTR, STEAP3, TMEM37*
2	71966346	72274962	308617	5	*CFAP221, PTPN4, TMEM177, TRNAC-GCA, TRNAE-UUC*
2	72274962	72659227	384266	8	*EPB41L5, LOC100139473, LOC101907871, LOC107131428, PTPN4, RALB, TMEM185B, TRNAG-CCC*
2	72659227	74758659	2099433	2	*CLASP1, GLI2, INHBB, LOC100139262, LOC100295231, LOC100335292, LOC100848336, LOC104971258, LOC104971259, LOC107131430, LOC781979, NIFK, RALB, TFCP2L1, TRNAE-UUC, TRNAL-CAA, TSN*
2	78541873	78832515	290643	2	.
2	82477565	82877505	399941	2	.
2	83494136	83500681	6546	2	.
2	83500681	83527672	26992	3	.
2	83527672	84360784	833113	2	*LOC100138726, LOC107131505*
2	86989029	87816781	827753	3	*BOLL, PLCL1*
2	87816781	88707135	890355	2	*SATB2*
2	91915109	92392572	477464	3	*ABI2, CARF, CYP20A1, ICA1L, LOC783376, NBEAL1, RAPH1, WDR12*
2	92392572	92729033	336462	2	*CD28, RAPH1*
2	92729033	93452842	723810	3	*CTLA4, ICOS, LOC101903301*
2	99600902	99869785	268884	2	*LOC101906862*
2	100357429	100446226	88798	5	*ERBB4*
2	100446226	100516918	70693	5	*ERBB4*
2	110475223	111184193	708971	2	*EPHA4, LOC104971315*
2	111184193	112193029	1008837	4	*ACSL3, FARSB, LOC104969778, LOC104971316, LOC107131711, LOC538702, MIR2284Y-5, MOGAT1, PAX3, SGPP2*
2	122369190	122572910	203721	3	*BSDC1, CCDC28B, DCDC2B, EIF3I, FAM167B, FAM229A, HDAC1, IQCC, LCK, LOC789869, MARCKSL1, MTMR9, TMEM234, TSSK3*
3	115	1047887	1047773	4	*ADCY10, DCAF6, GPR161, LOC100335782, LOC100336903, LOC101904335, LOC101907782, LOC104971400, LOC104971401, LOC104971402, LOC107131930, LOC107131946, LOC787328, LOC787668, LOC790004, MPC2, MPZL1, SFT2D2, TBX19, TIPRL*
3	1047887	1862160	814274	4	*CD247, CREG1, DUSP27, GPA33, LOC101907188, LOC104971405, LOC104971407, LOC104971408, POU2F1, RCSD1, TRNAP-CGG*
3	1862160	2256913	394754	5	*ILDR2, LOC789898, MAEL, POGK, TADA1*
3	2256913	2359972	103060	3	*LOC789898*
3	2359972	2539716	179745	4	*LOC100140223*
3	3581552	3794829	213278	2	*LMX1A, RXRG*
3	8155356	8600060	444705	2	*ADAMTS4, APOA2, ARHGAP30, B4GALT3, CFAP126, DEDD, F11R, FCER1G, ITLN2, KLHDC9, LOC104971424, LOC107132053, LOC107132054, LOC107132055, LOC107132057, MPZ, NDUFS2, NIT1, NR1I3, PCP4L1, PFDN2, PPOX, PVRL4, SDHC, TOMM40L, TRNAD-GUC, TRNAE-CUC, TRNAG-GCC, TRNAG-UCC, TRNAL-CAG, TRNAV-CAC, TSTD1, UFC1, USF1, USP21*
3	8600060	8675999	75940	5	*LOC100137986, LOC788783*
3	8675999	8714093	38095	7	*LOC100336682, LOC786046*
3	8714093	8733485	19393	8	.
3	8733485	8810383	76899	7	*ITLN2, LOC100138220, LOC613299, LOC790169*
3	8810383	10201579	1391197	7	*ATP1A2, ATP1A4, CASQ1, CD244, CD48, CD84, CFAP45, COPA, CRP, DCAF8, DUSP23, FCRL6, IGSF8, IGSF9, KCNJ10, KCNJ9, LOC100138271, LOC100139781, LOC101902818, LOC101902959, LOC101903058, LOC101903248, LOC104971425, LOC107132062, LOC613822, LOC787875, LOC788736, LOC790098, LY9, MIR1584, NCSTN, NHLH1, PEA15, PEX19, PIFO, PIGM, SLAMF1, SLAMF6, SLAMF7, SLAMF8, SLAMF9, TAGLN2, VANGL2, VSIG8*
3	10394411	11046761	652351	2	*ACKR1, AIM2, CADM3, FCER1A, IFI16, LOC100848774, LOC101903951, LOC538552, LOC616952, LOC790111, MPTX, OR10J1, OR10J3, TRNAC-ACA, TRNAR-UCU*

3	13129321	13410275	280955	5	*LOC101904923, LOC101905561, LOC107132121, LOC107132273, LOC783963, LOC784007*
3	13410275	13477335	67061	8	.
3	13477335	13514898	37564	8	.
3	13514898	13704429	189532	7	*LOC101906176, LOC101906408*
3	13704429	14053110	348682	4	*ETV3, ETV3L, LOC104971436, LOC107131245, LOC787257, TRNAS-GGA*
3	14053110	15536710	1483601	7	*APOA1BP, ARHGEF2, ASH1L, BCAN, BGLAP, CCT3, CRABP2, DAP3, FDPS, GLMP, GON4L, GPATCH4, HAPLN2, HDGF, INSRR, IQGAP3, ISG20L2, KIAA0907, LAMTOR2, LMNA, LOC100848553, LOC100848574, LOC100848721, LOC101906206, LOC101906276, LOC104971464, LOC104971465, LOC511220, LOC784055, LOC784466, LOC786728, LRRC71, MEF2D, MEX3A, MIR6534, MRPL24, MSTO1, NES, NTRK1, PAQR6, PEAR1, PMF1, PRCC, RAB25, RHBG, RIT1, RRNAD1, RUSC1, RUSC1-AS1, RXFP4, SEMA4A, SH2D2A, SLC25A44, SMG5, SSR2, SYT11, TMEM79, TSACC, TTC24, UBOLN4*
3	21113171	21271572	158402	3	*LOC101907327, MGC134040, TRNAA-CGC, TRNAE-UUC, TRNAG-CCC, TRNAH-GUG, TRNAN-GUU, TRNAQ-CUG, TRNAV-AAC, TRNAV-CAC, TRNAV-UAC*
3	21271572	21367397	95826	2	*LOC614643, TRNAE-UUC, TRNAG-CCC, TRNAH-GUG, TRNAN-GUU, TRNAQ-CUG, TRNAV-AAC, TRNAV-CAC, TRNAV-UAC*
3	21367397	21616259	248863	3	*LOC107132278, LOC107132279, LOC107132280, TRNAE-UUC, TRNAG-CCC, TRNAG-UCC, TRNAH-GUG, TRNAK-CUU, TRNAN-GUU, TRNAQ-CUG, TRNAV-AAC, TRNAV-CAC, TRNAV-UAC*
3	23095483	24935203	1839721	4	*HAO2, HMGCS2, HSD3B1, LOC101903694, LOC104971535, LOC107132287, LOC508547, LOC531521, LOC789317, LOC789334, NOTCH2, PHGDH, REG4, SEC22B, TBX15, WARS2, ZNF697*
3	24935203	24990983	55781	5	.
3	24990983	25232148	241166	6	*LOC100141072, LOC100847756, LOC101903862*
3	25232148	25419353	187206	5	*SPAG17*
3	28396266	28480007	83742	3	.
3	28480007	28534786	54780	3	.
3	28534786	28687338	152553	2	*SYCP1, TSHB, TSPAN2*
3	38027343	38607628	580286	2	*LOC104971607, LOC104971608*
3	47407060	47559336	152277	2	*LOC781902*
3	54365913	54627815	261903	3	*GBP2, GBP5, GBP6, LOC107131333, LOC512486, LOC786389*
3	54627815	54737143	109329	3	*GBP2, LOC781596, LOC785445*
3	54737143	55017267	280125	7	*GBP4, GBP6, LOC104968497, LOC781675, LOC781710*
3	55017267	55107873	90607	2	*LOC100336669, LOC510382*
3	56808531	57707009	898479	5	*HS2ST1, LMO4, LOC101903905, LOC101904240, LOC104969806, LOC104969808, LOC107132316, LOC784606, SEP15, SH3GLB1*
3	60024749	61299768	1275020	2	*DNASE2B, GNG5, LOC100139065, LOC100300942, LOC104971676, PRKACB, RPF1, SAMD13, SPATA1, TTLL7, UOX*
3	72663221	73328200	664980	2	*LOC785254*
3	76688753	76949740	260988	3	.
3	76949740	77564498	614759	3	*LOC101907544, LOC107132322, LOC107132325, LOC527397, RPE65*
3	77564498	78430488	865991	2	*DIRAS3, GADD45A, GNG12, LOC101907753, LOC104971707, TRNAD-GUC, WLS*
3	85943842	87060147	1116306	2	*C3H1orf87, CYP2J2, HOOK1, LOC100139588, LOC104971726, LOC107132327, LOC508252, LOC511936, LOC521656, LOC530875, LOC530929*
3	94149880	97321955	3172076	4	*AGBL4, BTF3L4, C3H1orf185, CC2D1B, CDKN2C, DMRTA2, ECHDC2, ELAVL4, EPS15, FAF1, FAM159A, GPX7, KTI12, LOC101905708, LOC101906012, LOC101906178, LOC101906381, LOC101906433, LOC104971767, LOC104971768, LOC104971769, LOC107132335, LOC107132348, LOC520518, LOC786738, MIR761, NRDC, ORC1, OSBPL9, PRPF38A, RAB3B, RNF11, SCP2, TTC39A, TXNDC12, ZCCHC11, ZFYVE9, ZYG11A, ZYG11B*
3	104221017	104966185	745169	2	*C3H1orf50, CCDC30, CLDN19, ERMAP, FOXJ3, LOC101906941, LOC101907688, LOC531467, LOC538699, LOC614376, LOC788834, P3H1, PPCS, PPIH, RIMKLA, SLC2A1, SVBP, YBX1, ZMYND12, ZNF691*
3	104966185	105141892	175708	2	*FOXJ3, GUCA2A, GUCA2B, HIVEP3*
3	116852818	116963265	110448	5	*IQCA1, TRNAG-CCC*
4	5784478	5806768	22291	3	*ZPBP*
4	5806768	6429468	622701	4	*LOC101904266, VWC2, ZPBP*
4	6429468	7234678	805211	3	*ABCA13, LOC104970217, LOC107132367, LOC781773*
4	7234678	8278950	1044273	5	*C4H7orf57, CDK14, CLDN12, LOC100847712, LOC101910184, LOC107131279, LOC107132370, LOC107132371, LOC107132372, LOC107132373, LOC107132374, LOC107132375, SUN3, UPP1*
4	8278950	8606252	327303	2	*CDK14, FZD1, LOC104971924*
4	14947273	15471274	524002	4	*ASNS, C1GALT1, LOC104971948, LOC107131229, TAC1*
4	29097312	29937284	839973	2	*ABCB5, ITGB8, LOC100138363, LOC104971982, SP8*
4	64264349	65194119	929771	2	*AVL9, BBS9, FKBP9, KBTBD2, LOC104972083, LOC107132408, LOC107132409, LOC782987, LOC785232, LOC786199, LSM5, NT5C3A, PDE1C, RP9*
4	65791977	66781544	989568	2	*ADCYAP1R1, AQP1, CRHR2, FAM188B, GARS, GGCT, GHRHR, INMT, LOC101904290, LOC104976909, LOC107132410, MIR2419, NOD1, ZNRF2*
4	68451810	68611162	159353	2	*LOC104972107*
4	71826303	72190768	364466	2	*DFNA5, LOC107132418, MPP6*
4	83520047	83522826	2780	3	.
4	83522826	83552912	30087	6	*LOC107132425*
4	83552912	83687255	134344	6	*LOC100295719, LOC100335205, LOC101907022, LOC101907237, LOC104976912, LOC104976913, LOC107132422*
4	83687255	83852992	165738	6	*LOC107132423, LSM8, TRGC3, TRGC4*
4	83852992	84031826	178835	4	.
4	84031826	84070396	38571	3	.
4	84070396	84411475	341080	6	*LOC107132427*
4	84411475	84677865	266391	4	.
4	84677865	84847336	169472	2	.
4	84847336	84858487	11152	2	.
4	84858487	85178117	319631	2	*LOC100139485, LOC512673, LOC786533*

4	87411115	89248836	1837722	2	*AASS, ASB15, CADPS2, FEZF1, IQUB, LMOD2, LOC100294792, LOC100847585, LOC104972175, LOC104972176, LOC518161, NDUFA5, PTPRZ1, RNF133, RNF148, SLC13A1, TAS2R16, WASL*
4	89248836	89429384	180549	3	*LOC107132424, LOC107132426, LOC509761, LOC518161, LOC527125, SPAM1*
4	89429384	89643758	214375	5	*LOC509761, LOC782002, TMEM229A*
4	90623361	90767040	143680	2	.
4	90767040	90947479	180440	2	*LOC782604*
4	92806991	93209240	402250	3	*ARF5, FSCN3, GCC1, LOC107132428, PAX4, SND1*
4	95429067	96066329	637263	2	*COPG2, KLF14, LOC100138131, LOC100848808, LOC100848846, LOC101904500, LOC104972200, LOC107131356, LOC107132430, LOC107132431, MIR29A, MIR29B-1, TSGA13*
4	96066329	96280199	213871	2	*LOC104972201, MKLN1, TRNAE-UUC*
4	99548941	99964093	415153	2	*AGBL3, BPGM, CALD1*
4	101309337	101497365	188029	3	*LOC786173*
4	101497365	102081800	584436	2	*CHRM2, MIR490, PTN, TRNAC-GCA*
4	103222137	103301162	79026	2	.
4	105790687	106388431	597745	2	*AGK, KIAA1147, LOC104972259, LOC615237, LOC785954, LOC787707, OR9A4, PRSS37, SSBP1, TAS2R3, TAS2R4, TAS2R5, TMEM178B, TRNAE-CUC, WEE2*
4	107799457	108367650	568194	2	*CASP2, CLCN1, EPHA1, FAM131B, GSTK1, LOC100299556, LOC101905088, LOC101905883, LOC506549, LOC524903, LOC532208, LOC613799, LOC785618, LOC787500, TAS2R40, TAS2R41, TAS2R60, TCAF1, TCAF2, TMEM139, TRNAC-ACA, 7YX*
4	108636755	108782393	145639	2	*LOC100141283, LOC507423, LOC508101, LOC511400, LOC514662, LOC519916, LOC787786*
4	109841023	109914270	73248	2	.
4	109914270	110112590	198321	2	.
4	113569404	113782191	212788	2	*KRBA1, LOC104972285, TRNAC-GCA, ZNF746, ZNF777*
4	115049944	116584543	1534600	5	*ACTR3B, CCT8L2, CRYGN, GALNT11, GALNTL5, KMT2C, LOC100847784, LOC104972293, LOC107132443, LOC504219, LOC789764, NUB1, RHEB, WDR86, XRCC2*
4	116584543	116593066	8524	7	.
4	116593066	116882680	289615	7	*LOC509941, TRNAC-GCA, TRNAG-CCC*
4	116882680	116926358	43679	8	.
4	116926358	116978379	52022	7	.
4	116978379	117034233	55855	6	.
4	117034233	117288397	254165	3	.
5	439925	564943	125019	3	*LOC100300697, LOC107132450, LOC782348, MIR2284Y-6*
5	564943	586883	21941	4	.
5	586883	597090	10208	5	.
5	597090	623898	26809	5	.
5	623898	634013	10116	3	.
5	634013	724185	90173	2	.
5	724185	893922	169738	2	*TRNAC-GCA, TSPAN8*
5	893922	1028087	134166	3	.
5	1028087	1100189	72103	3	*LGR5*
5	1100189	1777877	677689	4	*LGR5, LOC104972316, LOC104972318, LOC107131380, LOC107132451, RAB21, TBC1D15, THAP2, TMEM19, TPH2, ZFC3H1*
5	1914509	2186305	271797	2	*TRHDE*
5	2186305	2203081	16777	2	*TRHDE*
5	2203081	2379233	176153	5	*TRHDE*
5	2379233	2411220	31988	5	.
5	2411220	2423814	12595	4	.
5	2423814	2474456	50643	4	.
5	2474456	2483082	8627	3	.
5	2632324	2646959	14636	3	.
5	2646959	2692819	45861	4	.
5	2692819	2874861	182043	3	.
5	3620248	3856426	236179	2	.
5	3856426	3998534	142109	3	.
5	3998534	4008099	9566	2	.
5	4008099	4114435	106337	2	*ATXN7L3B*
5	4114435	4165563	51129	4	*LOC523590*
5	4165563	4239082	73520	5	.
5	4239082	4251199	12118	5	.
5	4251199	4310635	59437	4	.
5	4310635	4399763	89129	6	.
5	4399763	4527920	128158	3	*KCNC2, TRNAY-GUA*
5	4527920	4611238	83319	3	*KCNC2*
5	4611238	4701459	90222	7	*KCNC2*
5	4701459	4805869	104411	7	*CAPS2*
5	4805869	4825374	19506	7	*CAPS2, GLIPR1L1*
5	4825374	4859596	34223	7	*GLIPR1L1*
5	4859596	4891877	32282	6	*GLIPR1L1, GLIPR1L2*
5	4891877	4939903	48027	5	*GLIPR1L2*
5	4939903	5137431	197529	4	*GLIPR1, KRR1, LOC107132452*
5	5137431	5229672	92242	6	*LOC104972322*
5	5229672	5292853	63182	7	.
5	5292853	5411798	118946	4	*LOC785400*
5	5411798	5630469	218672	2	*NAP1L1, PHLDA1*
5	6311101	6330739	19639	2	*LOC104972329*
5	6330739	6420487	89749	2	*LOC104972328, LOC104972329*
5	6693208	6904829	211622	2	.
5	6904829	7138623	233795	2	.
5	7196789	7242823	46035	2	.
5	7270196	7319421	49226	2	*NAV3*
5	7319421	7512172	192752	3	*NAV3*
5	7512172	7711444	199273	3	*NAV3*

5	7711444	7838466	127023	2	*LOC104972331*
5	7838466	7985794	147329	2	.
5	8012345	8017098	4754	2	.
5	8017098	8088122	71025	3	.
5	8088122	8349091	260970	9	*LOC104972332*
5	8349091	8545718	196628	14	*LOC101907549, SYT1*
5	8545718	8736391	190674	16	*SYT1*
5	8736391	8826461	90071	22	*LOC104972336, SYT1*
5	8826461	8894814	68354	17	*LOC104972336, LOC107131353, SYT1*
5	8894814	9100879	206066	12	*LOC104972334, LOC104972335, SYT1*
5	9100879	9290345	189467	12	*PAWR*
5	9290345	9444803	154459	9	*LOC104972337, PPP1R12A*
5	9444803	9658212	213410	8	*PPP1R12A*
5	9658212	9690965	32754	6	.
5	9690965	9982724	291760	2	*OTOGL*
5	10010817	10132980	122164	2	*PTPRQ*
5	10132980	10296641	163662	3	*PTPRQ*
5	10296641	10409532	112892	4	*MYF5, MYF6, PTPRQ*
5	10409532	10551759	142228	3	*LIN7A*
5	10551759	10729620	177862	4	*LIN7A*
5	13831494	13886098	54605	2	.
5	13886098	13947817	61720	2	.
5	13947817	14000549	52733	2	.
5	14000549	14177952	177404	2	.
5	15155020	15209144	54125	2	.
5	15209144	15522159	313016	3	.
5	15522159	15583971	61813	4	*RASSF9*
5	15583971	15665847	81877	5	*LOC107132461, NTS, RASSF9*
5	15805773	15830618	24846	5	*MGAT4C*
5	15830618	15916364	85747	5	*MGAT4C*
5	15916364	15941849	25486	4	.
5	16279758	16528289	248532	5	.
5	16528289	16704527	176239	8	.
5	16704527	16801918	97392	7	.
5	16801918	16804356	2439	6	.
5	16804356	16870500	66145	7	*LOC781871*
5	16870500	16905216	34717	6	.
5	16905216	16930517	25302	9	.
5	16930517	16952638	22122	8	.
5	16952638	17002132	49495	7	.
5	17002132	17065149	63018	7	.
5	17065149	17128261	63113	3	.
5	17618052	17808403	190352	2	.
5	17808403	17846180	37778	2	.
5	17846180	18173970	327791	2	*C5H12orf29, C5H12orf50, CEP290, TMTC3*
5	18300447	18500724	200278	3	*KITLG*
5	18500724	18653953	153230	3	*LOC107132455, TRNAG-CCC*
5	18653953	18666885	12933	4	.
5	18666885	18783346	116462	3	*LOC104972350*
5	18783346	18927558	144213	3	.
5	18927558	19018920	91363	2	*LOC100138578, LOC104972370*
5	19123771	19571124	447354	2	*DUSP6, GALNT4, LOC101906850, LOC101906971, LOC104972351, LOC107131351, LOC107132457, LOC781851, POC1B, TRNAK-UUU*
5	19571124	19717683	146560	3	*ATP2B1*
5	19717683	19784656	66974	2	.
5	19784656	19882814	98159	3	.
5	19882814	20099437	216624	2	.
5	20099437	20213170	113734	3	.
5	20213170	20343409	130240	2	.
5	20571197	20611925	40729	2	*LOC100848024*
5	20611925	20768416	156492	2	.
5	20768416	20890950	122535	6	.
5	20890950	21037819	146870	5	*CCER1, EPYC*
5	21037819	21196770	158952	2	*DCN, KERA, LUM, TRNAC-ACA*
5	22021953	22294610	272658	3	*BTG1, LOC104972363, LOC107132458, LOC107132459*
5	22294610	22463653	169044	3	*LOC107132463*
5	22463653	22642083	178431	2	*LOC107132460, LOC784883*
5	22642083	22674044	31962	2	.
5	22674044	22889738	215695	2	*EEA1, LOC104972371, LOC107132468, LOC519417*
5	22889738	23348216	458479	2	*EEA1, LOC107132464, LOC107132465, LOC107132466, LOC107132467*
5	23348216	23631496	283281	6	*LOC101905513, LOC107132465, MRPL42, NUDT4, SOCS2, UBE2N*
5	23631496	24040418	408923	2	*CRADD, LOC101905585, LOC104972373, LOC104972374, LOC104972375*
5	25608175	26157128	548954	2	*CBX5, COPZ1, GLYCAM1, GPR84, GTSF1, HNRNPA1, ITGA5, LOC100847613, LOC101906546, LOC101907193, LOC534520, MIR148B, NCKAP1L, NFE2, PDE1B, PPP1R1A, SMUG1, ZNF385A*
5	26157128	26341839	184712	3	*HOXC10, HOXC11, HOXC12, HOXC13, HOXC4, HOXC5, HOXC6, HOXC8, HOXC9, LOC100847613, LOC101906996, LOC101907194, LOC101907241, LOC107132469, LOC107132470, LOC785891, MIR196A-2, MIR615*
5	29840141	29914762	74622	2	*LIMA1*
5	31082135	31405283	323149	2	*ADCY6, ARF3, CACNB3, CCDC65, CCNT1, DDX23, FKBP11, KANSL2, LOC100335701, LOC107132475, LOC783895, MIR1291, RND1*
5	31405283	31435844	30562	5	*LALBA, LOC104972406*
5	31435844	31465094	29251	4	*LOC617415*
5	31465094	31484287	19194	4	*LOC526630*
5	31484287	31528809	44523	3	*LOC614370*
5	31528809	31582471	53663	2	*LOC515887*
5	31742466	31812280	69815	2	*C5H12orf54, LOC781840*

5	31812280	31904284	92005	2	*LOC509817*
5	31904284	32113679	209396	2	*LOC100300010, LOC518129, LOC523258, LOC617380, LOC617388, LOC781968*
5	32113679	32186128	72450	3	*LOC100848234, ZNF641*
5	32186128	32199156	13029	3	*H1FNT, LOC784038*
5	32199156	32226441	27286	4	.
5	32226441	32522177	295737	4	*ASB8, CCDC184, COL2A1, LOC100139168, LOC101902265, LOC101905376, LOC104972407, LOC513452, LOC619137, OR10AD1, PFKM, SENP1*
5	32522177	32599220	77044	2	*COL2A1, TMEM106C*
5	33811792	33938204	126413	3	.
5	33938204	34169187	230984	2	*LOC107132478, SLC38A2*
5	34169187	34239479	70293	2	*SLC38A1*
5	34239479	34306959	67481	2	*SLC38A1*
5	34462922	34562320	99399	3	*ARID2, SCAF11*
5	34562320	34610038	47719	4	*ARID2, TRNAC-ACA*
5	34610038	34617829	7792	4	*ARID2*
5	34617829	34769744	151916	4	*ARID2*
5	34769744	34888938	119195	5	*ARID2*
5	34888938	35227840	338903	3	*ANO6*
5	35978920	36197204	218285	2	*LOC107132476, NELL2*
5	36197204	36232019	34816	2	*LOC107132476*
5	36232019	36316484	84466	2	*TMEM117*
5	36316484	36337947	21464	2	*TMEM117*
5	36337947	36406600	68654	3	*TMEM117*
5	36406600	36443954	37355	3	.
5	36443954	36542742	98789	2	*LOC107131297*
5	37025559	37236600	211042	4	*ADAMTS20*
5	37236600	37255967	19368	5	*ADAMTS20*
5	37255967	37392896	136930	3	*ADAMTS20*
5	37392896	37489647	96752	3	*ADAMTS20, LOC101904720, LOC534627*
5	37489647	37538959	49313	3	*LOC786089*
5	37538959	37777002	238044	3	*LOC786089*
5	37777002	37945765	168764	4	*TRNAS-GGA*
5	37945765	38114469	168705	3	.
5	38314607	38614126	299520	3	*LOC100336326, PPHLN1, PRICKLE1*
5	38614126	38864463	250338	4	*GXYLT1, PPHLN1, YAF2, ZCRB1*
5	39528650	39659992	131343	2	*LOC107131267*
5	39659992	39731473	71482	2	*LOC101905399, LOC107131267, TRNAQ-CUG*
5	39731473	39819256	87784	2	*LOC107131267*
5	39819256	40012213	192958	2	*LOC100139418, LOC107131267*
5	40012213	40027223	15011	2	.
5	40027223	40333876	306654	2	*CNTN1*
5	40523390	40545395	22006	7	.
5	40545395	40593945	48551	6	.
5	40593945	40673770	79826	7	*MUC19*
5	40673770	40694473	20704	7	*MUC19*
5	40694473	40729225	34753	5	*MUC19*
5	40729225	40830261	101037	2	*LOC534391, LRRK2, MUC19*
5	40918915	41278624	359710	3	*LRRK2, SLC2A13*
5	41278624	41384642	106019	5	*SLC2A13*
5	41384642	41611222	226581	2	*SLC2A13*
5	41611222	41673692	62471	2	*SLC2A13*
5	41673692	41851800	178109	3	*ABCD2, C5H12orf40*
5	41851800	42020746	168947	3	*LOC785955*
5	42020746	42032185	11440	3	*LOC100847470*
5	42032185	42256395	224211	2	*KIF21A, MIR2428*
5	42457477	42571775	114299	5	*CPNE8, LOC101907676*
5	42571775	42767965	196191	11	*CPNE8*
5	42767965	42792545	24581	13	*CPNE8*
5	42792545	42820516	27972	12	*CPNE8*
5	42820516	42861062	40547	12	.
5	42861062	42997132	136071	8	*PTPRR*
5	42997132	43013887	16756	10	*PTPRR*
5	43013887	43051636	37750	10	*PTPRR*
5	43051636	43066541	14906	10	*PTPRR*
5	43066541	43305508	238968	7	*PTPRB, PTPRR*
5	43305508	43406678	101171	6	*KCNMB4, MIR2284Z-5, PTPRB, TRNAC-GCA*
5	43406678	43453830	47153	9	*KCNMB4, LOC100138492*
5	43453830	43612144	158315	10	*CNOT2, KCNMB4, LOC107132479*
5	43612144	43718585	106442	8	*LOC104972423, LOC107131684*
5	43718585	43825499	106915	6	.
5	43825499	43971017	145519	3	*MYRFL*
5	43971017	44051445	80429	3	*MYRFL, RAB3IP*
5	44051445	44165151	113707	4	*BEST3, LOC107132482, RAB3IP*
5	44165151	44426004	260854	5	*BEST3, CCT2, FRS2, LOC100138933, LOC107132483, LRRC10, MIR2427*
5	44840196	44884875	44680	4	*LYZ*
5	44884875	44977579	92705	4	*CPSF6*
5	44977579	45069213	91635	3	*LOC107132485*
5	45069213	45274321	205109	3	*CPM, LOC104969823*
5	45274321	45577631	303311	3	*CPM, LOC104972426, LOC104972427, LOC107132480, MDM2, NUP107, RAP1B, SLC35E3*
5	45594832	45608608	13777	2	.
5	45608608	45718274	109667	3	*LOC104972428*
5	46328167	46361300	33134	2	.
5	46361300	46506680	145381	3	*DYRK2*
5	46702779	46763955	61177	2	.
5	46784332	47368052	583721	2	*CAND1, LOC100337478, LOC100848339, LOC107132481, TRNAE-UUC*
5	47368052	47678820	310769	2	*LOC100337478, TRNAC-GCA*

5	47782922	48188745	405824	3	*HELB, IRAK3, LLPH, LOC107132486, LOC525592, LOC783726, TMBIM4, TRNAK-CUU*
5	48188745	48243170	54426	2	*HMGA2, LOC100848387*
5	48508578	48647839	139262	2	*LOC101905641, LOC101905683*
5	48647839	48673623	25785	2	.
5	48673623	48916848	243226	9	*MSRB3*
5	48916848	49146564	229717	8	*LEMD3, MIR2429, WIF1*
5	49146564	49311967	165404	8	*LOC107132487, WIF1*
5	49311967	49517029	205063	5	*GNS, LOC101906182, TBC1D30*
5	49517029	50067479	550451	2	*C5H12orf56, C5H12orf66, LOC101906522, LOC107132488, MIR2285F-1, RASSF3, SRGAP1, TBK1, TRNAF-AAA, XPOT*
5	50102686	50162577	59892	6	*SRGAP1*
5	50162577	50314159	151583	5	*LOC107132489, SRGAP1*
5	50314159	50434366	120208	2	*TMEM5*
5	50561941	50635650	73710	3	.
5	50635650	50697994	62345	3	.
5	50697994	50725587	27594	3	.
5	50725587	50778363	52777	3	*AVPR1A*
5	50778363	50875417	97055	4	.
5	50875417	50949612	74196	5	*LOC100140195*
5	50949612	50957299	7688	6	.
5	50957299	51252430	295132	5	*PPM1H*
5	51252430	51519839	267410	6	*LOC104972443, LOC784406, MIRLET7I, MON2, PPM1H*
5	51519839	51527022	7184	7	.
5	51527022	51729680	202659	8	*USP15*
5	51729680	51965933	236254	2	*FAM19A2*
5	51965933	52060683	94751	3	*FAM19A2*
5	52060683	52329927	269245	2	*FAM19A2, LOC101903699, LOC104972445*
5	52329927	52573428	243502	2	*FAM19A2*
5	52573428	52584781	11354	3	.
5	52584781	52750281	165501	4	.
5	52750281	52856899	106619	6	.
5	52856899	53022987	166089	8	*LOC617908*
5	53022987	53104152	81166	9	*LOC617908*
5	53104152	53114278	10127	9	.
5	53114278	53226677	112400	8	.
5	53226677	53238343	11667	9	.
5	53238343	53329666	91324	9	.
5	53329666	53410321	80656	8	*LOC788961*
5	53410321	53610267	199947	8	.
5	53610267	53652100	41834	8	.
5	53652100	53696353	44254	7	.
5	53696353	53835646	139294	7	.
5	53835646	53953297	117652	6	.
5	53953297	54236924	283628	6	*SLC16A7*
5	54236924	54261341	24418	6	*SLC16A7*
5	54261341	54638166	376826	6	*LOC101907520, LOC512953, SLC16A7*
5	54638166	54664486	26321	5	.
5	54664486	54717844	53359	4	.
5	54968693	55152212	183520	3	*LRIG3*
5	55152212	55179298	27087	3	.
5	69423137	69605239	182103	2	.
5	78796860	79351588	554729	6	*AMN1, DENND5B, ETFBKMT, FAM60A, LOC101902656, LOC107132510, LOC107132512*
5	79351588	79411652	60065	5	*LOC101903536, LOC107132512*
5	87693343	88486577	793235	2	*C2CD5, ETNK1, LOC100139600, LOC100335949, TRNAG-CCC, TRNAK-UUU*
5	98719095	99679305	960211	3	*BOTA-T2R10B, ETV6, LOC100138383, LOC100140395, LOC100141277, LOC101903336, LOC104972542, LOC104976968, LOC616478, LOC782957, SMIM10L1, STYK1, T2R10C, T2R12, T2R65A, TAS2R10, TAS2R42, TAS2R46, TAS2R7, TAS2R8, TAS2R9, YBX3*
5	99679305	99886865	207561	3	*KLRA1, LOC100140174, LOC101903003, LOC513869, LOC788694, MAGOHB, STYK1*
5	102930963	103110464	179502	3	*LOC100335428, WC1-12*
5	121453798	121497102	43305	2	*MLC1, MOV10L1*
6	24986	307217	282232	3	*APELA, LOC100299844, LOC100299892, LOC101905490*
6	307217	446776	139560	3	*LOC513842*
6	446776	510323	63548	2	.
6	1342122	1528525	186404	2	*LOC101907838, LOC107132536*
6	2079968	2358380	278413	2	*LOC107132537, MARCH1, TKTL2, TMA16*
6	2358380	2683253	324874	2	*LOC107132538, NAF1, NPY1R, NPY5R*
6	4377275	4830964	453690	2	*NDNF, PRDM5*
6	4830964	5004058	173095	3	.
6	17781495	18099168	317674	4	*COL25A1, ETNPPL, OSTC, RPL34*
6	18099168	18413419	314252	4	*LOC104972676, LOC104972677, LOC104972678, LOC104972680*
6	18413419	18886359	472941	2	*CYP2U1, HADH, LEF1, LOC107132549, SGMS2, TRNAC-GCA, TRNAG-CCC*
6	18886359	19199129	312771	3	*LOC104972682, PAPSS1*
6	19199129	19323028	123900	3	*LOC104972682*
6	19323028	19347807	24780	4	*LOC104972682*
6	19347807	19670505	322699	5	*DKK2, LOC104972682*
6	19670505	19813349	142845	7	*DKK2*
6	19813349	19996558	183210	4	*TRNAC-GCA*
6	19996558	20484214	487657	4	*AIMP1, GIMD1, LOC104972685, TBCK*
6	24646787	24849048	202262	6	*TRNAW-CCA*
6	24849048	26040868	1191821	3	*EMCN, LOC104972699, PPP3CA*
6	28172336	28312561	140226	2	*LOC100301359, LOC104972708*
6	28312561	28338758	26198	3	*LOC104972708*
6	28338758	28372077	33320	3	*LOC104972708*
6	28372077	28473009	100933	3	*LOC104972708*

6	28473009	29194773	721765	2	*LOC104972708, LOC104972710, LOC104972713, LOC782904, LOC782977, TRNAC-GCA*
6	29981797	30300325	318529	3	*LOC104972712, PDHA2*
6	30300325	31905934	1605610	3	*BMPR1B, HPGDS, PDLIM5, TRNAK-CUU, UNC5C*
6	31905934	32352232	446299	2	*ATOH1, HPGDS, LOC100140135, SMARCAD1*
6	32352232	32378781	26550	3	.
6	32378781	32830464	451684	3	*LOC107132560, LOC536367, TRNAE-UUC*
6	33342585	33549452	206868	3	*LOC783261*
6	35166605	35408630	242026	2	*CCSER1, LOC509118*
6	35408630	35556058	147429	7	*CCSER1*
6	35556058	35697025	140968	7	*CCSER1*
6	35697025	36219277	522253	6	*CCSER1*
6	36219277	36662626	443350	4	*MMRN1, SNCA*
6	36662626	36946706	284081	5	.
6	41086945	41102846	15902	4	.
6	41102846	41333703	230858	4	.
6	41333703	42137451	803749	4	*KCNIP4, LOC100138737, MIR218-1, PACRGL, SLIT2*
6	43139934	43168885	28952	3	*KCNIP4*
6	43168885	43628920	460036	3	*KCNIP4, LOC104972732, LOC782172*
6	43628920	43927191	298272	2	*ADGRA3, LOC101904854*
6	43927191	44259004	331814	3	*GBA3*
6	44259004	45006656	747653	2	*LOC104972733, LOC107132564*
6	45006656	45268654	261999	6	*PPARGC1A*
6	45268654	45755125	486472	2	.
6	46396270	46532050	135781	4	*SEPSECS*
6	46532050	46804412	272363	6	*ANAPC4, LOC101905518, PI4K2B, ZCCHC4*
6	46804412	46810205	5794	8	.
6	46810205	46862906	52702	8	*LOC104970156*
6	46862906	47259965	397060	3	*LOC104968857, SEL1L3, SLC34A2, SMIM20, TRNAA-UGC*
6	48649938	48724873	74936	2	.
6	48724873	48738248	13376	2	.
6	48738248	48838466	100219	3	.
6	48838466	48956663	118198	3	.
6	48956663	49229815	273153	4	.
6	49229815	49405584	175770	3	.
6	49405584	49663465	257882	2	.
6	49663465	49832730	169266	2	.
6	49832730	50120022	287293	4	*LOC107132565*
6	50120022	50291734	171713	4	.
6	50291734	50674431	382698	2	*LOC100298058, TRNAS-GGA*
6	50674431	50753830	79400	2	.
6	50753830	50909774	155945	2	.
6	50909774	50991725	81952	2	.
6	52334765	52358451	23687	2	.
6	52358451	52467416	108966	2	*LOC784827*
6	52467416	52486573	19158	2	.
6	52486573	52589394	102822	2	.
6	53101390	53245790	144401	2	.
6	53799194	54511182	711989	4	.
6	54511182	54664692	153511	6	*TRNAC-GCA*
6	54664692	54794332	129641	4	.
6	54794332	55182575	388244	3	.
6	56957766	57419866	462101	5	*ARAP2, LOC781670*
6	57419866	57759068	339203	8	*ARAP2, DTHD1*
6	57759068	57803509	44442	11	.
6	57803509	57844452	40944	10	.
6	57844452	57913813	69362	9	*LOC104972734*
6	57913813	57916916	3104	6	*LOC104972734*
6	57916916	58294387	377472	7	*LOC104972734, LOC104972735*
6	63733355	64613770	880416	6	*LOC101906152, TRNAC-ACA*
6	64613770	65124356	510587	13	*KCTD8, LOC100848032*
6	65124356	65495708	371353	10	*GNPDA2, GUF1, LOC104968873, LOC104972745, TRNAC-ACA, YIPF7*
6	65495708	67161799	1666092	9	*GABRA2, GABRG1, LOC100139936, LOC100298320, LOC104972745, LOC104972749, LOC107131339, LOC784591, TRNAW-CCA*
6	67161799	68180823	1019025	16	*ATP10D, COMMD8, COX7B2, GABRA4, GABRB1, LOC781223*
6	68180823	68336991	156169	17	*ATP10D, CORIN, LOC101906472, TRNAC-GCA*
6	68336991	69743581	1406591	10	*CNGA1, CORIN, CWH43, DCUN1D4, FRYL, LOC101905687, LOC104968875, LOC104968876, NFXL1, NIPAL1, OCIAD1, OCIAD2, SLAIN2, SLC10A4, TEC, TRNASTOP-UCA, TXK, ZAR1*
6	69743581	69981769	238189	5	*LOC107132568, LRRC66, SGCB, SPATA18*
6	69981769	72097370	2115602	14	*CHIC2, DANCR, FIP1L1, GSX2, KIT, LNX1, LOC100296505, LOC100296974, LOC100847183, LOC101905946, LOC101906021, LOC101906092, LOC104968884, MIR4449, PDGFRA, RASL11B, SCFD2, USP46*
6	76176091	76432765	256675	5	.
6	76432765	77451977	1019213	4	*LOC100141023*
6	77895593	78296831	401239	3	.
6	87965199	88038537	73339	2	*AMTN*
6	88038537	88404577	366041	2	*AMBN, ENAM, GRSF1, JCHAIN, LOC100848253, LOC101905786, MOB1B, RUFY3, UTP3*
6	88404577	89568233	1163657	3	*ADAMTS3, DCK, GC, LOC782958, NPFFR2, SLC4A4*
6	89568233	90111673	543441	2	*ADAMTS3, TRNAC-GCA*
6	90111673	91324655	1212983	2	*AFM, AFP, ALB, ANKRD17, COX18, CXCL2, CXCL3, CXCL5, CXCL8, GRO1, LOC100140503, LOC100847175, LOC101906736, LOC107132573, LOC107132574, LOC534181, MTHFD2L, PF4, PPBP, RASSF6*
6	94209669	94457428	247760	2	*CXCL13, LOC100137799, LOC100139130*
6	94457428	94519611	62184	4	.
6	94519611	94818224	298614	2	*CNOT6L, LOC107131294, MRPL1*

6	94818224	95400444	582221	3	*LOC101902382, LOC107131184*
6	98750261	99047763	297503	5	.
6	99047763	99106892	59130	3	.
6	99106892	99250485	143594	2	.
6	100969075	101015489	46415	2	.
7	251220	679297	428078	2	*CNOT6, FLT4, LOC100125913, LOC101902704, LOC101902742*
7	24802266	26174090	1371825	2	*ADAMTS19, CHSY3, KIAA1024L, LOC789648, TRNAQ-UUG*
7	26174090	26206792	32703	3	.
7	26206792	26354985	148194	4	*ISOC1, SLC27A6*
7	26354985	27471924	1116940	5	*FBN2, LOC104969125, LOC107132618, SLC12A2, SLC27A6*
7	27471924	27585887	113964	3	*LOC107132618*
7	27692130	27895171	203042	3	*LOC101904034, LOC107132618, PRRC1*
7	27895171	28347916	452746	3	*C7H5orf63, LOC104969128, MEGF10*
7	30151328	31066257	914930	3	*LOC104969139, LOC107132620, LOC789456, ZNF608*
7	35188358	35267381	79024	2	.
7	36674555	37944481	1269927	4	*LOC100138116, LOC104969148*
7	37944481	38268431	323951	3	.
7	38268431	38275876	7446	3	.
7	45014934	45725639	710706	2	*ABCA7, APC2, ARID3A, ATP5D, AZU1, C7H19orf24, C7H19orf25, CBARP, CFD, CIRBP, CNN2, DAZAP1, EFNA2, ELANE, FGF22, FSTL3, GAMT, GPX4, GRIN3B, HCN2, HMHA1, KISS1R, LOC104972838, LOC107131164, LOC107132631, LOC107132632, MED16, MIDN, MISP, MUM1, NDUFS7, PALM, PCSK4, PLPPR3, POLR2E, POLRMT, PRSS57, PRTN3, PTBP1, R3HDM4, REEP6, RNF126, RPS15, SBNO2, STK11, TMEM259, TRNAE-UUC, TRNAF-GAA, TRNAN-GUU, WDR18*
7	45725639	46602147	876509	2	*ABHD17A, ADAT3, AFF4, ATP8B3, BTBD2, CSNK1G2, GDF9, HSPA4, KLF16, LEAP2, LOC104969170, LOC107131164, LOC107132633, LOC107132634, LOC781086, LOC781691, MBD3, MEX3D, MIR6120, PLK5, REXO1, SCAMP4, SHROOM1, SOWAHA, TCF3, UQCR11, UQCRQ, ZCCHC10*
7	55035843	55250130	214288	2	*LOC100298854, LOC107132644, LOC527450, LOC783976, LOC784019, LOC784050, LOC784477, LOC784598, OR4A16*
7	64903971	65069679	165709	6	*BERV-K1, FAT2, SLC36A1*
7	65069679	65405962	336284	11	*ATOX1, G3BP1, GLRA1, LOC104969220, SPARC*
7	65405962	68171229	2765268	2	*FAM114A2, GALNT10, GLRA1, GRIA1, HAND1, LOC100140426, LOC100296765, LOC101903312, LOC104969222, LOC104969225, LOC784755, MFAP3, NMUR2, SAP30L, SMIM15*
7	68171229	68605158	433930	5	*CNOT8, FAXDC2, GEMIN5, LARP1, LOC100335564, LOC101903541, LOC104969230, MRPL22*
7	68605158	69326742	721585	2	*LOC104969230, LOC107132652*
7	69326742	70063537	736796	2	*LOC104969236, SGCD*
7	70334735	70665842	331108	3	*HAVCR1, LOC107132653, SGCD, TIMD4, TRNAG-UCC*
7	70665842	71014173	348332	2	*LOC100294726, LOC101904859, LOC101905139, LOC101905257, LOC101907226, LOC101907274, LOC104969231, TIMD4*
7	71903551	71984425	80875	2	*LOC785534*
7	79283005	79625587	342583	3	.
7	86432350	87056253	623904	2	*EDIL3*
7	93269435	93571127	301693	3	*LOC104968987, LOC507513, LOC789955*
7	93571127	94360798	789672	4	*ARRDC3, LOC101905238, LOC104968990, LOC104972872, LOC107132662*
7	94360798	95003279	642482	11	.
7	99463028	99494204	31177	2	.
7	99494204	99558061	63858	3	.
7	99558061	99625815	67755	3	.
7	99625815	99761008	135194	3	.
7	103152585	103539377	386793	7	*LOC107132668*
7	103539377	103754516	215140	3	*LOC107132668*
7	103754516	103980266	225751	4	.
7	103980266	104442596	462331	2	*LOC100847643, LOC104969005, LOC786268, SLCO4C1, SLCO6A1, TRNAE-UUC*
7	108291934	108966859	674926	5	.
7	108966859	109072654	105796	4	.
7	109072654	109164576	91923	3	.
7	109164576	109182012	17437	3	.
7	109182012	109200950	18939	3	.
7	109200950	109517831	316882	3	*TRNAC-ACA*
7	110116023	110529365	413343	3	*LOC523504*
7	110529365	112131509	1602145	7	*FER, LOC100140403, LOC104969022, LOC104969024, LOC104969026, LOC107132674, LOC523504, MAN2A1, MIR2465, PJA2, RPL19*
7	112131509	112235723	104215	2	*LOC104969027, LOC104969028*
8	966610	1208763	242154	4	*CBR4, LOC101903041, MIR2467, SH3RF1*
8	1208763	2178035	969273	2	*AADAT, C8H4orf27, CLCN3, LOC100848912, LOC101904130, LOC101904351, LOC520638, LOC615521, MFAP3L, NEK1*
8	10468431	12031431	1563001	2	*ACO1, CCDC25, CLU, DDX58, ELP3, ESCO2, LOC100616098, LOC101907891, LOC104969266, LOC104969267, LOC104969268, MIR2469, NDUFB6, NUGGC, PBK, SCARA3, SCARA5, TMEM215, TOPORS, TRNAC-ACA*
8	12031431	12177162	145732	3	.
8	12177162	12531648	354487	4	*LOC100139644, TRNAC-ACA*
8	12531648	13117071	585424	6	*LOC104969269, LOC104969270, LOC104969271*
8	13117071	13130716	13646	2	*LOC107132681*
8	13130716	13169166	38451	2	*MIR2285M-5*
8	14263406	14318457	55052	5	.
8	14318457	14639811	321355	6	.
8	14639811	14982948	343138	2	.
8	14982948	15320997	338050	5	*LINGO2, MIR873, MIR876*
8	15320997	15667796	346800	4	*LINGO2*
8	15667796	15716338	48543	6	*LINGO2*
8	15716338	15765399	49062	5	*LINGO2*
8	17184834	17568938	384105	2	*CAAP1, IFT74, LOC101904667, LRRC19, PLAA, TEK, TRNAC-GCA*
8	19837985	20005984	168000	2	*LOC100296162, LOC104969286, LOC104969287*
8	20005984	20031875	25892	2	.

8	22692098	23269512	577415	4	*IFN-TAU, IFN-tau-c1, IFNA16, IFNAC, IFNAD, IFNAG, IFNAH, IFNB3, IFNT2, IFNW1, INFAF, KLHL9, LOC100298530, LOC100298573, LOC100299481, LOC100335340, LOC100335490, LOC100337470, LOC100847720, LOC100847941, LOC104968438, LOC104969292, LOC104969295, LOC104969298, LOC104969299, LOC107132684, LOC107132685, LOC513706, LOC515485, LOC515953, LOC523244, LOC523509, LOC538620, LOC616463, LOC616977, LOC617112, LOC617135, LOC617556, LOC618801, LOC618859, LOC618943, LOC618947, LOC618985, LOC619113, LOC781778. LOC781815. LOC781853. LOC781948. LOC783912*
8	23269512	23442890	173379	2	*HACD4, IFNB1, IL6, LOC100848709, LOC101904152, LOC101904352, LOC107131314, LOC107132683, LOC107132686, LOC517108, LOC525550, LOC784525, LOC787343*
8	24973934	25500923	526990	4	*DENND4C, HAUS6, LOC104969307, LOC107131197, LOC107132687, PLIN2, RPS6, RRAGA, SAXO1*
8	31318886	31506133	187248	2	*MPDZ*
8	35350784	35555906	205123	2	*LOC101904827*
8	41496231	42488158	991928	2	*KCNV2, LOC100139044, LOC101905621, LOC101905770, LOC101905923, LOC101905994, LOC104969336, LOC104972899, LOC107131327, LOC107132691, PUM3, RFX3, VLDLR*
8	43739945	43752061	12117	2	.
8	43752061	44388345	636285	4	*DMRT1, DMRT2, DMRT3, KANK1, LOC101906315*
8	44388345	44467119	78775	6	*DOCK8, KANK1*
8	44467119	44774042	306924	7	*CBWD2, DOCK8, LOC101906398, LOC529657*
8	45600092	46492323	892232	2	*APBA1, C8H9orf135, FAM189A2, FXN, LOC101902390, LOC104969349, PTAR1, TJP2, TRNAY-GUA*
8	46492323	46658362	166040	2	.
8	47906136	48157753	251618	2	*LOC107132693*
8	48157753	48314369	156617	3	.
8	48314369	48361186	46818	4	.
8	48361186	48550867	189682	3	*LOC101904574, TMEM2*
8	53852427	54953273	1100847	2	*CEP78, GNA14, GNAQ, LOC101907250, LOC104969366, LOC104972905, LOC107132694, PSAT1, VPS13A*
8	54953273	55341984	388712	4	*LOC515468*
8	55341984	55461686	119703	3	.
8	61532015	62479739	947725	2	*FBXO10, GRHPR, LOC100849747, LOC101904207, LOC101907860, LOC104972910, PAX5, POLR1E, TOMM5, TRNAC-GCA, ZBTB5, ZCCHC7*
8	62479739	62585496	105758	3	*FRMPD1, LOC101909682*
8	62585496	63176337	590842	3	*DCAF10, EXOSC3, FRMPD1, LOC101904753, LOC101905995, LOC104969378, LOC104969379, LOC514189, LOC782838, MIR2472, MIR2473, MIR2474, SHB, SLC25A51, TRMT10B*
8	65364422	65476773	112352	6	*LOC101908166*
8	65476773	66108488	631716	5	*ERP44, INVS, LOC101908166, LOC107132702, NR4A3, STX17, TEX10*
8	66108488	66131519	23032	4	.
8	66131519	66179554	48036	6	*LOC527795*
8	66179554	66266313	86760	5	*LOC101902217, LOC527795*
8	66266313	66525208	258896	4	*LOC100297540, LOC101902217, LOC107132703, LOC107132704, LOC784042, LOC784079, LOC784731*
8	67704620	68044247	339628	2	*LOC100298923, LOC100848109, LOC784966, LPL*
8	69722173	69910884	188712	3	*GFRA2, TRNAC-GCA, TRNAG-UCC*
8	69910884	70504019	593136	3	*BMP1, DMTN, DOK2, FAM160B2, GFRA2, HR, LGI3, LOC104969383, LOC104969384, MIR320A-1, NPM2, NUDT18, PHYHIP, PIWIL2, POLR3D, REEP4, SFTPC, TRNAG-CCC, XPO7*
8	70504019	71198441	694423	2	*BIN3, C8H8orf58, CCAR2, EGR3, LOC104969396, LOC107132705, PDLIM2, PEBP4, PIWIL2, PPP3CC, RHOBTB2, SLC39A14, SORBS3*
8	71691617	71821814	130198	2	*ENTPD4, LOC104972922, LOXL2*
8	71821814	72101145	279332	2	*ENTPD4, LOC101904726, LOC107132707, NKX2-6, NKX3-1, SLC25A37*
8	73860300	74224793	364494	2	*CDCA2, DOCK5, GNRH1, KCTD9, LOC107132709, MIR2285AE*
8	76297003	76792433	495431	5	*APTX, B4GALT1, CHRNA2, DNAJA1, EPHX2, GULO, LOC100848199, LOC101902886, LOC107132710, LOC107132711, LOC781261, LOC781381, PTK2B, SMU1, SPINK4*
8	76792433	77203530	411098	4	*AQP3, AQP7, BAG1, CHMP5, LOC107131236, NFX1, NOL6, UBAP2, UBE2R2*
8	77203530	77298073	94544	2	*DCAF12, UBAP2*
8	77298073	78042268	744196	2	*ARID3C, C8H9orf24, CCL19, CCL21, CCL27, CNTFR, DCAF12, DCTN3, DNAI1, ENHO, FAM205A, FAM219A, GALT, IL11RA, KIF24, LOC104969408, LOC508933, LOC783070, NUDT2, RASEF, RPP25L, SIGMAR1, UBAP1*
8	80774245	80988795	214551	6	*AGTPBP1, LOC784808*
8	80988795	81389376	400582	5	*C8H9orf153, GOLM1, ISCA1, LOC101906316, NAA35, ZCCHC6*
8	89229645	89341375	111731	3	.
8	89341375	89458582	117208	3	*LOC101902908*
8	89458582	89551775	93194	3	*LOC101902908*
8	89551775	89778476	226702	3	*LOC100336643*
8	89778476	89822039	43564	6	.
8	89822039	89829752	7714	5	.
8	89829752	89854996	25245	4	.
8	89854996	89906249	51254	3	.
8	89906249	89989985	83737	2	.
8	89989985	90357779	367795	2	.
8	91240353	91387494	147142	2	.
8	91387494	91649076	261583	3	*LOC100140258, NXNL2*
8	91649076	91991292	342217	3	*LOC100298873, LOC101902554, LOC101902749, LOC104969476, SPIN1*
8	91991292	92276536	285245	2	*CDK20, FBXW12, LOC101904508, LOC107132723, LOC617539*
8	92276536	92299561	23026	2	.
8	92299561	92478137	178577	2	*MURC, TMEFF1*
8	92556259	92911505	355247	2	*LOC104969481*
8	92911505	93056211	144707	5	*PLPPR1*

8	93056211	93350931	294721	5	*ALDOB, LOC104969482, LOC530653, LOC786798, MRPL50, PLPPR1, TMEM246, ZNF189*
8	93350931	93393803	42873	5	*TMEM246*
8	93393803	94093105	699303	8	*GRIN3A, LOC101906606, LOC101906688, LOC104969483, LOC107132724, PPP3R2, RNF20*
8	94093105	94846979	753875	8	*CYLC2, LOC104972935*
8	94846979	95256966	409988	6	.
8	95256966	95265313	8348	9	.
8	95265313	95324801	59489	9	.
8	95324801	95647189	322389	9	*LOC104972936*
8	95647189	95731163	83975	4	.
8	95731163	96328919	597757	3	*LOC100337180, LOC101906834, LOC101906978, LOC107132725, LOC107132728, LOC107132729, LOC107132730, LOC509830, OR13F1, SMC2*
8	96328919	96765790	436872	2	*LOC100140461, LOC107132726, LOC107132727, LOC513099, LOC783328, LOC783367, LOC788390, LOC788401, LOC789766, LOC789787, LOC789802, LOC789810, LOC789812, LOC789815, LOC789817, LOC789943, OR13C3, OR13C8*
8	96765790	96785852	20063	3	.
8	96785852	97682095	896244	3	*ABCA1, FKTN, FSD1L, LOC101902998, LOC104969470, LOC785559, LOC785644, NIPSNAP3A, SLC44A1, TAL2*
8	100295224	100325688	30465	2	.
8	100325688	101682436	1356749	3	*ACTL7A, ACTL7B, CTNNAL1, EPB41L4B, FAM206A, FRRS1L, IKBKAP, LOC100847230, LOC104969439, LOC107132734, LOC783421, MIR32, PALM2, PTPN3, TMEM245, TRNAC-GCA, TRNAE-UUC*
8	102581024	102780465	199442	6	*LOC101907488, LPAR1, TRNAC-GCA*
8	102780465	102816330	35866	5	*MIR2285N-5*
8	102816330	103279305	462976	4	*KIAA0368, LOC539574, PTGR1, ZNF483*
8	103279305	103613163	333859	7	*C8H9orf84, DNAJC25, GNG10, LOC104972945, MIR7863, TRNAC-ACA, UGCG*
8	103613163	104269620	656458	2	*HSDL2, INIP, KIAA1958, LOC101905894, LOC534155, PTBP3, SUSD1*
8	104269620	104566787	297168	2	*INIP, LOC101906267, LOC104969447, LOC104969451, LOC513329, LOC783399, SLC46A2, SNX30*
8	106244842	106853824	608983	2	*LOC101908167, LOC104969458, LOC107132740, TNC, TNFSF15, TNFSF8*
8	106853824	106918003	64180	2	.
8	108615037	109083273	468237	6	*ASTN2, TRNAS-AGA*
8	109083273	109352447	269175	4	.
8	111031030	111315197	284168	5	*BRINP1*
8	111315197	111643825	328629	4	*LOC101903765*
8	111643825	111657155	13331	3	*LOC100848999, LOC101903765, LOC104968520*
9	5788	157338	151551	3	*LOC100139593, LOC100336757*
9	157338	321444	164107	4	*LOC784048, LOC786133, LOC788937, LOC788947, LOC789025, LOC789033*
9	321444	1137141	815698	5	*LOC107132746, LOC616304, PHF3, PTP4A1*
9	2974899	3467064	492166	2	.
9	20961034	21039749	78716	5	.
9	21039749	21192036	152288	4	.
9	21192036	21723680	531645	6	*LOC104972971*
9	21723680	23133818	1410139	2	*FAM46A, IBTK, LOC100138784, LOC101906475, LOC104969506, LOC107132757, TPBG*
9	27337323	27664454	327132	2	.
9	27664454	29613821	1949368	7	*CLVS2, FABP7, HSF2, LOC100848869, LOC101904186, LOC101905260, LOC101908885, LOC101909028, LOC107132761, LOC781754, MIR2478, PKIB, SERINC1, SMPDL3A, TRDN*
9	29613821	31022040	1408220	4	*GJA1, LOC100295149, LOC101905771, LOC104969516, TBC1D32*
9	31022040	31044751	22712	6	.
9	31044751	31198107	153357	8	.
9	31198107	31218966	20860	9	.
9	31218966	33634792	2415827	9	*ASF1A, CEP85L, FAM184A, LOC100848035, LOC101906051, LOC104969513, LOC614138, LOC782839, MAN1A1, MCM9, PLN, SLC35F1, TRNAK-UUU, TRNAV-CAC*
9	35417671	35987129	569459	3	*LOC100140173*
9	43486563	43977997	491435	4	*BEND3, C9H6orf203, CD24, LOC100137843, LOC104969546, LOC107132765, LOC614649, PDSS2, QRSL1*
9	43977997	44173280	195284	5	*AIM1, QRSL1, RTN4IP1*
9	44173280	44192510	19231	6	*AIM1*
9	44192510	45282530	1090021	5	*AIM1, ATG5, LOC100336714, LOC100847546, LOC104969548, LOC104972981, LOC107132766, LOC781322, LOC784251, PRDM1*
9	45282530	46382936	1100407	5	*BVES, HACE1, LOC100138772, LOC101902273, LOC104969553, POPDC3, PREP*
9	46382936	46804617	421682	8	*LOC785087*
9	46804617	47195595	390979	10	*LOC101902933, TRNAC-ACA, TRNAM-CAU*
9	47195595	47264584	68990	11	.
9	47264584	49939450	2674867	10	*ASCC3, GRIK2, LOC104972983, LOC107132767, LOC528043, LOC783945*
9	49939450	50065016	125567	4	*ASCC3*
9	50065016	50231581	166566	5	*ASCC3, LOC518885*
9	50231581	50297434	65854	2	*ASCC3*
9	50297434	52466829	2169396	3	*ASCC3, CCNC, COQ3, FAXC, FBXL4, LOC101904646, LOC101904783, LOC101904829, LOC104969561, LOC107132768, LOC107132769, LOC781964, LOC781989, MCHR2, PNISR, POU3F2, PRDM13, SIM1, TRNAC-GCA, TSTD3, USP45*
9	52466829	53212056	745228	2	*LOC788043, TRNAS-GGA, TRNAY-GUA*
9	53212056	53806563	594508	7	*KLHL32, LOC100848581, LOC104969563, MMS22L*
9	53806563	55006264	1199702	6	*FHL5, FUT9, GPR63, LOC100336742, LOC104969564, LOC107132770, LOC782007, NDUFAF4, TRNAC-GCA, TRNAG-UCC, UFL1*
9	55006264	55070966	64703	6	.
9	55070966	56327104	1256139	6	*LOC783932, MANEA*

9	59994749	63677782	3683034	2	*AKIRIN2, ANKRD6, BACH2, CASP8AP2, CFAP206, CNR1, GABRR1, GABRR2, GJA10, LOC100140683, LOC100336843, LOC100847428, LOC101902479, LOC101903075, LOC104969735, LOC104970129, LOC107132771, LOC107132772, LOC107132773, LOC782675, LYRM2, MAP3K7, MDN1, MIR2903, MIR7862, ORC3, PM20D2, PNRC1, RARS2, RNGTT, RRAGD, SLC35A1, SPACA1, SRSF12, TRNAD-GUC, TRNAE-UUC, UBE2J1*
9	63677782	63888234	210453	6	*C9H6orf163, GJB7, LOC104969583, LOC107132774, SMIM8, ZNF292*
9	63888234	65176462	1288229	4	*CGA, HTR1E, LOC100174924, LOC509829, NT5E, SNX14, SYNCRIP, ZNF292*
9	65176462	65285014	108553	3	*LOC100335322, LOC101906498, LOC104968426, LOC509791*
9	65285014	65312497	27484	2	.
9	65312497	65692509	380013	4	*LOC101907349, LOC104970099*
9	65692509	66291280	598772	4	*LOC101907627, LOC107132775, TBX18*
9	72399930	73421315	1021386	2	*EYA4, LOC104969595, TCF21*
9	84366153	84917546	551394	4	*EPM2A, FBXO30, GRM1, SHPRH*
9	86227784	86352129	124346	2	*LOC100850276, SAMD5*
9	86352129	86711520	359392	3	*SAMD5*
9	86711520	88665014	1953495	8	*GINM1, KATNA1, LATS1, LOC100299874, LOC100336795, LOC101903261, LOC104969619, LOC104972992, LOC107131280, LOC107132784, LOC107132785, LOC107132786, LOC782566, LOC784274, LRP11, NUP43, PCMT1, PPIL4, RAET1G, SASH1, TAB2, ULBP27, UST, ZC3H12D*
9	88665014	88819879	154866	6	*LOC104969628, LOC107132787, LOC513548, LOC786867, PPP1R14C, ULBP21*
9	88819879	88999220	179342	3	*LOC100848282, PPP1R14C*
9	88999220	89361599	362380	8	*IYD, LOC100848331, LOC104972994, LOC785982, PLEKHG1*
9	89361599	90914671	1553073	4	*AKAP12, ARMT1, CCDC170, ESR1, LOC100335416, LOC100848490, LOC101904510, LOC104969623, LOC104969627, LOC104972994, LOC104972995, LOC107131177, LOC107132788, LOC107132789, LOC107132790, MIR2285E-2, MTHFD1L, PLEKHG1, RMND1, SYNE1, ZBTB2*
9	90914671	91600639	685969	5	*FBXO5, LOC107131177, MTRF1L, MYCT1, RGS17, TRNAC-ACA, TRNAS-GGA, VIP*
9	91600639	94000874	2400236	3	*CLDN20, CNKSR3, IPCEF1, LOC100298280, LOC100848475, LOC104970693, LOC104972996, LOC107132791, LOC616500, LOC616595, LOC617457, LOC783190, LOC788337, LOC789941, LOC789961, MIR2480, NOX3, OPRM1, OR4A15, OR4C6, SCAF8, TFB1M, TIAM2*
9	94000874	94091133	90260	5	.
9	94091133	94729591	638459	4	*LOC104969630*
9	104510643	104883534	372892	2	*DACT2, LOC101907159, LOC104969666, LOC107132802, LOC107132803, LOC788572, SMOC2*
9	104883534	106206340	1322807	2	*C9H6orf120, DLL1, ERMARD, FAM120B, LOC101907681, LOC104969678, LOC107132805, LOC107132806, PDCD2, PHF10, PSMB1, TBP, TCTE3, THBS2, WDR27*
10	1184	99231	98048	14	*LOC104973003*
10	99231	897998	798768	4	*LOC101902502, LOC101902806, LOC101902859, LOC101909710, LOC104973003, LOC104973005, LOC783211, LOC783248, LOC787243, MCC*
10	897998	1513285	615288	2	*APC, DCP2, EPB41L4A, LOC104973004, MCC, REEP5, SRP19*
10	1513285	1895590	382306	3	*EPB41L4A*
10	1895590	2056531	160942	3	.
10	2056531	2509844	453314	6	*LOC101903138, LOC101903289, LOC104973030, LOC107132807, LOC785663, NREP, YTHDC2*
10	2509844	2657561	147718	7	.
10	2657561	2678719	21159	4	.
10	7794882	7805981	11100	2	*F2RL2, IQGAP2, LOC107132815*
10	14032572	14657034	624463	2	*AAGAB, C10H15orf61, IQCH, LOC104973050, MAP2K5*
10	17498048	17886166	388119	2	*LARP6, LOC101903602, LOC104973065, LOC107132827, LRRC49, THAP10, UACA*
10	22054922	22453121	398200	3	*ABHD4, DAD1, LOC100336282, LOC100337215, LOC100337505, LOC101908015, LOC101908096, LOC104969841, LOC104973085, LOC785995, LOC786614*
10	23333085	23469583	136499	2	*LOC100296102, LOC104968717, LOC104969863, LOC107131332, LOC107132829, LOC786594, LOC786608, LOC786717, LOC786802, LOC786881, TRNAW-CCA*
10	25647369	25702685	55317	2	*LOC522609, OR4E2*
10	31551834	32252502	700669	2	*TRNAC-GCA, TRNAS-AGA*
10	33975663	34620089	644427	2	*FAM98B, LOC104973119, LOC107132843, RASGRP1, SPRED1*
10	34620089	34714231	94143	3	.
10	34714231	35136864	422634	2	*C10H15orf54, LOC104973121, LOC104973122, LOC107132846*
10	35136864	35193883	57020	3	*LOC104973122*
10	35193883	35221127	27245	3	.
10	45517223	46264103	746881	2	*CSNK1G1, DAPK2, FAM96A, KIAA0101, LOC100336855, LOC101905285, LOC101907658, LOC107132856, OAZ2, PPIB, RBPMS2, SNX1, SNX22, TRIP4, ZNF609*
10	55251483	55410939	159457	2	*LOC104973165, PIGB, RAB27A, RSL24D1*
10	55410939	55584199	173261	2	.
10	59586472	59709071	122600	2	*LOC104973172, TNFAIP8L3*
10	59709071	60424977	715907	2	*AP4E1, GABPB1, HDC, LOC101906656, LOC101906715, LOC101906880, LOC101906954, LOC104973174, LOC107132871, LOC107132872, SPPL2A, TNFAIP8L3, TRPM7, USP50, USP8*
10	60424977	60596181	171205	2	*ATP8B4, SLC27A2*
10	60596181	60688571	92391	5	*ATP8B4*
10	60688571	62824288	2135718	4	*ATP8B4, CEP152, COPS2, CTXN2, DTWD1, DUT, EID1, FAM227B, FBN1, FGF7, GALK2, LOC101907659, LOC104973175, LOC104973177, LOC107132866, LOC613512, MYEF2, SECISBP2L, SHC4, SLC12A1, SLC24A5*
10	71608828	71705664	96837	2	*LOC104973193*
10	71862617	72049250	186634	2	*DAAM1*
10	72049250	72532880	483631	2	*CCDC175, DAAM1, GPR135, JKAMP, L3HYPDH, LOC104973196, LOC781059, RTN1*
10	72532880	72844509	311630	2	*LOC101902937, LOC537580, PCNX4, RTN1*
10	72844509	73305474	460966	4	*C10H14orf39, DHRS7, PPM1A, SIX6, TRNAE-UUC, TRNAQ-UUG*
10	75279800	75626927	347128	2	*KCNH5, LOC107132878*
10	77811225	78350723	539499	2	*FUT8, MAX, TRNAC-GCA*
10	78350723	78557862	207140	2	*FUT8, LOC104973214, LOC104973215*
10	84474223	84599159	124937	2	*LOC104973236, RGS6, TRNAE-UUC*

10	84994696	86057787	1063092	2	*ACOT2, ACOT4, ACOT6, BBOF1, C10H14orf169, COQ6, DCAF4, DNAL1, DPF3, ELMSAN1, ENTPD5, FAM161B, HEATR4, LOC100847232, LOC101903632, LOC101904062, LOC101905999, LOC504425, LOC512541, MIR7859, NUMB, PAPLN, PNMA1, PSEN1, PTGR2, RBM25, TRNAE-UUC, ZFYVE1, ZNF410*
10	89582936	89711754	128819	3	*CIPC, LOC101904398, LOC101904443, ZDHHC22*
10	91214524	91478479	263956	5	.
10	92766156	93033744	267589	2	*DIO2, LOC101905198, LOC104973262, TRNAC-GCA*
10	94614742	95133604	518863	2	*LOC104970660, LOC107132890, LOC785767*
10	95133604	95208623	75020	2	.
10	95208623	95324014	115392	2	.
10	95324014	95406197	82184	2	.
10	96423256	96627858	204603	2	.
10	99167477	99877446	709970	2	*LOC511898, LOC616051, LOC785091, TRNAC-ACA*
10	100259170	100898371	639202	2	*LOC100295545*
10	103715935	104465097	749163	11	*CASC4, CTDSPL2, EIF3J, LOC101901960, LOC101902036, LOC101908218, LOC104973277, LOC107132892, PATL2, RPS6KA5, SPG11, TTC7B*
10	104465097	104654089	188993	12	*B2M, C10H15orf43, LOC104973278, LOC508285, LOC783680, TRIM69*
11	1141	75423	74283	4	*LOC786581, LOC786619, ZC3H6*
11	75423	440285	364863	2	*FBLN7, MERTK, TMEM87B, ZC3H6, ZC3H8*
11	8383146	8999583	616438	4	*LOC101903633, LOC101909815*
11	8999583	9214697	215115	4	*LOC101909778, LOC101909815, LOC107132905, LOC107132906, MRPS9*
11	10876798	11463279	586482	3	*ALMS1, CCT7, DUSP11, EGR4, FBXO41, LOC101902604, LOC101903026, LOC101903097, MIR2294, MIR2295, NOTO, PRADC1, RAB11FIP5, SFXN5, SMYD5, TPRKB*
11	13157358	13243265	85908	2	*DYSF, LOC101903215, ZNF638*
11	13243265	13367286	124022	2	*ZNF638*
11	14382588	14572571	189984	3	*LOC107132910, SRD5A2*
11	16562178	16756034	193857	4	.
11	16756034	16761823	5790	3	.
11	16761823	16842594	80772	5	.
11	16842594	17466455	623862	5	*LOC107132909, LOC782032*
11	17466455	17878882	412428	4	.
11	20780234	22242558	1462325	4	*ARHGEF33, ATL2, CDKL4, DHX57, GALM, GEMIN6, HNRNPLL, LOC100847282, LOC104973309, LOC104973310, LOC107132913, LOC107132914, LOC782437, LOC782845, LOC783737, MAP4K3, MIR2284Z-2, MORN2, SOS1, SRSF7, THUMPD2, TMEM178A*
11	26519082	26873588	354507	4	*CAMKMT, LOC101906743, PPM1B, PREPL, SLC3A1*
11	26873588	27456726	583139	2	*CAMKMT, LOC101906905, LOC104973324, LOC104973326, LOC107132919, SIX2, SIX3, TRNAE-UUC*
11	31859281	33134037	1274757	2	*NRXN1*
11	34244293	36063575	1819283	2	*LOC101901933, LOC104973343*
11	40086176	41325481	1239306	3	*FANCL, LOC107132926, TRNAC-ACA, TRNAC-GCA, VRK2*
11	43865434	44329286	463853	3	*EVA1A, GCFC2, KIAA1841, LOC100139010, LOC101904087, LOC104973365, LOC615002, MRPL19, SEPT10, SOWAHC*
11	45205464	45357605	152142	3	.
11	45357605	47265277	1907673	3	*C11H2orf40, CHCHD5, CKAP2L, IL1A, IL1B, IL1F10, IL1RN, IL36A, IL36B, IL36G, IL36RN, IL37, LOC100294952, LOC100335748, LOC100847269, LOC101903687, LOC101903923, LOC101904177, LOC104973371, LOC107132933, LOC107132936, LOC107132937, LOC532089, LOC614219, LOC786288, NCK2, NT5DC4, PAX8, POLR1B, PSD4, SLC20A1, ST6GAL2, TTL, UXS1*
11	65532376	69582688	4050313	2	*AAK1, ANTXR1, ANXA4, APLF, ARHGAP25, ASPRV1, BMP10, C11H2orf42, C1D, CAPN13, CAPN14, CNRIP1, EHD3, ETAA1, FBXO48, GALNT14, GFPT1, GKN1, GKN2, GMCL1, LCLAT1, LOC100139826, LOC101904865, LOC101905499, LOC101905676, LOC104973409, LOC104973410, LOC104973411, LOC104973412, LOC107132938, LOC107132939, LOC509961, LOC786621, LOC787229, MXD1, NFU1, PCBP1, PCYOX1, PLEK, PNO1, PPP3R1, PROKR1, SNRNP27, SNRPG, TIA1, TRNAG-CCC, WDR92*
11	69582688	69598132	15445	3	*LCLAT1*
11	69598132	70386497	788366	2	*LBH, LCLAT1, LOC101905873, LOC104968430, YPEL5*
11	74496513	75047830	551318	2	*ADCY3, CENPO, DNAJC27, LOC107132952, NCOA1, PTRHD1*
11	76288153	76553347	265195	7	*LOC101908123, LOC107132946*
11	79651365	80061687	410323	4	*OSR1*
11	98013442	98401269	387828	3	*ANGPTL2, GARNL3, RALGPS1*
11	99381647	99591279	209633	2	*C11H9orf114, ENDOG, LOC101908339, LOC104973489, PKN3, SET, SPTAN1, TBC1D13, WDR34, ZDHHC12, ZER1*
11	99591279	99611209	19931	2	*C11H9orf114, CCBL1*
12	735504	907553	172050	3	.
12	907553	941221	33669	2	.
12	941221	1034660	93440	3	.
12	1034660	1332026	297367	2	*LOC540096*
12	1332026	1556918	224893	5	*TDRD3*
12	1556918	1839307	282390	14	*LOC104973528, TDRD3*
12	1839307	1877866	38560	12	.
12	1877866	1903851	25986	11	*LOC100140262*
12	1903851	2887203	983353	7	*DIAPH3, LOC100140262, LOC104973529, LOC789865*
12	3856446	4304312	447867	4	.
12	4304312	4321010	16699	6	.
12	4321010	4566741	245732	6	.
12	4566741	4782788	216048	6	.
12	6183477	6390654	207178	5	.
12	6390654	6621592	230939	2	.
12	6621592	7891018	1269427	2	*LOC100141157*
12	7920825	8051329	130505	5	.
12	8051329	8527084	475756	4	.
12	8527084	8540293	13210	2	.
12	17456211	18152802	696592	2	*LOC104973549, LOC107132977, LOC783657, MED4, NUDT15, SUCLA2*

12	18152802	18429320	276519	3	*ITM2B, LOC101903713, LOC506251, LPAR6, RB1, RCBTB2, TRNAE-UUC*
12	18429320	18738807	309488	2	*CYSLTR2, LOC101903925*
12	23594944	23932617	337674	3	*UFM1*
12	37898367	37924877	26511	2	.
12	37924877	38461763	536887	2	*LOC100296100*
12	40007229	40521012	513784	2	*PCDH9*
12	50144781	50291271	146491	2	.
12	50291271	50508384	217114	3	.
12	60696090	60980279	284190	2	*LOC104973615*
12	60980279	61520260	539982	2	*SLITRK6, TRNAC-GCA*
12	61520260	62400058	879799	2	*LOC100139019, LOC781157*
12	62400058	62903304	503247	10	*SLITRK5*
12	62903304	63064239	160936	6	.
12	63064239	63420912	356674	8	*LOC107133002*
12	63420912	63668217	247306	9	.
12	63668217	63770247	102031	7	.
12	63770247	64927326	1157080	2	*LOC107133003, LOC616944*
12	64927326	65090750	163425	6	.
12	65090750	65183055	92306	6	.
12	65183055	65383845	200791	4	.
12	65383845	65513040	129196	5	*LOC101907749*
12	66991721	67739127	747407	7	*GPC5*
12	67739127	68297800	558674	2	*LOC101902424, LOC104970044, LOC104973623*
12	70686914	71095315	408402	6	*LOC101903142, LOC101903219, LOC104973632, LOC107131272, LOC107133005, LOC784305*
12	71095315	71271064	175750	3	*LOC100336232, LOC100848652, LOC101902253, LOC101903219, LOC510333*
12	71271064	71506447	235384	2	*LOC100336232, LOC100848700, LOC101902419, LOC107133006*
12	72560494	72588022	27529	2	*LOC100337069*
12	72588022	72700406	112385	2	*LOC100337069, LOC101902505*
12	77246683	77829502	582820	5	*DNAJC3, DZIP1, HS6ST3, LOC785064, UGGT2*
12	77829502	78177583	348082	13	*HS6ST3, TRNAG-CCC*
12	78177583	78301251	123669	7	*HS6ST3*
12	78301251	78452482	151232	9	*HS6ST3, LOC107133014, LOC782740*
12	78452482	78851744	399263	5	*LOC107133015, MBNL2, OXGR1, TRNAC-GCA*
12	78851744	79078553	226810	9	*LOC104973647, RAP2A*
12	79078553	79333664	255112	8	.
12	80648029	80662845	14817	2	.
12	80662845	81576880	914036	5	*CLYBL, GGACT, LOC100140038, LOC101905905, LOC101906778, LOC104973656, LOC107133016, LOC787451, MIR2285N-1, PCCA, TM9SF2, TRNAG-ACC, TRNAG-CCC, ZIC2*
12	81576880	81733302	156423	8	*LOC100848638, MIR2892, TMTC4*
12	83234978	83453920	218943	2	*BIVM, KDELC1, LOC101906350, LOC101906427, LOC104973658, METTL21C, TEX30, TPP2*
13	71	167957	167887	11	*LOC101906171, LOC104968831, LOC107133020, LOC616519*
13	167957	192608	24652	9	*LOC107133020, LOC788047*
13	192608	258305	65698	7	*LOC100298428, LOC788064*
13	258305	289652	31348	5	*LOC100298428, LOC101909256*
13	289652	352165	62514	6	*LOC100296695, LOC788089, LOC788106*
13	352165	506060	153896	9	*LOC788120*
13	506060	728955	222896	3	*TMX4*
13	728955	869152	140198	4	*PLCB1*
13	869152	1039007	169856	4	*PLCB1*
13	1213727	1292999	79273	2	*PLCB1*
13	1292999	1511344	218346	3	*MIR2285M-1, PLCB1*
13	1511344	2199763	688420	2	*LOC107133021, PLCB1*
13	2199763	2415434	215672	4	*PLCB4*
13	2415434	2577850	162417	3	*LAMP5, MGC148692, PAK7, PLCB4*
13	3907070	4819405	912336	2	*LOC101901915, LOC104973695*
13	7243912	7495673	251762	5	*ESF1, TASP1*
13	7495673	8239622	743950	4	*ESF1, FLRT3, LOC104973699, MACROD2, NDUFAF5, SEL1L2*
13	8239622	8436068	196447	3	*MACROD2*
13	13955780	14115557	159778	3	.
13	14115557	14304689	189133	4	.
13	14304689	14682257	377569	2	.
13	17033465	17766303	732839	2	*FBXO18, IL15RA, IL2RA, LOC101904563, LOC101904996, LOC104973718, LOC104973720, PFKFB3, PRKCQ, RBM17, TRNAF-GAA*
13	17766303	17879515	113213	2	*LOC101909551, LOC513969, LOC615768, LOC619000*
13	17879515	17942101	62587	2	*LOC101903895, LOC101904138, LOC104968423, LOC522845*
13	17942101	18138448	196348	3	*ACBD5, ANKRD26, LOC101902607, LOC104973722, LOC782950, MASTL, YME1L1*
13	18138448	18154688	16241	2	.
13	18154688	18167166	12479	2	.
13	39611279	40351058	739780	3	*CFAP61, CRNKL1, LOC101905046, LOC101905154, LOC530211, MIR2305, NAA20, RIN2, SLC24A3*
13	41586589	41981780	395192	3	.
13	44669372	44773729	104358	4	.
13	46695895	48280994	1585100	3	*ADARB2, CDS2, DIP2C, GTPBP4, IDI1, LARP4B, LOC101904942, LOC101905075, LOC101905155, LOC107133051, LOC107133053, LOC787679, LOC787698, MIR2285M-2, PCNA, PRND, PRNP, PROKR2, RASSF2, SLC23A2, TMEM230, WDR37, ZMYND11*
13	49991135	50171444	180310	2	.
13	52034362	52372503	338142	8	*ADAM33, AP5S1, ATRN, C13H20orf27, CDC25B, CENPB, GFRA4, HSPA12B, LOC104976448, LOC507743, SIGLEC1, SPEF1*
13	52372503	53090397	717895	10	*ATRN, AVP, C13H20orf141, C13H20orf194, CPXM1, DDRGK1, FASTKD5, GNRH2, ITPA, LOC101903899, LOC104973820, MRPS26, OXT, PCED1A, PROSAPIP1, PTPRA, SLC4A11, TMEM239, UBOX5, VPS16*
13	53090397	53292643	202247	8	*EBF4, IDH3B, LOC101902712, LOC101904587, NOP56, TMC2*

13	53292643	55800220	2507578	4	*ABHD16B, ADRM1, ARFGAP1, ARFRP1, BHLHE23, BIRC7, C13H20orf195, CABLES2, CHRNA4, COL20A1, COL9A3, DIDO1, DNAJC5, EEF1A2, GATA5, GID8, GINS1, GMEB2, HRH3, KCNQ2, LAMA5, LIME1, LKAAEAR1, LOC100847256, LOC100848770, LOC101902869, LOC101904896, LOC101905484, LOC104973823, LOC104973824, LOC104973826, LOC104973827, LOC104973829, LOC104973830, LOC107131125, LOC107133055, LOC107133069, LOC107133070, LOC536097, LOC615706, LOC786039, LOC786845, LOC789034, LSM14B, MIR1-1, MIR124B, MIR133A-1, MIR133C, MIR1388, MIR2306, MRGBP, MTG2, MYT1, NPBWR2, NTSR1, OGFR, OPRL1, OSBPL2, PCMTD2, PDYN, PPDPF, PRPF6, PSMA7, PTK6, RBBP8NL, RGS19, RPS21, RTEL1, SAMD10, SIRPA, SIRPB1, SLC17A9, SLC2A4RG, SLCO4A1, SNRPB, SOX18, SRMS, SS18L1, STK35, STMN3, TCEA2, TCFL5, TGM3, TGM6, TMC2, TNFRSF6B, TPD52L2, UCKL1, YTHDF1, ZBTB46, ZGPAT, ZNF512B*
13	55800220	55950654	150435	10	*CDH4, LOC101906818, TAF4, TRNAW-CCA*
13	56518267	56858000	339734	2	.
13	63271803	63309494	37692	6	*BPIFA2A*
13	63309494	63741640	432147	8	*BPIFA1, BPIFA2B, BPIFA2C, BPIFA2D, BPIFA3, BPIFA4, BPIFB1, BPIFB5, CDK5RAP1, LOC100848568, LOC101906405, LOC101908058, LOC107133057, LOC107133078, SNTA1, TRNAG-CCC*
13	63741640	64078829	337190	23	*ACTL10, C13H20orf144, CBFA2T2, CHMP4B, E2F1, LOC101901940, LOC785968, NECAB3, PXMP4, SNTA1, TRNAC-GCA, ZNF341*
13	64078829	64377115	298287	27	*CHMP4B, EIF2S2, RALY*
13	64377115	64401804	24690	29	.
13	64401804	64475104	73301	31	.
13	64475104	64640895	165792	26	*AHCY, ASIP, ITCH, LOC782136, TRNAG-CCC*
13	64640895	66868834	2227940	9	*AAR2, ACSS2, C13H20orf173, C13H20orf24, CEP250, CNBD2, CPNE1, DLGAP4, DYNLRB1, EDEM2, EIF6, EPB41L1, ERGIC3, FAM83C, GDF5, GGT7, GSS, ITCH, LOC100295994, LOC101903101, LOC101903639, LOC101905432, LOC104973867, LOC104973870, LOC526745, LOC533073, LOC786401, LOC788449, MAP1LC3A, MIR499, MMP24, MYH7B, MYL9, NCOA6, NDRG3, NFS1, PHF20, PIGU, PROCR, RBL1, RBM12, RBM39, ROMO1, SAMHD1, SCAND1, SLA2, SPAG4, TGIF2, TLDC2, TP53INP2, TRNAC-GCA, TRNAE-UUC, TRNAG-CCC, TRNAM-CAU, TRPC4AP, UQCC*
13	66868834	67601611	732778	3	*BLCAP, CTNNBL1, DSN1, GHRH, LOC100849067, LOC104973872, LOC104973873, LOC107133079, LOC107133080, LOC614284, MANBAL, MROH8, NNAT, RBL1, RPN2, SOGA1, SRC*
13	70311992	70422096	110105	3	*LOC101907117, MAFB*
13	74260971	74933957	672987	3	*DBNDD2, ELP, KCNS1, LOC100196900, LOC100295842, LOC100850808, LOC101903403, LOC101903858, LOC104973891, LOC107133082, LOC404105, LOC407163, LOC512548, LOC512838, LOC783797, MATN4, PABPC1L, PI3, PIGT, RBPJL, SDC4, SLPI, STK4, SYS1, TOMM34, TP53TG5, WFDC15B, WFDC5, YWHAB*
13	75241799	75278915	37117	3	*LOC104973892, LOC404061, LOC404103*
13	75278915	75308339	29425	2	*LOC104973892, LOC404061*
13	82576712	82637270	60559	2	*LOC104973930*
13	82637270	82657685	20416	2	.
13	82657685	83072565	414881	2	*CYP24A1, DOK5, PFDN4, TRNAL-UAA*
13	83072565	83172837	100273	7	*DOK5*
13	83172837	83416894	244058	8	*LOC101903665*
13	83416894	83553734	136841	12	.
13	83553734	83682043	128310	11	.
13	83682043	83708467	26425	12	.
13	83708467	84589399	880933	12	*CBLN4, LOC100298309, LOC107133062, TRNAC-GCA, TRNAG-GCC*
14	6427039	7069301	642263	2	.
14	7069301	7253159	183859	3	.
14	19252740	19367880	115141	2	.
14	19367880	19455245	87366	3	*LOC100139328*
14	19470387	19556574	86188	2	*LOC100139328*
14	19556574	20078786	522213	3	*HAS2, LOC107133107, LOC515601, LOC522769*
14	20078786	20230111	151326	6	.
14	20230111	20377777	147667	5	*TRNAM-CAU*
14	20377777	20987889	610113	2	*CEBPD, LOC100297500, LOC100847223, LOC101902180, LOC529047, SPIDR*
14	20987889	21337809	349921	3	*H3F3C, LOC107133112, MCM4, PRKDC, SPIDR*
14	21337809	21549635	211827	2	*LOC100139903, MCM4, UBE2V2*
14	22735944	23478178	742235	4	*FAM150A, LOC100141260, LOC101906226, LOC101906592, LOC104974017, LOC104974020, NPBWR1, PCMTD1, RB1CC1, ST18*
14	23478178	23872616	394439	3	*ATP6V1H, LYPLA1, MRPL15, OPRK1, RGS20, TCEA1*
14	25309184	25350724	41541	2	*SDR16C6*
14	25350724	25413115	62392	2	*LOC101907667, PENK, SDR16C6*
14	25413115	25568995	155881	4	*LOC101907667*
14	25568995	25755179	186185	3	*IMPAD1, LOC101907667*
14	25857221	25879266	22046	2	.
14	25879266	25978896	99631	2	.
14	32716034	33078652	362619	2	*ADHFE1, C14H8orf46, MYBL1, RRS1, ZSCAN5B*
14	33078652	33359502	280851	2	*LOC100847363, LOC104974037, LOC784087, MCMDC2, MYBL1, SGK3, VCPIP1*
14	33359502	33674740	315239	4	*ARFGEF1, COPS5, CSPP1, PPP1R42, TCF24*
14	33674740	33894668	219929	2	*ARFGEF1, CPA6, LOC101902584, TRNAC-GCA*
14	45386678	45595383	208706	2	*LOC100847158, MRPS28*
14	54779793	55485770	705978	2	.
14	56000432	56479255	478824	5	*LOC104974084*
14	56479255	56704593	225339	2	.
14	57413080	57714035	300956	2	*ENY2, NUDCD1, PKHD1L1*
14	57714035	57880030	165996	5	*LOC104974087, TRHR*
14	57880030	57929158	49129	3	*LOC104974087*
14	57929158	57939603	10446	2	.
14	57939603	58003049	63447	2	.
14	58003049	58636410	633362	6	*EMC2, LOC101905702, TMEM74*
14	58636410	59312867	676458	5	*EIF3E, LOC104974091, LOC511981, RSPO2*

14	59312867	59330440	17574	4	.
14	59330440	59658987	328548	4	*ANGPT1, LOC614209*
14	59658987	59722201	63215	4	*ANGPT1*
14	59722201	60225049	502849	3	*ANGPT1, LOC104974093, LOC782496*
14	60225049	60465778	240730	7	*ABRA, LOC100141107, LOC104974093, OXR1*
14	60465778	60641238	175461	5	*OXR1*
14	60641238	60848518	207281	5	*OXR1*
14	60848518	61169916	321399	3	*OXR1*
14	61169916	61197795	27880	4	.
14	61197795	61290348	92554	5	.
14	61290348	61304555	14208	6	.
14	61304555	61364287	59733	5	*LOC107133124, ZFPM2*
14	61364287	61888437	524151	5	*LOC104974098, LOC107133124, ZFPM2*
14	61888437	61970947	82511	5	.
14	61970947	62247363	276417	2	.
14	64133806	64900188	766383	2	*KLF10, LOC104974106, LOC104974107, LOC107133128, LOC107133129, ODF1, RRM2B, TRNAE-UUC, UBR5*
14	65664946	65831750	166805	2	*ZNF706*
14	65831750	66112562	280813	3	*LOC101905128, LOC101907120, YWHAZ*
14	66112562	66439028	326467	3	*ANKRD46, LOC104974111, PABPC1, SNX31, TRNAC-GCA, TRNAY-AUA*
14	66439028	66964190	525163	5	*FBXO43, LOC104972815, POLR2K, RGS22, RNF19A, SPAG1*
14	66964190	67123787	159598	7	*MGC148714, VPS13B*
14	67123787	67148445	24659	6	*VPS13B*
14	67148445	67222961	74517	4	*VPS13B*
14	67222961	68480690	1257730	2	*KCNS2, LOC100298420, LOC104974113, LOC104974115, LOC107133130, MIR599, MIR875, OSR2, STK3, TRNAG-UCC, VPS13B*
14	68480690	68493869	13180	3	*LOC100298420*
14	68493869	68883420	389552	4	*ERICH5, LOC100298420, MATN2, NIPAL2, POP1, RIDA, RPL30*
14	68883420	68959967	76548	3	*MATN2*
14	68959967	69398336	438370	2	*LAPTM4B, LOC104974118, LOC782052, MATN2, MTDH*
14	69398336	69595652	197317	3	*TSPYL5*
14	71322882	71714765	391884	2	.
14	72905561	73185960	280400	5	*LOC104974129, PDP1, TMEM67*
14	73185960	73417045	231086	6	*FAM92A1, LOC107133136, RBM12B*
14	76643509	77788779	1145271	2	*LOC104974150, LOC107131287, LOC783798, LOC786994, MMP16*
14	77788779	77857468	68690	3	.
14	77857468	77968489	111022	3	.
14	78404054	78470889	66836	4	*CNBD1*
14	78470889	78713978	243090	6	*CNBD1*
14	78713978	78902767	188790	7	*CNGB3, CPNE3*
14	78902767	79047749	144983	14	*RMDN1, WWP1*
14	79047749	79106527	58779	12	*LOC104974135, WWP1*
14	79106527	80211641	1105115	5	*ATP6V0D2, C14H8orf59, CA1, CA13, CA2, CA3, E2F5, LOC100138079, LOC100196897, LOC100847874, LOC101903860, LOC104974136, LOC104974137, LOC107133131, LOC784254, LRRCC1, MIR2311, PSKH2, SLC7A13, TRNAS-GGA*
14	80211641	80386978	175338	8	*LOC107133132, LOC506670, LOC518422, LOC787733, RALYL*
14	80386978	80643048	256071	8	*RALYL*
14	80643048	80876286	233239	6	*RALYL*
14	80876286	81384919	508634	5	*LOC616400, RALYL*
14	81384919	81498694	113776	3	.
14	81498694	81523448	24755	2	.
14	82008361	82037031	28671	3	.
14	82037031	82209556	172526	4	.
14	82450651	82700671	250021	2	.
14	83058713	83075571	16859	4	.
14	83075571	83125396	49826	8	.
14	83125396	83255189	129794	10	*LOC107133137, LOC784458*
14	83255189	83280976	25788	11	.
14	83280976	83389009	108034	12	.
14	83389009	84030913	641905	10	*CHMP4C, DEPTOR, DSCC1, ENPP2, IMPA1, LOC100335514, LOC104974144, LOC107133138, LOC107133139, MIR2285N-6, SLC10A5, SNX16, TAF2, ZFAND1*
14	84030913	84196275	165363	8	*DEPTOR*
14	84196275	85007025	810751	2	*COL14A1, LOC104974149, LOC107133140, LOC107133141, MRPL13, MTBP, SNTB1*
15	14636	163292	148657	8	*LOC104974152*
15	163292	189786	26495	7	*LOC104974153*
15	189786	233002	43217	7	*LOC101909994, LOC509895, LOC784899*
15	233002	247904	14903	7	*LOC784899*
15	247904	290646	42743	6	*LOC100299084, LOC530292, LOC781133*
15	290646	542306	251661	4	*LOC107133142, LOC518869, LOC526715, LOC781246, LOC781264, LOC781286, LOC781326, LOC781366, LOC781406, LOC781447, LOC781518, LOC781636*
15	542306	700993	158688	2	*LOC781245, LOC781285, LOC781325, LOC790589, OR9G1*
15	700993	798516	97524	2	*LOC100139651, LOC781053, LOC781089, LOC781209*
15	798516	927750	129235	5	*LOC107133143, LOC517325, LOC531237, LOC618523, LOC788868, LOC788875, OR4C15*
15	927750	999765	72016	5	*LOC100139475, LOC530524, LOC532486*
15	999765	1064091	64327	5	*LOC786395*
15	1064091	1128925	64835	4	*LOC515790, LOC786445, LOC786467*
15	1128925	1219097	90173	4	*LOC508315, LOC786525, LOC787524, OR4C16*
15	1219097	1287849	68753	2	*LOC787524*
15	1287849	1342835	54987	2	*LOC100300262, LOC100300302*
15	1342835	1507825	164991	3	.
15	1507825	1537140	29316	3	*IZUMO1R*
15	1537140	1670784	133645	2	*GPR83, MRE11A*
15	2561559	2611609	50051	4	*GRIA4*
15	2611609	2630928	19320	4	*GRIA4*
15	2630928	2809624	178697	5	*GRIA4*
15	2809624	2865901	56278	4	.

15	2865901	2915278	49378	4	.
15	2915278	3014146	98869	3	.
15	3014146	3036600	22455	2	.
15	3036600	3134225	97626	3	.
15	3134225	3147569	13345	3	.
15	3147569	3216393	68825	2	.
15	4311001	4359127	48127	2	.
15	4523355	4548650	25296	2	*LOC104974160, PDGFD*
15	4548650	4592761	44112	3	*PDGFD*
15	4592761	4798875	206115	3	*DDI1, LOC104974158, PDGFD*
15	4798875	4896744	97870	4	*PDGFD*
15	4896744	5030200	133457	4	.
15	5030200	5078468	48269	3	.
15	5078468	5106564	28097	6	.
15	5106564	5413931	307368	4	*DYNC2H1*
15	5413931	5502085	88155	2	*DYNC2H1*
15	5502085	5594309	92225	2	*DYNC2H1, TRNAY-AUA*
15	5594309	5644722	50414	2	*DYNC2H1*
15	5644722	5822012	177291	2	*DCUN1D5, DYNC2H1*
15	6622738	6679986	57249	2	*BIRC2*
15	6694197	6803597	109401	4	*BIRC3, LOC100848148, LOC104974163, LOC104974165, LOC107133144, LOC783022, YAP1*
15	6803597	6915737	112141	6	*YAP1*
15	6915737	6937162	21426	7	*YAP1*
15	6937162	6991992	54831	8	*C15H11orf70, LOC107133145, YAP1*
15	6991992	7171690	179699	10	*C15H11orf70, CEP126, LOC101902649*
15	7171690	7195534	23845	9	*CEP126*
15	7195534	7251907	56374	8	*ANGPTL5*
15	7251907	7275497	23591	8	.
15	7275497	7391335	115839	8	.
15	7391335	7402768	11434	10	.
15	7402768	7447810	45043	8	.
15	7447810	7460094	12285	5	.
15	7460094	7468770	8677	3	.
15	7468770	7537576	68807	4	.
15	7537576	7597750	60175	5	*LOC787373*
15	7597750	7651811	54062	6	*TRPC6*
15	7651811	7750682	98872	6	*TRPC6*
15	7750682	7856066	105385	3	*TRPC6*
15	7856066	7875692	19627	4	.
15	7875692	7956395	80704	3	.
15	7956395	7997690	41296	4	*LOC787452*
15	7997690	8041421	43732	5	.
15	8217258	8282475	65218	2	*PGR*
15	8300166	8313410	13245	3	.
15	8313410	8379060	65651	4	*ARHGAP42*
15	8379060	8559453	180394	3	*ARHGAP42, LOC107133147*
15	8559453	8724877	165425	2	*ARHGAP42*
15	8724877	8783048	58172	5	.
15	8783048	8948707	165660	5	*LOC101903693*
15	8948707	8978430	29724	5	.
15	8978430	9105346	126917	4	*LOC782668*
15	9105346	9146354	41009	4	.
15	9146354	9162502	16149	2	.
15	9162502	9253461	90960	2	.
15	9253461	9322157	68697	2	.
15	9322157	9325130	2974	2	.
15	9325130	9352143	27014	4	.
15	9352143	9363622	11480	4	.
15	9363622	9437747	74126	4	.
15	9437747	9480106	42360	4	.
15	9480106	9496428	16323	3	.
15	9496428	9542150	45723	3	*CNTN5*
15	9689095	9751449	62355	3	*CNTN5*
15	9751449	9782462	31014	3	*CNTN5*
15	9782462	9788664	6203	2	*CNTN5*
15	9788664	9932903	144240	4	*CNTN5*
15	9932903	9945498	12596	3	*CNTN5*
15	9945498	9979898	34401	2	*CNTN5*
15	10187137	10296843	109707	2	.
15	10296843	10352428	55586	2	.
15	10388445	10504667	116223	2	.
15	10504667	10581564	76898	2	.
15	10581564	10661811	80248	2	.
15	10661811	10736157	74347	2	.
15	10736157	10861560	125404	3	.
15	10861560	10958768	97209	3	*LOC100138161*
15	10958768	11028247	69480	2	.
15	11452892	11475405	22514	2	.
15	11475405	11644433	169029	2	.
15	13456001	13576215	120215	2	.
15	13576215	13612776	36562	2	*TRNAW-CCA*
15	13612776	13681837	69062	3	.
15	13681837	13725630	43794	3	.
15	13725630	13885055	159426	3	*LOC100300167*
15	13885055	13965740	80686	2	.
15	13965740	13985195	19456	3	.

15	13985195	14051835	66641	3	.
15	14051835	14117419	65585	3	*LOC783497*
15	14117419	14210766	93348	2	*LOC101905762*
15	14210766	14312414	101649	2	*CCDC82, JRKL, MAML2, MIR1260B*
15	14568802	15321300	752499	3	*CEP57, FAM76B, LOC101905912, MAML2, MTMR2*
15	22694978	23094071	399094	2	*BCO2, C15H11orf1, C15H11orf52, C15H11orf57, CRYAB, DIXDC1, DLAT, HSPB2, IL18, LOC101903059, LOC101903125, LOC101906594, LOC104968480, LOC104974189, PIH1D2, PTS, SDHD, TEX12, TIMM8B*
15	23198666	23309471	110806	2	*LOC100847772*
15	23309471	23501646	192176	2	*LOC100847772*
15	23501646	23833546	331901	3	*LOC104974191, LOC107133153*
15	26967717	27099326	131610	4	.
15	30423392	30510276	86885	4	*ABCG4, CBL, CCDC153, NLRX1, PDZD3*
15	30510276	30869701	359426	5	*C1QTNF5, CBL, LOC101903835, MCAM, MFRP, RNF26, THY1, TRNAG-CCC, USP2*
15	30869701	31087909	218209	3	*MIR378B, PVRL1*
15	34249521	34351275	101755	3	*BSX, C15H11orf63, LOC104974229, LOC107133161*
15	34351275	34537981	186707	3	*CLMP, HSPA8, LOC107133162*
15	34537981	34696293	158313	3	*LOC107133163*
15	37495307	37655305	159999	3	*LOC104974235, TRNAW-CCA*
15	37655305	38055456	400152	4	*LOC104974236, LOC104974237*
15	38055456	38087339	31884	4	*INSC*
15	38087339	38280952	193614	4	*CALCB, INSC*
15	38280952	38282300	1349	3	.
15	38282300	38453064	170765	5	*CALCA, TRNAY-GUA*
15	38453064	38478909	25846	4	.
15	38478909	38788999	310091	3	*CALCB, CYP2R1, LOC784945, PDE3B*
15	38788999	38819963	30965	3	*PDE3B, TRNAC-GCA*
15	38819963	38972409	152447	4	*COPB1, LOC101905000, PDE3B, PSMA1*
15	38972409	39219097	246689	4	*COPB1, LOC104974239, LOC782705, RRAS2, SPON1*
15	39219097	39240866	21770	3	*SPON1*
15	39240866	39263524	22659	3	*SPON1*
15	39263524	39386073	122550	3	*MIR2284T, SPON1*
15	40010673	40021288	10616	2	*BTBD10*
15	40021288	40220738	199451	2	*ARNTL, BTBD10*
15	40220738	40235819	15082	2	.
15	40235819	40242932	7114	2	.
15	40242932	40534092	291161	2	*RASSF10, TEAD1*
15	44995186	44998444	3259	2	.
15	44998444	45179949	181506	2	*LMO1, RIC3*
15	45179949	45212167	32219	3	*RIC3*
15	45212167	45233004	20838	3	*LOC104974253, RIC3*
15	45233004	45310156	77153	4	*LOC104974253, LOC107133167, TUB*
15	45310156	45449101	138946	4	*EIF3F, LOC107133167, LOC782660, LOC782731, LOC782910, LOC785565, NLRP10, OR10A3, OR10A6*
15	45449101	45562110	113010	2	*LOC529308, LOC539064, OR5P3, OVCH2*
15	45562110	45607287	45178	2	*LOC617062, LOC783210, OVCH2*
15	45607287	45628443	21157	2	*LOC538744, OVCH2*
15	45628443	45668395	39953	2	*LOC782576, LOC783281, OVCH2*
15	46084774	46523739	438966	2	*NLRP14, RBMXL2, SYT9*
15	49848538	49904509	55972	2	*LOC100137850, LOC782254, OR51F2, OR51S1*
15	49904509	50002618	98110	3	*LOC100852273, LOC508595, LOC527918, LOC782373, LOC782428, LOC784379, LOC788815, OR51T1*
15	50002618	50066692	64075	3	*LOC100337195, LOC511823, LOC785207, LOC785238, LOC785407*
15	50066692	50136952	70261	2	*LOC100337265, LOC100847430, LOC100847455, LOC785049, OR52E2*
15	50136952	50181043	44092	2	*LOC617700, LOC785144*
15	50517660	50605658	87999	2	*LOC782960, LOC783446, LOC783488, LOC783529, LOC783558*
15	50605658	50632792	27135	2	*LOC783401*
15	50632792	50677785	44994	2	*LOC100848221, LOC530990, LOC783323, LOC783401*
15	50677785	50693905	16121	2	*LOC107131374*
15	50787692	50850242	62551	4	*LOC524658, LOC528515, LOC788674, LOC788692*
15	50850242	50960440	110199	6	*LOC100848377, LOC100848398, LOC107133172, LOC788703, LOC788722, LOC788731, LOC788750, LOC788763*
15	50960440	51032155	71716	2	*LOC504551, LOC506897, LOC782623, LOC782658, LOC782692, OR51A7*
15	52642370	52760299	117930	2	*IL18BP, LRRC51, NUMA1, RNF121*
15	52760299	52813813	53515	3	*ANAPC15, LAMTOR1, LRRC51, TOMT*
15	52813813	52885415	71603	2	*FOLR1, FOLR2, FOLR3, INPPL1, PHOX2A, TRNAG-CCC*
15	52885415	53227393	341979	2	*CLPB, LOC104974266, LOC104974267, MIR139, PDE2A*
15	53269943	53543723	273781	5	*ARAP1, ATG16L2, FCHSD2, STARD10*
15	53543723	53689333	145611	6	*FCHSD2*
15	53689333	53800683	111351	5	*LOC104974269, P2RY2*
15	53800683	53838935	38253	4	*P2RY6*
15	53838935	54009255	170321	5	*ARHGEF17, FAM168A, RELT*
15	54009255	54085744	76490	5	.
15	55604265	55880540	276276	2	*GDPD5, KLHL35, LOC101905131, MAP6, RPS3, SERPINH1*
15	57501392	57529882	28491	6	*B3GNT6*
15	57529882	57712553	182672	6	*CAPN5, MYO7A*
15	57712553	57895111	182559	6	*LOC786511, LOC786996*
15	57895111	58137771	242661	4	*ANO3*
15	58137771	58486397	348627	4	*ANO3, MUC15, SLC5A12*
15	58486397	58553800	67404	4	.
15	58553800	58625548	71749	2	.
15	58625548	58756742	131195	2	.
15	58756742	58945254	188513	2	*BBOX1, FIBIN, LOC104974280*
15	58945254	58986097	40844	2	*LOC104974279, LOC104974280*
15	59492081	60398586	906506	2	*KIF18A, METTL15, TRNAC-GCA*
15	60514152	60614145	99994	3	.
15	60614145	60649299	35155	4	.

15	60649299	60765431	116133	5	.
15	60765431	60772144	6714	3	.
15	62905024	63031312	126289	2	.
15	63096354	63285273	188920	2	*DCDC1, DNAJC24*
15	63285273	63428577	143305	4	*DNAJC24, ELP4, IMMP1L*
15	63428577	63468424	39848	2	*ELP4*
15	65782970	65863754	80785	3	*ABTB2, CAPRIN1, NAT10*
15	65863754	66011612	147859	2	*ABTB2*
15	66011612	66239371	227760	4	*ABTB2, CAT, ELF5, LOC104974297*
15	66239371	66289548	50178	6	.
15	66289548	66314351	24804	7	.
15	66314351	66433469	119119	3	*EHF, LOC100140140*
15	66492589	66570667	78079	2	*APIP, PDHX*
15	67016664	67301731	285068	4	*FJX1, PAMR1, SLC1A2*
15	67301731	67360846	59116	4	*TRIM44*
15	67360846	67459127	98282	5	*TRIM44*
15	67459127	67469527	10401	4	.
15	67469527	67575822	106296	3	*LDLRAD3, LOC104974302, LOC104974303*
15	68296993	68333207	36215	2	.
15	68333207	68416858	83652	3	.
15	68416858	68672236	255379	3	.
15	68672236	68719014	46779	2	.
15	68719014	68721815	2802	2	.
15	68721815	68970434	248620	3	.
15	68970434	69007980	37547	2	.
15	69050267	69155847	105581	3	.
15	69155847	69211436	55590	2	.
15	69211436	69357951	146516	4	*LOC617085*
15	69357951	69414086	56136	4	.
15	69414086	69574994	160909	5	.
15	69574994	69635298	60305	6	.
15	69635298	69684992	49695	5	.
15	70217332	70301067	83736	2	.
15	70301067	70413442	112376	3	.
15	70953910	71033477	79568	3	.
15	71033477	71139300	105824	3	.
15	71139300	71168246	28947	3	.
15	71502793	71643861	141069	2	*LRRC4C*
15	71643861	71648547	4687	2	*LRRC4C*
15	71648547	71836206	187660	3	*LOC104974309, LRRC4C*
15	71836206	71968450	132245	4	*LRRC4C*
15	71968450	72108708	140259	3	*LRRC4C*
15	72108708	72185684	76977	9	*LRRC4C*
15	72185684	72198480	12797	9	*LRRC4C*
15	72198480	72250100	51621	10	*LRRC4C*
15	72250100	72254368	4269	13	*LRRC4C*
15	72254368	72303509	49142	15	*LRRC4C*
15	72303509	72415617	112109	14	*LRRC4C*
15	72415617	72812971	397355	10	*LRRC4C*
15	72812971	72822012	9042	9	*LRRC4C, MSANTD3*
15	72822012	72877384	55373	4	*LRRC4C, MSANTD3*
15	72877384	72923858	46475	2	*LRRC4C*
15	72923858	73157900	234043	4	*LOC107133187, LRRC4C*
15	73157900	73175015	17116	4	.
15	73175015	73284840	109826	3	*LOC107133188*
15	73376693	73517398	140706	2	.
15	73517398	73625299	107902	3	.
15	73625299	73949859	324561	3	.
15	73981098	74027572	46475	2	.
15	74027572	74101940	74369	2	.
15	78102753	78212796	110044	2	*LOC100138331, LOC104970192, LOC107133184, LOC509025, LOC509617, LOC617614*
15	78212796	78401816	189021	2	*LOC100336492, LOC100848485, LOC104974327*
15	78867381	78966357	98977	4	*CELF1, KBTBD4, NDUFS3, PTPMT1*
15	78966357	79014661	48305	5	*C1QTNF4, FAM180B, MTCH2*
15	79014661	79082648	67988	4	*AGBL2, FNBP4*
15	79082648	79252657	170010	3	*FNBP4, LOC100848627, LOC104974328, LOC104974330, NUP160*
15	79993193	80043709	50517	4	*LOC521252, LOC789247, LOC789269*
15	80043709	80089785	46077	3	*LOC100140748, LOC528722, LOC789269, OR5D13*
15	80089785	80184812	95028	3	*LOC100140283, LOC511865, LOC520162, LOC787563, LOC789288, LOC789293, LOC789300, OR5I1*
15	83495928	83520040	24113	2	*LOC515045*
15	83520040	83580022	59983	3	*LOC100301403, LOC101907482, LOC509710*
15	83580022	83648435	68414	3	*LPXN, ZFP91*
15	83648435	83764513	116079	3	*CNTF, LOC518623, LOC787861, TRNAF-GAA, ZFP91*
15	83764513	83777255	12743	2	*LOC518623*
15	83917047	83999005	81959	3	*FAM111B, LOC100138922*
15	84112781	84181593	68813	2	*LOC107133206, LOC519282*
15	84181593	84345360	163768	3	*LOC100299686, LOC100848982, LOC107131345, LOC504200, LOC516439, LOC785818, LOC785899*
15	84345360	84593201	247842	4	*LOC101907407, LOC107133209, LOC505072, LOC512441, LOC514071, LOC514367, LOC530068, LOC616011, LOC788587, OR4D11, OR4D6, OR4D9, OR5A1, OSBP, PATL1, TRNAF-GAA, TRNAK-UUU, TRNAL-UAA, TRNAR-UCU, TRNAV-UAC*
15	84593201	84642437	49237	4	*LOC101907407, OR10V1*
15	84642437	84701574	59138	4	*LOC527077, STX3*
15	84701574	84798461	96888	3	*GIF, MRPL16, STX3, TCN1, TRNAG-CCC*
15	84798461	84856465	58005	4	*LOC101903724, LOC104970391, OOSP1*

15	84856465	85129664	273200	4	*LOC100849053, LOC101903724, LOC107133207, MIR6519, MS4A1, MS4A13, MS4A14, MS4A2, MS4A3, MS4A5, MS4A7*
15	85452457	85561261	108805	2	*LOC100335879, LOC104969826, LOC107133208*
16	1767	470502	468736	4	*LOC524282, LOC618608, LOC787541, LOC787560, LOC787592, LOC787605, LOC787620, LOC787642, LOC787667, LOC787745, LOC789376, OR5L1, TRNAR-UCU*
16	470502	1258146	787645	6	*ADORA1, ATP2B4, BTG2, CHI3L1, CHIT1, FMOD, LOC100139016, LOC100140718, LOC100847554, LOC101906460, LOC101906704, LOC107133210, LOC107133212, LOC107133214, MYBPH, MYOG, OPTC, PPFIA4, PRELP, TMEM183A*
16	1258146	1272655	14510	4	*ATP2B4*
16	1272655	2013054	740400	4	*ATP2B4, ETNK2, GOLT1A, KISS1, LAX1, LOC101907689, LOC101907913, LOC104974354, LOC107133215, PIK3C2B, PLEKHA6, PPP1R15B, REN, SNRPE, SOX13, ZC3H11A*
16	13671828	13749912	78085	3	.
16	13749912	13949666	199755	3	*LOC107133223, RGS18*
16	21651315	21920738	269424	2	*GPATCH2, SPATA17*
16	28610182	29173240	563059	4	*CNIH3, DNAH14, LOC107133226, LOC785251*
16	35761270	35791594	30325	2	*LOC104974409, LOC104974410*
16	35791594	36076798	285205	2	*BECN2, CHML, EXO1, LOC101907876, MAP1LC3C, WDR64*
16	36076798	36608105	531308	2	*FH, KMO, LOC100298153, LOC782999, RGS7*
16	36608105	36628161	20057	2	*RGS7*
16	36628161	36705878	77718	2	*RGS7*
16	36705878	38273516	1567639	2	*ATP1B1, BLZF1, CCDC181, DPT, F5, LOC100848690, LOC101908227, LOC104974416, LOC104974419, LOC104974420, LOC107133232, LOC107133233, LOC783740, NME7, RGS7, SELP, SLC19A2, TRNAC-ACA, TRNAC-GCA, TRNAG-CCC, TRNAY-GUA, XCL1, XCL2*
16	54884776	54958733	73958	4	*KAZN*
16	54958733	55011164	52432	4	*KAZN*
16	55011164	55411802	400639	7	*KAZN, PRDM2*
16	56115073	58259980	2144908	6	*AADACL3, ANKRD45, C16H1orf158, CACYBP, CENPL, DARS2, GPR52, KIAA0040, KLHL20, LOC100138041, LOC100299783, LOC100299866, LOC100336249, LOC100336653, LOC100848270, LOC100848315, LOC100848389, LOC101908155, LOC104974479, LOC104974493, LOC107131215, LOC107133244, LOC517884, LOC522065, LOC528132, LOC530538, LOC617709, LOC781733, LOC790218, MRPS14, PRDX6, RABGAP1L, RC3H1, SERPINC1, SLC9C2, TNN, TNR, ZBTB37*
16	61953278	62607812	654535	9	*ABL2, AXDND1, FAM163A, FAM20B, LOC101903932, LOC107133254, NPHS2, SOAT1, TDRD5, TOR3A, TRNAS-GGA*
16	62607812	63371862	764051	4	*ACBD6, CEP350, FAM163A, LHX4, QSOX1, TOR1AIP1, TOR1AIP2*
16	70586673	70868851	282179	3	*CENPF, PTPN14*
16	71378471	71986857	608387	2	*LOC100139281, LOC101909678, LOC104972797, LOC104972800, LOC104974513, PPP1R12B, PROX1, SMYD2, SYT2*
17	1395	587843	586449	4	*CPE, KLHL2, LOC100295562, LOC101902493, LOC104974555, LOC508314, LOC511436, LOC781637, LOC781676, LOC781712, MSMO1, TMEM192*
17	1805734	2131417	325684	3	*MAP9, NPY2R, TLL1*
17	2131417	2247074	115658	2	.
17	2247074	2484578	237505	2	.
17	4893111	5408428	515318	2	*FBXW7, LOC104974562, LOC107133272, TIGD4, TMEM154, TRNAY-GUA*
17	5408428	5942670	534243	4	*LOC785575, TRNAW-CCA*
17	5942670	6560919	618250	3	*BRAP, FAM160A1, GATB, MIR2404-1, PRSS48, SH3D19*
17	8342362	8662697	320336	6	.
17	8662697	8705526	42830	3	*TRNAC-GCA*
17	8705526	8859112	153587	2	.
17	8859112	8870332	11221	7	.
17	8870332	8890825	20494	13	.
17	8890825	9017455	126631	15	*LOC100336978, LOC523431*
17	9017455	9426429	408975	2	*LOC101905833, LOC104974568*
17	9426429	9761623	335195	12	*LOC104974568, LOC104974569*
17	9761623	9804448	42826	10	*LOC104974569, NR3C2*
17	9804448	10822405	1017958	2	*ARHGAP10, EDNRA, LOC100297399, LOC100847708, LOC101906279, LOC107133274, NR3C2, PRMT9, TMEM184C, TRNAC-ACA*
17	10822405	10954800	132396	4	*EDNRA*
17	11304404	11454829	150426	2	*LOC101906544*
17	11454829	11606876	152048	2	*TTC29*
17	11836924	11939871	102948	3	*POU4F2*
17	11939871	12064974	125104	5	*SLC10A7*
17	12064974	12197387	132414	4	*SLC10A7*
17	12197387	12217654	20268	3	.
17	12217654	12341724	124071	2	*LOC101906679, LOC107133273*
17	13170454	13335712	165259	2	*LOC100847839, LOC101907935*
17	14667857	15140145	472289	2	*GAB1, LOC101906545, LOC107133279, TRNAK-UUU, USP38*
17	16072619	16305262	232644	3	*INPP4B*
17	16399789	16995936	596148	2	*IL15, ZNF330*
17	17170033	17456583	286551	3	*RNF150, TBC1D9, TRNAR-CCU*
17	18549027	18744651	195625	4	*LOC100848703, LOC101907619, NAA15, RAB33B, SETD7*
17	18744651	18832024	87374	4	*LOC101907758, LOC101907835, LOC107133275, MGARP, NAA15, NDUFC1, TRNAE-CUC*
17	25102772	25990366	887595	8	*LOC100336402, LOC107131277, LOC786811, PCDH10, TRNAC-GCA*
17	25990366	26051601	61236	9	.
17	26051601	26338823	287223	6	.
17	26338823	27276113	937291	4	.
17	27276113	27531537	255425	5	*LOC104974594*
17	27531537	27989253	457717	4	*LOC104974594*
17	27989253	28490828	501576	8	.
17	28490828	28813313	322486	8	*LOC785705*
17	28813313	29011304	197992	10	*LOC782706*
17	29011304	29184303	173000	5	.

17	29184303	30592020	1407718	2	*ABHD18, C17H4orf33, HSPA4L, INTU, JADE1, LARP1B, LOC100138580, LOC100140839, LOC100336168, LOC101902922, LOC101903013, LOC101903064, LOC101904901, LOC107133276, LOC107133277, LOC107133282, LOC107133283, LOC781255, LOC781318, LOC781528, MFSD8, PGRMC2, PLK4, SCLT1, SLC25A31*
17	30592020	30811918	219899	4	*LOC101903780*
17	30811918	30839331	27414	2	.
17	30839331	30874321	34991	2	.
17	31727457	32321406	593950	2	*LOC104974601, LOC104974602, LOC107133286, LOC781055*
17	32321406	32405749	84344	4	*LOC781055*
17	32405749	32655550	249802	5	.
17	32655550	32672983	17434	6	.
17	32672983	33234404	561422	6	*FAT4*
17	33234404	33433568	199165	2	.
17	33433568	33478164	44597	2	.
17	33478164	33795557	317394	2	*ANKRD50*
17	34201391	34318319	116929	2	.
17	34318319	35528384	1210066	3	*BBS12, CETN4, FGF2, LOC104974605, LOC104974607, LOC104974608, LOC107133284, NUDT6, SPATA5, SPRY1*
17	35528384	35753987	225604	10	*IL2, IL21*
17	35753987	36393410	639424	9	*ADAD1, KIAA1109, LOC100300040, LOC100336709, LOC100336794, LOC107131163, LOC107133285, LOC781972, LOC789301, TRNAW-CCA*
17	36393410	36450587	57178	6	*LOC107131163*
17	36450587	36638846	188260	3	*LOC107131163, LOC782754*
17	36638846	37058800	419955	2	*LOC101909438, LOC101909472, TRPC3*
17	37058800	37129898	71099	2	.
17	39386465	39986037	599573	5	*LOC783124*
17	39986037	40116898	130862	2	*ULBP3*
17	40424224	41590744	1166521	3	*C17H4orf45, C17H4orf46, ETFDH, FNIP2, LOC101904878, LOC101904929, LOC107133287, LOC107133289, PPID, RAPGEF2, RXFP1, TNIP3*
17	41630435	41943543	313109	7	*FAM198B, LOC107133288, TMEM144*
17	41943543	42350898	407356	4	*LOC101903911*
17	42407254	42738964	331711	4	*TRNAG-CCC*
17	42738964	43251347	512384	5	*GLRB, GRIA2, MIR2321, TRNAC-GCA*
17	43251347	43635581	384235	5	*LOC104974614, LOC107133291, PDGFC*
17	43635581	43933300	297720	2	*LOC107133291*
17	43933300	44231724	298425	5	.
17	44231724	44396481	164758	5	.
17	44396481	44563542	167062	4	*ASIC5, CTSO, TDO2*
17	44563542	44620523	56982	5	*ASIC5*
17	44620523	44645887	25365	6	*GUCY1B3*
17	44645887	44853002	207116	5	*GUCY1A3, GUCY1B3, LOC101904168*
17	44853002	44966876	113875	4	*LOC101904168*
17	46717178	46743055	25878	2	.
17	46743055	47206627	463573	4	*ADGRD1, LOC104974620*
17	47206627	47417244	210618	3	*ADGRD1, RAN, TRNAW-CCA*
17	49136770	49229676	92907	2	*TMEM132D*
17	49229676	49396026	166351	2	*GLT1D1, LOC101907645*
17	62008520	62185923	177404	2	*TRNAG-CCC*
18	13832408	15288711	1456304	2	*ACSF3, ANKRD11, APRT, C18H16orf87, CBFA2T3, CDH15, CDK10, CDT1, CENPBD1, CHMP1A, CPNE7, CTU2, CYBA, DBNDD1, DEF8, DPEP1, FANCA, GALNS, GAS8, IL17C, LOC100296324, LOC101904595, LOC104974755, LOC104974758, LOC104974760, LOC104974835, LOC107131144, LOC517901, LOC532875, MC1R, MIR2327, MVD, MYLK3, ORC6, PABPN1L, PIEZO1, RNF166, RPL13, SHCBP1, SLC22A31, SNAI3, SPATA2L, SPATA33, SPG7, SPIRE2, TCF25, TRAPPC2L, TUBB3, VPS35, VPS9D1, ZC3H18, ZFPM1, ZNF276*
18	15647931	15935247	287317	3	*ITFG1, NETO2*
18	16274612	18184433	1909822	2	*ABCC11, ABCC12, C18H16orf78, CBLN1, LOC104970029, LOC104974836, LOC522437, LOC533093, LONP2, N4BP1, SIAH1, ZNF423*
18	27272163	27369681	97519	2	.
18	27369681	27864967	495287	2	.
18	27864967	29389709	1524743	5	*LOC781392*
18	29639253	29660159	20907	2	*CDH8*
18	29660159	30072683	412525	2	*CDH8*
18	47287636	47658413	370778	4	*LOC100848226, LOC101907195, LOC101907883, LOC107131425, LOC613546, ZFP14, ZFP82, ZNF382, ZNF461, ZNF566, ZNF585A, ZNF793, ZNF829*
18	47658413	48152056	493644	3	*LOC101901900, LOC101908181, LOC107131422, LOC509804, LOC519309, LOC524350, LOC787074, LOC787397, SIPA1L3, TRNAE-UUC, ZFP30, ZNF345, ZNF383, ZNF420, ZNF527, ZNF569, ZNF570*
18	48152056	48164032	11977	3	*SIPA1L3*
18	48164032	48429406	265375	3	*DPF1, LOC101902440, SIPA1L3*
18	48429406	48530477	101072	2	*C18H19orf33, PPP1R14A, SPINT2, YIF1B*
18	52248566	52578498	329933	6	*CADM4, ETHE1, IRGC, IRGQ, KCNN4, LOC100337113, LOC101909226, LOC104974883, LOC107131429, LOC506634, LOC512005, LOC526915, LOC616722, LOC616860, LYPD5, PHLDB3, PLAUR, SMG9, SRRM5, XRCC1, ZNF428, ZNF575, ZNF576*
18	52578498	52604599	26102	4	.
18	52604599	53080991	476393	5	*CEACAM20, IGSF23, LOC101903309, LOC104974888, LOC107131431, LOC107131433, LOC107131442, LOC526865, LOC538686, ZNF180, ZNF226, ZNF227, ZNF233, ZNF235, ZNF283, ZNF285, ZNF404, ZNF45*
18	57902581	57935903	33323	2	*LOC104974923, LOC107131444*
18	57935903	58245134	309232	9	*C18H19orf84, CLDND2, ETFB, IGLON5, LIM2, LOC100297056, LOC100300478, LOC104974925, LOC104974926, LOC107131445, LOC107131446, LOC107133350, LOC506364, LOC512863, LOC614923, LOC618268, LOC786291, LOC789748, NKG7, SIGLEC10, TRNAG-CCC, VSIG10L, ZNF175*

18	58245134	58479771	234638	5	*HAS1, LOC101907648, LOC101907942, LOC107131224, LOC107131449, LOC787309, LOC787554, MIR125A, MIR99B, MIRLET7E, ZNF350, ZNF432, ZNF613, ZNF614*
18	58479771	58486961	7191	2	*LOC787309*
18	59941353	60132763	191411	3	*LOC100299712, LOC100848077, LOC101907570, LOC101907718, LOC788019*
18	60372843	60519933	147091	2	*LOC100140226, LOC101903915, LOC107131455, LOC788150, LOC788599*
18	60519933	60810520	290588	4	*LOC100138666, LOC100139360, LOC101904879, LOC101905467, LOC101905616, LOC101909507, LOC104974934, LOC104974935, LOC104974936, LOC107131392, LOC107131456, LOC107131457, LOC531059, LOC788599, LOC788871, ZNF677, ZNF729*
18	60810520	60943782	133263	20	*LOC104974937, LOC104974938, LOC104976942, LOC107131458, LOC616720, LOC618289, LOC782961, LOC785408, LOC786224*
18	60943782	61024783	81002	23	*LOC101906283, LOC101906367, LOC101907390, LOC104968479, LOC788268, LOC788928*
18	61024783	61093395	68613	21	*LOC101906367, LOC101907041, LOC101909040, LOC104970711, LOC107133353, LOC618456*
18	61093395	61172232	78838	5	*LOC100336208, LOC100849068, LOC101902722, LOC104976972*
18	61512254	61621892	109639	2	*LOC504704, LOC506868, LOC515600, LOC615600, LOC784517, MGC157082*
18	61621892	61720911	99020	8	*LOC101904080, LOC101904151, LOC511982, LOC513941, LOC527385, LOC784517, LOC785919, MGC157082*
18	61720911	61763855	42945	8	*LOC785919*
18	61763855	61996635	232781	10	*LOC100337097, LOC101907900, LOC107131285, LOC510904, LOC517090, LOC528332, LOC528568, LOC617141, LOC618873, MGC138914*
18	61996635	62223881	227247	5	*LOC100300449, LOC100300531, LOC100336759, LOC100848932, LOC510904, LOC514552, LOC518964, LOC525820, LOC618662*
18	66159699	66344542	184844	10	*A1BG, CHMP2A, LOC100139764, LOC100847831, LOC100852286, LOC101903385, LOC101903649, LOC107133357, LOC107133358, MZF1, RPS5, SLC27A5, TRIM28, UBE2M, ZBTB45, ZNF132, ZNF324, ZNF446, ZNF584, ZSCAN22*
19	2147	234729	232583	7	*LOC107131482, LOC107131483, LOC524702, LOC531039, LOC788149, LOC789077*
19	234729	350370	115642	6	*LOC107131484, LOC107131485*
19	350370	977239	626870	6	*CA10, LOC101907572*
19	977239	1006675	29437	5	*CA10*
19	1006675	1209662	202988	4	*CA10*
19	1209662	1215800	6139	6	*CA10*
19	1215800	1242159	26360	6	*CA10, TRNAS-GGA*
19	1242159	1438254	196096	9	*CA10*
19	1438254	1605449	167196	6	*CA10*
19	1605449	1663957	58509	7	.
19	1663957	1860926	196970	8	.
19	1860926	1968160	107235	6	*LOC100138219*
19	1968160	2067688	99529	4	.
19	2067688	2258739	191052	4	.
19	2258739	2295465	36727	5	.
19	2295465	2370243	74779	3	.
19	2370243	2617188	246946	5	*LOC785473*
19	2617188	2678867	61680	3	.
19	2678867	2907092	228226	6	.
19	2907092	2935868	28777	6	.
19	2935868	2975907	40040	7	.
19	2975907	3200733	224827	6	.
19	3200733	3221986	21254	7	.
19	3221986	3487354	265369	5	*LOC100138652, LOC100141253, LOC100852304*
19	3487354	3524717	37364	5	.
19	3524717	3695944	171228	2	.
19	3695944	3842129	146186	4	.
19	3842129	4335161	493033	4	*KIF2B*
19	4335161	4658226	323066	4	*LOC790351, LOC790354*
19	5527395	5634182	106788	3	*STXBP4*
19	5634182	5783537	149356	2	*HLF, LOC781218*
19	5941227	6043434	102208	2	.
19	6043434	6223690	180257	3	*LOC100139255*
19	7707386	8199646	492261	2	*C19H17orf67, COIL, DGKE, LOC101904010, LOC107131487, LOC784678, NOG, SCPEP1, TRIM25*
19	8942697	9405202	462506	3	*CUEDC1, DYNLL2, LOC101905090, LOC104974989, LOC107131488, LOC508468, LOC512973, LOC514828, LOC788693, LOC788704, MRPS23, OR4D2, SRSF1, VEZF1*
19	9928248	10814694	886447	2	*GDPD1, LOC100847304, LOC101906048, LOC107131490, LOC539693, MGC137055, MIR301A, MIR454, PPM1E, PRR11, RAD51C, SKA2, SMG8, TEX14, TRIM37, TRNAE-UUC, YPEL2*
19	10814694	11578231	763538	2	*CLTC, DHX40, INTS2, LOC101907677, LOC104968960, LOC104968971, LOC107131490, LOC107131491, MED13, MIR21, PTRH2, RNFT1, RPS6KB1, TUBD1, VMP1*
19	12683013	12953193	270181	3	*APPBP2, C19H17orf64, LOC100295130, LOC101905169, LOC107131501, PPM1D*
19	12953193	13495359	542167	3	*CA4, DHRS11, GGNBP2, LOC101903622, LOC101905618, LOC104970422, LOC107131496, LOC783641, MRM1, MYO19, PIGW, USP32, ZNHIT3*
19	17984796	18336722	351927	2	*C19H17orf75, CDK5R1, LOC101903704, MYO1D, PSMD11, RHBDL3, TRNAQ-UUG, ZNF207*
19	18336722	18798317	461596	4	*ADAP2, ATAD5, COPRS, CRLF3, LOC101902800, LOC107131499, MIR2332, RHOT1, RNF135, SUZ12, TEFM, UTP6*
19	18798317	19059067	260751	4	*LOC107131500, MIR193A, MIR2333, MIR365-2, RAB11FIP4, TRNAT-CGU*
19	19059067	19255530	196464	3	*EVI2A, EVI2B, LOC101903070, NF1, OMG*
19	19639375	19941401	302027	5	*KSR1, LGALS9, LOC100848006, LOC101904699, LOC101904856, LOC104976580, LOC785307, NOS2, TRNAR-CCU*
19	19941401	19995708	54308	4	*LOC783508, LOC785589, RAET1L*

19	23623512	24897425	1273914	2	*CLUH, DPH1, HIC1, LOC100336161, LOC101901928, LOC101902679, LOC101906154, LOC101906737, LOC508307, LOC508980, LOC509525, LOC509526, LOC511509, LOC515540, LOC517569, LOC520835, LOC522582, LOC523547, LOC526294, LOC532238, LOC538966, LOC540082, LOC615901, LOC617122, LOC618112, LOC618124, LOC618593, LOC786951, LOC787076, METTL16, MIR132, MIR212, MIR2337, MNT, OR1D5, OR1E1, OR1G1, OR3A1, OVCA2, PAFAH1B1, RAP1GAP2, RPA1, RTN4RL1, SGSM2, SMG6, SRR, TSR1*
19	45202558	45359898	157341	2	*ADAM11, CCDC43, DBF4B, GJC1, MEIOC*
19	45359898	45373471	13574	2	*GJC1*
19	45373471	45797855	424385	2	*ACBD4, C1QL1, CCDC103, DCAKD, EFTUD2, FMNL1, GFAP, GJC1, HEXIM1, HEXIM2, HIGD1B, KIF18B, LOC101903790, LOC104975097, LOC104975099, LOC614821, MAP3K14, MIR2343, NMT1, PLCD3, SPATA32*
19	52356802	53209634	852833	8	*AATK, BAIAP2, CHMP6, ENDOV, LOC101902772, LOC509283, LOC512869, MIR338, NPTX1, RPTOR, TRNAG-CCC*
19	53209634	53920430	710797	4	*CARD14, CBX2, CBX4, CBX8, CCDC40, EIF4A3, ENPP7, GAA, LOC101907000, LOC101907247, LOC512869, RBFOX3, SGSH, SLC26A11, TBC1D16*
19	60980442	61386245	405804	2	*LOC104975159*
19	61386245	61518606	132362	4	*KCNJ16, KCNJ2, LOC104975162*
19	61518606	61664949	146344	2	*LOC100848333, LOC107131547*
19	61664949	62170451	505503	2	*ABCA5, LOC104975164, MAP2K6*
19	62170451	62268200	97750	12	*ABCA10, ABCA5, ABCA6, LOC100848794*
19	62268200	62466977	198778	10	*ABCA6, ABCA9, LOC107131548, MGC134105*
20	1649	136642	134994	9	*LOC100300814, LOC100300861, LOC107133371, LOC614697, LOC618225, LOC784595*
20	136642	567257	430616	7	*LOC784724, MIR103-1, MIR218-2, PANK3, SLIT3, SPZ1*
20	5855837	6286905	431069	2	*LOC785458, MIR584-6*
20	11055524	11300934	245411	2	*LOC101902212*
20	11300934	11608135	307202	2	*PIK3R1*
20	15112617	15287906	175290	2	*LOC107131560, RNF180*
20	15287906	15536194	248289	2	*MIR320A-2, RNF180*
20	15536194	15570780	34587	3	.
20	15570780	15741286	170507	3	*HTR1A*
20	15741286	15787696	46411	3	.
20	15787696	15828064	40369	2	.
20	15828064	16018176	190113	2	*LOC104975226*
20	17565117	17832309	267193	2	*LOC104975232, LOC107131561, LOC781741, TRNAG-CCC*
20	17832309	18367785	535477	3	*LOC100850100, LOC101907741, LOC104975230, LOC104975233, LOC107131409, MIR2889, NDUFAF2, ZSWIM6*
20	18438071	19483000	1044930	2	*DEPDC1B, ELOVL7, ERCC8, LOC100848933, LOC101903569, LOC104975237, LOC107131563, LOC107131564, NDUFAF2, PDE4D*
20	27418100	27971314	553215	3	*ISL1*
20	28685366	29009151	323786	3	*EMB, LOC104975257, LOC104975258, LOC785429*
20	32031953	32204927	172975	2	*GHR*
20	32204927	32459668	254742	5	*LOC104975266, LOC104975269*
20	32459668	32654935	195268	8	.
20	32654935	32834649	179715	7	*FBXO4, OXCT1*
20	32834649	33238498	403850	3	*OXCT1, PLCXD3, TRNAY-GUA*
20	41068096	41207419	139324	2	*LOC104975283, NPR3*
20	41207419	41244125	36707	2	.
20	41244125	41600868	356744	2	*MTMR12, SUB1, TRNAG-CCC, ZFR*
20	41600868	41886874	286007	2	*GOLPH3, LOC107131574, LOC614117, PDZD2, TRNAW-CCA*
20	46908310	47592275	683966	2	*CDH9*
20	47592275	48062009	469735	2	*LOC104975285, TRNAC-ACA, TRNAS-GGA*
20	48410738	48725491	314754	2	.
20	48725491	48990997	265507	2	*TRNAF-AAA*
20	48990997	49596686	605690	6	*CDH10, LOC100138662, LOC786112*
20	49596686	49708820	112135	4	*LOC101904270*
20	49708820	49734301	25482	3	*LOC101904270*
20	49734301	49879328	145028	3	.
20	49879328	49912827	33500	2	.
20	50495201	51167558	672358	2	*LOC100137772, TRNAC-GCA*
20	51844216	52189222	345007	5	*TRNAC-GCA*
20	52189222	52440810	251589	4	.
20	52440810	52783961	343152	2	.
20	52831099	52947521	116423	2	*TRNAC-GCA*
20	52947521	53226746	279226	2	*LOC781924*
20	53226746	53317918	91173	4	.
20	53656285	53759278	102994	2	*CDH18*
20	53759278	53899669	140392	2	*CDH18*
20	53899669	54253902	354234	2	*CDH18*
20	54253902	54284742	30841	2	.
20	54284742	54747417	462676	2	.
20	54747417	54968067	220651	3	*LOC781508*
20	55084351	55407826	323476	2	.
20	55407826	55694535	286710	3	*LOC101905359, LOC784462*
20	55694535	55892478	197944	2	*LOC101905359, LOC516790*
20	55964327	56159139	194813	2	*LOC100849014, LOC107131579*
20	56159139	56705905	546767	7	*BASP1, LOC100849043, MYO10*
20	56705905	56912867	206963	8	*MYO10*
20	56912867	57453246	540380	6	*FAM134B, MARCH11, TRNAC-ACA, ZNF622*
20	58368032	59487392	1119361	2	*ANKH, DNAH5, FAM105A, LOC104969091, LOC107131578, LOC107131580, LOC107131581, OTULIN, TRIO, TRNAC-ACA, TRNAG-CCC, TRNAG-UCC*
20	59612259	59666404	54146	6	*DNAH5*
20	59666404	59980110	313707	4	*DNAH5*
20	59980110	60144977	164868	3	.
20	60144977	60458957	313981	4	.
20	60458957	60530815	71859	5	*LOC101902130*

20	60530815	60566284	35470	5	.
20	60566284	60740238	173955	6	.
20	60740238	60965332	225095	5	.
20	60965332	61030254	64923	3	.
20	61030254	61155431	125178	3	*LOC781723*
20	64218498	64327876	109379	2	*SEMA5A*
20	64327876	64439336	111461	5	*SEMA5A*
20	64439336	64949906	510571	4	*LOC104975291, SEMA5A, TRNAC-GCA*
20	64949906	65245654	295749	3	.
20	65245654	65736415	490762	2	*C20H5orf49, FASTKD3, MTRR*
21	3715289	3923973	208685	2	.
21	3923973	3951064	27092	2	.
21	4133215	5427363	1294149	4	*GABRA5, GABRB3, GABRG3, LOC783909, LOC789997, VIMP*
21	19934161	20172946	238786	6	*AEN, DET1, ISG20, LOC101903487, LOC101903572, LOC107131588, LOC107131596, LOC522763, LOC616063, LOC781624, LOC781745, MIR1179, MIR7-1, MRPS11*
21	20345887	20412002	66116	4	*LOC100301248, LOC107133375*
21	20412002	20503861	91860	6	*LOC101906218, LOC107131597, LOC508153, LOC520626, LOC522540, LOC782682*
21	20503861	20628594	124734	7	*LOC100298625, LOC100298937, LOC100336774, LOC100848921, LOC508153, LOC526148, LOC526149, LOC613992*
21	20628594	20638709	10116	5	*LOC100298937, LOC100336807*
21	34781428	35665666	884239	2	*CCDC33, CYP11A1, GZMB, ISLR, ISLR2, LOC100139881, LOC101903457, LOC104969313, LOC104969321, LOC104969340, LOC104975377, LOC107131612, LOC505326, LOC505658, LOC508646, LOC508858, LOC509956, LOC540321, LOC617313, LOC618420, LOC786126, LOC788565, LOC788601, LOC788612, LOXL1, PML, STOML1, STRA6, STXBP6*
21	54259170	54516492	257323	2	*LOC101905447*
22	3322	74405	71084	13	*LOC528613, LOC787303, LOC787323, LOC787350, LOC787369, LOC787388*
22	74405	268600	194196	12	*LOC100336401, MRPS24, URGCP*
22	268600	426619	158020	11	*DBNL, LOC107131641, LOC107131642, LOC107131643, PGAM2, UBE2D4*
22	426619	652885	226267	13	*LOC107131640, VOPP1*
22	652885	768616	115732	9	*LANCL2, VOPP1*
22	768616	771967	3352	8	.
22	771967	1252901	480935	7	*EGFR*
22	2363503	2611085	247583	6	.
22	2611085	2644499	33415	4	.
22	2644499	2908080	263582	5	*AZI2, CMC1, ZCWPW2*
22	2908080	2953514	45435	3	*ZCWPW2*
22	2953514	3042272	88759	2	*ZCWPW2*
22	3042272	3237872	195601	2	*LOC100336827, LOC104975474*
22	7598600	7750191	151592	4	*CRTAP, FBXL2, SUSD5*
22	7750191	8265974	515784	2	*CLASP2, FBXL2, LOC101908214, LOC107131651, LOC785477, PDCD6IP, UBP1*
22	9674277	9915148	240872	4	*ARPP21, MIR128-2*
22	9915148	10586146	670999	8	*LOC100848011, LOC104975489, LOC107131652, LRRFIP2, MLH1, STAC*
22	10586146	12623547	2037402	2	*ACAA1, ACVR2B, C22H3orf35, CSRNP1, CTDSPL, DCLK3, DLEC1, EPM2AIP1, EXOG, GOLGA4, GORASP1, ITGA9, LOC101902747, LOC104975491, LOC104975492, LOC104976677, LOC107131653, LOC107131654, LOC107131655, MIR2367, MIR26A-1, MIR26C, MLH1, MYD88, OXSR1, PLCD1, SCN10A, SCN11A, SCN5A, SLC22A13, SLC22A14, TRANK1, TTC21A, VILL, WDR48, XIRP1, XYLB*
22	13738652	13804799	66148	2	.
22	13804799	14058262	253464	4	*CTNNB1, LOC104975498, ULK4*
22	14058262	14136802	78541	3	*ULK4*
22	14136802	14212799	75998	5	*ULK4*
22	14212799	14218615	5817	4	*ULK4*
22	14218615	14287814	69200	6	*ULK4*
22	14287814	14300245	12432	5	*ULK4*
22	14300245	14451203	150959	4	*ULK4*
22	14451203	14463775	12573	6	*ULK4*
22	14463775	15045625	581851	9	*ABHD5, LOC104975501, LOC107131657, LOC539893, LOC784464, MIR138-1, ULK4*
22	15045625	15417962	372338	4	*CCK, LOC107131659, LYZL4, SEC22C, TRAK1, ULK4, VIPR1*
22	15417962	15484840	66879	4	*NKTR, SS18L2*
22	15484840	15703484	218645	2	*ACKR2, CCDC13, CYP8B1, HHATL, HIGD1A, KLHL40, LOC101905008, LOC101905143, LOC107133382, NKTR, TRNAG-CCC, ZBTB47*
22	16224164	16462998	238835	4	*LOC101902415, LOC101902478, LOC101905720, LOC782599, TCAIM, TOPAZ1, ZNF445*
22	16462998	16564727	101730	3	*LOC107131645, LOC107131660, ZKSCAN7, ZNF197, ZNF445, ZNF852*
22	16564727	17183754	619028	4	*ARPC4, BRK1, BRPF1, CAMK1, CIDEC, CPNE9, CRELD1, EMC3, FANCD2, FANCD2OS, IL17RC, IL17RE, IRAK2, JAGN1, KIAA1143, KIF15, LOC100139192, LOC101905925, LOC101906342, LOC101906632, LOC104975513, MTMR14, OGG1, PRRT3, RPUSD3, TADA3, TATDN2, TTLL3, VHL, ZNF197, ZNF35, ZNF501, ZNF502*
22	18233976	18523669	289694	2	.
22	18523669	18661254	137586	2	*LOC104975517*
22	18681583	18917893	236311	3	*GRM7, LOC100848430, LOC107131663, LOC107131664*
22	20649816	20769316	119501	2	.
22	20769316	21017031	247716	2	.
22	26073690	26137576	63887	2	.
22	26659289	26827642	168354	5	*LOC107131673*
22	26827642	26945594	117953	7	.
22	26945594	27266733	321140	5	.
22	27266733	27339509	72777	5	.
22	27698000	28243684	545685	3	.
22	28243684	28480278	236595	2	*LOC104975536, PDZRN3*
22	29736780	30087423	350644	3	.
22	33408973	34131131	722159	2	*FAM19A1, LOC101904222, SUCLG2*
22	34131131	34405118	273988	5	*SUCLG2*
22	38164998	38453092	288095	3	*LOC107131683, SYNPR*
22	38453092	39281442	828351	2	*C22H3orf14, CADPS, FEZF2, LOC101907894*

22	40426318	40486781	60464	6	*PTPRG*
22	40486781	40592853	106073	5	*PTPRG*
22	40592853	40596824	3972	4	.
22	40596824	40627043	30220	3	.
22	40627043	40758848	131806	2	*FHIT*
22	40758848	41122513	363666	4	*FHIT*
22	41122513	41219530	97018	4	*FHIT*
22	44798294	45660995	862702	2	*ARHGEF3, CCDC66, ERC2, FAM208A, LOC104975557, MIR2371, TRNAG-UCC*
22	52512338	52911635	399298	3	*CSPG5, DHX30, ELP6, MAP4, SCAP, SMARCC1*
22	52911635	53013368	101734	3	*PTPN23, SCAP*
22	53013368	53345164	331797	3	*CCDC12, KIF9, KLHL18, LOC101906318, NBEAL2, NGP, NRADD, PTPN23, SETD2*
22	53345164	53374549	29386	2	*PTH1R*
22	60781533	61050040	268508	2	*LOC101905309, MCM2, PODXL2, TPRA1*
22	61050040	61320381	270342	4	*CHCHD6, PLXNA1*
22	61320381	61679250	358870	3	*ALDH1L1, C22H3orf22, CFAP100, CHST13, KLF15, LOC101905897, LOC104975617, LOC107131704, LOC107131705, LOC107131706, SLC41A3, TXNRD3, UROC1, ZXDC*
23	807	117408	116602	2	.
23	117408	330195	212788	3	*KHDRBS2*
23	330195	569693	239499	4	*KHDRBS2*
23	569693	661238	91546	4	*KHDRBS2*
23	661238	746752	85515	4	*KHDRBS2*
23	746752	850285	103534	4	*KHDRBS2*
23	850285	902787	52503	2	*KHDRBS2*
23	2289786	2690660	400875	4	*LOC100138918, PRIM2, TRNAC-ACA*
23	2690660	2742116	51457	4	*PRIM2*
23	2742116	2876809	134694	4	*PRIM2*
23	9866316	11055108	1188793	2	*BRPF3, C23H6orf222, C23H6orf89, CDKN1A, CPNE5, FGD2, KCTD20, LOC101907363, LOC101907794, LOC104975639, LOC104975643, LOC107131716, LOC520334, LOC618881, LOC790266, MAPK13, MAPK14, MTCH1, PI16, PNPLA1, PPIL1, PXT1, RAB44, RPS4Y1, SLC26A8, SRSF3, STK38, TRNAE-UUC, TRNAG-CCC*
23	11055108	11404700	349593	3	*CCDC167, CMTR1, LOC522610, PIM1, RNF8, TBC1D22B, TMEM217*
23	12116301	12812940	696640	3	*BTBD9, DNAH8, GLO1, LOC101904084, LOC781915*
23	12995423	13534721	539299	2	*KCNK16, KCNK17, KCNK5, LOC100139627, LOC104968668, LOC107131717, MIR2377, SAYSD1*
23	20312066	21147506	835441	3	*ADGRF2, ADGRF4, CD2AP, LOC100296156, LOC107131722, OPN5, PTCHD4, TNFRSF21*
23	21147506	21245035	97530	12	.
23	21245035	21370784	125750	12	.
23	21370784	21489661	118878	9	.
23	21489661	22187500	697840	3	*C23H6orf141, CENPQ, CRISP2, GLYATL3, LOC785693, MUT, RHAG*
23	22886516	23099335	212820	4	*TFAP2D*
23	23099335	23224007	124673	3	*TFAP2B*
23	24039816	24107037	67222	3	*PKHD1*
23	24107037	24124395	17359	2	*PKHD1*
23	24124395	24403162	278768	2	*IL17A, MIR133B, MIR206, PKHD1, TRNAG-CCC*
23	24418110	24916401	498292	3	*EFHC1, GSTA2, IL17F, LOC100138641, LOC101906535, LOC107131727, LOC107131728, MCM3, PAQR8, TMEM14A, TRAM2*
23	26892752	27138894	246143	2	*AGER, AGPAT1, ATF6B, EGFL8, FKBPL, LOC100138858, LOC101903877, LOC107131209, LOC615223, LOC786065, NOTCH4, PBX2, PPT2, PRRT1, RNF5*
23	27138894	27193097	54204	2	*LOC107131127, LOC107131209, TNXB*
23	27193097	27212778	19682	2	*TNXB*
23	27212778	27246043	33266	4	*CYP21, LOC101904057, LOC101904187, LOC617696, LOC781663, TNXB*
23	27246043	27261793	15751	6	*LOC617696*
23	27261793	27727673	465881	8	*ABHD16A, AIF1, APOM, ATP6V1G2, BAG6, BoLA, C2, C23H6orf25, C23H6orf47, CFB, CLIC1, CSNK2B, DDAH2, DDX39B, DXO, EHMT2, GPANK1, HSPA1A, HSPA1L, LOC100139548, LOC100847561, LOC104975663, LOC107131731, LOC107133388, LOC107133390, LOC617696, LOC781796, LOC783502, LSM2, LST1, LTA, LTB, LY6G5B, LY6G5C, LY6G6C, LY6G6D, LY6G6E, LY6G6F, MCCD1, MGC126945, MSH5, NCR3, NELFE, NEU1, NFKBIL1, PRRC2A, SAPCD1, SLC44A4, STK19, TNF, VARS, VWA7, ZBTB12*
23	27727673	27835871	108199	2	*BOLA, LOC788634, MIC1*
23	27961821	28535849	574029	3	*ABCF1, ATAT1, BOLA-NC1, C23H6orf136, DDR1, DHX16, DPCR1, FLOT1, GNL1, GTF2H4, IER3, LOC100847761, LOC101905956, LOC101906024, LOC104975665, LOC104975666, LOC104975667, LOC107131733, LOC107131734, LOC107133389, LOC512672, LOC614091, LOC615278, LOC787188, MDC1, MIR2378, MIR877, MRPS18B, MUC21, NRM, PPP1R10, PPP1R18, PRR3, RPP21, SFTA2, TUBB, VARS2*
23	29226041	29280403	54363	3	*LOC512579, LOC514434, LOC618034, LOC785431, OR12D3, OR5V1*
23	29280403	29504617	224215	7	*LOC107131736, LOC509155, LOC510530, LOC515704, LOC516273, LOC516274, LOC618052, LOC618064, LOC618070, LOC782262, LOC782301, LOC782341, LOC782379, LOC784557, LOC784614, LOC785162, LOC785252, LOC785277, LOC785479, OR14J1*
23	29504617	29576679	72063	8	*LOC528343, LOC784652, LOC784681*
23	29576679	29613713	37035	7	*LOC784787, OR2J3*
23	29613713	29699728	86016	9	*LOC511103, LOC616517, LOC784858, LOC785760, LOC789358, LOC789367*
23	29699728	29787545	87818	10	*LOC509280, LOC618140, LOC788663, LOC788675, OR2J2, OR2W1*
23	29787545	29851107	63563	9	*LOC104970719, LOC107133391, OR2W1*
23	29851107	29914101	62995	11	*LOC107131741, LOC782475, LOC782554*
23	29914101	29934831	20731	11	*LOC107131741*
23	29934831	30076786	141956	10	*LOC100847345, LOC101909685, LOC107131411, LOC784131, TRIM27, TRNAA-AGC, TRNAA-CGC, TRNAA-UGC, TRNAE-CUC, TRNAF-GAA, TRNAK-UUU, TRNAL-AAG, TRNAL-CAA, TRNAM-CAU, TRNAQ-CUG, TRNAR-CCG, ZNF311*
23	30076786	30240924	164139	2	*TRNAA-AGC, TRNAA-CGC, TRNAA-UGC, TRNAF-GAA, TRNAK-UUU, TRNAL-AAG, TRNAQ-UUG, TRNAS-GCU, TRNAT-AGU, TRNAT-CGU, ZSCAN12, ZSCAN31*

23	30240924	30525904	284981	4	*LOC104975673, LOC528914, LOC529518, LOC532291, LOC617979, NKAPL, PGBD1, TRNAS-GCU, ZKSCAN4, ZKSCAN8, ZNF165, ZNF389, ZSCAN16, ZSCAN26, ZSCAN9*
23	30525904	30710207	184304	3	*LOC100337366, LOC100849008, LOC101904987, LOC518468, LOC526989, LOC528373, LOC615902, LOC616650, LOC616658, LOC616700, LOC619021, LOC783203, LOC783311, OR2B6*
23	30710207	30966347	256141	5	*HIST1H1B, HIST1H2AG, HIST1H2AJ, HIST1H2BB, HIST1H2BI, HIST1H3C, LOC100295221, LOC100847609, LOC101906102, LOC104968446, LOC104975676, LOC522315, LOC527645, LOC528329, LOC529277, LOC614970, LOC616819, LOC616868, LOC783002, LOC783041, MIR2379, TRNAA-AGC, TRNAD-GUC, TRNAF-GAA, TRNAG-GCC, TRNAI-AAU, TRNAI-UAU, TRNAK-UUU, TRNAL-CAA, TRNAL-UAA, TRNAM-CAU, TRNAQ-CUG, TRNAQ-UUG, TRNAR-ACG, TRNAR-UCU, TRNAS-AGA, TRNAS-CGA, TRNAT-AGU, TRNAT-CGU, TRNAV-AAC*
23	30966347	31006337	39991	3	*MIR7857, TRNAI-AAU, TRNAM-CAU, TRNAQ-CUG, TRNAR-ACG, TRNAS-AGA, TRNAS-GCU, TRNAS-UGA, TRNAV-CAC*
23	31006337	31091923	85587	2	*TRNAC-GCA, TRNAD-GUC, TRNAQ-CUG, TRNAS-AGA, TRNAS-GCU, TRNAS-UGA, TRNAV-UAC, ZNF184, ZNF391*
23	31429004	32375669	946666	4	*BTN1A1, BTN2A1, BTN3A3, H2B, H4, HFE, HIST1H1A, HIST1H1C, HIST1H1D, HIST1H1E, HIST1H2AA, HIST1H2AC, HIST1H2AM, HIST1H2BA, HIST1H2BB, HIST1H2BD, HIST1H2BM, HIST1H3G, HIST1H3I, LOC100848369, LOC104968456, LOC104968510, LOC104968768, LOC104975682, LOC104975683, LOC104975684, LOC104975686, LOC107131750, LOC107133392, LOC505183, LOC518961, LOC521580, LOC527388, LOC537017, LOC617875, LOC617905, LOC786706, LOC787237, LOC787269, LOC787465, LRRC16A, MIR6531, SCGN, SLC17A1, SLC17A2, SLC17A3, SLC17A4, TRIM38, TRNAG-GCC, TRNAI-AAU, TRNAL-AAG, TRNAM-CAU, TRNAQ-UUG, TRNAR-ACG, TRNAR-UCG, TRNAS-AGA, TRNAS-GCU, TRNAS-UGA, TRNAW-CCA*
23	33250104	33515885	265782	10	*DCDC2, LOC104975690, NRSN1*
23	33515885	34742999	1227115	2	*LOC100296918, LOC100298767, LOC100336278, LOC522180, LOC780995, LOC788801, PRP1, PRP2, PRP4, PRP6, PRP8*
23	34742999	35401635	658637	5	*CSH2, LOC100336962, LOC615210, LOC787975, LOC787990, PRL, PRP-VII, PRP14, PRP3, PRP9*
23	35401635	35591864	190230	2	*HDGFL1, LOC104975692*
23	35591864	35695837	103974	3	.
23	35695837	35973313	277477	2	*LOC100847951, LOC104975693, LOC788707, MIR2284C*
23	35973313	36923152	949840	2	*CDKAL1, LOC100847951, LOC104968751, LOC104975694, LOC107131752, SOX4*
23	44249893	44474396	224504	3	*HIVEP1, LOC107131758*
23	44474396	44578434	104039	3	*LOC104969821, LOC107131758*
23	47810101	48172325	362225	4	*CAGE1, DSP, LOC104969862, RIOK1, RREB1, SSR1, TRNAE-UUC*
23	48172325	48206866	34542	4	.
23	48206866	48560728	353863	4	*LOC101902551, LOC101902683, LOC104969889, LY86*
23	52505108	52683926	178819	2	*LOC100138976, LOC101909053, LOC101909723, LOC104970720, LOC104970721, LOC107133395, LOC532311, LOC613394, LOC781404, OR5M10*
24	328670	459908	131239	5	*LOC100298725, LOC100301465, LOC515622, LOC518218, LOC788429, LOC788445, LOC788455, LOC789923, LOC789929*
24	459908	2216332	1756425	3	*ADNP2, ATP9B, CTDP1, HSBP1L1, KCNG2, LOC101902808, LOC101902953, LOC104975719, LOC107131769, LOC107131770, LOC515578, LOC529511, LOC788494, LOC789936, NFATC1, PARD6G, PQLC1, RBFA, SALL3, TRNAK-UUU, TXNL4A*
24	2216332	2385938	169607	8	*LOC101903078*
24	2385938	3647012	1261075	8	*GALR1, LOC100848212, LOC100848495, LOC101903290, LOC101904059, LOC104975721, MBP, ZNF236, ZNF516*
24	6566552	7656498	1089947	2	*CD226, LOC101907394, LOC104975731, RTTN, SOCS6*
24	7656498	8302723	646226	4	*CD226, DOK6, LOC101907606*
24	8302723	8323104	20382	5	.
24	8323104	8478283	155180	5	*CCDC102B, LOC104975734*
24	8478283	8715370	237088	4	*CCDC102B*
24	8715370	8954899	239530	2	*CCDC102B, LOC100337355, LOC107133396, TMX3*
24	9982903	11016329	1033427	5	*CDH19*
24	12870482	13110525	240044	3	*LOC100848171, LOC104976722, SYT4*
24	13110525	13195539	85015	4	*LOC781724*
24	13195539	13461018	265480	8	.
24	13461018	14804731	1343714	10	*LOC781770, PIK3C3, TRNAC-ACA*
24	14804731	14986844	182114	8	.
24	14986844	15251197	264354	5	*TRNAC-GCA*
24	15251197	16436545	1185349	10	*LOC100140947, LOC783699*
24	16436545	16703323	266779	8	.
24	16703323	17105354	402032	6	*LOC104975749*
24	17105354	18808533	1703180	4	*LOC107131773, LOC505339, TRNAK-UUU, TRNAW-CCA*
24	29404104	29509381	105278	6	.
24	29509381	29869700	360320	7	*LOC782418*
24	31899448	33256722	1357275	4	*CABYR, HRH4, IMPACT, LAMA3, LOC107131777, OSBPL1A, TRNAC-GCA, TTC39C, ZNF521*
24	35045493	35561252	515760	2	*ABHD3, ESCO1, GREB1L, LOC100847265, LOC107133397, MGC133647, SNRPD1*
24	49534282	49924926	390645	2	*C24H18orf32, DYM, LOC107131298, LOC509808, MIR2381, RPL17*
25	668	301100	300433	10	*FAM234A, HBA, HBA1, HBM, HBQ1, HBZ, IL9R, LOC100137913, LOC101906502, LOC789192, LUC7L, MPG, NPRL3, POLR3K, RGS11, RHBDF1, SNRNP25*
25	301100	939905	638806	3	*ARHGDIG, AXIN1, C1QTNF8, C25H16orf13, CACNA1H, CAPN15, CCDC78, CHTF18, DECR2, FAM173A, FAM195A, FBXL16, GNG13, HAGHL, LMF1, LOC100139040, LOC104975822, LOC516108, LOC531296, METRN, MRPL28, MSLN, NARFL, NHLRC4, NME4, PDIA2, PIGQ, PRR35, RAB11FIP3, RAB40C, RHBDL1, RHOT2, RPUSD1, SOX8, SSTR5, STUB1, TEKT4, TMEM8A, TRNAG-CCC, WDR24, WDR90, WFIKKN1*
25	8520122	8622964	102843	3	*GRIN2A, TRNAG-CCC*
25	18714140	18988244	274105	6	*ACSM3, DCUN1D3, ERI2, LOC104975861, LOC107131813, LYRM1, MGC134577, MIR2384*

25	18988244	19123369	135126	3	*LOC107131814, TRNAR-CCU*
25	21161644	21765543	603900	2	*COG7, DCTN5, EARS2, ERN2, NDUFAB1, PALB2, PLK1, SCNN1B, SCNN1G, UBFD1*
25	23416757	23717648	300892	3	*LOC100296463*
25	40648430	40894010	245581	6	*MIR2390, SDK1*
26	6554	613565	607012	4	*LOC100138850, LOC101903496, LOC104970047, LOC107131859, LOC107131861, LOC522322, LOC782168, LOC784314, LOC784380, LOC784465, LOC784714, LOC784751, OR5D18*
26	613565	868763	255199	2	*CISD1, IPMK, LOC107131860, UBE2D1*
26	1582391	1812286	229896	2	.
26	2805234	3281110	475877	3	*LOC785617, ZWINT*
26	3281110	3353881	72772	3	.
26	6417140	6658589	241450	2	*LOC101907086, LOC615206*
26	6658589	7214659	556071	4	*DKK1, PRKG1*
26	7214659	7301637	86979	4	*PRKG1*
26	7301637	7524860	223224	2	*PRKG1, TRNAQ-CUG*
26	7524860	7611087	86228	2	*CSTF2T, PRKG1*
26	8765310	9181088	415779	2	*LOC104975938, SGMS1*
26	16329919	16442455	112537	2	*CYP2C19, LOC505468*
26	16762406	17622790	860385	2	*ALDH18A1, BLNK, C26H10orf131, CC2D2B, CCNJ, DNTT, ENTPD1, LOC100848660, LOC101903321, LOC101903400, LOC101903992, LOC104975956, LOC107131876, LOC525649, SORBS1, TCTN3, ZNF518A*
26	25315262	25819181	503920	5	*CFAP58, LOC104975980, LOC104975981, LOC104975982, MIR2393, SORCS3*
26	31758403	32313440	555038	2	*ADRA2A, BBIP1, LOC100852061, LOC101906327, LOC107131889, MIR4680, MIR6524, PDCD4, RBM20, SHOC2*
26	35714685	35972066	257382	4	*FAM160B1, TRUB1*
26	35972066	36309014	336949	3	*ATRNL1*
26	40430609	40672518	241910	3	*INPP5F, MCMBP, SEC23IP*
26	40672518	40801067	128550	4	.
26	50798678	51172047	373370	2	*ADGRA1, LOC100850437, LOC101906136, LOC101908581, LOC104976015, LOC532031, LOC536342, LOC613726, LOC786314, TCERG1L*
26	51172047	51492542	320496	5	*INPP5A, LOC100848865, LOC101906198, LOC104968408, LOC104968412, LOC104976018, NKX6-2*
26	51492542	51887948	395407	6	*BNIP3, DPYSL4, JAKMIP3, LOC101902400, LOC104976020, LOC107131400, LRRC27, PWWP2B, STK32C*
27	26474312	27281408	807097	3	*LOC107133410, LOC107133411, WRN*
27	29115228	30761774	1646547	2	*DUSP26, LOC104976098, LOC104976101, LOC104976102, LOC783570, RNF122, UNC5D*
27	37857449	38442388	584940	2	*CSGALNACT1, SH2D4A*
27	38442388	38651622	209235	2	*LOC104970724, LOC107133412*
28	2900860	2978193	77334	5	*LOC100336396, LOC100847909*
28	15323668	15594721	271054	2	*MIR2403, SLC16A9*
28	15594721	15772990	178270	2	*CCDC6, MRLN*
28	20759229	20862144	102916	2	.
28	20862144	21441018	578875	2	.
28	23455477	24122830	667354	3	*CTNNA3, LOC101902668, LRRTM3, TRNAW-CCA*
28	24122830	24303173	180344	3	*CTNNA3*
28	29269052	29889777	620726	2	*ANXA7, CFAP70, DNAJC9, ECD, FAM149B1, LOC101902840, LOC101904869, LOC101904940, LOC107131940, MRPS16, MSS51, MYOZ1, NUDT13, P4HA1, PPP3CB, USP54*
28	31173519	31594380	420862	2	*COMTD1, LOC101903749, LOC101907017, LOC104969722, LOC107131935, LOC107131943, LOC508976, SAMD8, VDAC2, ZNF503*
28	31594380	32104452	510073	2	.
28	32104452	32133154	28703	2	.
28	37370303	37926764	556462	3	*NRG3*
28	38669808	39017338	347531	5	.
28	39017338	39171949	154612	3	.
28	39171949	39490317	318369	2	*LOC781868*
28	40289853	41077888	788036	2	*GRID1, TRNAG-CCC*
28	41077888	42182436	1104549	2	*ADIRF, BMPR1A, FAM25A, FAM35A, GLUD1, GRID1, LDB3, LOC104971065, MIR346, MMRN2, OPN4, SNCG, TRNAC-GCA, WAPL*
28	42182436	42373688	191253	2	*FAM35A, GPRIN2, SYT15*
28	42373688	42730191	356504	2	*ANTXRL, ANXA8L1, LOC101907562, LOC104971095, LOC107131410, LOC615066, PPYR1*
28	42730191	43192098	461908	2	*FRMPD2, GDF10, GDF2, LOC100294763, PTPN20, RBP3, ZNF488*
29	21878497	21961790	83294	4	.
29	27116249	27258966	142718	6	*LOC100299320, LOC100299628, LOC100300434, LOC100301071, LOC100336852, LOC104968406, LOC107133419, LOC506981, LOC510293, LOC522385, OR10S1*
29	27258966	27510423	251458	6	*LOC100301320, LOC504623, LOC517144, LOC523680, LOC616631, LOC781901, LOC782146, LOC782216, LOC782248, LOC782288, LOC782329, LOC782366, OR8G2, OR8G5, VWA5A*
29	27510423	27535814	25392	5	*LOC788328*
29	27535814	27580808	44995	6	*LOC100301231, LOC107133420*
29	27580808	27865578	284771	5	*LOC100301231, LOC100336420, LOC100850308, LOC506960, LOC509323, LOC512722, LOC531816, LOC614419, LOC781277, LOC781319, LOC781359, LOC781398, LOC781509, LOC781589, LOC781627, LOC781666, LOC781706, LOC781746, LOC781797, LOC781828, LOC782155, TMEM225*
29	27865578	28229632	364055	5	*LOC100139052, LOC100140862, LOC100141288, LOC100301297, LOC510984, LOC513062, LOC514864, LOC516940, LOC526276, LOC531225, LOC616755, LOC781383, LOC781842, LOC782009, LOC782191, LOC782221, LOC782257, LOC782297, LOC782375, LOC787625, LOC787694, LOC788544, LOC788554, LOC788573, LOC788590, LOC788607, LOC788620, OR8D2, OR8G5, TRNAV-CAC*
29	28229632	28258424	28793	4	*LOC100139434, LOC785107*
29	35026127	35124885	98759	2	*OPCML*

Supplementary Table 22. Summary of the results from the enrichment analysis of genes introgressed from *Bos javanicus* into Chinese indicine cattle and from yak into Tibetan taurine cattle according to RFMix analyses. The GO and KEGG analyses performed with DAVID 6.7 use lists of genes present in chromosomal regions detected as introgressed from *Bos javanicus* into Chinese indicine cattle and from yak into Tibetan taurine cattle according to RFMix analyses. *P* values are Bonferroni-corrected *P* values ⩽ 10^{-2}.

Category	Database entry	Term	Number of genes	Bonferroni	Enrichment
Enrichment analysis of genes introgressed from *Bos javanicus* into Chinese indicine					
GOTERM_BP_DIRECT	GO:0002323	Natural killer cell activation involved in immune response	30	1.31136E-10	4.2
GOTERM_BP_DIRECT	GO:0007186	G-protein coupled receptor signaling pathway	224	6.5058E-10	1.6
GOTERM_BP_DIRECT	GO:0002286	T cell activation involved in immune response	30	1.24248E-09	3.9
GOTERM_BP_DIRECT	GO:0033141	Positive regulation of peptidyl-serine phosphorylation of STAT	29	4.79439E-09	3.9
GOTERM_BP_DIRECT	GO:0042100	B cell proliferation	31	1.81381E-07	3.4
GOTERM_BP_DIRECT	GO:0006959	Humoral immune response	33	1.14066E-06	3.1
GOTERM_BP_DIRECT	GO:0043330	Response to exogenous dsRNA	31	1.55757E-06	3.2
GOTERM_BP_DIRECT	GO:0019221	Cytokine-mediated signaling pathway	51	4.22693E-06	2.4
GOTERM_BP_DIRECT	GO:0002250	Adaptive immune response	37	1.27905E-05	2.7
GOTERM_BP_DIRECT	GO:0030183	B cell differentiation	32	9.93E-04	2.6
GOTERM_BP_DIRECT	GO:0007608	Sensory perception of smell	69	1.13E-03	1.8
KEGG_PATHWAY	bta04740:	Olfactory transduction	269	1.20782E-05	1.3
KEGG_PATHWAY	bta04742:	Taste transduction	22	3.33E-04	3.0
KEGG_PATHWAY	bta05322:	Systemic lupus erythematosus	60	1.20E-03	1.8
Enrichment analysis of genes introgressed from yak into Tibetan taurine cattle					
GOTERM_BP_DIRECT	GO:0007608	sensory perception of smell	33	1.10927E-07	3.9
GOTERM_BP_DIRECT	GO:1903352	L-ornithine transmembrane transport	9	2.51448E-06	19.3
GOTERM_BP_DIRECT	GO:0002504	antigen processing and presentation of peptide or polysaccharide antigen via MHC class II	9	5.12E-04	11.8
KEGG_PATHWAY	bta05332:	Graft-versus-host disease	16	2.18123E-08	8.8
KEGG_PATHWAY	bta04940:	Type I diabetes mellitus	15	6.09463E-06	6.6
KEGG_PATHWAY	bta05323:	Rheumatoid arthritis	18	1.28E-04	4.4
KEGG_PATHWAY	bta05330:	Allograft rejection	13	1.91E-04	6.1
KEGG_PATHWAY	bta05416	Viral myocarditis	14	1.58E-03	4.7
KEGG_PATHWAY	bta05321	Inflammatory bowel disease (IBD)	14	1.86E-03	4.6
KEGG_PATHWAY	bta05320	Autoimmune thyroid disease	13	2.25E-03	4.9

Supplementary Table 23. Information of regions of banteng introgression into Chinese indicine cattle associted with heat stress

Chrom	Start (bp)	End (bp)	Size (bp)	Hap (n)	Gene content of the introgressed interval	Biological process
6	31905934	32352232	446299	2	***ATOH1*** *HPGDS LOC100140135 SMARCAD1*	ATOH1 is involved in hair cell differentiation
7	45725639	46602147	876509	2	*ABHD17A ADAT3 AFF4 ATP8B3 BTBD2 CSNK1G2 GDF9* ***HSPA4*** *KLF16 LEAP2 LOC104969170 LOC107131164 LOC107132633 LOC107132634 LOC781086 LOC781691 MBD3 MEX3D MIR6120 PLK5 REXO1 SCAMP4 SHROOM1 SOWAHA TCF3 UQCR11 UQCRQ ZCCHC10*	
8	53852427	54953273	1100847	2	*CEP78 GNA14 GNAQ LOC101907250 LOC104969366 LOC104972905 LOC107132694 PSAT1* ***VPS13A***	Blood circulation
8	65476773	66108488	631716	5	*ERP44* ***INVS*** *LOC101908166 LOC107132702 NR4A3 STX17 TEX10*	Blood circulation
9	27664454	29613821	1949368	7	*CLVS2 FABP7* ***HSF2*** *LOC100848869 LOC101904186 LOC101905260 LOC101908885 LOC101909028 LOC107132761 LOC781754 MIR2478 PKIB SERINC1 SMPDL3A TRDN*	The protein encoded by this gene belongs to the HSF family of transcription factors that bind specifically to the heat-shock promoter element and activate transcription. Heat shock transcription factors activate heat-shock response genes under conditions of heat or other stresses.
15	34351275	34537981	186707	3	*CLMP* ***HSPA8*** *LOC107133162*	Heat stress induces formation of cytoplasmic granules
15	22694978	23094071	399094	2	*BCO2 C15H11orf1 C15H11orf52 C15H11orf57 CRYAB DIXDC1 DLAT* ***HSPB2*** *IL18 LOC101903059 LOC101903125 LOC101906594 LOC104968480 LOC104974189 PIH1D2 PTS SDHD TEX12 TIMM8B*	
19	3842129	4335161	493033	4	***KIF2B***	Blood circulation
23	27261793	27727673	465881	8	*ABHD16A AIF1 APOM ATP6V1G2 BAG6 BoLA C2 C23H6orf25 C23H6orf47 CFB CLIC1 CSNK2B DDAH2 DDX39B DXO EHMT2 GPANK1* ***HSPA1A HSPA1L*** *LOC100139548 LOC100847561 LOC104975663 LOC107131731 LOC107133388 LOC107133390 LOC617696 LOC781796 LOC783502 LSM2 LST1 LTA LTB LY6G5B LY6G5C LY6G6C LY6G6D LY6G6E LY6G6F MCCD1 MGC126945 MSH5 NCR3 NELFE NEU1 NFKBIL1 PRRC2A SAPCD1 SLC44A4 STK19 TNF VARS VWA7 ZBTB12*	This intronless gene encodes a 70kDa heat shock protein which is a member of the heat shock protein 70 family. In conjuction with other heat shock proteins, this protein stabilizes existing proteins against aggregation and mediates the folding of newly translated proteins in the cytosol and in organelles.The HSPA1A are heat inducible.

Supplementary Table 24. Most negative and significant *f3* statistics for various species of the tribe of Bovini, with Tibetan taurine cattle as the target population and Hanwoo or Mishima as one of the source populations.

Source1	Source2	Target	*f3*	Standard error	Z-score
**Yak**	**Hanwoo**	**Tibetan**	**-0.05006**	**0.004209**	**-11.893**
Wisent	Hanwoo	Tibetan	-0.02297	0.002637	-8.708
Bison	Hanwoo	Tibetan	-0.01903	0.002587	-7.356
Gaur	Hanwoo	Tibetan	-0.01573	0.002362	-6.661
Banteng	Hanwoo	Tibetan	-0.01401	0.002252	-6.221
Buffalo	Hanwoo	Tibetan	-0.01202	0.002171	-5.535
Gayal	Hanwoo	Tibetan	-0.00995	0.002097	-4.746
**Yak**	**Mishima**	**Tibetan**	**-0.05583**	**0.004202**	**-13.287**
Wisent	Mishima	Tibetan	-0.02943	0.002712	-10.85
Bison	Mishima	Tibetan	-0.02596	0.002704	-9.601
Gaur	Mishima	Tibetan	-0.02287	0.002492	-9.176
Banteng	Mishima	Tibetan	-0.02115	0.002365	-8.94
Buffalo	Mishima	Tibetan	-0.01851	0.002283	-8.111
Gayal	Mishima	Tibetan	-0.01754	0.002363	-7.42

Supplementary Table 25. The result of *D* statistic tests performed to detect admixture from of the tribe of Bovini (Y) to either W or X. Negative *D* statistic values indicate that gene flow has occurred from Y to X, and positive *D* statistic values indicate that gene flow has occurred from Y to W.

W	X	Y	Z	*D* statistic	Z-value
**Mishima**	**Tibetan**	Yak	**Buffalo**	**-0.1492**	**-11.731**
Mishima	Tibetan	Wisent	Buffalo	-0.0505	-13.005
Mishima	Tibetan	Bison	Buffalo	-0.0422	-10.546
Mishima	Tibetan	Banteng	Buffalo	-0.0505	-7.5572
Mishima	Tibetan	Gayal	Buffalo	-0.0115	-2.0212
Mishima	Tibetan	Gaur	Buffalo	-0.0276	-6.3492

Supplementary Table 26. Statistics on introgressed intervals for nine Tibetan taurine cattle. The following values are shown for each Tibetan taurine cattle: the number of introgressed intervals (count); the minimal (min), maximal (max), mean (mean), and sum (sum) of introgressed interval lengths. The proportion of the cattle genome that is introgressed (proportion).

Number	Sample	Count	Introgression interval length				Proportion
			Min	Max	Mean	Sum	
1	Tibetan01	696	1,423	1,977,400	78,147	54,390,591	0.01075
2	Tibetan02	701	1,292	1,235,984	65,133	45,658,902	0.00903
3	Tibetan03	846	1,425	1,312,877	78,387	66,315,608	0.01311
4	Tibetan04	1,132	1,448	3,799,924	63,388	71,755,549	0.01419
5	Tibetan05	574	1,495	1,830,515	117,618	67,512,744	0.01335
6	Tibetan06	626	1,454	5,531,465	237,645	148,765,835	0.02941
7	Tibetan07	981	1,442	1,246,271	29,123	28,570,204	0.00565
8	Tibetan08	891	1,423	1,307,606	53,318	47,506,582	0.00939
9	Tibetan09	332	1,448	1,541,354	71,514	23,742,899	0.00469
	Max	1,132	1,495	5,531,465	237,645	148,765,835	0.02941
	Min	332	1,292	1,235,984	29,123	23,742,899	0.00469
	Mean	753	1,427	2,198,155	88,252	61,579,879	0.01218
	Standard deviation	238	55	1,487,471	607,554	36,720,324	0.00726
	Standard error	58	13	360,764	147,353	8,905,986	0.00176

Supplementary Table 27. List of introgression intervals from yak into Tibetan taurine cattle detected by RFMix analyses and were shared by at least two haplotypes

Chromosome (Btau_5.0.1) start and end positions and number of introgressed haplotype and gene content of introgessed intervals.

Chr	Start (bp)	End (bp)	Length (bp)	Hap (n)	Gene ID
1	113643307	114213616	570310	4	*LOC100847501 MME TRNAK-UUU*
1	114213616	114277037	63422	4	.
1	114277037	114768002	490966	5	*ARHGEF26 DHX36 GPR149 TRNAC-GCA*
1	114768002	115005269	237268	5	*ARHGEF26*
1	115005269	115199245	193977	6	*LOC783160 LOC784259*
1	115199245	115553700	354456	6	*LOC784259*
1	115553700	115675721	122022	6	.
1	115675721	115992033	316313	9	*LOC100299503 RAP2B TRNAG-CCC*
1	115992033	116023617	31585	8	*LOC104970034*
1	116023617	116558953	535337	8	*LOC104969763 P2RY1*
1	116558953	117229917	670965	8	*LOC615622 MBNL1*
1	117229917	117861610	631694	7	*AADAC AADACL2 LOC100300210 LOC107132208 LOC523130 LOC613884 LOC782222 LOC782258 LOC782298 MIR1246 SUCNR1*
1	117861610	117979692	118083	8	*LOC104971000*
1	117979692	118068325	88634	8	*IGSF10 MED12L P2RY12*
1	118068325	118619420	551096	8	*CLRN1 GPR171 GPR87 LOC104971001 MED12L P2RY12 P2RY13 P2RY14*
1	118619420	119420496	801077	6	*EIF2A ERICH6 LOC104971002 LOC104971004 LOC104971005 LOC104971006 SELT SERP1 SIAH2 TRNAR-CCU TSC22D2*
1	119420496	119543658	123163	7	*LOC101905340*
1	119543658	119662047	118390	6	*PFN2 RNF13*
1	119662047	120450802	788756	7	*ANKUB1 COMMD2 CP HPS3 LOC100847676 LOC101905481 LOC104971009 LOC526230 RNF13 TM4SF1 TM4SF18 TM4SF4 TRNAG-CCC WWTR1*
1	120450802	120625485	174684	7	*GYG1 HLTF*
1	120625485	120792006	166522	8	*CPA3 CPB1*
1	120792006	120805239	13234	8	.
1	120805239	120910944	105706	8	*AGTR1*
1	120910944	121561875	650932	6	*AGTR1 LOC104968807 LOC781192*
1	121561875	122566404	1004530	7	*LOC100139345 LOC100295922 LOC101905819 LOC101906885 LOC786256 TRNAC-ACA ZIC1 ZIC4*
1	122566404	122960109	393706	6	.
1	144615772	145122298	506527	2	*LOC101905728 PDE9A RSPH1 SLC37A1 TFF1 TFF2 TMPRSS3 UBASH3A*
2	13155924	13784582	628659	3	*DUSP19 FRZB NCKAP1 NUP35 TRNAC-GCA*
2	13784582	14475877	691296	3	*DNAJC10 FRZB LOC107132237 PDE1A*
2	18361681	18637847	276167	2	*DFNB59 FKBP7 LOC785777 OSBPL6 PLEKHA3 PRKRA TTN*
2	18637847	19005589	367743	7	*CYCT OSBPL6 PDE11A RBM45*
2	19005589	19542351	536763	9	*AGPS LOC781702 PDE11A TTC30A TTC30B*
2	19542351	19578766	36416	7	.
2	19578766	19648656	69891	7	*AGPS*
2	19648656	20143878	495223	6	*HNRNPA3 LOC104971138 MIR4444 NFE2L2*
2	20143878	20911198	767321	6	*HOXD1 HOXD10 HOXD3 HOXD4 HOXD8 HOXD9 LOC100140646 LOC100847387 LOC104971140 LOC104971141 LOC104971146 MIR10B MTX2*
2	20911198	20977966	66769	3	*HOXD11 HOXD12 HOXD13 LOC107132238*
2	25940708	27771222	1830515	3	*ABCB11 BBS5 CCDC173 CERS6 DHRS9 FASTKD1 G6PC2 KLHL23 KLHL41 LOC101906273 LOC104971168 LRP2 METTL5 MIR2353 MYO3B NOSTRIN PHOSPHO2 PPIG SPC25 SSB TRNAR-UCU UBR3*
2	27771222	27809053	37832	2	.
2	27809053	27897825	88773	2	.
2	27897825	28116851	219027	2	*LOC107132248 STK39*
3	53008466	53315742	307277	2	.
3	64688481	65175398	486918	2	.
3	81905816	82166303	260488	2	*ROR1 UBE2U*
3	82166303	82540034	373732	2	*ROR1*
3	82540034	82737858	197825	2	*EFCAB7 ITGB3BP PGM1*
3	82737858	83160984	423127	3	*ALG6 FOXD3 ITGB3BP LOC101906485*
4	1282343	2864382	1582040	2	*LOC107132368 LOC784126*
4	2864382	2893888	29507	2	.
4	2893888	3170182	276295	2	.
4	3170182	3300026	129845	2	.
4	3300026	3826707	526682	2	*LOC107132369*
4	3826707	4103887	277181	2	*LOC100139578*
4	4103887	4484206	380320	2	.
4	4484206	5369594	885389	2	*COBL DDC GRB10 LOC107131141 TRNAT-UGU*
4	5369594	5784693	415100	2	*C4H7orf72 DDC FIGNL1 IKZF1 LOC101903668 ZPBP*
4	5784693	5818696	34004	2	*ZPBP*
4	5818696	5856645	37950	2	*ZPBP*
4	5856645	6436870	580226	2	*LOC101904266 VWC2 ZPBP*
4	7685123	7771543	86421	2	*CLDN12*
4	7771543	7994889	223347	2	*CDK14 LOC100847712 LOC101910184*
4	7994889	8032883	37995	2	*CDK14*
4	8032883	8150521	117639	3	*CDK14*
4	8150521	8245381	94861	3	*CDK14*
4	8245381	8612189	366809	3	*CDK14 FZD1 LOC104971924*
4	8612189	8626304	14116	3	.
4	8626304	8648253	21950	3	*LOC782091*
4	8648253	9009448	361196	3	*LOC100140224*
4	9009448	9107648	98201	3	*LOC101908673 LOC107132376*
4	9107648	9132995	25348	3	*LOC101908673*
4	9132995	9182533	49539	3	*LOC101908673 MTERF1*
4	9182533	10134283	951751	2	*AKAP9 ANKIB1 CDK6 CYP51A1 FAM133B GATAD1 KRIT1 LOC101906914 LOC104971926 LOC107132377 LOC524650 LRRD1 MIR584-5 PEX1 RBM48 TMBIM1B TRNAC-ACA TRNAG-CCC*
4	31106122	31219675	113554	3	*RAPGEF5*
4	31219675	31268443	48769	4	*RAPGEF5*

4	31268443	32028173	759731	4	*FAM126A IL6 LOC100138586 LOC100139677 LOC100848985 LOC101907914 LOC101907978 LOC107132385 LOC613401 RAPGEF5 TOMM7*
4	32028173	32404013	375841	4	*GPNMB IGF2BP3 KLHL7 LOC101902372 LOC107132386 MALSU1 NUPL2 TRA2A*
4	32404013	33716889	1312877	4	*ABCB1 ABCB4 CCDC126 CROT DBF4 DMTF1 KIAA1324L LOC100296627 LOC101902435 LOC101902991 LOC107132387 LOC107132388 LOC107132389 LOC511386 LOC512293 LOC781303 LOC785370 RUNDC3B SLC25A40 TMEM243 TRNAC-GCA*
4	33716889	34054750	337862	5	*GRM3 KIAA1324L LOC107132390*
4	34054750	34690818	636069	5	*GRM3 LOC100337094 LOC101904927 LOC107132390 TRNAS-GGA*
4	34690818	34894334	203517	4	*LOC781830*
4	34894334	35380283	485950	4	*LOC104969864 TRNAC-GCA*
4	35380283	35437839	57557	5	.
4	35437839	36119017	681179	5	*LOC104972008 SEMA3D*
4	36119017	36577232	458216	6	.
4	36577232	37109665	532434	5	*LOC104972001 SEMA3A*
4	37109665	37912794	803130	5	*LOC104972003 PCLO SEMA3E*
4	37912794	38042750	129957	3	*PCLO*
4	38042750	38049926	7177	2	*PCLO*
4	61426832	63140519	1713688	3	*ANLN DPY19L1 DPY19L2 EEPD1 HERPUD2 KIAA0895 LOC101906333 LOC107132407 LOC780968 LOC782400 NPSR1 SEPT7 TBX20 TRNAG-GCC*
4	63140519	63575683	435165	5	*BMPER*
4	63575683	63781722	206040	4	*MIR1814C*
4	73912316	74181346	269031	2	*ZNF804B*
4	74181346	74377877	196532	2	.
5	40725879	40835036	109158	2	*LOC534391 LRRK2 MUC19*
5	40835036	40914259	79224	2	*LRRK2*
5	41851904	42002693	150790	2	*LOC785955*
5	42002693	42012783	10091	2	.
5	42012783	42032266	19484	2	*LOC100847470*
5	42032266	42391311	359046	2	*KIF21A LOC617654 MIR2428*
5	42391311	42650020	258710	2	*CPNE8 LOC101907676*
5	42650020	42795774	145755	2	*CPNE8*
5	43468188	43676372	208185	2	*CNOT2 KCNMB4 LOC107131684 LOC107132479*
5	43676372	44587511	911140	2	*BEST3 CCT2 FRS2 LOC100138933 LOC104972423 LOC107132482 LOC107132483 LOC781146 LOC785803 LRRC10 LYZ1 MIR2427 MYRFL RAB3IP YEATS4*
5	44587511	44659440	71930	2	.
5	102669251	102876585	207335	3	*LOC101907335 LOC751788 WC-7*
5	102876585	103143527	266943	2	*LOC100335428 LOC751788 LOC751789 WC1-12*
5	103143527	103362683	219157	2	*LOC100336766 LOC107132521 LOC786796 WC1-10*
5	103362683	103455585	92903	3	*LOC100299671 WC1-10 WC1.3*
5	103455585	103469955	14371	2	*WC1.3*
5	103469955	103698356	228402	2	*LOC540180 LOC751804 WC1 WC1-8 WC1.3*
5	106437129	107631887	1194759	2	*CCND2 CRACR2A FGF23 FGF6 LOC100847686 LOC101902968 LOC104972567 LOC104972568 LOC107132523 LOC107132524 PARP11 PRMT8 TEAD4 TIGAR TSPAN11 TSPAN9*
5	114435850	114819029	383180	2	*ARFGAP3 LOC100298838 PACSIN2*
6	22371695	22449947	78253	2	.
6	22449947	22592758	142812	2	.
6	28312754	28473100	160347	2	*LOC104972708*
6	28473100	29194796	721697	2	*LOC104972708 LOC104972710 LOC104972713 LOC782904 LOC782977 TRNAC-GCA*
6	94322155	94324396	2242	2	.
6	94324396	94447149	122754	2	*CXCL13 LOC100137799*
7	30141886	33941809	3799924	2	*CEP120 CSNK1G3 FTMT LOC100140613 LOC101906976 LOC101907246 LOC104969136 LOC104969139 LOC104969140 LOC104972827 LOC107132619 LOC107132620 LOC107132621 LOC107132622 LOC521901 LOC781423 LOC789456 LOX PPIC PRDM6 SNCAIP SNX2 SNX24 SRFBP1 ZNF608*
7	33941809	35188079	1246271	6	*FAM170A LOC100296599 LOC782989 LOC784563 PRR16*
7	35188079	35932756	744678	5	*HSD17B4 LOC100295797 LOC101907740 LOC104970228 LOC107132623 LOC540403*
7	92743862	93116615	372754	2	*ADGRV1 LYSMD3 MBLAC2 POLR3G*
8	18529969	19837574	1307606	2	*IZUMO3 LOC104969285 LOC104969286 LOC104969287 TUSC1*
9	64906054	65310701	404648	2	*LOC100174924 LOC100335322 LOC101906498 LOC104968426 LOC509791 NT5E SNX14 SYNCRIP*
9	65310701	65692356	381656	2	*LOC101907349 LOC104970099*
9	86236191	86711367	475177	2	*LOC100850276 SAMD5*
9	86711367	88829836	2118470	2	*GINM1 KATNA1 LATS1 LOC100299874 LOC100336795 LOC100848282 LOC101903261 LOC104969619 LOC104969628 LOC104972992 LOC107131280 LOC107132784 LOC107132785 LOC107132786 LOC107132787 LOC513548 LOC782566 LOC784274 LOC786867 LRP11 NUP43 PCMT1 PPIL4 PPP1R14C RAET1G SASH1 TAB2 ULBP21 ULBP27 UST ZC3H12D*
9	91600513	96158556	4558044	2	*ARID1B CLDN20 CNKSR3 IPCEF1 LDHAL6B LOC100298280 LOC100848475 LOC101902301 LOC101902393 LOC104969630 LOC104969633 LOC104970693 LOC104972996 LOC107132791 LOC514725 LOC616500 LOC616595 LOC617457 LOC783190 LOC788337 LOC789941 LOC789961 MIR2480 MIR2481 NOX3 OPRM1 OR4A15 OR4C6 SCAF8 TFB1M TIAM2 TMEM242 TRNAC-ACA ZDHHC14*

9	96158556	100211145	4052590	2	*ACAT2 AGPAT4 AIRN DYNLT1 EZR FNDC1 GTF2H5 IGF2R LOC100847777 LOC101903438 LOC101904173 LOC101906344 LOC104969639 LOC104969640 LOC104969641 LOC104969644 LOC107132792 LOC107132793 LOC107132794 LOC107132795 LOC521027 LOC781966 LOC783891 MAP3K4 MAS1 MIR2482 MRPL18 PACRG PARK2 PLG PNLDC1 RSPH3 SERAC1 SLC22A1 SLC22A3 SNX9 SOD2 SYNJ2 SYTL3 TAGAP TCP1 TMEM181 TRNAG-UCC TULP4 WTAP ZDHHC14*
10	2518634	2657018	138385	2	.
10	10164286	10326715	162430	2	*BHMT JMY TRNAC-GCA*
10	10326715	10785480	458766	2	*CMYA5 HOMER1 JMY LOC104973035 LOC107132818 PAPD4*
10	96423289	96630703	207415	3	.
10	96630703	97043276	412574	3	.
10	97043276	97304221	260946	3	.
10	97304221	97603679	299459	4	.
10	97603679	97868957	265279	4	*MIR2293*
11	45357627	45689572	331946	2	*ST6GAL2*
11	45689572	45841028	151457	4	*LOC107132937 LOC532089 UXS1*
11	45841028	46240416	399389	5	*C11H2orf40 LOC101903923 LOC786288 NCK2*
11	46240416	46248833	8418	6	.
11	46248833	46348628	99796	6	*CHCHD5 POLR1B TTL*
11	46348628	47006375	657748	6	*CKAP2L IL1A IL1B IL1F10 IL1RN IL36A IL36B IL36G IL36RN IL37 LOC101903687 LOC101904177 LOC104973371 LOC107132933 LOC107132936 NT5DC4 PAX8 PSD4 SLC20A1*
11	47006375	47214431	208057	5	*LOC100335748 LOC100847269 PAX8*
11	47214431	47259744	45314	3	*LOC100294952 LOC100335748 LOC100847269 LOC614219*
11	47259744	47280760	21017	3	*LOC100294952*
11	47280760	47775386	494627	4	*EIF2AK3 FOXI3 LOC100294952 LOC784634 RPIA TEX37 TRNAC-ACA TRNAS-GGA*
12	7538138	7538883	746	2	.
12	7980108	8016320	36213	2	.
12	8251330	8252089	760	2	.
12	8475839	8477312	1474	2	.
12	8610210	8610712	503	2	.
12	8978864	8979483	620	2	.
12	9024310	9030056	5747	2	.
12	9030056	9071761	41706	2	.
12	9071761	9098634	26874	3	.
12	9098634	9111749	13116	3	.
12	9111749	9133887	22139	3	*LOC101905933*
12	9133887	9150883	16997	2	.
12	9150883	9163103	12221	2	.
12	9497165	9497829	665	2	.
12	55214601	55217820	3220	2	*LOC104973609*
12	55217820	55258686	40867	2	*LOC104973609*
12	55258686	55289226	30541	2	*LOC104973609*
12	55289226	55293758	4533	2	*LOC104973609*
12	55293758	55293813	56	3	*LOC104973609*
12	55293813	55294014	202	3	*LOC104973609*
12	55294014	55298329	4316	3	*LOC101904423 LOC104973609*
12	55298329	55300769	2441	4	*LOC101904423 LOC104973609*
12	55300769	55349623	48855	3	*LOC101904423 LOC104973609*
12	55349623	55381927	32305	2	*LOC104973609*
12	55381927	55402608	20682	2	*LOC104973609*
12	55402608	55463374	60767	2	*LOC104973609*
12	55463374	55472521	9148	2	.
12	55472521	55477506	4986	2	.
12	55477506	55508881	31376	2	.
12	55508881	55509247	367	2	.
12	55509247	55515543	6297	3	.
12	55515543	55538706	23164	3	.
12	55538706	55588322	49617	3	.
12	55588322	55661418	73097	3	*TRNAW-CCA*
12	55661418	55666219	4802	3	.
12	55666219	55672035	5817	3	.
12	55672035	55672210	176	3	.
12	55672210	55754907	82698	3	*LOC614423*
12	55754907	55856189	101283	3	*LOC107133001 SPRY2*
12	55856189	55877673	21485	3	*TRNAG-UCC*
12	55877673	55922400	44728	3	.
12	55922400	55939474	17075	2	*LOC104976344*
12	55939474	55965662	26189	2	*LOC104976344*
12	55965662	55967484	1823	2	*LOC104976344*
12	55967484	55969726	2243	2	*LOC104976344*
12	70372587	70372981	395	3	*LOC515333*
12	70372981	70374360	1380	4	*LOC515333*
12	70374360	70374899	540	4	*LOC515333*
12	70374899	70375199	301	4	*LOC515333*
12	70375199	70376645	1447	4	*LOC515333*
12	70376645	70381901	5257	4	*LOC515333*
12	70381901	70390764	8864	5	*LOC515333*
12	70390764	70404034	13271	5	*LOC515333*
12	70404034	70404416	383	5	*LOC515333*
12	70404416	70405761	1346	5	*LOC515333*
12	70405761	70414629	8869	4	*LOC515333*
12	70414629	70423556	8928	4	.
12	70423556	70440976	17421	5	.
12	70440976	70444039	3064	6	.
12	70444039	70454402	10364	5	.
12	70454402	70488945	34544	4	.
12	70488945	70497990	9046	4	.

12	70497990	70572258	74269	4	*LOC100336951*
12	71248881	71506201	257321	2	*LOC100336232 LOC100848700 LOC101902419 LOC107133006*
12	71506201	71671198	164998	2	*LOC100336999 LOC100848700*
12	71671198	71942344	271147	2	*LOC100298891 LOC100336999 LOC100337006*
12	72474848	72508883	34036	2	*LOC100337053 LOC100850004*
12	72590708	72598620	7913	2	*LOC100337069*
12	72598620	72615021	16402	3	*LOC100337069*
12	72615021	72626512	11492	4	*LOC100337069*
12	72626512	72692021	65510	5	*LOC100337069 LOC101902505*
12	72692021	72739795	47775	7	.
12	72739795	72742095	2301	8	.
12	72742095	72742566	472	9	.
12	72742566	73124296	381731	8	*LOC100337076 LOC100337108 LOC100848914 LOC104969875 LOC530803 TRNAC-GCA*
12	73124296	74360279	1235984	10	*LOC100299180 LOC100337129 LOC101902555 LOC101902625 LOC101904517 LOC101908868 LOC104969875 LOC104970090 LOC104970255 LOC104970322 LOC104970696 LOC107131247 LOC107131259 LOC107131271 LOC107131276 LOC107131284 LOC107131396 LOC789637 MIR3141*
12	74360279	74828299	468021	9	*LOC100847245 LOC101902687 LOC104970365 LOC104973638 LOC107131255 LOC107131271 LOC107133007 LOC107133008 LOC520016 LOC790322*
12	74828299	75318435	490137	9	*LOC100337244 LOC107133009 LOC107133010 LOC509854 LOC520016*
12	75318435	75667025	348591	8	*LOC100847383 LOC101902837 LOC101906170 LOC107131256 LOC107131273 LOC506823 LOC509854*
12	75667025	75682157	15133	8	.
12	75682157	75744655	62499	8	*LOC100337292*
12	75744655	75769234	24580	8	*LOC101906567*
12	75769234	75957740	188507	9	*LOC100296113 LOC101906567 LOC101908758 LOC104973640 LOC107133011 LOC522174*
12	75957740	76192411	234672	9	*LOC101906812 LOC104969731 LOC523126*
12	76192411	76239846	47436	9	*LOC523126*
12	76239846	76316144	76299	9	*LOC100847548*
12	76316144	76421981	105838	9	*LOC100847548 LOC101906931 LOC101907065 LOC104973641*
12	76421981	76835626	413646	9	*LOC100335848 LOC100337390 LOC101902894 LOC101907065 LOC101907355 LOC101909319 LOC101909342 LOC104970697 LOC107133012 LOC107133013 LOC530437*
12	76835626	76983325	147700	8	*LOC101907471 LOC616707*
12	76983325	77084292	100968	8	*LOC100851360 LOC101903448 LOC104970698 LOC527057*
12	77084292	77110586	26295	8	*LOC101903448*
12	77110586	77138628	28043	7	*CLDN10 LOC101903448*
12	77138628	77146359	7732	6	*CLDN10*
12	77146359	77151877	5519	5	*CLDN10*
12	77151877	77159609	7733	4	*CLDN10*
12	77159609	77164744	5136	4	*CLDN10 LOC104973643*
12	77164744	77180349	15606	4	*CLDN10*
12	77180349	77208462	28114	4	*CLDN10*
12	77208462	77215792	7331	4	*CLDN10*
12	77215792	77222993	7202	4	*CLDN10*
12	77222993	77223831	839	4	*CLDN10*
12	77223831	77254667	30837	4	*CLDN10 DZIP1*
12	77254667	77283755	29089	4	*DZIP1*
12	77283755	77286290	2536	4	*DZIP1*
12	77286290	77305962	19673	4	*DZIP1*
12	77305962	77311859	5898	4	.
12	77311859	77323832	11974	4	*LOC785064*
12	77323832	77328079	4248	4	.
12	77328079	77328353	275	4	.
12	77328353	77331474	3122	4	.
12	77331474	77337002	5529	4	*DNAJC3*
12	77337002	77350199	13198	4	*DNAJC3*
12	77350199	77351876	1678	4	*DNAJC3*
12	77351876	77352964	1089	4	*DNAJC3*
12	77352964	77356055	3092	4	*DNAJC3*
12	77356055	77362214	6160	4	*DNAJC3*
12	77362214	77363175	962	5	*DNAJC3*
12	77363175	77363579	405	6	*DNAJC3*
12	77363579	77364041	463	5	*DNAJC3*
12	77364041	77402048	38008	4	*DNAJC3*
12	77402048	77406930	4883	4	*UGGT2*
12	77406930	77427307	20378	4	*UGGT2*
12	77427307	77427739	433	4	*UGGT2*
12	77427739	77437194	9456	4	*UGGT2*
12	77437194	77439412	2219	4	*UGGT2*
12	77439412	77462516	23105	4	*UGGT2*
12	77462516	77504916	42401	4	*UGGT2*
12	77504916	77519177	14262	4	*UGGT2*
12	77519177	77524301	5125	4	*UGGT2*
12	77524301	77532233	7933	4	*UGGT2*
12	77532233	77547001	14769	3	*UGGT2*
12	77547001	77557892	10892	3	*UGGT2*
12	77557892	77566741	8850	2	.
12	77566741	77577160	10420	2	.
12	77577160	77578469	1310	2	.
12	87440367	87440909	543	2	.
12	87889766	87939946	50181	2	.
12	87939946	87940714	769	2	.
12	87940714	87985678	44965	2	.
12	88104642	88118906	14265	2	*LOC107131142*
12	88118906	88139033	20128	2	.

12	88139033	88142755	3723	2	.
12	88142755	88145354	2600	2	.
12	88145354	88203388	58035	2	.
12	88203388	88204359	972	2	.
12	88303286	88312967	9682	2	.
12	88312967	88373387	60421	2	*MYO16*
12	88470650	88480564	9915	2	*MYO16*
12	88480564	88485972	5409	2	*MYO16*
12	88485972	88578709	92738	2	*LOC104973670 MYO16*
12	88578709	88594536	15828	3	*MYO16*
12	88594536	88604196	9661	2	*MYO16*
12	88604196	88609662	5467	2	*MYO16*
12	88609662	88654866	45205	2	*MYO16*
12	88654866	88655578	713	2	*MYO16*
12	88655578	88750752	95175	2	*MYO16 TRNAY-AUA*
12	88750752	88755266	4515	2	.
12	90144735	90147454	2720	2	.
12	90147454	90169116	21663	2	.
12	90169116	90177829	8714	2	.
12	90177829	90178871	1043	2	.
12	90178871	90196246	17376	2	.
12	90196246	90223606	27361	2	.
12	90223606	90227672	4067	2	.
12	90227672	90231301	3630	2	.
12	90231301	90239718	8418	2	.
12	90239718	90240042	325	2	.
12	90240042	90325214	85173	2	.
12	90325214	90335488	10275	2	.
12	90335488	90356932	21445	3	.
12	90356932	90393417	36486	3	.
12	90393417	90394235	819	3	.
12	90394235	90397636	3402	3	.
12	90397636	90398629	994	4	.
12	90398629	90398785	157	5	.
12	90398785	90399181	397	3	.
12	90399181	90412126	12946	3	.
12	90412126	90425843	13718	3	.
12	90425843	90459955	34113	3	*SOX1*
12	90459955	90478469	18515	3	*SOX1*
12	90478469	90483375	4907	3	*LOC100852089*
12	90483375	90517367	33993	3	*LOC100852089*
12	90517367	90517773	407	3	.
12	90517773	90518065	293	4	.
12	90518065	90526217	8153	3	.
12	90526217	90534690	8474	3	.
12	90534690	90535785	1096	4	.
12	90535785	90574783	38999	3	.
12	90574783	90588530	13748	3	.
12	90588530	90591615	3086	3	.
12	90591615	90597956	6342	3	.
12	90597956	90598123	168	4	.
12	90598123	90630377	32255	3	*SPACA7 TUBGCP3*
12	90630377	90642641	12265	3	*TUBGCP3*
12	90642641	90690404	47764	3	*LOC104973680 TUBGCP3*
12	90690404	90691111	708	3	*LOC104973680*
12	90691111	90703265	12155	3	*LOC104973680*
12	90703265	90706538	3274	3	.
12	90706538	90712146	5609	3	.
12	90712146	90714860	2715	2	.
12	90714860	90722955	8096	2	.
13	1213601	1221686	8086	2	*PLCB1*
13	1221686	1259118	37433	2	*PLCB1*
13	1259118	1296348	37231	2	*PLCB1*
13	1296348	1368940	72593	2	*PLCB1*
13	1368940	1485557	116618	2	*MIR2285M-1 PLCB1*
13	1485557	1489035	3479	3	*PLCB1*
13	1489035	1511332	22298	3	*PLCB1*
13	1511332	1582165	70834	5	*PLCB1*
13	1582165	1608212	26048	5	*PLCB1*
13	1608212	1624849	16638	5	*PLCB1*
13	1624849	1630911	6063	5	*PLCB1*
13	1630911	1648133	17223	4	*PLCB1*
13	1648133	1683212	35080	3	*PLCB1*
13	1683212	1683930	719	3	*PLCB1*
13	1683930	1773122	89193	3	*PLCB1*
13	1773122	1773355	234	4	.
13	1773355	1806641	33287	3	.
13	1806641	1848482	41842	3	.
13	1848482	1863072	14591	3	.
13	1863072	1915589	52518	3	.
13	1915589	1965025	49437	3	.
13	1965025	1966105	1081	3	.
13	1966105	1984515	18411	3	.
13	1984515	1993557	9043	3	.
13	1993557	2022227	28671	3	.
13	2022227	2022949	723	3	.
13	2022949	2036423	13475	3	.
13	2036423	2119832	83410	2	.
13	2119832	2123245	3414	2	*LOC107133021*
13	2123245	2132048	8804	2	*LOC107133021*
13	2132048	2152577	20530	2	*LOC107133021*

13	2152577	2195316	42740	2	.
13	2195316	2221349	26034	2	.
13	2221349	2254340	32992	4	*PLCB4*
13	2254340	2293290	38951	4	*PLCB4*
13	2293290	2295790	2501	4	*PLCB4*
13	2295790	2344013	48224	4	*PLCB4*
13	2344013	2361424	17412	3	*PLCB4*
13	2361424	2363134	1711	3	*PLCB4*
13	2363134	2382502	19369	3	*PLCB4*
13	2382502	2383537	1036	4	*PLCB4*
13	2383537	2402674	19138	3	*PLCB4*
13	2402674	2412938	10265	3	*PLCB4*
13	2412938	2448431	35494	3	*PLCB4*
13	2448431	2485562	37132	3	*LAMP5*
13	2485562	2493843	8282	3	*PAK7*
13	2493843	2502720	8878	3	*PAK7*
13	2502720	2503465	746	5	*PAK7*
13	2503465	2503923	459	4	*PAK7*
13	2503923	2518726	14804	2	*PAK7*
13	2518726	2551367	32642	2	*PAK7*
13	2551367	2577880	26514	2	*MGC148692*
13	2577880	2595791	17912	2	.
13	2595791	2658625	62835	3	*LOC104973690*
13	2658625	2745880	87256	3	*LOC104973690*
13	2745880	2816505	70626	3	*LOC104973690*
13	2816505	2860725	44221	3	*LOC104973690*
13	2860725	2896751	36027	4	*LOC104973690*
13	2896751	2901799	5049	4	*LOC104973690*
13	2901799	2967947	66149	4	*LOC104973690 LOC783610*
13	2967947	2974611	6665	4	.
13	2974611	3042196	67586	4	.
13	3042196	3099897	57702	5	*LOC100848820*
13	3099897	3145736	45840	5	.
13	3145736	3146885	1150	5	.
13	3146885	3265900	119016	5	*ANKEF1*
13	3265900	3268583	2684	5	.
13	3268583	3271834	3252	5	.
13	3271834	3274583	2750	4	.
13	3274583	3287390	12808	4	.
13	3287390	3315522	28133	4	.
13	3315522	3356491	40970	4	.
13	3356491	3375175	18685	4	.
13	3375175	3447059	71885	4	*SNAP25*
13	3447059	3450567	3509	3	*SNAP25*
13	3450567	3511948	61382	3	*SNAP25*
13	3511948	3526752	14805	3	.
13	3526752	3535203	8452	3	.
13	3535203	3535988	786	4	.
13	3535988	3536323	336	3	.
13	3536323	3549188	12866	3	.
13	3549188	3673770	124583	3	*MKKS SLX4IP*
13	3673770	3674476	707	2	*SLX4IP*
13	3674476	3692788	18313	3	*SLX4IP*
13	3692788	3696158	3371	3	*SLX4IP*
13	3696158	3722113	25956	3	*SLX4IP*
13	3722113	3734628	12516	3	*SLX4IP*
13	3734628	3746919	12292	3	*SLX4IP*
13	3746919	3786349	39431	3	*SLX4IP*
13	3786349	3790198	3850	2	*SLX4IP*
13	3790198	3906900	116703	3	*JAG1 LOC107133024 SLX4IP*
13	3906900	3974951	68052	3	.
13	3974951	3997987	23037	4	.
13	3997987	4031425	33439	3	.
13	4031425	4034667	3243	3	.
13	4034667	4097575	62909	3	*LOC101901915*
13	4097575	4099119	1545	3	*LOC101901915*
13	4099119	4303176	204058	3	*LOC101901915*
13	4303176	4305955	2780	3	.
13	4305955	4375705	69751	3	*LOC104973695*
13	4375705	4438070	62366	5	.
13	4438070	4520341	82272	5	.
13	4520341	4535920	15580	5	.
13	4535920	4536849	930	6	.
13	4536849	4551670	14822	5	.
13	4551670	4582200	30531	6	.
13	4582200	4583755	1556	6	.
13	4583755	4584695	941	6	.
13	4584695	4593220	8526	5	.
13	4593220	4610411	17192	5	.
13	4610411	4627870	17460	4	.
13	4627870	4630692	2823	5	.
13	4630692	4631609	918	4	.
13	4631609	4667054	35446	4	.
13	4667054	4679888	12835	4	.
13	4679888	4695316	15429	4	.
13	4695316	4714244	18929	4	.
13	4714244	4721456	7213	4	.
13	4721456	4723810	2355	3	.
13	4723810	4734314	10505	3	.
13	4734314	4757557	23244	4	.
13	4757557	4920647	163091	4	*LOC101902229*

13	4920647	4923453	2807	4	.
13	4923453	4959703	36251	4	.
13	4959703	4968340	8638	4	.
13	4968340	4992364	24025	5	.
13	4992364	5021163	28800	5	.
13	5021163	5027195	6033	5	.
13	5027195	5053560	26366	5	.
13	5053560	5071077	17518	5	.
13	5071077	5084825	13749	5	.
13	5084825	5142618	57794	5	.
13	5142618	5151129	8512	5	.
13	5151129	5159424	8296	5	.
13	5159424	5224823	65400	4	.
13	5224823	5275685	50863	3	.
13	5275685	5277206	1522	3	.
13	5277206	5277383	178	3	.
13	5277383	5278615	1233	3	.
13	5278615	5361778	83164	3	.
13	5361778	5636886	275109	3	*BTBD3 SRY*
13	5636886	5674788	37903	2	*BTBD3*
13	5674788	5679287	4500	2	.
13	5679287	5828640	149354	2	*LOC104973698*
13	5828640	5891502	62863	3	.
13	5891502	5923491	31990	4	.
13	5923491	5968879	45389	4	*LOC107133022*
13	5968879	6002950	34072	4	.
13	6002950	6035591	32642	4	.
13	6035591	6053030	17440	4	.
13	6053030	6055122	2093	4	.
13	6055122	6097079	41958	4	.
13	6097079	6128968	31890	4	.
13	6128968	6152871	23904	4	.
13	6152871	6187497	34627	4	.
13	6187497	6194645	7149	4	.
13	6194645	6209996	15352	3	.
13	6209996	6223937	13942	3	.
13	6223937	6264085	40149	3	.
13	6264085	6293737	29653	3	.
13	6293737	6334452	40716	3	.
13	6334452	6433842	99391	3	.
13	6433842	6457291	23450	3	.
13	6457291	6496990	39700	3	.
13	6496990	6647755	150766	3	*LOC107133023*
13	6647755	6690331	42577	3	*LOC107133023*
13	6690331	6734185	43855	3	.
13	6734185	6745492	11308	3	.
13	6745492	6841374	95883	3	*LOC107133025 SPTLC3*
13	6841374	6874944	33571	3	*SPTLC3*
13	6874944	6932785	57842	2	*SPTLC3*
13	6932785	6996843	64059	2	*ISM1 SPTLC3*
13	6996843	7051927	55085	2	*ISM1*
13	7051927	7052707	781	2	*ISM1*
13	7052707	7098584	45878	2	*ISM1*
13	7098584	7100258	1675	2	.
13	7100258	7167553	67296	2	*TASP1*
14	33359502	33670520	311019	2	*ARFGEF1 COPS5 CSPP1 PPP1R42 TCF24*
14	33670520	33894951	224432	2	*ARFGEF1 CPA6 LOC101902584 TRNAC-GCA*
14	33894951	34063319	168369	2	*CPA6*
14	34063319	34425945	362627	2	*CPA6 PREX2*
14	34425945	34545262	119318	3	*PREX2*
14	34545262	34581708	36447	2	*PREX2*
14	34581708	34630905	49198	2	*LOC100337031*
14	56805392	57883901	1078510	3	*EBAG9 ENY2 KCNV1 LOC104974087 LOC107133125 LOC521950 LOC785158 NUDCD1 PKHD1L1 SYBU TRHR*
14	57883901	57934078	50178	2	*LOC104974087*
14	57934078	58007882	73805	2	.
14	62247363	62638376	391014	2	*LRP12*
14	62878562	63404244	525683	2	*RIMS2*
14	63404244	63593594	189351	2	*DCAF13 LOC104974100 LOC107133127 SLC25A32*
14	63593594	63747831	154238	3	*CTHRC1 FZD6 LOC104974101 LOC783954 SLC25A32*
14	63747831	63858038	110208	2	*BAALC*
14	63858038	64133767	275730	2	*ATP6V1C1 AZIN1 BAALC LOC104974103 LOC107133126 TRNAS-GGA*
14	64133767	64283925	150159	2	*LOC107133128 TRNAE-UUC*
14	64283925	64724867	440943	3	*KLF10 LOC104974106 LOC104974107 LOC107133129 ODF1 RRM2B UBR5*
14	64724867	64896329	171463	3	.
14	64896329	65212623	316295	3	*NCALD*
14	78270424	78473275	202852	2	*CNBD1 LOC100137877 LOC783984*
14	78473275	78717249	243975	3	*CNBD1*
14	78717249	78902799	185551	3	*CNGB3 CPNE3*
14	78902799	79039230	136432	3	*RMDN1 WWP1*
14	79039230	79102153	62924	4	*LOC104974135 WWP1*
14	79102153	79610042	507890	4	*ATP6V0D2 LOC100138079 LOC101903860 LOC104974136 MIR2311 PSKH2 SLC7A13 TRNAS-GGA*
14	79610042	79953854	343813	3	*CA1 CA2 CA3 LOC100196897 LOC104974137 LOC107133131 LOC784254*
14	79953854	79982734	28881	2	.
14	79982734	80211584	228851	3	*C14H8orf59 CA13 E2F5 LOC100847874 LRRCC1*
14	80211584	80386909	175326	3	*LOC107133132 LOC506670 LOC518422 LOC787733 RALYL*
14	80386909	80876099	489191	4	*RALYL*
14	81500845	81700029	199185	3	*TRNAS-GGA*
14	81700029	81751649	51621	4	.

14	81751649	81827228	75580	4	.
14	81827228	81931836	104609	4	*LOC100138329*
14	81931836	82008428	76593	4	.
14	82008428	82209714	201287	4	.
14	82209714	82459104	249391	3	.
14	82459104	82581008	121905	4	.
14	82581008	82697711	116704	3	.
14	82697711	82756524	58814	3	.
14	82756524	82881023	124500	3	.
14	82881023	83058730	177708	2	.
15	1888766	2607577	718812	2	*GRIA4 KBTBD3 LOC104974155 LOC104974161 MSANTD4*
15	2607577	2925506	317930	2	*GRIA4*
15	2925506	3010968	85463	2	.
15	3010968	3748993	738026	2	*CASP1 CASP4 LOC100140955*
15	3748993	3781653	32661	2	.
15	3781653	3843784	62132	2	.
15	3843784	3927888	84105	2	.
15	3927888	4269990	342103	2	.
15	4269990	4359153	89164	2	.
15	4359153	4563203	204051	2	*LOC104974160 PDGFD*
15	4563203	4586707	23505	2	*PDGFD*
15	4586707	5078465	491759	2	*DDI1 LOC104974158 PDGFD*
15	5078465	6174006	1095542	2	*DCUN1D5 DYNC2H1 LOC100138231 MMP1 MMP12 MMP13 MMP3 TRNAY-AUA*
15	6174006	6187106	13101	2	*LOC107131204 MMP1*
15	7655244	7857693	202450	3	*TRPC6*
15	7857693	7997685	139993	4	*LOC787452*
15	7997685	8062538	64854	4	.
15	8062538	8162827	100290	4	.
15	8162827	8228964	66138	4	*LOC107133146 PGR*
15	8228964	8783088	554125	3	*ARHGAP42 LOC107133147 PGR*
15	8783088	8978532	195445	3	*LOC101903693*
15	8978532	9162231	183700	3	*LOC782668*
15	9162231	9249998	87768	3	.
15	9249998	9830178	580181	3	*CNTN5 LOC107133148*
15	13447809	13996183	548375	2	*LOC100300167 TRNAW-CCA*
15	13996183	14316746	320564	2	*CCDC82 JRKL LOC101905762 LOC783497 MAML2 MIR1260B*
15	14316746	15321327	1004582	2	*CEP57 FAM76B LOC101905912 MAML2 MTMR2*
15	15321327	16222714	901388	2	*AMOTL1 CWC15 ENDOD1 LOC100295831 LOC101906086 LOC104974176 LOC107133150 LOC524768 LOC617971 SESN3*
15	16222714	16322141	99428	2	*PIWIL4*
15	16322141	16894242	572102	2	*C15H11orf97 CWF19L2 FUT4 GUCY1A2 LOC107133149*
15	17386117	17819886	433770	2	*ALKBH8 ELMOD1 LOC107133151 SLC35F2 SLN*
15	17819886	18101287	281402	2	*CUL5 LOC107133152 RAB39A SLC35F2*
15	18101287	18365033	263747	2	*ACAT1 ATM CUL5 NPAT*
15	18365033	18477897	112865	2	*ATM C15H11orf65*
15	18477897	18697606	219710	2	*C15H11orf65 EXPH5 KDELC2 LOC101902366*
15	18697606	18706947	9342	2	*EXPH5*
15	18706947	20055547	1348601	2	*C15H11orf87 DDX10 EXPH5 LOC786394*
15	20055547	20175016	119470	2	.
15	20175016	20723480	548465	2	*LOC104974178 LOC783106 LOC784944 TRNAR-CCU ZC3H12C*
15	20723480	21161982	438503	2	*ARHGAP20 FDX1 LOC100847607 LOC782951 RDX*
15	21161982	21625691	463710	2	*ARHGAP20 LOC783349*
15	21625691	21693863	68173	2	.
15	21693863	21900494	206632	2	.
15	21900494	22192784	292291	2	*C15H11orf53 COLCA2 POU2AF1*
15	22192784	22695068	502285	2	*ALG9 BTG4 C15H11orf1 C15H11orf88 FDXACB1 LAYN LOC101906330 MIR34B MIR34C PPP2R1B SIK2 TRNAE-CUC*
15	22695068	22988949	293882	2	*BCO2 C15H11orf1 C15H11orf52 C15H11orf57 CRYAB DIXDC1 DLAT HSPB2 IL18 LOC101903059 LOC101903125 LOC101906594 LOC104968480 LOC104974189 PIH1D2 SDHD TEX12 TIMM8B*
15	22988949	23101598	112650	2	*BCO2 PTS*
15	23101598	25651730	2550133	2	*ANKK1 C15H11orf34 C15H11orf71 CLDN25 DRD2 FAM55A HTR3A HTR3B LOC100847772 LOC101903190 LOC101907641 LOC104974191 LOC107133153 LOC511161 LOC781913 LOC782610 LOC787274 NCAM1 NXPE2 NXPE4 RBM7 REXO2 TMPRSS5 TRNAG-CCC TTC12 USP28 ZBTB16 ZW10*
15	25651730	25711833	60104	2	*LOC510786 NXPE2*
15	25711833	26775912	1064080	2	*CADM1 LOC101908225 LOC104974202*
15	26775912	26967631	191720	2	*LOC101901976 LOC101902024*
15	65782970	65865397	82428	2	*ABTB2 CAPRIN1 NAT10*
15	65865397	66008423	143027	2	*ABTB2*
15	66008423	66275787	267365	2	*ABTB2 CAT ELF5 LOC104974297*
15	75442069	75957657	515589	2	*ALX4 CD82 EXT2 LOC100337430 LOC104974319*
15	75957657	76216466	258810	2	*LOC107133185 TP53I11*
16	66193221	66303668	110448	2	*NCF2 SMG7*
16	79845738	79909739	64002	2	*LOC104974541 PTPRC*
16	79909739	80542590	632852	2	*LOC104974542 LOC104974544 LOC104974545 LOC107133267 MIR181A-1 MIR181B-1*
16	80542590	80685804	143215	2	*LOC101904123*
16	80685804	80710541	24738	2	.
16	80710541	80810722	100182	3	.
16	80810722	81073948	263227	3	*NR5A2*
16	81073948	81195665	121718	3	*NR5A2*
16	81195665	81304555	108891	2	*ZNF281*
17	4542981	4799440	256460	2	*ARFIP1 FHDC1 LOC104976512*
17	4799440	4820191	20752	2	*ARFIP1*
17	4820191	4892814	72624	2	*ARFIP1 TIGD4*
17	4892814	5012602	119789	2	*LOC104974562 LOC107133272 TIGD4 TMEM154*
17	5012602	5091918	79317	3	*TMEM154*

17	5091918	5127903	35986	3	*TRNAY-GUA*
17	5127903	5157927	30025	3	*FBXW7*
17	5157927	5276235	118309	3	*FBXW7*
17	5276235	5408319	132085	2	*FBXW7*
17	9202967	9357096	154130	4	*LOC104974568*
17	9357096	9423678	66583	4	*LOC104974568*
17	9423678	9512216	88539	4	*LOC104974568*
17	9512216	9525507	13292	4	.
17	9525507	9604580	79074	4	.
17	9604580	9622570	17991	2	.
17	71804664	71845465	40802	3	*LOC101906447 LOC619125 SEC14L4*
17	71845465	71879837	34373	3	*GAL3ST1 PES1 TRNAS-GGA*
17	71879837	71994178	114342	4	*C17H5orf52 DUSP18 OSBP2 PES1 SLC35E4 TCN2*
17	71994178	72030759	36582	4	*OSBP2*
17	72030759	72154080	123322	4	*MORC2 OSBP2*
17	72154080	72248991	94912	4	*SMTN TUG1*
17	72248991	72469197	220207	4	*DRG1 INPP5J LIMK2 LOC101903756 PATZ1 PIK3IP1 PLA2G3 RNF185 SELM SMTN*
17	72469197	72710006	240810	4	*DEPDC5 DRG1 EIF4ENIF1 LOC104974705 PISD PRR14L SFI1*
17	72716768	72813393	96626	4	*DEPDC5*
17	72813393	72888505	75113	4	*SLC5A1 YWHAH*
18	60943728	60973450	29723	4	*LOC101907390 LOC104968479*
18	60973450	61236243	262794	6	*LOC100336208 LOC100336431 LOC100336448 LOC100849068 LOC101902722 LOC101903016 LOC101906283 LOC101906367 LOC101907041 LOC101907390 LOC101909040 LOC104970711 LOC104976972 LOC107133353 LOC618456 LOC788268 LOC788405 LOC788928 ZNF679*
18	61236243	61351842	115600	6	*LOC100139104 LOC504861 ZNF331 ZNF679*
18	61351842	61369952	18111	6	*NLRP12*
18	61369952	61415205	45254	6	*LOC532048 NLRP12*
18	61415205	61507521	92317	6	*LOC107131459 LOC789229 MGC139164 MIR371 NLRP12*
18	61507521	61538180	30660	6	*LOC504704 LOC506868*
18	61538180	61583660	45481	6	*LOC515600 LOC615600 MGC157082*
18	61583660	61601509	17850	7	*LOC784517 MGC157082*
18	61601509	61764023	162515	7	*LOC101904080 LOC101904151 LOC511982 LOC513941 LOC527385 LOC784517 LOC785919 MGC157082*
18	61764023	61842496	78474	7	*LOC517090 LOC528568 LOC618873*
18	61842496	61960159	117664	7	*LOC107131285 LOC510904 LOC528332 LOC617141 MGC138914*
18	61960159	62170418	210260	7	*LOC100300449 LOC100300531 LOC100336759 LOC100337097 LOC101907900 LOC510904 LOC514552 LOC518964 LOC525820*
18	62170418	62386153	215736	6	*CACNG7 LOC100848932 LOC101904722 LOC101904797 LOC101904855 LOC104974943 LOC538810 LOC618662 LOC788745 LOC789418 MYADM PRKCG*
20	32104262	32460457	356196	2	*GHR LOC104975266 LOC104975269*
20	32460457	32663938	203482	2	.
20	32663938	32839701	175764	2	*FBXO4 OXCT1*
20	32839701	33238463	398763	2	*OXCT1 PLCXD3 TRNAY-GUA*
22	27695490	27787199	91710	2	.
22	27787199	28243694	456496	3	.
22	28243694	28480244	236551	2	*LOC104975536 PDZRN3*
22	28480244	28781676	301433	2	*PDZRN3*
23	25397164	25854966	457803	3	*BOLA-DQA1 BOLA-DQA2 BOLA-DQA5 BOLA-DQB BOLA-DRA BOLA-DRB2 BOLA-DRB3 BTNL2 LOC100848815 LOC100851058 LOC101902890 LOC101903077 LOC504295 LOC525599 LOC786695*
23	25854966	26184884	329919	2	*BLA-DQB BTNL2 LOC101903077 LOC101903155 LOC101903211 LOC101908667 LOC104969893 LOC104975658 LOC783151*
23	27825007	29802406	1977400	2	*ABCF1 ATAT1 BOLA BOLA-NC1 C23H6orf136 C23H6orf15 CCHCR1 CDSN DDR1 DHX16 DPCR1 FLOT1 GABBR1 GNL1 GTF2H4 IER3 JSP.1 LOC100141101 LOC100295296 LOC100300439 LOC100847761 LOC101904511 LOC101905956 LOC101906024 LOC101906691 LOC101907137 LOC104975665 LOC104975666 LOC104975667 LOC104975670 LOC107131305 LOC107131733 LOC107131734 LOC107131735 LOC107131736 LOC107131737 LOC107131738 LOC107133389 LOC107133391 LOC504548 LOC506486 LOC509155 LOC509280 LOC510530 LOC511103 LOC512579 LOC512672 LOC514434 LOC515704 LOC516273 LOC516274 LOC523389 LOC523768 LOC523769 LOC526288 LOC528343 LOC614091 LOC615278 LOC616306 LOC616517 LOC616942 LOC618034 LOC618052 LOC618064 LOC618070 LOC618140 LOC619014 LOC782262 LOC782301 LOC782341 LOC782379 LOC784557 LOC784614 LOC784652 LOC784681 LOC784787 LOC784858 LOC785162 LOC785252 LOC785277 LOC785431 LOC785479 LOC785582 LOC785639 LOC785672 LOC785712 LOC785747 LOC785760 LOC785779 LOC785811 LOC785832 LOC785858 LOC785873 LOC785884 LOC785910 LOC786846 LOC786987 LOC787188 LOC788663 LOC788675 LOC789358 LOC789367 LOC789690 MDC1 MIR2378 MIR877 MOG MRPS18B MUC21 NRM OR10C1 OR12D2 OR12D3 OR14J1 OR2H1 OR2J2 OR2J3 OR2W1 OR5V1 POU5F1 PPP1R10 PPP1R11 PPP1R18 PRR3 PSORS1C2 RNF39 RPP21 SFTA2 TCF19 TRIM10 TRIM15 TRIM31 TRIM40 TRNAG-CCC TRNAS-CGA TUBB UBD VARS2 ZFP57 ZNRD1*
23	29802406	30512304	709899	2	*LOC100847345 LOC101909685 LOC104970719 LOC104975673 LOC107131411 LOC107131741 LOC528914 LOC529518 LOC532291 LOC617979 LOC782475 LOC782554 LOC784131 NKAPL PGBD1 TRIM27 TRNAA-AGC TRNAA-CGC TRNAA-UGC TRNAE-CUC TRNAF-GAA TRNAK-UUU TRNAL-AAG TRNAL-CAA TRNAM-CAU TRNAQ-CUG TRNAQ-UUG TRNAR-CCG TRNAS-GCU TRNAT-AGU TRNAT-CGU ZKSCAN4 ZKSCAN8 ZNF165 ZNF311 ZNF389 ZSCAN12 ZSCAN16 ZSCAN26 ZSCAN31 ZSCAN9*

24	6698234	7285114	586881	2	*LOC101907394 LOC104975731*
24	7285114	7292705	7592	2	.
24	47215620	47607020	391401	2	*HDHD2 IER3IP1 KATNAL2 LOC100847542 LOC104975783 LOC783504 LOC785129 PIAS2 SKOR2*
24	47607020	48007916	400897	2	.
24	48007916	49525735	1517820	2	*CTIF DYM LOC101904605 LOC104969950 LOC107131402 LOC107133399 LOC786409 SMAD2 SMAD7 ZBTB7C*
24	49525735	49901454	375720	2	*C24H18orf32 DYM LOC107131298 LOC509808 MIR2381 RPL17*
24	49901454	49926879	25426	2	.
24	49926879	50068182	141304	2	*LOC107131789*
24	50068182	55599646	5531465	2	*ACAA2 C24H18orf54 CCDC68 CFAP53 CXXC1 DCC ELAC1 LOC100137989 LOC101904580 LOC104972848 LOC104975792 LOC104975794 LOC104975818 LOC107131794 LOC107131795 LOC789119 MAPK4 MBD1 MBD2 ME2 MEX3C MRO MYO5B POLI RAB27B SKA1 SMAD4 STARD6 TCF4 TRNAC-GCA*
24	55599646	57079493	1479848	2	*LOC101906559 LOC101906657 LOC104975797 LOC104975798 LOC104975799 LOC107131796 TXNL1 WDR7*
24	57079493	57938545	859053	2	*ATP8B1 FECH LOC107131797 NARS NEDD4L ONECUT2 ST8SIA3 TRNAE-CUC*
24	60032599	61613864	1581266	2	*CDH20 KIAA1468 LOC101902622 LOC101903157 LOC104975811 LOC107131802 LOC783926 PIGN RNF152 TNFRSF11A TRNAC-GCA ZCCHC2*
24	62574040	62641660	67621	2	*LOC100337223 LOC107131200 LOC107131803 LOC519132 LOC786348 LOC786410*
25	8189597	8190031	435	2	.
27	15857929	15860219	2291	2	.
27	15936317	15936511	195	2	.
27	15975850	15979401	3552	2	.
27	16017303	16017952	650	2	.
27	16187774	16188159	386	2	*LOC107131908*
27	16288455	16289066	612	2	*LOC101905556*
27	16289066	16289846	781	2	*LOC101905556*
27	16289846	16290290	445	2	*LOC101905556*
27	16487704	16497432	9729	2	.
27	16497432	16505579	8148	2	.
27	16505579	16507368	1790	2	.
27	16507368	16513357	5990	2	.
27	16739322	16739775	454	2	*ZFP42*
27	16739775	16744892	5118	2	.
27	16744892	16753615	8724	2	*TRNAG-UCC*
27	16753615	16755172	1558	2	.
27	16781395	16781599	205	2	.
27	16831698	16832220	523	2	.
27	16832220	16832828	609	2	.
27	16854438	16854695	258	2	.
27	17486656	17499388	12733	2	.
27	17499388	17510848	11461	2	.
27	17511069	17511839	771	2	.
27	17511839	17528052	16214	2	.
27	17528052	17529460	1409	3	.
27	17529460	17543551	14092	2	.
27	17543551	17543921	371	2	.
27	17543921	17545038	1118	2	.
27	19247072	19250159	3088	3	*ZDHHC2*
27	19250159	19265034	14876	3	*ZDHHC2*
27	19265034	19276157	11124	3	*ZDHHC2*
27	19276157	19282867	6711	2	.
27	19282867	19292339	9473	3	.
27	19292339	19294203	1865	4	.
27	19294203	19298215	4013	3	*MICU3*
27	19298215	19309522	11308	3	*MICU3*
27	19309522	19312361	2840	3	*MICU3*
27	19312361	19314441	2081	3	*MICU3*
27	19314441	19317131	2691	3	*MICU3*
27	19317131	19317896	766	3	*MICU3*
27	19317896	19331537	13642	3	*MICU3*
27	19331537	19346031	14495	4	*MICU3*
27	19346031	19349512	3482	3	*MICU3*
27	19349512	19349875	364	3	*MICU3*
27	19349875	19350814	940	3	*MICU3*
27	19350814	19366289	15476	3	*MICU3*
27	19366289	19370385	4097	3	*MICU3*
27	19370385	19374221	3837	3	.
27	19374221	19382214	7994	3	.
27	19382214	19407739	25526	3	*FGF20*
27	19407739	19426163	18425	3	*FGF20*
27	19426163	19429020	2858	3	.
27	19429020	19431732	2713	3	.
27	19431732	19436240	4509	3	.
27	19436240	19437619	1380	3	.
27	19437619	19438902	1284	4	.
27	19438902	19440182	1281	3	.
27	19440182	19443250	3069	3	.
27	19443250	19443487	238	3	.
27	19443487	19448290	4804	3	.
27	19448290	19450042	1753	4	.
27	19450042	19465709	15668	3	.
27	19465709	19469083	3375	3	.
27	19469083	19469823	741	3	.
27	19469823	19485723	15901	3	.
27	19485723	19485970	248	4	.
27	19485970	19492376	6407	3	.
27	19492376	19495709	3334	3	.
27	19495709	19498754	3046	3	.

27	19498754	19501106	2353	3	.
27	19501106	19511302	10197	3	.
27	19511302	19515767	4466	3	.
27	19515767	19522723	6957	3	.
27	19522723	19532708	9986	3	.
27	19532708	19540146	7439	4	.
27	19540146	19545383	5238	4	.
27	19545383	19548037	2655	2	.
27	19548037	19554939	6903	3	.
27	19554939	19563556	8618	4	.
27	19563556	19571607	8052	3	.
27	19571607	19581225	9619	3	.
27	19581225	19583013	1789	4	.
27	19583013	19583110	98	3	.
27	19583110	19589274	6165	3	.
27	19589274	19593031	3758	3	.
27	19593031	19593903	873	4	.
27	19593903	19595039	1137	3	.
27	19595039	19596380	1342	2	.
27	19596380	19596858	479	2	.
27	19596858	19597175	318	3	.
27	19597175	19600251	3077	3	.
27	19600251	19603191	2941	3	.
27	19603191	19604513	1323	2	.
27	19604513	19605560	1048	2	.
27	19605560	19607438	1879	3	.
27	19607438	19620419	12982	3	.
27	19620419	19623017	2599	2	.
27	19623017	19625843	2827	3	.
27	19625843	19627870	2028	3	.
27	19627870	19629236	1367	3	.
27	19629236	19641925	12690	3	.
27	19641925	19642190	266	3	.
27	19642190	19642900	711	3	.
27	19642900	19652044	9145	3	.
27	19652044	19653033	990	3	.
27	19653033	19656046	3014	3	.
27	19656046	19666279	10234	2	.
27	19671327	19672124	798	2	.
27	19672124	19675950	3827	3	.
27	19675950	19682238	6289	4	.
27	19682238	19682600	363	4	.
27	19682600	19685471	2872	4	.
27	19685471	19687677	2207	4	.
27	19687677	19690080	2404	3	.
27	19690080	19692250	2171	3	.
27	19692250	19695070	2821	3	.
27	19695070	19697202	2133	3	.
27	19697202	19702059	4858	3	.
27	19702059	19702912	854	4	.
27	19702912	19717340	14429	3	.
27	19717340	19720663	3324	3	.
27	19720663	19721712	1050	3	.
27	19721712	19726110	4399	3	.
27	19726110	19727064	955	3	.
27	19727064	19727460	397	3	.
27	19727460	19740024	12565	4	.
27	19740024	19744447	4424	4	.
27	19744447	19751180	6734	3	.
27	19751180	19752027	848	3	.
27	19752027	19754320	2294	3	.
27	19754320	19758312	3993	3	.
27	19758312	19759796	1485	3	.
27	19759796	19760800	1005	3	.
27	19760800	19761887	1088	3	.
27	19761887	19763311	1425	3	.
27	19763311	19767434	4124	3	.
27	19767434	19770351	2918	3	.
27	19770351	19777287	6937	3	.
27	19777287	19778149	863	3	.
27	19778149	19778451	303	3	.
27	19778451	19785623	7173	2	.
27	19785623	19794354	8732	2	.
27	19794354	19794684	331	2	.
27	19794684	19795310	627	2	.
27	19795310	19796467	1158	2	.
27	19796467	19798537	2071	2	.
27	19798724	19802894	4171	2	.
27	19802894	19803108	215	2	.
27	19803108	19803866	759	2	.
27	19803866	19804394	529	2	.
27	19804394	19805080	687	2	.
27	19805080	19805923	844	2	.
27	19805923	19811664	5742	2	.
27	19811664	19815404	3741	2	.
27	19815404	19815820	417	2	.
27	19815820	19816389	570	3	.
27	19816389	19817045	657	2	.
27	19817045	19824305	7261	2	.
27	19824305	19825088	784	2	.
27	19825088	19842445	17358	3	.

27	19842445	19842880	436	3	.
27	19842880	19845295	2416	3	*LOC104976064*
27	19845295	19846870	1576	2	*LOC104976064*
27	25295114	25295481	368	2	.
27	25295481	25297362	1882	5	.
27	25297362	25319494	22133	5	.
27	25319494	25319863	370	5	.
27	25319863	25321573	1711	4	.
27	25321573	25324375	2803	5	.
27	25324375	25326920	2546	5	.
27	25326920	25327064	145	4	.
27	25327064	25331706	4643	5	.
27	25331706	25337592	5887	5	.
27	25337592	25338580	989	5	.
27	25338580	25338978	399	5	.
27	25338978	25339727	750	5	.
27	25339727	25351574	11848	5	.
27	25351574	25379257	27684	5	.
27	25379257	25380607	1351	5	.
27	25380607	25380885	279	5	.
27	25380885	25385023	4139	5	.
27	25385023	25385805	783	6	.
27	25385805	25388277	2473	6	.
27	25388277	25391139	2863	6	.
27	25391139	25393988	2850	6	.
27	25393988	25399911	5924	6	.
27	25399911	25401686	1776	6	.
27	25401686	25405659	3974	5	.
27	25405659	25407112	1454	5	.
27	25407112	25408474	1363	5	.
27	25408474	25409196	723	5	.
27	25409196	25410735	1540	6	.
27	25410735	25420742	10008	5	.
27	25420742	25423748	3007	5	.
27	25423748	25425313	1566	6	.
27	25425313	25427534	2222	5	.
27	25427534	25427862	329	5	.
27	25427862	25436168	8307	5	.
27	25436168	25444931	8764	5	.
27	25444931	25453651	8721	5	.
27	25453651	25455330	1680	5	.
27	25455330	25455877	548	6	.
27	25455877	25463194	7318	5	.
27	25463194	25466008	2815	5	.
27	25466008	25466608	601	5	.
27	25466608	25467220	613	5	.
27	25467220	25467531	312	4	.
27	25467531	25469854	2324	4	.
27	25469854	25470571	718	4	.
27	25470571	25472791	2221	4	.
27	25472791	25481916	9126	5	.
27	25481916	25484006	2091	5	.
27	25484006	25486578	2573	5	.
27	25486578	25490630	4053	4	.
27	25490630	25490803	174	4	.
27	25490803	25492348	1546	4	.
27	25492348	25495480	3133	4	.
27	25495480	25495922	443	5	.
27	25495922	25496067	146	6	.
27	25496067	25503354	7288	5	.
27	25503354	25503676	323	5	.
27	25503676	25506161	2486	5	.
27	25506161	25510257	4097	5	.
27	25510257	25510861	605	6	.
27	25510861	25511184	324	7	.
27	25511184	25512887	1704	7	.
27	25512887	25513989	1103	5	.
27	25513989	25517232	3244	5	.
27	25517232	25517451	220	5	.
27	25517451	25519075	1625	5	.
27	25519075	25524431	5357	5	.
27	25524431	25524721	291	5	.
27	25524721	25526214	1494	5	.
27	25526214	25528142	1929	5	.
27	25528142	25543983	15842	5	*SARAF*
27	25543983	25544869	887	5	*SARAF*
27	25544869	25551205	6337	6	*SARAF*
27	25551205	25552776	1572	5	*SARAF*
27	25552776	25553675	900	7	*SARAF*
27	25553675	25554241	567	7	*SARAF*
27	25554241	25555059	819	5	*SARAF*
27	25555059	25555680	622	5	*SARAF*
27	25555680	25560016	4337	5	*SARAF*
27	25560016	25567970	7955	5	*LEPROTL1 SARAF*
27	25567970	25568454	485	5	*LEPROTL1*
27	25568454	25568890	437	5	*LEPROTL1*
27	25568890	25569391	502	6	*LEPROTL1*
27	25569391	25569977	587	6	*LEPROTL1*
27	25569977	25573784	3808	6	*LEPROTL1*
27	25573784	25579330	5547	6	*LEPROTL1*
27	25579330	25580413	1084	5	.

27	25580413	25595646	15234	5	*MBOAT4*
27	25595646	25596218	573	5	*MBOAT4*
27	25596218	25599023	2806	5	*MBOAT4*
27	25599023	25600505	1483	4	*MBOAT4*
27	25600505	25603219	2715	5	*MBOAT4*
27	25603219	25604029	811	5	.
27	25604029	25605056	1028	5	.
27	25605056	25605399	344	5	.
27	25605399	25606770	1372	4	.
27	25606770	25612082	5313	5	*DCTN6*
27	25612082	25612533	452	5	*DCTN6*
27	25612533	25613815	1283	5	*DCTN6*
27	25613815	25614037	223	6	*DCTN6*
27	25614037	25614229	193	5	*DCTN6*
27	25614229	25614653	425	5	*DCTN6*
27	25614653	25619499	4847	5	*DCTN6*
27	25619499	25619872	374	6	*DCTN6*
27	25619872	25622814	2943	5	*DCTN6*
27	25622814	25623366	553	4	*DCTN6*
27	25623366	25638724	15359	5	*DCTN6*
27	25638724	25638994	271	4	.
27	25638994	25639177	184	5	.
27	25639177	25641080	1904	5	*LOC104976086*
27	25641080	25642192	1113	5	*LOC104976086*
27	25642192	25642988	797	5	*LOC104976086*
27	25642988	25649083	6096	5	*LOC104976086*
27	25649083	25649657	575	5	.
27	25649657	25651968	2312	5	.
27	25651968	25653071	1104	5	.
27	25653071	25661098	8028	5	.
27	25661098	25662051	954	5	.
27	25662051	25663433	1383	5	.
27	25663433	25665415	1983	5	.
27	25665415	25667187	1773	5	.
27	25667187	25668443	1257	5	.
27	25668443	25668964	522	5	.
27	25668964	25678192	9229	5	.
27	25678192	25679304	1113	5	.
27	25679304	25688548	9245	5	.
27	25688548	25689452	905	5	.
27	25689452	25694948	5497	5	.
27	25694948	25695196	249	5	.
27	25695196	25698106	2911	5	.
27	25698106	25698709	604	5	.
27	25698709	25700739	2031	5	*LOC101906081*
27	25700739	25703248	2510	6	*LOC101906081*
27	25703248	25705900	2653	6	*LOC101906081*
27	25705900	25706751	852	5	*LOC101906081*
27	25706751	25710123	3373	5	*LOC101906081*
27	25710123	25711436	1314	5	*LOC101906081*
27	25711436	25713587	2152	5	*LOC101906081*
27	25713587	25714391	805	6	*LOC101906081*
27	25714391	25718458	4068	5	*LOC101906081*
27	25718458	25721586	3129	5	*LOC101906081*
27	25721586	25722473	888	5	*LOC101906081*
27	25722473	25727292	4820	5	*LOC101906081*
27	25727292	25729476	2185	6	.
27	25729476	25730676	1201	5	.
27	25730676	25732588	1913	5	.
27	25732588	25738859	6272	5	.
27	25738859	25739188	330	5	.
27	25739188	25742718	3531	5	.
27	25742718	25745220	2503	5	.
27	25745220	25746192	973	5	.
27	25746192	25748865	2674	5	.
27	25748865	25753771	4907	5	.
27	25753771	25755454	1684	5	.
27	25755454	25758131	2678	5	.
27	25758131	25759444	1314	5	.
27	25759444	25760793	1350	5	.
27	25760793	25766085	5293	5	.
27	25766085	25766588	504	4	.
27	25766588	25767491	904	4	.
27	25767491	25771933	4443	6	.
27	25771933	25777524	5592	5	.
27	25777524	25781093	3570	5	.
27	25781093	25782693	1601	5	*RBPMS*
27	25782693	25784029	1337	5	*RBPMS*
27	25784029	25786041	2013	5	*RBPMS*
27	25786041	25787310	1270	5	*RBPMS*
27	25787310	25789326	2017	5	*RBPMS*
27	25789326	25789930	605	5	*RBPMS*
27	25789930	25795920	5991	5	*RBPMS*
27	25795920	25796400	481	5	*RBPMS*
27	25796400	25798665	2266	5	*RBPMS*
27	25798665	25802510	3846	5	*RBPMS*
27	25802510	25806734	4225	5	*RBPMS*
27	25806734	25807250	517	4	*RBPMS*
27	25807250	25807432	183	5	*RBPMS*
27	25807432	25809219	1788	5	*RBPMS*
27	25809219	25809365	147	5	*RBPMS*

27	25809365	25810363	999	5	*RBPMS*
27	25810363	25811061	699	5	*RBPMS*
27	25811061	25813135	2075	5	*RBPMS*
27	25813135	25816418	3284	5	*RBPMS*
27	25816418	25819989	3572	5	*RBPMS*
27	25819989	25820294	306	5	*RBPMS*
27	25820294	25821452	1159	5	*RBPMS*
27	25821452	25821722	271	5	*RBPMS*
27	25821722	25823257	1536	5	*RBPMS*
27	25823257	25828943	5687	5	*RBPMS*
27	25828943	25829691	749	5	*RBPMS*
27	25829691	25829886	196	5	*RBPMS*
27	25829886	25830634	749	6	*RBPMS*
27	25830634	25831948	1315	5	*RBPMS*
27	25831948	25832470	523	5	*RBPMS*
27	25832470	25838195	5726	5	*RBPMS*
27	25838195	25845915	7721	5	*RBPMS*
27	25845915	25847741	1827	5	*RBPMS*
27	25847741	25849391	1651	6	*RBPMS*
27	25849391	25860013	10623	5	*MIR2399 RBPMS*
27	25860013	25860809	797	4	*RBPMS*
27	25860809	25865434	4626	5	*RBPMS*
27	25865434	25871042	5609	5	*RBPMS*
27	25871042	25871230	189	5	*RBPMS*
27	25871230	25881884	10655	5	*RBPMS*
27	25881884	25892465	10582	5	*RBPMS*
27	25892465	25892979	515	5	*RBPMS*
27	25892979	25894181	1203	4	*RBPMS*
27	25894181	25903305	9125	5	*RBPMS*
27	25903305	25904370	1066	5	*RBPMS*
27	25904370	25904836	467	5	*RBPMS*
27	25904836	25908154	3319	5	*RBPMS*
27	25908154	25911250	3097	5	*RBPMS*
27	25911250	25911362	113	5	*RBPMS*
27	25911362	25913806	2445	5	*RBPMS*
27	25913806	25918471	4666	5	*RBPMS*
27	25918471	25919415	945	6	*RBPMS*
27	25919415	25924288	4874	5	*RBPMS*
27	25924288	25925086	799	6	*RBPMS*
27	25925086	25925269	184	6	*RBPMS*
27	25925269	25926519	1251	5	*RBPMS*
27	25926519	25929942	3424	5	*RBPMS*
27	25929942	25940216	10275	5	*RBPMS*
27	25940216	25946787	6572	5	*RBPMS*
27	25946787	25950472	3686	5	*RBPMS*
27	25950472	25950797	326	6	*RBPMS*
27	25950797	25955185	4389	5	*RBPMS*
27	25955185	25956095	911	5	*RBPMS*
27	25956095	25960913	4819	5	*RBPMS*
27	25960913	25961066	154	5	.
27	25961066	25963312	2247	6	.
27	25963312	25970245	6934	5	.
27	25970245	25971071	827	5	.
27	25971071	25982769	11699	5	.
27	25982769	25982837	69	5	.
27	25982837	25983642	806	5	.
27	25983642	25993976	10335	5	*GTF2E2*
27	25993976	26000840	6865	5	*GTF2E2*
27	26000840	26001044	205	6	*GTF2E2*
27	26001044	26001384	341	7	*GTF2E2*
27	26001384	26002046	663	6	*GTF2E2*
27	26002046	26002446	401	6	*GTF2E2*
27	26002446	26005736	3291	5	*GTF2E2*
27	26005736	26007300	1565	5	*GTF2E2*
27	26007300	26012401	5102	5	*GTF2E2*
27	26012401	26012917	517	4	*GTF2E2*
27	26012917	26014160	1244	5	*GTF2E2*
27	26014160	26014744	585	5	*GTF2E2*
27	26014744	26015892	1149	5	*GTF2E2*
27	26015892	26016070	179	5	*GTF2E2*
27	26016070	26030264	14195	5	*GTF2E2*
27	26030264	26030990	727	5	*GTF2E2*
27	26030990	26035090	4101	5	*GTF2E2*
27	26035090	26035275	186	4	*GTF2E2*
27	26035275	26040751	5477	5	*GTF2E2*
27	26040751	26041412	662	5	*GTF2E2*
27	26041412	26044782	3371	5	*GTF2E2*
27	26044782	26050765	5984	6	*GTF2E2 SMIM18*
27	26050765	26052382	1618	5	*GTF2E2 SMIM18*
27	26052382	26052592	211	5	*GTF2E2 SMIM18*
27	26052592	26052782	191	6	*GTF2E2 SMIM18*
27	26052782	26053007	226	5	*GTF2E2 SMIM18*
27	26053007	26056410	3404	6	*GTF2E2 SMIM18*
27	26056410	26057610	1201	5	*GTF2E2*
27	26057610	26059937	2328	5	*GTF2E2*
27	26059937	26063309	3373	5	*GTF2E2*
27	26063309	26066233	2925	6	*GTF2E2*
27	26066233	26066612	380	5	*GTF2E2*
27	26066612	26081369	14758	5	*GTF2E2*
27	26081369	26081910	542	5	.
27	26081910	26082070	161	4	.

27	26082070	26082978	909	6	.
27	26082978	26092491	9514	6	*GSR*
27	26092491	26093062	572	5	*GSR*
27	26093062	26093424	363	5	*GSR*
27	26093424	26094275	852	5	*GSR*
27	26094275	26094516	242	5	*GSR*
27	26094516	26094677	162	5	*GSR*
27	26094677	26095871	1195	5	*GSR*
27	26095871	26096363	493	5	*GSR*
27	26096363	26101946	5584	5	*GSR*
27	26101946	26102309	364	5	*GSR*
27	26102309	26103251	943	5	*GSR*
27	26103251	26109062	5812	5	*GSR*
27	26109062	26111290	2229	5	*GSR*
27	26111290	26113678	2389	5	*GSR*
27	26113678	26115917	2240	5	*GSR*
27	26115917	26116173	257	4	*GSR*
27	26116173	26116349	177	3	*GSR*
27	26116349	26116646	298	4	*GSR*
27	26116646	26121530	4885	5	*GSR*
27	26121530	26121735	206	5	*GSR*
27	26121735	26122849	1115	4	*GSR*
27	26122849	26126705	3857	4	*GSR*
27	26126705	26132301	5597	4	*GSR*
27	26132301	26132736	436	4	*GSR*
27	26132736	26135351	2616	4	*GSR*
27	26135351	26138456	3106	4	*LOC782955*
27	26138456	26142409	3954	4	*LOC782955*
27	26142409	26144728	2320	5	.
27	26144728	26146139	1412	5	.
27	26146139	26146538	400	5	.
27	26146538	26154308	7771	5	*UBXN8*
27	26154308	26156574	2267	5	*UBXN8*
27	26156574	26160526	3953	5	*UBXN8*
27	26160526	26164925	4400	4	*UBXN8*
27	26164925	26171176	6252	4	*UBXN8*
27	26171176	26172306	1131	4	*UBXN8*
27	26172306	26173360	1055	4	.
27	26173360	26180587	7228	4	.
27	26180587	26181114	528	5	.
27	26181114	26185250	4137	4	.
27	26185250	26188664	3415	4	*PPP2CB*
27	26188664	26191741	3078	4	*PPP2CB*
27	26191741	26193730	1990	4	*PPP2CB*
27	26193730	26195177	1448	5	*PPP2CB*
27	26195177	26197362	2186	4	*PPP2CB*
27	26197362	26204835	7474	4	*PPP2CB*
27	26204835	26209799	4965	4	*PPP2CB*
27	26209799	26211210	1412	4	*PPP2CB*
27	26211210	26213486	2277	4	.
27	26213486	26215051	1566	4	.
27	26215051	26217015	1965	4	.
27	26217015	26230960	13946	4	*TEX15*
27	26230960	26231878	919	4	*TEX15*
27	26231878	26236145	4268	4	*TEX15*
27	26236145	26251936	15792	4	*TEX15*
27	26251936	26262871	10936	4	*TEX15*
27	26262871	26263249	379	5	*TEX15*
27	26263249	26263518	270	6	*TEX15*
27	26263518	26269290	5773	4	*TEX15*
27	26269290	26270583	1294	4	*TEX15*
27	26270583	26270980	398	4	*TEX15*
27	26270980	26281163	10184	4	*TEX15*
27	26281163	26285527	4365	5	*TEX15*
27	26285527	26287247	1721	6	*TEX15*
27	26287247	26287436	190	5	*TEX15*
27	26287436	26289107	1672	6	*TEX15*
27	26289107	26295262	6156	5	*TEX15*
27	26295262	26298970	3709	4	.
27	26298970	26300322	1353	4	.
27	26300322	26302575	2254	4	*LOC107131911*
27	26302575	26312013	9439	4	*LOC107131911*
27	26312013	26314680	2668	4	.
27	26314680	26324299	9620	4	.
27	26324299	26325925	1627	5	.
27	26325925	26328123	2199	4	.
27	26328123	26333889	5767	4	.
27	26333889	26334709	821	3	.
27	26334709	26336952	2244	4	.
27	26336952	26341526	4575	4	.
27	26341526	26342123	598	4	.
27	26342123	26342681	559	4	.
27	26342681	26345283	2603	4	*LOC104976090*
27	26345283	26346432	1150	4	*LOC104976090*
27	26346432	26347700	1269	4	*LOC104976090*
27	26347700	26348418	719	3	*LOC104976090*
27	26348418	26348589	172	2	*LOC104976090*
27	26348589	26348689	101	2	*LOC104976090*
27	38360905	38361292	388	2	.
27	38398324	38401369	3046	2	.
27	38401369	38402179	811	2	.

27	38402179	38403747	1569	2	.
27	38403747	38404581	835	2	.
27	38404581	38404690	110	2	.
27	38404690	38405210	521	2	.
27	38405210	38406204	995	2	.
27	38406204	38408898	2695	2	.
27	38408898	38409298	401	2	.
27	38409298	38411169	1872	2	.
27	38411169	38425877	14709	2	.
27	38425877	38426189	313	2	.
27	38426189	38427510	1322	2	.
27	38427510	38427841	332	2	.
27	38427841	38428673	833	2	.
27	38428673	38429397	725	2	.
27	38429397	38429982	586	2	.
27	38429982	38441026	11045	2	.
27	38441026	38441395	370	2	.
27	38441395	38442830	1436	2	.
27	38442830	38458420	15591	2	.
27	38458420	38459936	1517	2	.
27	38459936	38469125	9190	2	.
27	38469125	38476609	7485	2	.
27	38476609	38491560	14952	2	.
27	38491560	38500791	9232	2	.
27	38500791	38501597	807	2	.
27	38501597	38509392	7796	2	.
27	38509392	38509538	147	2	.
27	38509538	38510724	1187	2	.
27	38510724	38521911	11188	2	.
27	38523092	38530512	7421	2	.
27	38530512	38534818	4307	2	.
27	38534818	38537296	2479	2	.
27	38537296	38537973	678	2	.
27	38537973	38538783	811	2	.
27	38538783	38538912	130	2	.
27	38538912	38539701	790	2	.
27	38539701	38541123	1423	2	.
27	38541123	38559781	18659	2	.
27	38559781	38571874	12094	2	.
27	38571874	38573654	1781	2	.
27	38573654	38574873	1220	3	.
27	38574873	38575207	335	3	.
27	38575207	38575857	651	2	.
27	38575857	38585326	9470	2	.
27	38585326	38586720	1395	2	.
27	38586720	38587904	1185	2	.
27	38587904	38588641	738	2	.
27	38588641	38588788	148	2	.
27	38588788	38590409	1622	2	.
27	38590409	38591278	870	2	.
27	38591278	38594284	3007	2	.
27	38594284	38597955	3672	2	.
27	38597955	38600101	2147	2	.
27	38600101	38603612	3512	2	.
27	38603612	38604091	480	2	.
27	38673881	38674849	969	2	.
27	38674849	38675887	1039	2	.
27	38675887	38679439	3553	2	.
27	38679439	38689702	10264	2	.
27	38689702	38691279	1578	2	.
27	38691279	38701826	10548	2	.
27	38702212	38702903	692	2	.
27	38702903	38719043	16141	2	.
27	38719043	38722021	2979	2	.
27	38722021	38724414	2394	2	.
27	38724414	38726171	1758	2	.
27	38726171	38742663	16493	2	*PSD3*
27	38857761	38859757	1997	2	*PSD3*
27	39394600	39401315	6716	2	*LRRC3B*
27	39401315	39402951	1637	2	*LRRC3B*
27	39402951	39406429	3479	2	*LRRC3B*
27	39476968	39477355	388	2	*LRRC3B*
27	39529187	39529309	123	2	.
27	39760649	39761558	910	2	.
27	39785111	39785444	334	2	.
27	39841340	39843522	2183	2	.
27	39843522	39844175	654	2	.
27	39960489	39960753	265	2	.
27	39985140	39986278	1139	2	.
27	39986278	39986906	629	2	.
27	39986906	39987280	375	2	.
27	39987280	39987536	257	2	.
27	39987536	39991116	3581	2	.
27	39991116	39994662	3547	2	.
27	40033585	40034107	523	2	.
27	40034107	40034176	70	2	.
27	40034176	40034398	223	2	.
27	40046559	40047761	1203	2	.
28	45232	45747	516	2	.
28	92191	104300	12110	2	.
28	104300	108746	4447	2	.

28	146504	146678	175	2	.
28	264682	264877	196	2	.
28	297478	299311	1834	2	*CCSAP*
28	299311	308614	9304	2	*CCSAP*
28	308614	309218	605	2	*CCSAP*
28	309218	312503	3286	2	*CCSAP LOC107131917*
28	312503	329497	16995	2	*LOC107131917*
28	329497	332609	3113	2	*LOC107131917*
28	335315	338267	2953	2	*LOC107131917*
28	338267	339167	901	2	*LOC107131917*
28	339167	342070	2904	2	*LOC107131917*
28	342070	343224	1155	2	*LOC107131917*
28	343224	343688	465	2	*LOC107131917*
28	343688	348770	5083	2	.
28	348770	369750	20981	2	*LOC100848254*
28	369750	371582	1833	2	.
28	371582	372422	841	2	.
28	372422	382883	10462	2	*LOC789763*
28	382883	397069	14187	2	.
28	397069	405723	8655	2	.
28	405723	421534	15812	2	.
28	421534	431362	9829	2	.
28	431362	434607	3246	2	*ACTA1*
28	434607	435611	1005	2	.
28	435611	436190	580	2	.
28	436190	442350	6161	2	*NUP133*
28	442350	443507	1158	3	*NUP133*
28	443507	452557	9051	2	*NUP133*
28	452557	466570	14014	2	*NUP133*
28	466570	467836	1267	2	*NUP133*
28	467836	469226	1391	2	*NUP133*
28	469226	477194	7969	2	*NUP133*
28	477194	478043	850	2	*NUP133*
28	478043	478366	324	3	*NUP133*
28	478366	480983	2618	2	*NUP133*
28	480983	487027	6045	2	*NUP133*
28	487027	489569	2543	2	*NUP133*
28	489569	494506	4938	2	*LOC100140514 NUP133*
28	494506	495628	1123	2	*NUP133*
28	495628	499425	3798	2	*NUP133*
28	499425	507781	8357	2	*ABCB10*
28	507781	531978	24198	2	*ABCB10*
28	531978	532403	426	3	*ABCB10*
28	532403	548101	15699	2	*ABCB10*
28	548101	558763	10663	2	.
28	558763	559136	374	2	.
28	559136	560259	1124	2	.
28	560259	561549	1291	2	.
28	561549	609396	47848	2	*TAF5L URB2*
28	609396	624008	14613	2	*URB2*
28	624008	627840	3833	2	*URB2*
28	627840	635283	7444	2	*URB2*
28	635283	637011	1729	3	*URB2*
28	637011	642959	5949	2	.
28	642959	649178	6220	2	.
28	649178	676276	27099	2	.
28	676276	687965	11690	2	.
28	687965	688303	339	2	.
28	688303	689690	1388	2	.
28	689690	690086	397	2	.
28	690086	690576	491	2	.
28	690576	692397	1822	2	.
28	692397	693262	866	3	.
28	693262	694050	789	3	.
28	694050	703668	9619	2	.
28	703668	719237	15570	2	.
28	719237	721561	2325	2	.
28	721561	735583	14023	2	.
28	735583	752622	17040	2	.
28	752622	756172	3551	2	.
28	756172	777385	21214	2	.
28	777385	779853	2469	2	.
28	779853	785921	6069	2	.
28	785921	787302	1382	2	.
28	787302	799827	12526	2	*LOC783687*
28	799827	805530	5704	3	.
28	805530	812715	7186	3	.
28	812715	814038	1324	3	.
28	814038	824117	10080	3	.
28	824117	826786	2670	3	.
28	826786	829492	2707	2	.
28	829492	834016	4525	2	.
28	834016	845461	11446	2	.
28	845461	846037	577	2	.
28	846037	863230	17194	2	.
28	863230	868942	5713	2	.
28	868942	870144	1203	2	.
28	870144	870839	696	2	.
28	870839	874529	3691	2	.
28	874529	896793	22265	2	.
28	896793	898812	2020	2	.

28	898812	904007	5196	2	.
28	904007	904605	599	2	.
28	904605	913122	8518	2	.
28	913122	913994	873	2	.
28	913994	915607	1614	3	.
28	915607	916477	871	3	.
28	916477	918384	1908	2	.
28	918384	928005	9622	2	.
28	928005	950401	22397	2	.
28	950401	950528	128	2	.
28	950528	953069	2542	3	.
28	953069	964120	11052	3	.
28	964120	968894	4775	3	.
28	968894	1002610	33717	2	.
28	1004505	1013898	9394	2	.
28	1013898	1014152	255	2	.
28	1014152	1014220	69	2	.
28	1014220	1014945	726	2	.
28	1014945	1016828	1884	2	.
28	1016828	1020223	3396	2	.
28	1020223	1024605	4383	2	.
28	1024605	1025431	827	2	.
28	1025431	1028094	2664	2	.
28	1028094	1028787	694	2	.
28	1028787	1038665	9879	2	.
28	1038665	1039502	838	2	.
28	1039502	1055293	15792	2	.
28	1055293	1055833	541	3	.
28	1055833	1061066	5234	2	.
28	1061066	1062699	1634	2	.
28	1062699	1070553	7855	2	.
28	1070553	1070892	340	2	.
28	1070892	1075576	4685	2	.
28	1075576	1090881	15306	2	.
28	1090881	1102456	11576	2	.
28	1102456	1106619	4164	3	.
28	1106619	1135758	29140	2	.
28	1135758	1139178	3421	2	.
28	1139178	1187089	47912	2	*GALNT2*
28	1187089	1189778	2690	2	*GALNT2*
28	1189778	1218206	28429	2	*GALNT2*
28	1218206	1222776	4571	2	*GALNT2*
28	1222776	1222861	86	2	*GALNT2*
28	1222861	1244154	21294	2	*GALNT2*
28	1244154	1254058	9905	2	*GALNT2*
28	1254058	1264239	10182	2	*GALNT2*
28	1264239	1276872	12634	2	*GALNT2*
28	1276872	1290588	13717	2	*GALNT2*
28	1291072	1297324	6253	2	*GALNT2*
28	1297324	1313769	16446	2	*GALNT2*
28	1336807	1356648	19842	2	.
28	1356648	1357520	873	2	.
28	1357520	1360454	2935	3	.
28	1360454	1361679	1226	3	.
28	1361679	1373400	11722	3	*PGBD5*
28	1373400	1386643	13244	3	*PGBD5*
28	1386643	1387484	842	3	*PGBD5*
28	1387484	1392079	4596	3	*PGBD5*
28	1392079	1405164	13086	3	*PGBD5*
28	1405164	1414575	9412	3	*PGBD5*
28	1414575	1421620	7046	3	*PGBD5*
28	1421620	1421887	268	3	*PGBD5*
28	1421887	1423295	1409	3	*PGBD5*
28	1423295	1423502	208	3	*PGBD5*
28	1423502	1425058	1557	3	*PGBD5*
28	1425058	1426023	966	3	*PGBD5*
28	1426023	1433613	7591	3	*PGBD5*
28	1433613	1433793	181	3	*PGBD5*
28	1433793	1438663	4871	3	*PGBD5*
28	1438663	1438984	322	2	*PGBD5*
28	1438984	1442722	3739	3	*PGBD5*
28	1442722	1444261	1540	3	*PGBD5*
28	1444261	1448968	4708	3	*PGBD5*
28	1448968	1454106	5139	3	*PGBD5*
28	1454106	1455559	1454	4	*PGBD5*
28	1455559	1469877	14319	3	*LOC107133413 PGBD5*
28	1469877	1477834	7958	3	*LOC107133413*
28	1477834	1488227	10394	3	*LOC107133413*
28	1488227	1489127	901	3	*LOC107133413*
28	1489127	1489979	853	3	*LOC107133413*
28	1489979	1491738	1760	3	*LOC107133413*
28	1491738	1492052	315	4	*LOC107133413*
28	1492052	1502363	10312	3	*LOC107133413*
28	1502363	1503374	1012	2	*LOC107133413*
28	1503374	1504344	971	2	*LOC107133413*
28	1504344	1507418	3075	2	*LOC107133413*
28	1507418	1514543	7126	3	*LOC107133413*
28	1514543	1540512	25970	3	*LOC107133413*
28	1540512	1543087	2576	3	.
28	1543087	1548452	5366	3	.
28	1548452	1548793	342	3	.

28	1548793	1549844	1052	3	.
28	1549844	1551569	1726	3	.
28	1551569	1559852	8284	3	.
28	1559852	1560008	157	3	.
28	1560008	1561030	1023	3	.
28	1561030	1569965	8936	3	.
28	1569965	1570216	252	2	.
28	1570216	1572244	2029	3	.
28	1572244	1573357	1114	3	.
28	1573357	1580999	7643	4	.
28	1580999	1588128	7130	4	.
28	1588128	1591988	3861	3	.
28	1591988	1599770	7783	3	.
28	1599770	1600990	1221	3	.
28	1600990	1605811	4822	3	.
28	1605811	1608354	2544	3	.
28	1608354	1608508	155	3	.
28	1608508	1608818	311	3	.
28	1608818	1609460	643	4	.
28	1609460	1610493	1034	4	.
28	1610493	1614821	4329	3	.
28	1614821	1622002	7182	3	.
28	1622002	1631482	9481	3	.
28	1631482	1636431	4950	3	.
28	1636431	1636717	287	3	.
28	1636717	1638859	2143	4	.
28	1638859	1640623	1765	4	.
28	1640623	1646250	5628	3	.
28	1646250	1649995	3746	2	.
28	1649995	1659007	9013	2	.
28	1659007	1664502	5496	2	.
28	1664502	1670511	6010	2	.
28	1670511	1677943	7433	2	.
28	1677943	1691509	13567	2	.
28	1691509	1692077	569	2	.
28	1692077	1696099	4023	2	.
28	1696099	1696362	264	2	.
28	1696362	1701995	5634	2	.
28	1701995	1710580	8586	3	.
28	1710580	1718681	8102	3	.
28	1718681	1730759	12079	2	.
28	1730950	1731313	364	2	.
28	1731313	1745529	14217	3	.
28	1745529	1745715	187	3	.
28	1745715	1752544	6830	3	.
28	1752544	1756991	4448	3	.
28	1756991	1758438	1448	4	.
28	1758438	1763053	4616	3	.
28	1763053	1772775	9723	3	.
28	1772775	1773870	1096	2	.
28	1773870	1774751	882	2	.
28	1774751	1776985	2235	3	.
28	1776985	1777664	680	3	.
28	1777664	1790476	12813	3	.
28	1790476	1792252	1777	4	.
28	1792252	1798971	6720	3	.
28	1798971	1803816	4846	3	.
28	1803816	1804554	739	3	.
28	1804554	1805975	1422	3	.
28	1805975	1813776	7802	3	.
28	1813776	1814804	1029	3	.
28	1814804	1817992	3189	3	.
28	1817992	1823256	5265	3	.
28	1823256	1830054	6799	3	.
28	1830054	1871030	40977	3	.
28	1871030	1872169	1140	3	.
28	1872169	1877187	5019	3	.
28	1877187	1880179	2993	3	.
28	1880179	1889315	9137	3	.
28	1889315	1892486	3172	3	.
28	1892486	1892733	248	2	.
28	1892733	1896906	4174	2	.
28	1896906	1897124	219	2	.
28	1897124	1897328	205	2	.
28	1897328	1900117	2790	3	.
28	1900117	1904889	4773	3	.
28	1904889	1906377	1489	3	.
28	1906377	1916737	10361	3	.
28	2901077	2917744	16668	2	*LOC100336396*
28	2917744	2918984	1241	2	*LOC100336396*
28	2918984	2920092	1109	2	*LOC100336396*
28	2920092	2922656	2565	2	*LOC100336396 LOC100847909*
28	2922656	2937697	15042	2	*LOC100336396 LOC100847909*
28	2937697	2942412	4716	2	*LOC100336396*
28	2942412	2945311	2900	2	*LOC100336396*
28	2945311	2949643	4333	2	*LOC100336396*
28	2949643	2950376	734	2	*LOC100336396*
28	2950376	2952904	2529	2	*LOC100336396*
28	2952904	2953621	718	2	*LOC100336396*
28	2953621	2955160	1540	2	*LOC100336396*
28	2955160	2959274	4115	2	*LOC100336396*

28	2959274	2962600	3327	2	*LOC100336396*
28	2962600	2982774	20175	2	*LOC100336396*
28	2982774	2983978	1205	2	*LOC100336396*
28	2983978	2990836	6859	2	*LOC100336396*
28	2990836	2992912	2077	2	*LOC100336396*
28	2992912	2994982	2071	2	*LOC100336396*
28	2994982	2996933	1952	2	*LOC100336396*
28	2996933	2998645	1713	2	.
28	2998645	3024400	25756	2	.
28	3024400	3032096	7697	2	.
28	3032096	3033782	1687	2	.
28	3033782	3036602	2821	2	.
28	3036602	3046018	9417	2	.
28	3046018	3047152	1135	2	.
28	3047152	3059303	12152	2	.
28	3059303	3059844	542	2	.
28	3059844	3061976	2133	2	.
28	3061976	3068829	6854	2	.
28	3068829	3069664	836	2	.
28	3069664	3072454	2791	2	.
28	3072454	3074095	1642	3	*TSNAX*
28	3074095	3085037	10943	2	*TSNAX*
28	3085037	3089423	4387	2	*TSNAX*
28	3089423	3107411	17989	2	*TSNAX*
28	3107895	3113554	5660	2	*TSNAX*
28	3113554	3116630	3077	2	.
28	3116630	3118159	1530	2	.
28	3118159	3118500	342	2	.
28	3118500	3123732	5233	2	.
28	3123732	3125958	2227	2	.
28	3125958	3130152	4195	2	.
28	3130152	3131175	1024	3	.
28	3131175	3135239	4065	2	.
28	3135445	3137255	1811	2	.
28	3137255	3139022	1768	2	.
28	3139022	3145192	6171	2	.
28	3145192	3146314	1123	2	.
28	3146314	3146883	570	2	.
28	3146883	3149004	2122	2	.
28	3149004	3155973	6970	2	.
28	3155973	3156377	405	3	.
28	3156377	3167005	10629	2	.
28	3167005	3167867	863	2	.
28	3167867	3183763	15897	2	*LOC107131921*
28	3183763	3207827	24065	2	*LOC107131921 LOC533307*
28	3207827	3210667	2841	2	.
28	3210667	3235891	25225	2	*LOC784492*
28	3235891	3236642	752	2	.
28	3236642	3239684	3043	2	.
28	3239684	3240742	1059	2	.
28	3240742	3250265	9524	2	.
28	3250265	3267421	17157	3	.
28	3267421	3272244	4824	3	.
28	3272244	3273826	1583	4	.
28	3273826	3277196	3371	3	.
28	3277196	3297645	20450	2	.
**28**	**3297645**	**3318884**	**21240**	**2**	***EGLN1***
28	3318884	3341051	22168	3	.
28	3341051	3351556	10506	3	.
28	3351556	3354386	2831	3	.
28	3354386	3356258	1873	4	*LOC107131274*
28	3356258	3356657	400	4	*LOC107131274*
28	3356657	3357374	718	4	*LOC107131274*
28	3357374	3368991	11618	4	*LOC107131274*
28	3368991	3369469	479	5	.
28	3369469	3369654	186	4	.
28	3369654	3405166	35513	4	*EXOC8 SPRTN*
28	3405166	3406176	1011	3	.
28	3406176	3407061	886	4	.
28	3407061	3440753	33693	3	*GNPAT*
28	3440753	3445445	4693	3	*GNPAT*
28	3445445	3447690	2246	4	*GNPAT*
28	3447690	3449561	1872	3	*GNPAT*
28	3449561	3451366	1806	3	*C28H1orf131 GNPAT*
28	3451366	3451750	385	3	*C28H1orf131*
28	3451750	3452098	349	3	*C28H1orf131*
28	3452098	3452716	619	3	*C28H1orf131*
28	3452716	3454427	1712	3	*C28H1orf131*
28	3454427	3456444	2018	3	*C28H1orf131*
28	3456444	3471329	14886	3	*C28H1orf131*
28	3471329	3472538	1210	3	*C28H1orf131*
28	3472538	3475045	2508	3	*C28H1orf131*
28	3475045	3483251	8207	3	*C28H1orf131*
28	3483251	3483410	160	4	.
28	3483410	3488073	4664	3	.
28	3488073	3506774	18702	3	*TRIM67*
28	3506774	3507445	672	3	*TRIM67*
28	3507445	3507795	351	3	*TRIM67*
28	3507795	3513137	5343	3	*TRIM67*
28	3513137	3517090	3954	3	*TRIM67*
28	3517090	3522091	5002	3	*TRIM67*

28	3522091	3540964	18874	3	*TRIM67*
28	3540964	3541072	109	3	*TRIM67*
28	3541072	3542026	955	2	*TRIM67*
28	3542026	3542957	932	3	*TRIM67*
28	3542957	3564328	21372	3	*TRIM67*
28	3564328	3568864	4537	3	.
28	3568864	3569003	140	3	.
28	3569003	3571874	2872	3	.
28	3571874	3572233	360	3	.
28	3572233	3575788	3556	3	.
28	3575788	3576756	969	3	.
28	3576756	3578039	1284	3	.
28	3578039	3605534	27496	3	.
28	3605534	3617403	11870	3	.
28	3617403	3617500	98	3	.
28	3617500	3617975	476	3	.
28	3617975	3618348	374	4	.
28	3618348	3622374	4027	3	.
28	3622374	3631663	9290	3	.
28	3631663	3633196	1534	3	.
28	3633196	3645331	12136	3	.
28	3645331	3645480	150	3	.
28	3645480	3646717	1238	4	.
28	3646717	3648936	2220	3	.
28	3648936	3650581	1646	3	.
28	3650581	3669239	18659	3	.
28	3669239	3680191	10953	3	.
28	3680191	3689917	9727	3	*FAM89A*
28	3689917	3690075	159	3	*FAM89A*
28	3690075	3698760	8686	3	*FAM89A*
28	3698760	3705386	6627	3	*FAM89A*
28	3705386	3707911	2526	3	*FAM89A*
28	3707911	3708314	404	4	.
28	3708314	3708669	356	4	*LOC107131922*
28	3708669	3709339	671	3	*LOC107131922*
28	3709339	3709743	405	4	*LOC107131922*
28	3709743	3746252	36510	3	*ARV1 LOC107131922 TTC13*
28	3746252	3750644	4393	3	*TTC13*
28	3750644	3754836	4193	2	*TTC13*
28	3754836	3757019	2184	3	*TTC13*
28	3757019	3764244	7226	2	*TTC13*
28	3764244	3765933	1690	2	*TTC13*
28	3765933	3769178	3246	3	*TTC13*
28	3769178	3784859	15682	3	*TTC13*
28	3784859	3790507	5649	4	*TTC13*
28	3790507	3802623	12117	4	*TTC13*
28	3802623	3804299	1677	4	*TTC13*
28	3804299	3812973	8675	4	*TTC13*
28	3812973	3814240	1268	4	*TTC13*
28	3814240	3814667	428	4	*TTC13*
28	3814667	3814822	156	4	*TTC13*
28	3814822	3816819	1998	4	*TTC13*
28	3816819	3817223	405	3	*TTC13*
28	3817223	3817705	483	2	*TTC13*
28	3817705	3822506	4802	2	*TTC13*
28	3822506	3827442	4937	2	*TTC13*
28	3827442	3843491	16050	2	.
28	3843491	3854598	11108	2	.
28	3854598	3857184	2587	2	.
28	3857184	3858811	1628	2	.
28	3858811	3862542	3732	2	.
28	3862542	3884959	22418	2	*C28H1orf198*
28	3884959	3889005	4047	2	*C28H1orf198*
28	3889005	3891045	2041	2	*C28H1orf198*
28	3891045	3891270	226	2	*C28H1orf198*
28	3891270	3901794	10525	2	*C28H1orf198*
28	3901794	3910090	8297	2	.
28	3910090	3913663	3574	3	.
28	3913663	3920337	6675	2	.
28	3920337	3922853	2517	2	.
28	3922853	3928864	6012	2	.
28	3928864	3950307	21444	2	.
28	3950307	3950994	688	2	.
28	3950994	3975866	24873	2	.
28	3975866	3976406	541	3	.
28	3976406	3985726	9321	2	.
28	3985726	4005747	20022	3	.
28	4005747	4006322	576	3	.
28	4006322	4007595	1274	3	.
28	4007595	4022028	14434	3	.
28	4022028	4023219	1192	4	.
28	4023219	4029351	6133	3	.
28	4029351	4041472	12122	3	.
28	4041472	4041859	388	3	.
28	4041859	4052936	11078	3	.
28	4052936	4054964	2029	3	.
28	4054964	4060783	5820	3	.
28	4060783	4061273	491	3	.
28	4061273	4088179	26907	3	.
28	4088179	4090663	2485	3	.
28	4090663	4096674	6012	3	.

28	4096674	4116434	19761	3	.
28	4116434	4126042	9609	3	.
28	4126042	4128720	2679	3	.
28	4128720	4151700	22981	4	.
28	4151700	4151926	227	4	.
28	4151926	4152670	745	4	.
28	4152670	4156312	3643	3	.
28	4156312	4157904	1593	3	*FMN2*
28	4157904	4169893	11990	3	*FMN2*
28	4169893	4170760	868	3	*FMN2*
28	4170760	4172753	1994	3	*FMN2*
28	4172753	4173014	262	2	*FMN2*
28	4173014	4173463	450	2	*FMN2*
28	4173463	4194229	20767	2	*FMN2*
28	4194229	4203784	9556	2	*FMN2*
28	4203784	4241223	37440	2	*FMN2*
28	4241223	4241594	372	3	*FMN2*
28	4241594	4244208	2615	3	*FMN2*
28	4244208	4255498	11291	2	*FMN2*
28	4255498	4258047	2550	2	*FMN2*
28	4258047	4268219	10173	2	*FMN2*
28	4268219	4270099	1881	2	*FMN2*
28	4270296	4285856	15561	2	*FMN2*
28	4285856	4287256	1401	2	*FMN2*
28	4287256	4296180	8925	2	*FMN2*
28	4296180	4296605	426	2	*FMN2*
28	4296605	4299326	2722	2	*FMN2*
28	4301334	4306659	5326	2	*FMN2*
28	4306659	4307607	949	2	*FMN2*
28	4307607	4311565	3959	3	*FMN2*
28	4311565	4324935	13371	3	*FMN2*
28	4324935	4339152	14218	3	*FMN2*
28	4339152	4351342	12191	3	*FMN2*
28	4351342	4352017	676	4	*FMN2*
28	4352017	4357503	5487	3	*FMN2*
28	4357503	4358539	1037	3	*FMN2*
28	4358539	4365204	6666	3	*FMN2*
28	4365204	4365821	618	4	*FMN2*
28	4365821	4386356	20536	3	*FMN2*
28	4386356	4401263	14908	3	*FMN2*
28	4401263	4408757	7495	3	*FMN2*
28	4408757	4412999	4243	3	*FMN2*
28	4412999	4419658	6660	3	*FMN2*
28	4419658	4420476	819	3	*FMN2*
28	4420476	4420857	382	3	*FMN2*
28	4420857	4421202	346	4	*FMN2*
28	4421202	4423815	2614	3	*FMN2*
28	4423815	4424094	280	3	*FMN2*
28	4424094	4426079	1986	3	*FMN2*
28	4426079	4427204	1126	4	*FMN2*
28	4427204	4433899	6696	3	*FMN2*
28	4433899	4433976	78	4	*FMN2*
28	4433976	4439190	5215	3	*FMN2*
28	4439190	4454614	15425	3	*FMN2*
28	4454614	4456505	1892	3	*FMN2*
28	4456505	4457002	498	5	*FMN2*
28	4457002	4466284	9283	4	*FMN2*
28	4466284	4469840	3557	4	*FMN2*
28	4469840	4470369	530	4	*FMN2*
28	4470369	4474396	4028	4	*FMN2*
28	4474396	4476571	2176	4	*FMN2*
28	4476571	4487736	11166	4	*FMN2*
28	4487736	4494525	6790	3	*FMN2*
28	4494525	4494771	247	2	*FMN2*
28	4494771	4509976	15206	3	*FMN2*
28	4509976	4512831	2856	3	*FMN2*
28	4512831	4516088	3258	3	*FMN2*
28	4516088	4536045	19958	3	*FMN2*
28	4536045	4563816	27772	3	.
28	4563816	4574331	10516	3	.
28	4574331	4580269	5939	3	.
28	4580269	4616972	36704	3	*LOC100337323*
28	4616972	4626800	9829	2	*DISC1*
28	4626800	4648998	22199	2	*DISC1*
28	4648998	4649297	300	2	*DISC1*
28	4649297	4651006	1710	2	*DISC1*
28	4651006	4660523	9518	2	*DISC1*
28	4660523	4663972	3450	2	*DISC1*
28	4663972	4667098	3127	2	*DISC1*
28	4667098	4668531	1434	2	*DISC1*
28	4668531	4675762	7232	2	*DISC1*
28	4675762	4676351	590	2	*DISC1*
28	4676351	4677476	1126	2	*DISC1*
28	4677476	4681658	4183	2	*DISC1*
28	4681658	4683552	1895	2	*DISC1*
28	4683552	4689248	5697	2	*DISC1*
28	4689248	4691552	2305	2	*DISC1*
28	4691552	4703355	11804	2	*DISC1*
28	4704552	4738167	33616	2	*DISC1*
28	4738167	4738458	292	3	*DISC1*
28	4738458	4740775	2318	2	*DISC1*

28	4740775	4741261	487	2	*DISC1*
28	4741261	4748996	7736	2	*DISC1*
28	4748996	4749440	445	2	*DISC1*
28	4749440	4754665	5226	2	*DISC1*
28	4754665	4768667	14003	2	*DISC1*
28	4768667	4772139	3473	2	*DISC1*
28	4772139	4774677	2539	2	*DISC1*
28	5480087	5482115	2029	2	.
28	5482115	5482327	213	2	.
28	5482327	5497325	14999	2	.
28	5497325	5524794	27470	2	.
28	5524794	5531204	6411	2	.
28	5531204	5532055	852	2	.
28	5532055	5532652	598	3	.
28	5532652	5533886	1235	2	.
28	5533886	5540798	6913	2	.
28	5540798	5564691	23894	2	.
28	5564691	5576562	11872	2	.
28	5576562	5593636	17075	2	.
28	5593636	5594737	1102	2	.
28	5594737	5620008	25272	2	.
28	5620008	5623545	3538	2	.
28	5623545	5623801	257	2	.
28	5623801	5624986	1186	2	.
28	5624986	5629935	4950	2	.
28	5629935	5657815	27881	2	.
28	5657815	5667995	10181	2	.
28	5667995	5672029	4035	2	.
28	5672029	5678419	6391	2	.
28	5678419	5683574	5156	2	.
28	5683574	5685604	2031	2	.
28	5687766	5699304	11539	2	.
28	5699304	5701387	2084	2	.
28	5701387	5708457	7071	2	.
28	5708457	5709061	605	2	.
28	5709061	5709967	907	3	.
28	5709967	5710338	372	3	.
28	5710338	5710547	210	2	.
28	5710547	5711624	1078	2	.
28	5711624	5713545	1922	2	.
28	5713545	5720252	6708	2	.
28	5720252	5736478	16227	2	.
28	5736478	5749354	12877	2	.
28	5749354	5753647	4294	2	*MAP10*
28	5753647	5755295	1649	2	.
28	5755456	5759674	4219	2	.
28	5759674	5762319	2646	2	.
28	5762319	5762692	374	2	.
28	5762692	5772730	10039	2	.
28	5772730	5778751	6022	2	.
28	5778751	5788215	9465	2	.
28	5788215	5808600	20386	2	.
28	5808600	5831092	22493	2	.
28	5831092	5835242	4151	2	.
28	5835242	5844298	9057	2	.
28	5844298	5853794	9497	2	.
28	5853794	5856411	2618	2	.
28	5856411	5858568	2158	2	.
28	5858568	5886226	27659	2	.
28	5886226	5886667	442	2	.
28	5886667	5888224	1558	2	.
28	5888224	5907562	19339	2	.
28	5907562	5914024	6463	2	*NTPCR*
28	5914024	5915786	1763	2	*NTPCR*
28	5915786	5918192	2407	2	*NTPCR*
28	5918192	5918391	200	2	*NTPCR*
28	5918391	5955491	37101	2	*NTPCR PCNX2 TRNAC-GCA*
28	5955491	5955732	242	2	*PCNX2*
28	5955732	5964025	8294	2	*PCNX2*
28	5964025	5973204	9180	2	*PCNX2*
28	5973832	5976470	2639	2	*PCNX2*
28	5976470	5978681	2212	3	*PCNX2*
28	5978681	5979072	392	3	*PCNX2*
28	5979072	5983960	4889	3	*PCNX2*
28	5983960	5995294	11335	3	*PCNX2*
28	5995294	5996131	838	2	*PCNX2*
28	5996131	5999866	3736	2	*PCNX2*
28	5999866	6004227	4362	2	*PCNX2*
28	6004227	6009080	4854	2	*PCNX2*
28	6009080	6009701	622	2	*PCNX2*
28	6009701	6016858	7158	2	*PCNX2*
28	6016858	6036645	19788	2	*PCNX2*
28	6036645	6044378	7734	2	*PCNX2*
28	6044378	6045939	1562	2	*PCNX2*
28	6045939	6046559	621	2	*PCNX2*
28	6046559	6055505	8947	2	*PCNX2*
28	6055505	6067559	12055	2	*PCNX2*
28	6067559	6068936	1378	2	*PCNX2*
28	6068936	6069234	299	3	*PCNX2*
28	6069234	6071463	2230	2	*PCNX2*
28	6071463	6078463	7001	2	*PCNX2*

28	6078463	6108443	29981	2	*PCNX2*
28	9233322	9235976	2655	2	*EDARADD*
28	9235976	9250616	14641	2	*EDARADD*
28	9250616	9250881	266	3	*EDARADD*
28	9250881	9262931	12051	2	*EDARADD*
28	9262931	9268095	5165	3	*LGALS8*
28	9268095	9273343	5249	2	*LGALS8*
28	9273343	9276417	3075	2	*LGALS8*
28	9276417	9285671	9255	2	*LGALS8*
28	9285671	9291834	6164	2	*LGALS8*
28	9291834	9294719	2886	2	*LGALS8*
28	9294719	9298101	3383	2	*LGALS8*
28	9298101	9312768	14668	2	.
28	9312768	9324042	11275	2	.
28	9324042	9350899	26858	2	*HEATR1*
28	9350899	9358967	8069	2	.
28	9358967	9359850	884	2	.
28	9359850	9363656	3807	3	.
28	9363656	9363795	140	3	.
28	9363795	9365121	1327	3	.
28	9365121	9365360	240	3	.
28	9365360	9368135	2776	3	.
28	9368135	9373176	5042	3	.
28	9373176	9373962	787	3	.
28	9373962	9375706	1745	3	.
28	9375706	9376688	983	3	.
28	9376688	9376979	292	3	.
28	9376979	9378114	1136	3	.
28	9378114	9378280	167	3	.
28	9378280	9387946	9667	3	*LOC101908221*
28	9387946	9389015	1070	3	*LOC101908221*
28	9389015	9390456	1442	2	*LOC101908221*
28	9390456	9395350	4895	2	.
28	9395350	9395612	263	2	.
28	9395612	9397610	1999	2	.
28	9397610	9422889	25280	2	.
28	9422889	9430845	7957	2	.
28	9430845	9431246	402	2	.
28	9431246	9434482	3237	2	.
28	9434482	9442772	8291	2	.
28	9442772	9444620	1849	2	.
28	9444620	9457768	13149	2	.
28	9457768	9468818	11051	2	*ACTN2*
28	9468818	9468946	129	3	*ACTN2*
28	9468946	9469111	166	3	*ACTN2*
28	9469111	9477275	8165	2	*ACTN2*
28	9477275	9480495	3221	2	*ACTN2*
28	9480495	9481476	982	2	*ACTN2*
28	9481476	9482192	717	2	*ACTN2*
28	9482192	9483441	1250	2	*ACTN2*
28	9483441	9484903	1463	2	*ACTN2*
28	9484903	9489469	4567	2	*ACTN2*
28	9489469	9500962	11494	2	*ACTN2*
28	9500962	9504481	3520	2	*ACTN2*
28	9504481	9515440	10960	2	*ACTN2*
28	9515440	9516423	984	3	.
28	9516423	9516680	258	2	.
28	9516680	9522271	5592	2	.
28	9522271	9529288	7018	2	*MTR*
28	9529724	9558498	28775	2	*MTR*
28	9558498	9559772	1275	2	*MTR*
28	9559772	9582074	22303	2	*MTR*
28	9585087	9604558	19472	2	*MTR*
28	9608754	9611609	2856	2	*MTR*
28	9611609	9626097	14489	2	*MTR*
28	9626097	9626729	633	2	*MTR*
28	9626729	9630171	3443	2	*MTR*
28	9630171	9664869	34699	2	*MTR*
28	9664869	9670635	5767	2	.
28	9670635	9671246	612	2	.
28	9671246	9675039	3794	2	.
28	9675039	9676485	1447	2	.
28	9676485	9676877	393	2	.
28	9676877	9677626	750	2	.
28	9677626	9682107	4482	2	.
28	9682107	9683193	1087	3	.
28	9683193	9690335	7143	2	.
28	9690335	9705360	15026	2	.
28	9705360	9707213	1854	2	.
28	9708759	9712930	4172	2	.
28	9712930	9717122	4193	2	.
28	9717122	9717466	345	2	.
28	9717466	9719373	1908	2	.
28	9719373	9738149	18777	2	.
28	9738149	9738824	676	3	.
28	9738824	9746381	7558	2	.
28	9746381	9760884	14504	2	.
28	9760884	9782761	21878	3	.
28	9782761	9788521	5761	3	.
28	9788521	9796498	7978	2	.
28	9796498	9800098	3601	2	.

28	10354990	10358433	3444	2	*RYR2*
28	10358433	10364707	6275	2	*RYR2*
28	10364707	10365083	377	2	*RYR2*
28	10365083	10375564	10482	2	*RYR2*
28	10375564	10376188	625	2	*RYR2*
28	10376188	10376376	189	2	*RYR2*
28	10418805	10418924	120	2	*RYR2*
28	10418924	10425004	6081	2	*RYR2*
28	10425004	10426026	1023	2	*RYR2*
28	10426026	10435536	9511	2	*RYR2*
28	10504537	10510491	5955	2	*RYR2*
28	10510491	10512284	1794	2	*RYR2*
28	10512284	10521925	9642	2	*RYR2*
28	10521925	10527910	5986	2	*RYR2*
28	10527910	10530050	2141	2	*RYR2*
28	10530050	10530421	372	3	*RYR2*
28	10530421	10536290	5870	2	*RYR2*
28	10536290	10553326	17037	2	*RYR2*
28	10553326	10560747	7422	2	*RYR2*
28	10560747	10569767	9021	2	*RYR2*
28	10657252	10657923	672	2	.
28	10698760	10699191	432	2	.
28	10796698	10799585	2888	2	.
28	11987844	11989800	1957	2	.
28	12266638	12280583	13946	2	.
28	12280583	12308996	28414	2	.
28	12308996	12310492	1497	2	.
28	12310492	12311321	830	2	.
28	12311321	12313675	2355	2	.
28	12313675	12314414	740	2	.
28	12314414	12321187	6774	2	.
28	12321187	12325246	4060	2	.
28	12325246	12326438	1193	2	.
28	12326438	12331873	5436	2	.
28	12331873	12339041	7169	2	.
28	12339041	12352631	13591	2	.
28	12352631	12354624	1994	2	.
28	12354624	12355356	733	2	.
28	12355356	12356530	1175	2	.
28	12356530	12359746	3217	2	.
28	12359746	12371872	12127	2	.
28	12371872	12387344	15473	2	.
28	12387344	12387632	289	2	.
28	12387632	12388421	790	2	.
28	12388421	12391167	2747	2	.
28	12391167	12392654	1488	2	.
28	12392654	12393419	766	2	.
28	12393419	12394462	1044	2	.
28	12394462	12395544	1083	2	.
28	12395544	12405255	9712	2	.
28	12405255	12422753	17499	2	*TRNAR-ACG*
28	12422753	12449622	26870	2	.
28	12449622	12454491	4870	2	.
28	12454491	12456420	1930	2	.
28	12456420	12457569	1150	2	.
28	12457569	12461480	3912	2	.
28	12461480	12462448	969	2	.
28	12462448	12463889	1442	2	.
28	12463889	12464157	269	2	.
28	12464157	12466198	2042	2	.
28	12466198	12467516	1319	2	.
28	12467516	12477395	9880	2	.
28	12477395	12481089	3695	2	.
28	12481089	12483431	2343	2	.
28	12483431	12490964	7534	2	.
28	12490964	12495866	4903	2	.
28	12495866	12521184	25319	2	.
28	12521184	12529016	7833	2	.
28	12529016	12533179	4164	2	.
28	12533179	12559647	26469	2	.
28	12559647	12581492	21846	2	*CHRM3*
28	12581492	12585198	3707	2	*CHRM3*
28	12585198	12588281	3084	2	*CHRM3*
28	12588281	12593512	5232	2	*CHRM3*
28	12593512	12596470	2959	2	*CHRM3*
28	12596470	12599435	2966	2	*CHRM3*
28	12599435	12604624	5190	2	*CHRM3*
28	12604624	12621534	16911	2	*CHRM3*
28	12621534	12631323	9790	2	*CHRM3*
28	12631323	12635363	4041	2	*CHRM3*
28	12635363	12641018	5656	2	*CHRM3*
28	12641018	12646239	5222	2	*CHRM3*
28	12646239	12646545	307	2	*CHRM3*
28	12646545	12647533	989	2	*CHRM3*
28	12647533	12649852	2320	2	*CHRM3*
28	12649852	12667847	17996	2	*CHRM3*
28	12667847	12671885	4039	2	*CHRM3*
28	12671885	12672990	1106	2	*CHRM3*
28	12672990	12674590	1601	2	*CHRM3*
28	12674590	12676320	1731	2	*CHRM3*
28	12676320	12688222	11903	2	*CHRM3*

28	12688222	12691370	3149	2	*CHRM3*
28	12691370	12693655	2286	2	*CHRM3*
28	12693655	12699531	5877	2	*CHRM3*
28	12699531	12703660	4130	2	*CHRM3*
28	12703660	12708595	4936	2	*CHRM3*
28	12708595	12709056	462	2	*CHRM3*
28	12709056	12709982	927	2	*CHRM3*
28	12709982	12718929	8948	2	*CHRM3*
28	12718929	12727305	8377	2	*CHRM3*
28	12727305	12728004	700	2	*CHRM3*
28	12728004	12730445	2442	2	*CHRM3*
28	12730445	12732439	1995	2	*CHRM3*
28	12732439	12736778	4340	2	*CHRM3*
28	12736778	12739927	3150	2	*CHRM3*
28	12739927	12741118	1192	2	*CHRM3*
28	12741118	12744717	3600	2	*CHRM3*
28	12744717	12746198	1482	2	*CHRM3*
28	12746198	12749102	2905	2	*CHRM3*
28	12749102	12749778	677	2	*CHRM3*
28	12749778	12752235	2458	2	*CHRM3*
28	12752235	12768799	16565	2	*CHRM3*
28	12768799	12769059	261	2	*CHRM3*
28	12769059	12785624	16566	2	*CHRM3*
28	12785624	12789310	3687	2	*CHRM3*
28	12789310	12792377	3068	2	*CHRM3*
28	12792377	12793729	1353	2	*CHRM3*
28	12793729	12798717	4989	2	*CHRM3*
28	12798717	12799136	420	2	*CHRM3*
28	12799136	12816680	17545	2	*CHRM3*
28	12816680	12820024	3345	2	*CHRM3*
28	12820024	12829544	9521	2	*CHRM3*
28	12829544	12833842	4299	2	*CHRM3*
28	12833842	12835667	1826	2	*CHRM3*
28	12835667	12838125	2459	2	*CHRM3*
28	12838125	12848908	10784	2	*CHRM3*
28	12848908	12860029	11122	2	*CHRM3*
28	12860029	12871554	11526	2	*CHRM3*
28	12871554	12876542	4989	2	*CHRM3*
28	12876542	12878429	1888	2	*CHRM3*
28	12878429	12881059	2631	2	.
28	12881059	12882969	1911	2	.
28	13189439	13190149	711	2	*LOC101905074*
28	13190149	13190926	778	2	*LOC101905074*
28	13190926	13195054	4129	2	*LOC101905074*
28	13195054	13205785	10732	2	*LOC101905074*
28	13205785	13215498	9714	2	*LOC101905074*
28	13215498	13215883	386	2	.
28	13494813	13496349	1537	2	.
28	13496349	13505328	8980	2	.
28	13505328	13515255	9928	2	.
28	13515255	13515806	552	2	.
28	13515806	13516480	675	2	.
28	13516480	13517464	985	2	.
28	13566275	13567098	824	2	.
28	13645010	13645211	202	2	*RET*
28	13645211	13646096	886	2	*RET*
28	13646096	13646799	704	2	*RET*
28	13646799	13649466	2668	2	*RET*
28	13649466	13654872	5407	2	*RET*
28	13654872	13657516	2645	2	*RET*
28	13657516	13669967	12452	2	*RET*
28	13669967	13671635	1669	2	*RET*
28	13671635	13672202	568	2	*RET*
28	13672202	13674989	2788	2	*RET*
28	13674989	13685708	10720	2	*RET*
28	13685708	13695069	9362	2	.
28	13695069	13699454	4386	2	.
28	13699454	13711625	12172	2	.
28	13711625	13760143	48519	2	*CSGALNACT2 LOC104970943 RASGEF1A*
28	13760143	13764629	4487	2	*RASGEF1A*
28	13764629	13765669	1041	2	.
28	13765669	13766680	1012	2	.
28	13766680	13767555	876	2	.
28	13767555	13768225	671	2	.
28	13768225	13768983	759	2	.
28	13768983	13769638	656	3	.
28	13769638	13770234	597	2	.
28	13770234	13771706	1473	3	.
28	13771706	13776563	4858	2	.
28	13776563	13783698	7136	2	.
28	14667813	14669162	1350	2	.
28	14669162	14690783	21622	2	.
28	14690783	14708913	18131	2	.
28	14708913	14747323	38411	2	.
28	14772974	14775722	2749	2	.
28	15110141	15110564	424	2	*FAM13C*
28	15144921	15161067	16147	2	*FAM13C*
28	15161067	15161180	114	2	*FAM13C*
28	15161180	15164226	3047	2	*FAM13C*
28	15164226	15166398	2173	2	*FAM13C*
28	16427365	16432299	4935	2	*LOC100337251*

28	16432299	16434281	1983	2	*LOC100337251*
28	16434281	16438913	4633	2	*LOC100337251 TRNAG-UCC*
28	16438913	16450701	11789	2	*LOC100337251*
28	16450701	16451854	1154	2	*LOC100337251*
28	16451854	16457743	5890	2	*LOC100337251*
28	16457743	16461785	4043	2	*LOC100337251*
28	16461785	16473344	11560	2	*LOC100337251*
28	16473344	16473740	397	2	*LOC100337251*
28	16473740	16485016	11277	2	*LOC100337251*
28	16485016	16485192	177	2	*LOC100337251*
28	16485192	16498527	13336	2	*LOC100337251*
28	16498527	16518037	19511	2	*LOC100337251*
28	16518037	16520941	2905	2	*LOC100337251*
28	16520941	16521339	399	2	*LOC100337251*
28	16521339	16522156	818	2	*LOC100337251*
28	16522156	16523620	1465	2	*LOC100337251*
28	16523620	16524236	617	2	*LOC100337251*
28	16524236	16524465	230	2	*LOC100337251*
28	16524465	16525446	982	2	*LOC100337251*
28	16525446	16530733	5288	2	*LOC100337251*
28	16530733	16560997	30265	2	*LOC100337251*
28	16560997	16566375	5379	2	*LOC100337251*
28	16566375	16567570	1196	2	*LOC100337251*
28	16567570	16584196	16627	2	*LOC100337251*
28	16584196	16585506	1311	4	*LOC100337251*
28	16585506	16585728	223	3	*LOC100337251*
28	16585728	16592271	6544	2	*LOC100337251*
28	16592271	16610822	18552	2	*LOC100337251*
28	16610822	16614050	3229	2	*LOC100337251*
28	16614050	16614480	431	2	*LOC100337251*
28	16614480	16616264	1785	2	*LOC100337251*
28	16616264	16617362	1099	2	*LOC100337251*
28	16617362	16617575	214	2	*LOC100337251*
28	16617575	16618331	757	2	*LOC100337251*
28	16618331	16627148	8818	2	*LOC100337251*
28	16627148	16633969	6822	2	*LOC100337251*
28	16633969	16634787	819	2	*LOC100337251*
28	16634787	16639883	5097	2	*LOC100337251*
28	16639883	16640701	819	2	*LOC100337251*
28	16640701	16641884	1184	2	*LOC100337251*
28	16641884	16644976	3093	2	*LOC100337251*
28	16644976	16657261	12286	3	*LOC100337251*
28	16657261	16670801	13541	3	.
28	16670801	16709611	38811	3	*CDK1*
28	16709611	16712326	2716	3	.
28	16712326	16717599	5274	3	.
28	16717599	16725096	7498	3	.
28	16725096	16727510	2415	3	.
28	16727510	16729681	2172	4	*LOC512594*
28	16729681	16730222	542	3	*LOC512594*
28	16730222	16737576	7355	3	*LOC512594*
28	16737576	16738463	888	4	.
28	16738463	16743159	4697	3	.
28	16743159	16754011	10853	3	.
28	16754011	16775284	21274	3	.
28	16775284	16784705	9422	3	.
28	16784705	16784806	102	3	.
28	16784806	16786929	2124	3	.
28	16786929	16787328	400	3	.
28	16787328	16792612	5285	3	.
28	16792612	16811932	19321	3	*RHOBTB1*
28	16811932	16813103	1172	3	*RHOBTB1*
28	16813103	16816813	3711	3	*RHOBTB1*
28	16816813	16826724	9912	3	*RHOBTB1*
28	16826724	16829296	2573	4	*RHOBTB1*
28	16829296	16836320	7025	3	*RHOBTB1*
28	16836320	16848855	12536	3	*RHOBTB1*
28	16848855	16860111	11257	3	*RHOBTB1*
28	16860111	16860477	367	4	*RHOBTB1*
28	16860477	16886393	25917	3	*RHOBTB1*
28	16886393	16890297	3905	3	.
28	16890297	16891134	838	3	.
28	16891134	16903508	12375	3	.
28	16903508	16906227	2720	3	.
28	16906227	16908701	2475	3	.
28	16908701	16910337	1637	3	.
28	16910337	16917553	7217	3	.
28	16917553	16919602	2050	2	.
28	19073578	19074019	442	2	.
28	19074019	19075343	1325	2	.
28	20106752	20107123	372	2	.
28	20255717	20259587	3871	2	.
28	20259587	20265168	5582	2	.
28	20265168	20269105	3938	2	.
28	20269105	20270029	925	2	.
28	20270029	20273244	3216	2	.
28	20273244	20274889	1646	2	.
28	20274889	20275910	1022	2	.
28	20275910	20285298	9389	2	.
28	20398147	20398460	314	2	.
28	20419017	20423011	3995	2	.

28	20423011	20424600	1590	2	.
28	20424600	20434825	10226	2	.
28	20434825	20436284	1460	2	.
28	20436284	20438983	2700	2	.
28	20438983	20439789	807	2	.
28	20439789	20440265	477	2	.
28	20440265	20450426	10162	2	.
28	20530796	20531797	1002	2	.
28	20531797	20532071	275	2	.
28	20532071	20535404	3334	2	.
28	20535404	20539190	3787	2	.
28	20539190	20539392	203	2	.
28	20539392	20547451	8060	2	.
28	20547451	20552953	5503	2	.
28	20552953	20563123	10171	2	.
28	20563123	20563416	294	2	.
28	20563416	20563677	262	2	.
28	20563677	20565776	2100	2	.
28	20565776	20568118	2343	2	.
28	20568118	20592056	23939	2	.
28	20592056	20592877	822	2	.
28	20592877	20593283	407	2	.
28	20654331	20656201	1871	2	.
28	20710161	20710768	608	2	.
28	20710768	20711106	339	2	.
28	20711106	20711433	328	2	.
28	20711433	20712957	1525	2	.
28	20712957	20714540	1584	2	.
28	20714540	20718959	4420	2	.
28	20718959	20719178	220	2	.
28	20719178	20726543	7366	2	.
28	20776355	20777266	912	2	.
28	34300447	34307012	6566	5	.
28	34307012	34309277	2266	5	.
28	34309277	34327359	18083	6	.
28	34327359	34331733	4375	6	.
28	34331733	34332362	630	6	.
28	34332362	34334507	2146	5	.
28	34383663	34384145	483	4	.
28	34384145	34392206	8062	4	*LOC104969725*
28	34392206	34392817	612	5	.
28	34392817	34407091	14275	6	.
28	34407091	34410398	3308	6	.
28	34410398	34411131	734	6	.
28	34411131	34411976	846	6	.
28	34411976	34413397	1422	6	.
28	34413397	34415719	2323	6	.
28	34415719	34420806	5088	6	.
28	34420806	34421897	1092	6	.
28	34421897	34441730	19834	6	.
28	34441730	34444914	3185	7	.
28	34444914	34461206	16293	6	.
28	34461206	34461546	341	5	.
28	34461546	34464360	2815	7	.
28	34464360	34464911	552	7	.
28	34464911	34465263	353	7	.
28	34465263	34465885	623	7	.
28	34465885	34466441	557	6	.
28	34466441	34467030	590	6	.
28	34467030	34467721	692	6	.
28	34467721	34470909	3189	6	.
28	34470909	34486885	15977	6	.
28	34486885	34487005	121	5	.
28	34487005	34489090	2086	6	.
28	34489090	34492446	3357	6	.
28	34492446	34493452	1007	7	.
28	34493452	34506225	12774	6	.
28	34506225	34509251	3027	6	.
28	34509251	34512379	3129	6	.
28	34512379	34516253	3875	6	.
28	34516253	34517639	1387	6	.
28	34517639	34518944	1306	6	.
28	34518944	34526055	7112	6	.
28	34526055	34535217	9163	6	.
28	34535217	34538756	3540	6	.
28	34538756	34556115	17360	6	.
28	34556115	34563340	7226	6	.
28	34563340	34566325	2986	6	.
29	109	121883	121775	3	*LOC101905316 LOC104969772*
29	121883	877969	756087	2	*HEPHL1 LOC100297728 LOC104970173 LOC104976209 LOC107131344 LOC516870 LOC789688 PANX1*
29	877969	1503538	625570	3	*C29H11orf54 CCDC67 CEP295 LOC100140332 LOC101906859 LOC107131948 LOC784357 MED17 SMCO4 TAF1D VSTM5*

Supplementary Table 28. Information of regions of yak introgression into Tibetan taurine cattle associted with hypoxia

Chr	Start (bp)	End (bp)	Size (bp)	Hap (n)	Gene content of the introgressed interval	Biological process
11	46348628	47006375	657748	6	*CKAP2L* ***IL1A IL1B*** *IL1F10 IL1RN IL36A IL36B IL36G IL36RN IL37 LOC101903687 LOC101904177 LOC104973371 LOC107132933 LOC107132936 NT5DC4 PAX8 PSD4 SLC20A1*	HIF pathway candidate genes
14	33359502	33670520	311019	2	*ARFGEF1* ***COPS5*** *CSPP1 PPP1R42 TCF24*	HIF pathway candidate genes
15	22695068	22988949	293882	2	*BCO2 C15H11orf1 C15H11orf52 C15H11orf57 CRYAB DIXDC1 DLAT HSPB2 IL18 LOC101903059 LOC101903125 LOC101906594 LOC104968480 LOC104974189 PIH1D2* ***SDHD*** *TEX12 TIMM8B*	Hypoxia should cause a significant increase in the concentration of intracellular reactive oxygen species (ROS) in pulmonary cells, and accordingly ROS should then lead to large increase in intracellular concentration of Ca^{2+} mediated by multiple ion channels. Both ROS and Ca^{2+} are important signaling molecules that regulate the response to hypoxia.
28	3297645	3318884	21240	2	***EGLN1***	HIF pathway candidate genes
28	10354990	10358433	3444	2	***RYR2***	RYR2, a ryanodine receptor, functions to release Ca^{2+} and plays a crucial role in pulmonary vasoconstriction during hypoxia to ensure regional alveolar ventilation and pulmonary perfusion
28	10358433	10364707	6275	2		
28	10364707	10365083	377	2		
28	10365083	10375564	10482	2		
28	10375564	10376188	625	2		
28	10376188	10376376	189	2		
28	10418805	10418924	120	2		
28	10418924	10425004	6081	2		
28	10425004	10426026	1023	2		
28	10426026	10435536	9511	2		
28	10504537	10510491	5955	2		
28	10510491	10512284	1794	2		
28	10512284	10521925	9642	2		
28	10521925	10527910	5986	2		
28	10527910	10530050	2141	2		
28	10530050	10530421	372	3		
28	10530421	10536290	5870	2		
28	10536290	10553326	17037	2		
28	10553326	10560747	7422	2		
28	10560747	10569767	9021	2		

Supplementary Table 29. Proportion of missing alleles in specific SNPs of different species of the tribe of Bovini.

Samples	Species	Missing alleles	Specific SNPs	Missing alleles in specific SNPs	Proportion of missing alleles in specific SNPs
Banteng01	*Bos javanicus*	2,385,779	4,551,280	175,760	0.038618
Banteng02	*Bos javanicus*	1,629,512	4,551,280	20,771	0.004564
Bision01	*Bison bision*	2,306,628	2,460,180	152,341	0.061923
Bision02	*Bison bision*	415,949	2,460,180	3,490	0.001419
Wisent01	*Bison bonasus*	1,457,280	2,477,513	84,589	0.034143
Wisent02	*Bison bonasus*	434,223	2,477,513	10,486	0.004232
Wisent03	*Bison bonasus*	534,416	2,477,513	13,235	0.005342
Yak01	*Bos grunniens*	623,929	3,092,273	29,831	0.009647
Yak02	*Bos grunniens*	580,270	3,092,273	26,409	0.00854
Yak03	*Bos grunniens*	4,135,174	3,092,273	391,329	0.126551
Yak04	*Bos grunniens*	2,436,909	3,092,273	242,469	0.078411
Yak05	*Bos grunniens*	1,332,805	3,092,273	112,896	0.036509
Yak06	*Bos grunniens*	1,018,264	3,092,273	78,608	0.025421
Yak07	*Bos grunniens*	1,341,774	3,092,273	113,086	0.036571
Yak08	*Bos grunniens*	1,742,468	3,092,273	159,483	0.051575
Yak09	*Bos grunniens*	1,243,638	3,092,273	103,064	0.03333
Yak10	*Bos grunniens*	573,124	3,092,273	26,325	0.008513
Yak11	*Bos grunniens*	5,717,308	3,092,273	466,308	0.150798
Yak12	*Bos grunniens*	935,004	3,092,273	40,851	0.013211
Yak13	*Bos grunniens*	551,396	3,092,273	27,053	0.008749
Gaurus01	*Bos gaurus*	6,281,380	3,999,129	576,628	0.144188
Gaurus02	*Bos gaurus*	2,798,378	3,999,129	109,727	0.027438
Buffalo01	*Bubalus bubalis*	4,543,003	2,241,071	37,147	0.016576
Buffalo02	*Bubalus bubalis*	4,546,969	2,241,071	38,238	0.017062

Supplementary Table 30. Parameters and results inferred by *∂a∂i* simulations

Events	Item symbols	Confidence interval (95%)		Point estimation
		2.50%	97.50%	
Ancestral population size	N.nuPre	20660.62	39352.4	22546.81492
Time0	T.Tpre	2863.039	6008.068	5429.796944
Population size of Chinese indicine cattle between Time0-Time1	N.nu1_0	822.5144	1217.9633	952.6081903
Population size of Indian indicine cattle between Time0-Time2	N.nu2_0	32908.68	45725.18	43839.00953
Population size of Chinese indicine cattle between Time1-Time4 (present)	N.nu1_1	29524.11	30995.31	29899.05347
Population size of Indian indicine cattle between Time3-Time4 (present)	N.nu2_1	11235.79	12976.05	11879.62965
Time1: the first split between Chinese indicine cattle and Indian indicine cattle	T.T1	30299.51	52677.4	40149.97
Time2: population size expansion of Chinese indicine cattle	T.T2	23709.87	40703	32015.99
Time3: population size decline of Indian indicine cattle	T.T3	9248.557	12923.49	11542.99
Migration from Indian indicine cattle into Chinese indicine cattle between Time1-Time2	M.m12_1	0.000144426	0.000291131	0.000244922
Migration from Chinese indicine cattle into Indian indicine cattle between Time1-Time2	M.m21_1	1.70E-08	1.43E-05	6.36E-06
Migration from Indian indicine cattle into Chinese indicine cattle between Time2-Time3	M.m12_2	8.36E-05	1.64E-04	0.000129225
Migration from Chinese indicine cattle into Indian indicine cattle between Time2-Time3	M.m21_2	9.77E-05	2.00E-04	0.000123741
Migration from Indian indicine cattle into Chinese indicine cattle between Time3-Time4 (present)	M.m12_3	5.30E-08	2.85E-06	2.26E-06
Migration from Chinese indicine cattle into Indian indicine cattle between Time3-Time4 (present)	M.m21_3	1.63E-08	2.51E-07	2.30E-07

Supplementary Note 1

Whole-genome resequencing

In total, 111 DNA samples of Chinese native cattle representing 22 breeds and three DNA samples of Indian indicine breeds were collected. Paired-end libraries were generated for each individual using standard procedures. The average insert size was 500 bp, and the read length was 150 bp. All libraries were sequenced on an Illumina HiSeq 2000 platform to an average raw read sequence coverage of 10 X, assuming a genome size of 2.87 Gb. The average coverage was approximately 11.88 X (ranging from 8.29 X to 36.85 X). Such depth ensured the accuracy of variant calling and genotyping, and met the requirements for population genetic analyses. Additional detailed information on the mapping rate and sequencing depth are provided in Supplementary Table 1.

Definition of geographic regions discussed in the text

We also combined our data with those from 146 available whole-genome resequencing samples from 24 breeds worldwide[1-6], including 83 European taurine cattle, 26 Northeast Asian taurine cattle, eight Iranian cattle, 17 American indicine cattle, and 12 African cattle. The sequencing coverage was approximately 13.37 X (ranging from 3.92 X to 25.26 X) per individual. According to the sample information and the origin of cattle breeds, ten geographic regions were defined (Fig. 1a and Supplementary Table 2). We also used 25 whole-genome data from extant domestic and wild species within Bovini, including gaur, bison, wisent, banteng, gayal, yak, and buffalo for outgroup and introgression analysis[1,7-10]. The average coverage was approximately 11.17 X (ranging from 4.12 X to 38.96 X) per individual.

Variant discovery and genotyping

A total of 260 samples were used as final set for variant discovery. First, all cleaned reads were mapped to the cattle reference assembly Btau_5.0.1 (GCF_000003205.7) using BWA-MEM (0.7.13-r1126) with default parameters[11]. The average mapping rate was 98.88%, and the sequencing coverage was approximately 12.72 X. Duplicate reads were removed using Picard Tools (http://broadinstitute.github.io/picard/). Then, the Genome Analysis Toolkit (GATK, version 3.6-0-g89b7209) was used to detect single nucleotide polymorphisms (SNPs)[12]. The following criteria were applied to all SNPs: (1) SNPs mean sequencing depth (over all included individuals) < 1/3X and > 3X were filtered; (2) SNPs with Variant Confidence/Quality by Depth (QD) < 2 were filtered; (3) SNPs with RMS Mapping Quality (MQ) < 40.0 were filtered; (4) SNPs with Phred-scaled *P*-value using Fisher's exact test to detect strand bias (FS) > 60 were filtered; (5) SNPs with Z-score according to the Wilcoxon rank sum test of Alt vs. Ref read mapping qualities (MQRankSum) < -12.5 were filtered; (6) SNPs with Z-score according to the Wilcoxon rank sum test of Alt vs. Ref read position bias (ReadPosRankSum) < -8 were filtered; (7) SNPs with maximum missing rate < 0.1; and (8) SNPs

with only two alleles. A total of 60.4 million autosomal SNPs were identified and used in subsequent analyses. The identified SNPs were further classified based on the gene annotation for the reference genome. SNPs were categorized as variants in intergenic regions, 5′-UTRs, coding sequences (exon), introns, 3′-UTRs and noncoding regions. SNPs in coding sequences were further grouped as synonymous SNPs not causing amino acid changes or nonsynonymous SNPs causing amino acid changes. In addition, nonsense mutations were defined as mutations causing premature stops, elongated transcripts or introduced false start codons. The distribution of SNPs within various genomic regions is shown in Supplementary Table 3.

The whole-genome data from seven extant species were mapped in the same way. We used 60.4 million SNPs as reference list to genotype the combined set of 260 cattle samples and 25 samples of seven extant Bovini species. The final genotype data were imputed and phased using BEAGLE (version 4.1).

Supplementary Note 2

Population genomic analyses

Principle component analysis (PCA)

VCF files encoding individual genotypes were generated. This merged VCF file containing a total of 60,449,904 variants was converted to PLINK format with VCFtools v0.1.12 (https://vcftools.github.io/index.html)[13]. SNPs were filtered based on a minor allele frequency (MAF) > 0.0057 to insure at least three alleles were found in sample set. We removed "all LD" using the --indep-pairwise 50 5 0.2 option of PLINK(version 1.9)[14]. The same SNPs set was used for PCA, TreeMix, and ADMIXTURE analysis. The PCA was performed based on the genome-wide unlinked SNP dataset using the software Eigensoft (version 4.2)[15]. The Tracy-Widom test was used to determine the significance level of the eigenvectors. This package was used with various functions to perform the PCA on the genetic data to analyse the population structure. The results were plotted with ggplot2 in R 3.02[16]. Three groups were used for the PCA: (1) all 260 animals were used as first group, with the first three principal components cumulatively explaining 7.40 % of the total variance (Fig. 1b and 1c, Supplementary Table 4); Within *Bos indicus*, a clear partitioning was apparent between cattle from India and South China (Fig. 1c). (2) And 142 individuals from 21 *Bos taurus* breeds, including 13 European breeds (Angus, Red Angus, Charolais, Devon, Gelbvieh, Hereford, Holstein, Jersey, Limousin, Maine Anjou, Piedmontese, Salers, and Simmental), three Northwest Chinese breeds (Chaidamu, Kazakh, and Mongolian), four East Asian breeds (Hanwoo, Kuchinoshima, Mishima, and Yanbian), and Tibetan cattle (Supplementary Fig. 1 and Supplementary Table 5); (3) Fifty individuals of *Bos indicus* from 13 breeds, including six Indian indicine breeds (Hariana, Sahiwal, Tharparkar, Brahman, Gir, and Nelore) and seven Chinese indicine breeds (Guangfeng, Jian, Jingjiang, Leiqiong,

Wannan, Wenshan, and Dianzhong) (Supplementary Fig. 1 and Supplementary Table 6). The first PC separates Indian indicine and Chinese indicine cattle. The second component displays the contrast of Nelore to other indicine cattle. The result was consist with the PCA of all samples. The genotype likelihood approach was also used to repeat the PCA result (Supplementary Fig. 3).

Neighbour-joining (NJ) tree and maximum likelihood (ML) phylogenetic trees

To identify closely related individuals, a total of 60,449,904 autosomal SNPs were selected to construct a NJ tree with PLINK using the matrix of pairwise genetic distances. Two banteng samples were rooted as outgroup (Fig. 1d). The pairwise distances used for building the NJ tree given in Supplementary Data 1. Then we inferred a population-level phylogeny using the ML approach implemented in TreeMix. We removed "all LD" using the --indep-pairwise 50 5 0.2 option of PLINK[14]. The window size of 1000 was used to account for linkage disequilibrium (-k) and "-global" to generate the ML tree (Supplementary Fig. 4). MEGA v7.0 and FigTree (http://tree.bio.ed.ac.uk/software/figtree/) were used to visualize the phylogenetic trees.

Structure analysis

To quantify the genome-wide admixture among modern cattle populations, we estimated the ancestry of each individual using the genome-wide unlinked SNP dataset used for the PCA with the model-based assignment program ADMIXTURE 1.3[17]. ADMIXTURE was run for each possible group number (K = 2 to 8), and 200 bootstrap replicates were performed to estimate the parameter standard errors, which were used to determine the optimal group number (K) (Supplementary Table 7). Surprisingly, number of presumed ancestral populations (K) showed genetically distinct clusters that reflect geographic proximity. We found strong support for two population subdivisions (K = 2), which was consistent with the deep division of the *Bos taurus* and *Bos indicus* lineages. The population subdivision at K = 3 reproduces the second PCA coordinated by separating the European cattle (West Europe and Central-South Europe) and East Asian (Tibetan and Northeast Asian) cattle. The population subdivision at K = 4 reproduces the third PCA coordinated by separating the cattle of South China from other indicine samples. The Chinese indicine cattle (Leiqiong, Jingjiang, Guangfeng, Ji'an, Wannan, and Wenshan) were clearly separated from the Indian indicine cattle (Hariana, Sahiwal, Tharparkar, Brahman, Gir, and Nelore) at K = 4 (Supplementary Fig. 5). Population subdivisions at K = 5 produced different *Bos taurus* groups and revealed a strong clustering of taurine cattle into three main clusters: European taurine ancestry (Hereford and Angus), Eurasian taurine ancestry (Piedmontese, Gelbvieh, Limousin, and Simmental), and East Asian taurine ancestry (Tibetan, Mishima, Kuchinoshima, Hanwoo, and Yanbian). We noticed that Tibetan and Northeast Asian breeds constituted a distinct group referred as East Asian taurine cattle. We also detected Indian indicine ancestry in Northwest and Southwest China, which could provide evidence for two immigration route of indicine cattle from India into China. Cattle from Middle East showed hybrid ancestries. To ensure

no bias in the results, NgsAdmix was used to repeat the ADMIXTURE result (Supplementary Fig. 6). The result of K= 5 is consistent with the result of ADMIXTURE, except that the Holstein cattle have both ancestries of European taurine and Eurasian taurine cattle.

Supplementary Note 3

Paternal analysis

The bovine male-specific region (MSY) consists of three major regions: X-degenerate (Xd), Y-transitional (Yt), and Y-ampliconic (Ya). The Xd region, split into two sub-regions at either end of the MSY, spans from 2.5-3.9 Mb (Xd1) and 42.2-43.3 Mb (Xd2). We only selected the Xd regions of the Btau_5.0.1 Y chromosome reference sequence (GCF_000003205.7)[18]. All analyses in this part were restricted to this region. We called genotypes as described in Supplementary Note 1. Thirty-one female cattle were used as controls. We also removed heterozygous sites and sites with missing genotypes in 10% of the sampled individuals. Missing calls ranged from 0 to 328 per individual. Final SNPs were filtered based on an MAF of 0.002. After performing quality control and filtering, we extracted 745 SNPs, and 69 were located in the *USP9Y*, *UTY*, and *ZFY* genes. The genotypes of 745 SNPs are shown in Supplementary Data 2. We used 745 SNPs in 214 individuals to construct a haplogroup tree, which was rooted using the yak sequence. Sequence alignments were built using CLUSTALW2 (http://www.clustal.org/), and a maximum parsimony (MP) phylogenetic tree was created using the software PHYLIP (http://evolution.gs.washington.edu/phylip.html) (Supplementary Fig. 7a). We used BEAGLE to impute missing alleles[19] and checked them according to the tree structure. The only errors introduced by this procedure will be missed reversions or recurrent mutations, both are rare. We then used imputed sequences to build a MP tree (Supplementary Fig. 7b). Additional phylogenetic trees were then inferred using both ML and Bayesian methods. A ML analysis was conducted with MEGA 7.0 (Supplementary Fig. 7c). We also performed Bayesian phylogenetic inferences using BEAST 1.8.2[20]. The Bayesian phylogenetic inferences were run for 50,000,000 generations, the chain was sampled every 5,000 generations (thin-in interval), and the first 10% of the chains was discarded as burn-in. We analysed the MCMC samples with TRACER 1.5, which indicated convergence and adequate mixing of the Markov chains. We then used TREEANNOTATOR 1.7.5 to summarize the MCMC samples as the maximum clade credibility topology. The tree drawn with Figtree v1.4.2 (http://tree.bio.ed.ac.uk/software/figtree/) is shown in Supplementary Fig. 7d. We also constructed a network using imputed data (Fig. 3a).

Whole mitochondrial genome phylogeny

Following the procedure described in Supplementary Note 1, we mapped the sequencing reads of all 260 modern cattle (Supplementary Table 1) against the reference bovine mitochondrial genome (GenBank: AY526085.1). To assemble the complete mitochondrial genome, samples with a

depth-of-coverage lower than 100 X were disregarded, which resulted in a total of 147 mitochondrial genomes (Supplementary Table 8). The average depth-of-coverage was 1152.04 X and ranged from 138.77 to 3626.71 X. BAM alignments were converted to fastq and subsequently used with Mapping Iterative Assembler V 1.0 (MIA) to assemble a mtDNA consensus sequence.

We aligned our 147 mitochondrial genome sequences to a collection of 24 published *genus Bos* and *Bison* mitochondrial genomes[21-24]. This collection encompasses most of the mitochondrial haplotypes present in cattle (Supplementary Table 8). The TrN+G+I model of nucleotide substitution was selected by comparing the Bayesian information criterion (BIC) scores in ModelGenerator v0.85[25]. A phylogenetic tree was then inferred using both ML and Bayesian methods. A ML analysis was performed with the program PhyML 3.0[26] using both NNI and SPR rearrangements to search for the tree topology, and approximate likelihood-ratio tests were performed to establish statistical support of internal branches (Supplementary Fig. 8a). We also performed Bayesian phylogenetic inferences using BEAST 1.8.2[20]. The Bayesian phylogenetic inferences were run for 50,000,000 generations, the chain was sampled every 5,000 generations (thin-in interval), and the first 10% of the chains was discarded as burn-in. We analysed the MCMC samples with TRACER 1.5, which indicated convergence and adequate mixing of the Markov chains. We then used TREEANNOTATOR 1.7.5 to summarize the MCMC samples as the maximum clade credibility topology. The tree drawn with Figtree v1.4.2 (http://tree.bio.ed.ac.uk/software/figtree) is shown in Supplementary Fig. 8b along with the corresponding node support values (posterior probabilities).

Consistent with previous reports that identified T4 haplogroup only in Northeast Asian cattle, the T4 mtDNA haplogroup was observed in our samples[23].

Supplementary Note 4

Details on the archaeological specimens

The Shimao archaeological site, which belongs to the Longshan period, is located in the town of Gaojiabao, which is approximately 40 km southwest of Shenmu County in Yulin District, Shaanxi Province, China. The large scale of this site (4,000,000 m^2), the skilled construction technique, and the integrated defence system contributed to the outstanding quality of the Shimao site among the Neolithic walled settlements in East Asia[27]. Eight bovine bones analysed in this study include a radius (left), tibia (right), tibia (left), metacarpal (left), humerus (right), metapodial (left), radius (left), and metapodial (right) (named Shimao01, Shimao02, Shimao03, Shimao04, Shimao05, Shimao06, Shimao07, and Shimao08, respectively). The humerus bone from specimen Shimao05 (Supplementary Fig. 9) was directly radiocarbon dated at the Beta Analytic Radiocarbon Dating Laboratory, Miami, Florida, and it was dated conservatively to 3,975-3,835 cal BP with a 95% confidence interval (Supplementary Table 9). These dates are consistent with previous radiocarbon determinations from Shimao, which dated the site to ca. 2300-1800 cal. BCE[27].

Ancient genome data processing

Sample preparation, extraction, and library preparation were performed in a dedicated ancient DNA laboratory in the Kunming Institute of Zoology, Yunnan, China. Paired-end sequencing data were generated using the Illumina® HiSeq X Ten platform at the Beijing Genome Institute (BGI), Shenzhen, China using 150 bp reads. Adapter sequences of the paired-end reads were identified and removed using AdapterRemoval version 2.2.0. We merged read pairs where the expected index was observed, and they required an overlap of at least 11 bp. All processed sequences were mapped to the Btau 5.0.1 reference genome using BWA version 0.7.12-r1039 with the parameters aln -l 1024, and PCR duplicate reads with identical start and end coordinates were collapsed into consensus sequences using SAMtools version 1.3.1. Then, we counted the mismatch using ANGSD with "-doMisMatch" option, and ploted the damage pattern (Supplementary Fig. 10). All ancient samples were genotyped according to the SNP set of all modern samples using the following criteria: (1) minimum Phred-scaled mapping quality of 20; (2) minimum base quality score of 30; (3) minimum fraction of reads supporting heterozygous of 0.2; and (4) maximum DP of 10. Finally, all sites containing transitions were removed because all ancient samples have post mortem damage that strongly increases the errors at sites that contain transitions. We obtained low-coverage genome sequences (0.01-2.63 average depth, overall average equal to 0.40) from eight ancient Shimao cattle (Supplementary Table 10).

To investigate the relationship of the Shimao specimens to current East Asian cattle populations, we selected "core" groups of the five components based on the structure according to AdmixTools for the phylogenetic analysis. We also used three-population test estimates (*f3* statistics) to test for admixtures across all modern cattle populations[28]. Three-population tests consider population triplets (C; A, B), where C is the test population and A and B are the reference populations. The Z-scores were calculated by jack-knifing with blocks of 445 SNPs. Significantly negative Z-scores ($Z \leq -3.80$, after Bonferroni correction for multiple testing) indicate evidence of test population C containing an admixture of both reference populations of A and B. The high number of significant *f3* statistics suggested an admixture for two breeds (Mongolian and Kazakh) from Northwest China (Supplementary Table 11), suggesting an additional southwestern immigration route of indicine cattle from India into northwest China in recent time. All African breeds exhibited evidence of *Bos taurus* and *Bos indicus* ancestries (Supplementary Table 12). Supplementary Table 13 shows examples of the most significant *f3* statistics for various cattle breeds of North-Central China, with Chinese indicine (Leiqiong) as one of the source populations. We performed all possible triplet combinations (Supplementary Table 14). Finally, a total of 109 individuals from the five “core” groups were selected (Supplementary Table 15), a neighbour-joining tree was constructed following the procedure described in Supplementary Note 2, and it was based on the pairwise distance matrix data calculated by PLINK for the five "core" group samples and eight ancient samples (Fig. 4a)[14]. The

results showed that the ancient samples were closely related to Hanwoo and Japanese cattle, indicating that the ancient cattle belonged to the East Asian taurine group.

To further investigate the gene flow between Shimao cattle and different worldwide populations, the *f3* statistics for (indicine cattle; Ancient, population B) were quantified for a set of 42 worldwide populations using ~50 M SNPs. Indicine group are three individuals of Hariana, Sahiwal, and Tharparkar cattle. The highest *f*3 statistics were observed for Northeast Asian cattle (Kuchinoshima, Yanbian, Hanwoo, and Mishima), followed by the West Europe and Central-South Europe populations (Fig. 4b).

We also used *D* statistics to test whether ancient Shimao cattle shared more alleles with East Asian taurine or with European taurine cattle[29]. The *D* statistics method considers the tree topology (((W, X), Y), O), where O represents the outgroup, Y represents the ancient population, the pattern of ABBA reflects gene flow between W and X, and the pattern BABA reflects gene flow between W and Y. The *D* statistics method quantifies the occurrence of two incomplete sorting patterns called ABBA and BABA events, where W and X refer to ancestral (namely, identical to O) and derived allelic states. ABBA events occur when X and Y share the derived allele (B) and W carries the ancestral allele (A). Conversely, in BABA events, the ancestral allele (A) is carried by X while the derived allele is shared by W and Y. Under the null hypothesis that the tree is correct and there is no gene flow connecting Y to either W or X, the ABBA and BABA events result from incomplete lineage sorting and thus should occur with equal frequency. The enrichment of ABBA or BABA sites, therefore, indicates the possible presence of gene flow between the X and Y or W and Y lineages, respectively. To comprehensively assess the amount of gene flow between the cattle lineages, we calculated multiple *D* statistics corresponding to different taxa combinations as W, X or Y compatible with the inferred tree topology (Supplementary Table 16). Buffalo was used as the outgroup species. Using *D* statistics, we confirmed that ancient Shimao cattle shared more derived polymorphisms with the East Asian taurine, except Tibetan taurine cattle (Supplementary Fig. 11).

Supplementary Note 5

Introgression analysis

The nucleotide diversity (π) of all groups were calculated using a sliding window approach with windows of 50-kb. The nucleotide diversity of five "core" groups revealed that Chinese indicine genomes showed the highest nucleotide diversity among the five "core" groups (Fig. 4c and Supplementary Fig. 12). To detected the effects of different coverages on analysis. We measured nucleotide diversity at group level again using genotype likelihood approach. For autosomal chromosomes, we estimated the SFS with ANGSD (-doSaf2). The nucleotide diversity were calculated with –doThetas. The result also recapitulated that Chinese indicine genomes showed the highest nucleotide diversity (Supplementary Fig. 13). The comparisons also showed that Chinese indicine

cattle and *Bos javanicus* shared the most SNPs (~4.7 M), followed by *Bos taurus* (~3.7 M) *and Bos frontalis* (~3.5 M) (Supplementary Table 17), suggesting interspecies introgressions in the history of Chinese indicine cattle. We hypothesised that this increased unique diversity could be influenced by particular (but unknown) historical demography such as population expansion but the scale of this unique diversity suggests hybridization with or introgression from different bovine species. So we used a combination of analyses, including *f*3 statistics, *D* statistics and Treemix, to test this introgression hypothesis. We ran *f*3 statistics on population triples using Chinese indicine cattle as a target (admixture population) and Indian indicine cattle and other Bovini species as the source populations, including two gaur, two bison, three wisent, two banteng, one gayal, 13 yaks, and two buffaloes (Supplementary Table 2). We used the KING software[30] to evaluate the relatedness of samples within each *Bos* species, including gaur, bison, wisent, banteng, and yak. The two banteng were not within a 3rd-degree relationship (Kinship = 0.0072). Negative kinship coefficients estimates indicated that the other *Bos* samples were unrelated.

Our test using banteng and Indian indicine cattle as the sources produced a remarkably significant Z-score of -26.97, followed by gayal (Supplementary Table 18). *D* statistics tests were applied following the tree topology (Buffalo, Chinese indicine cattle; Banteng, other Bovini species), and the results suggested that Chinese indicine cattle was possibly introgressed from banteng, followed by gayal (Supplementary Table 19). TreeMix also confirmed the gene flow from *Bos javanicus* into Chinese indicine cattle and the introgression from yak into Tibetan taurine cattle (Supplementary Fig. 14). Chinese Yunnan gayal, also named Dulong cattle, originated from the hybridization between male *Bos frontalis* and female *Bos taurus* or *Bos indicus*[31]. Consider that there was only one individual of gayal and it showed gene flow from Chinese indicine cattle into *Bos frontalis* in TreeMix. The introgression from *Bos frontalis* in Chinese indicine cattle needs further confirmation.

Possible Banteng introgression into Chinese indicine cattle

We then used the method for the analysis of introgressive hybridization of Mongolian yak[32] to confirm *Bos javanicus* introgression into Chinese indicine and yak introgression into Tibetan taurine cattle (Fig. 5). To detect introgression from banteng into Chinese indicine cattle, we first used two banteng genomes to identify banteng-specific alleles. Then, 5-kb sliding windows were used to calculate the mean frequency of banteng and indicine alleles in the Chinese indicine cattle and the frequency of two types of alleles were plotted (Supplementary Fig. 15). To identify introgressed intervals in Chinese indicine genomes, we applied a robust forward-backward algorithm for all autosomes of Chinese indicine genomes using the software RFMix[33]. Five other genetic groups were selected as reference panels, including European taurine, Eurasian taurine, and Eastern Asian taurine, Indian indicine as well as banteng. The proportion of the genome inferred to be of *Bos javanicus* ancestry ranged between 2.38% and 3.84% (mean ± standard error (SE) = 2.92 ± 0.11%) per animal

(Supplementary Table 20). Phylogenetic analysis based on the SNPs in 13 introgression intervals in 15 or more Chinese indicine haploids revealed a close genetic relationship between the Chinese indicine cattle and *Bos javanicus* (Supplementary Fig. 16 and Supplementary Table 21).

Annotation of the gene content of the introgressed segments

Gene set enrichment analyses were carried out with Gene Ontology (GO) categories and Kyoto Encyclopaedia of Genes and Genomes (KEGG) pathways for DAVID 6.7 (http://david.abcc.ncifcrf.gov/). To provide a first overview of the over-represented groups of genes and to test their reliability, we performed different GO and KEGG pathway enrichment analyses with DAVID using different lists of genes located in chromosomal regions detected as introgressed from banteng to Chinese indicine and form yak to Tibetan taurine cattle by RFMix analyses (results are presented in Table 1). Only pathways or annotations with Bonferroni-corrected $P < 10^{-2}$ were retained (Supplementary Table 22). We also identified several introgressed genes that favor local adaptation to hot environments in Chinese indicine cattle (Supplementary Table 23). Phylogenetic analyses of introgressed segments with signs of banteng ancestry in Chinese indicine genomes are shown Supplementary Fig. 19.

Yak introgression in Tibetan taurine cattle

To detect introgressions from yak into Tibetan taurine cattle, we used 13 individual genomes of yak to detected yak-specific alternate alleles and plotted the frequency of the yak and bovine alleles for all positions. Then, 5-kb sliding windows were used to calculate the mean frequency of yak and bovine alleles in the Tibetan taurine group (Supplementary Fig. 17). *f*3 statistics, *D* statistics and Treemix analyses also confirmed the gene flow from yak to Tibetan taurine cattle (Supplementary Fig. 14 and Supplementary Tables 24, 25). To identify introgressed intervals in Tibetan taurine genomes, we applied a robust forward-backward algorithm for all autosomes of Tibetan genomes using the software RFMix[33]. Five other genetic groups were selected as reference panels, including European taurine, Eastern Asian taurine, Indian indicine, and Chinese indicine as well as yak. The genome proportion inferred to be of yak ancestry ranged between 0.05% and 2.94% (mean ± standard error (SE) = 1.22 ± 0.18 %) per animal (Supplementary Table 26). Phylogenetic analysis based on the SNPs in 30 introgression intervals in eight or more Tibetan taurine haploids revealed a close genetic relationship between the Tibetan taurine cattle and yak (Supplementary Fig. 18 and Supplementary Table 27). In addition, we were able to retrieve hypoxia genes in introgressed regions that might help Tibetan taurine cattle adapt to hypoxic environments (Supplementary Fig. 20 and Supplementary Table 28).

Effect of sample size on introgression analysis

We also explored how sample size can impact the results of introgression analysis, especially for a species with only two individuals. Thirteen samples of yak in our study could be used to simulate the

effects of sample size on the results of introgression from yak into Tibetan taurine cattle. Although we cannot directly evaluate whether two banteng were sufficient, we can estimate the effects of sample sizes on the detection of yak introgression into taurine cattle since we have 13 yak available. In our study, a total of 4,238 introgressed segments (~246 Mb) were detected using the 13 yak reference. If two randomly selected yak individuals were used for introgression analysis (totally 78 combinations from 13 samples), the detection ratio of introgressed segments is >99% for highly frequent segments (ie., ≥ two alleles in nine Tibetan taurine cattle) and can still reaches 75% for low frequent ones (one allele in nine Tibetan taurine cattle). In our study, the mean proportion of the genome inferred to be of *Bos javanicus* ancestry was 2.92 ± 0.11 %, with 2.61 ± 0.10 % showing at least two alleles. So we believe that our two banteng samples should be sufficient for the detection of introgressed segments, especially for the highly confident regions with at least two shared alleles in indicine cattle. In addition, only introgressed segments that shared at least two alleles in Chinese indicine cattle were used for the functional enrichment and introgression time analysis. We also calculated the proportion of missing genotypes in specific SNPs in different *Bos genus* species. The result showed that the missing genotypes have lower proportion in specific SNPs of different *Bos* species. So imputing the lower coverage samples have little effect on RFMix analysis (Supplementary Table 29).

Timing of introgression

To date the banteng introgression, we selected a total of 13 non-coding regions introgressed in 15 or more Chinese indicine haploids (Supplementary Fig. 16). The divergence time of Chinese indicine cattle and banteng on these regions was used to represent the time of introgression. The software bpp3.3a was used to estimate the divergence time under the multispecies coalescent model on a fixed species phylogeny with the following Gamma priors: $\theta \sim G(2, 700)$, $\tau \sim G(20, 4,000,000)$[34]. The number of samples used in the MCMC algorithms was 100,000, and the sampling frequency was 10. The estimated mean divergence time (θ) was 0.000005, and 95% highest probability density (HPD) intervals were from 0.000003 to 0.000007, which were scaled to an absolute time of 2,381 years (1,428 years to 3,333 years, 95% HPD), with a mutation rate of $\mu_g = 1.26\times10^{-8}$ per generation[35] and a generation of six years[10].

To date the yak introgressions, we selected 30 non-coding regions introgressed into eight or more Tibetan taurine haploids (Supplementary Fig. 18). The divergence time of Tibetan taurine cattle and yak on these regions was used to represent the time of introgression. The software bpp3.3a was used to estimate the divergence time under the multispecies coalescent model on a fixed species phylogeny with the following Gamma priors: $\theta{\sim}G (2, 700)$, $\tau{\sim}G (20, 6,800,000)$[34]. The number of samples used in MCMC algorithms was 100,000, and the sampling frequency was 10. The estimated mean divergence time (θ) was 0.000005, and the 95% HPD intervals were from 0.000003 to 0.000007, which were scaled to an absolute time of 1,904 years (1,428 years to 2,380 years, 95% HPD), with a mutation rate of $\mu_g = 1.26\times10^{-8}$ per generation[35] and a generation interval of six years[10].

Supplementary Note 6

Estimates of the effective population size and divergence time

The multiple sequential coalescent Markovian model (MSMC) method was used to model the history of the five genetic groups and infer historical changes in the effective population size and population separation. We applied this method to all groups with two deep-coverage (>15 X) individuals per group. For each individual, we identified SNPs in the autosomes using GATK, and sites with extremely low or extremely high coverage were excluded. All sites were phased using BEAGLE (version 4.1)[19]. For effective population size inference, two individuals (4 phased haplotypes) from each population were used. The time scale is in generation time at g = 6, and a mutation rate per generation $\mu_g = 1.26\times10^{-8}$ was used[10,35].

Samples (coverage) used in this analysis are:

- East Asian taurine: Tibetan03 (27.06) , Tibetan04 (25.36)
- European taurine: Hereford06 (16.12), Hereford07 (17.34)
- European taurine: Gelbvieh03 (15.72), Gelbvieh04 (25.26)
- Chinese indicine: Wannan01 (23.96), Wannan02 (24.32)
- Indian indicine: Hariana (36.85), Sahiwal (21.99)

We define the estimated divergence time between a pair of populations as the first time point at which the cross-coalescence rate is at or above 0.5. For the range of divergence times, we used the first time point at which the cross-coalescence rate was at or above 0.25 and 0.75. We also assessed the impact of introgression on the estimates of effective population size (N_e) and divergence time in MSMC. We repeated the MSMC analysis using the same data but excluding the genomic regions representing banteng introgression into two Chinese indicine cattle (~3.5%) and yak introgression in two Tibetan taurine cattle (~1.3%), respectively (Supplementary Tables 20 and 26). The results showed that the limited introgression did not change the estimates of divergence time and N_e too much (Supplementary Fig. 21).

We also used diffusion approximations for demographic inference (∂a∂i)[36] to infer the divergence time between Chinese indicine and Indian indicine cattle. We simulated four models with the same dataset under the two-population model in ∂a∂i independently. Of the SNPs identified in 17 Chinese indicine and 20 Indian indicine individuals, we only considered those from intergenic regions in autosomal sequences to ensure their neutrality. Because the introgression happened in Chinese indicine cattle may disturb the analysis, here we used only non-introgressed regions to produce allele frequency spectra (AFS) data. The introgressed regions detected by RFMix were excluded firstly. Then we calculate number of SNPs that shared with *Bos javanicus* in every 10 k interval on whole-genome, and intervals contain no *Bos javanicus*-specific SNPs were remained. Only 56 M intergenic regions were used to produce AFS data using ANGSD software. The simulation of ∂a∂i showed that the Chinese indicine and Indian indicine populations diverged ~40,149 years ago

(~32,299 to 52,677 years ago, 95% CI; Fig. 6c, Supplementary Fig. 22 and Supplementary Table 30), which gave rise to today's pattern of two genetically distinct indicine populations.

Supplementary References

1. Heaton, M.P. *et al.* Using diverse U.S. beef cattle genomes to identify missense mutations in EPAS1, a gene associated with pulmonary hypertension. *F1000Research* **5**, 2003 (2016).
2. Shin, D.-H. *et al.* Deleted copy number variation of Hanwoo and Holstein using next generation sequencing at the population level. *BMC Genomics* **15**, 240 (2014).
3. Bickhart, D.M. *et al.* Diversity and population-genetic properties of copy number variations and multicopy genes in cattle. *DNA Res.* **23**, 253-262 (2016).
4. Tsuda, K. *et al.* Abundant sequence divergence in the native Japanese cattle Mishima-Ushi (*Bos taurus*) detected using whole-genome sequencing. *Genomics* **102**, 372-378 (2013).
5. Kawahara-Miki, R. *et al.* Whole-genome resequencing shows numerous genes with nonsynonymous SNPs in the Japanese native cattle Kuchinoshima-Ushi. *BMC Genomics* **12**, 103 (2011).
6. Stothard, P. *et al.* A large and diverse collection of bovine genome sequences from the Canadian Cattle Genome Project. *GigaScience* **4**, 49 (2015).
7. Wang, K. *et al.* The genome sequence of the wisent (*Bison bonasus*). *GigaScience* **6**, 1-5 (2017).
8. Qiu, Q. *et al.* Yak whole-genome resequencing reveals domestication signatures and prehistoric population expansions. *Nat. Commun.* **6**, 10283 (2015).
9. Mei, C. *et al.* Whole-genome sequencing of the endangered bovine species Gayal (*Bos frontalis*) provides new insights into its genetic features. *Sci. Rep.* **6**, 19787 (2016).
10. Gautier, M. *et al.* Deciphering the Wisent demographic and adaptive histories from individual whole-genome sequences. *Mol. Biol. Evol.* **33**, 2801-2814 (2016).
11. Li, H. & Durbin, R. Fast and accurate short read alignment with Burrows–Wheeler transform. *Bioinformatics* **25**, 1754-1760 (2009).
12. McKenna, A. *et* al. The Genome Analysis Toolkit: A MapReduce framework for analyzing next-generation DNA sequencing data. *Genome Res*. 20, 1297-1303 (2010).
13. Danecek, P. *et al.* The variant call format and VCFtools. *Bioinformatics* **27**, 2156-2158 (2011).
14. Purcell, S. *et al.* PLINK: A tool set for whole-genome association and population-based linkage analyses. *Am. J. Hum. Genet.* **81**, 559-575 (2007).
15. Patterson, N., Price, A.L. & Reich, D. Population Structure and Eigenanalysis. *PLoS Genet.* **2**, e190 (2006).
16. Wickham, H. ggplot2. *Wiley Interdisciplinary Reviews: Computational Statistics* **3**, 180-185 (2011).
17. Alexander, D.H., Novembre, J. & Lange, K. Fast model-based estimation of ancestry in

unrelated individuals. *Genome Res.* **19**, 1655-1664 (2009).

18. Chang, T.-C., Yang, Y., Retzel, E.F. & Liu, W.-S. Male-specific region of the bovine Y chromosome is gene rich with a high transcriptomic activity in testis development. *Proc. Natl. Acad. Sci. USA* **110**, 12373-12378 (2013).
19. Browning, S.R. & Browning, B.L. Rapid and accurate haplotype phasing and missing-data inference for whole-genome association studies by use of localized haplotype clustering. *Am. J. Hum. Genet.* **81**, 1084-1097 (2007).
20. Drummond, A.J., Suchard, M.A., Xie, D. & Rambaut, A. Bayesian Phylogenetics with BEAUti and the BEAST 1.7. *Mol. Biol. Evol.* **29**, 1969-1973 (2012).
21. Edwards, C.J. *et al.* A complete mitochondrial genome sequence from a mesolithic wild aurochs (*Bos primigenius*). *PLoS One* **5**, e9255 (2010).
22. Achilli, A. *et al.* The multifaceted origin of taurine cattle reflected by the mitochondrial genome. *PLoS One* **4**, e5753 (2009).
23. Achilli, A. *et al.* Mitochondrial genomes of extinct aurochs survive in domestic cattle. *Curr. Biol.* **18**, R157-R158 (2008).
24. Hiendleder, S., Lewalski, H. & Janke, A. Complete mitochondrial genomes of *Bos taurus* and *Bos indicus* provide new insights into intra-species variation, taxonomy and domestication. *Cytogenet. Genome Res.* **120**, 150-156 (2008).
25. Keane, T.M., Creevey, C.J., Pentony, M.M., Naughton, T.J. & McLnerney, J.O. Assessment of methods for amino acid matrix selection and their use on empirical data shows that ad hoc assumptions for choice of matrix are not justified. *BMC Evol. Biol.* **6**, 29 (2006).
26. Guindon, S. *et al.* New algorithms and methods to estimate maximum-likelihood phylogenies: assessing the performance of PhyML 3.0. *Syst. Biol.* **59**, 307-321 (2010).
27. Sun, Z. *et al.* The first Neolithic urban center on China's north Loess Plateau: The rise and fall of Shimao. *Archaeological Research in Asia* (2017).
28. Patterson, N. *et al.* Ancient admixture in human history. *Genetics* **192**, 1065 (2012).
29. Green, R.E. *et al.* A draft sequence of the Neandertal genome. *Science* **328**, 710 (2010).
30. Manichaikul, A. et al. Robust relationship inference in genome-wide association studies. *Bioinformatics* 26, 2867-2873 (2010).
31. Gou, X., Wang, Y., Yang, S., Deng, W. & Mao, H. Genetic diversity and origin of Gayal and cattle in Yunnan revealed by mtDNA control region and SRY gene sequence variation. *J. Anim. Breed. Genet.* **127**, 154-160 (2010).
32. Medugorac, I. *et al.* Whole-genome analysis of introgressive hybridization and characterization of the bovine legacy of Mongolian yaks. *Nat. Genet.* **49**, 470-475 (2017).
33. Maples, B.K., Gravel, S., Kenny, E.E. & Bustamante, C.D. RFMix: A discriminative modeling approach for rapid and robust local-ancestry inference. *Am. J. Hum. Genet.* **93**, 278-88 (2013).
34. Yang, Z. The BPP program for species tree estimation and species delimitation. *Current*

Zoology **61**, 854-865 (2015).

35. Liu, Y. *et al. Bos taurus* genome assembly. *BMC Genomics* **10**, 180 (2009).

37. Gutenkunst, R.N., Hernandez, R.D., Williamson, S.H. & Bustamante, C.D. Inferring the joint demographic history of multiple populations from multidimensional SNP frequency data. *PLoS Genet.* **5**, e1000695 (2009).
