# Supplementary material for: Whole-genome resequencing reveals world-wide ancestry and adaptive introgression events of domesticated cattle in East Asia
Source: Nat Commun. 2018 Jun 14;9:2337. doi: 10.1038/s41467-018-04737-0 (PMC6002414; doi:10.1038/s41467-018-04737-0)
Supplement: Supplementary file 3 — Description of Additional Supplementary Files [file 41467_2018_4737_MOESM3_ESM.pdf]

Description of Additional Supplementary Files

File Name: Supplementary Data 1
Description: Pariwise distance of 260 individuals.

File Name: Supplementary Data 2
Description: The genotype of 745 SNPs in X-degenerate region.
